# Molecular Detection of Colistin Resistance *mcr*-*1* Gene in Multidrug-Resistant *Escherichia coli* Isolated from Chicken

**DOI:** 10.3390/antibiotics11010097

**Published:** 2022-01-13

**Authors:** Md Bashir Uddin, Mohammad Nurul Alam, Mahmudul Hasan, S. M. Bayejed Hossain, Mita Debnath, Ruhena Begum, Mohammed A. Samad, Syeda Farjana Hoque, Md. Shahidur Rahman Chowdhury, Md. Mahfujur Rahman, Md. Mukter Hossain, Mohammad Mahmudul Hassan, Åke Lundkvist, Josef D. Järhult, Mohamed E. El Zowalaty, Syed Sayeem Uddin Ahmed

**Affiliations:** 1 Department of Medicine, Sylhet Agricultural University, Sylhet 3100, Bangladesh; dr.nurul_alam@ymail.com (M.N.A.); dr.bayejed@gmail.com (S.M.B.H.); shahidur.vetmed@sau.ac.bd (M.S.R.C.); mahfuj.vetmed@sau.ac.bd (M.M.R.); mukter.vetmed@sau.ac.bd (M.M.H.); 2 Department of Pharmaceuticals and Industrial Biotechnology, Sylhet Agricultural University, Sylhet 3100, Bangladesh; mhasan.pib@sau.ac.bd (M.H.); farjananasid@gmail.com (S.F.H.); 3 Kazi Farms Poultry Laboratory, Gazipur 1700, Bangladesh; debnath.mita@gmail.com; 4 Bangladesh Livestock Research Institute (BLRI), Savar 1341, Bangladesh; dr.ruhenabegum@gmail.com (R.B.); samad_blri@yahoo.co.nz (M.A.S.); 5 Department of Physiology, Biochemistry and Pharmacology, Chattogram Veterinary and Animal Sciences University, Chattogram 4225, Bangladesh; miladhasan@yahoo.com; 6 Department of Medical Biochemistry and Microbiology, Zoonosis Science Center, Uppsala University, SE 75 123 Uppsala, Sweden; ake.lundkvist@imbim.uu.se; 7 Department of Medical Sciences, Zoonosis Science Center, Uppsala University, SE 75 123 Uppsala, Sweden; josef.jarhult@medsci.uu.se; 8 Department of Epidemiology and Public Health, Sylhet Agricultural University, Sylhet 3100, Bangladesh

**Keywords:** *Escherichia coli*, antimicrobial resistance, PCR, *mcr-1* gene, poultry

## Abstract

Zoonotic and antimicrobial-resistant *Escherichia coli* (hereafter, *E. coli*) is a global public health threat which can lead to detrimental effects on human health. Here, we aim to investigate the antimicrobial resistance and the presence of *mcr-1* gene in *E. coli* isolated from chicken feces. Ninety-four *E. coli* isolates were obtained from samples collected from different locations in Bangladesh, and the isolates were identified using conventional microbiological tests. Phenotypic disk diffusion tests using 20 antimicrobial agents were performed according to CLSI-EUCAST guidelines, and minimum inhibitory concentrations (MICs) were determined for a subset of samples. *E. coli* isolates showed high resistance to colistin (88.30%), ciprofloxacin (77.66%), trimethoprim/sulfamethoxazole (76.60%), tigecycline (75.53%), and enrofloxacin (71.28%). Additionally, the pathotype *eaeA* gene was confirmed in ten randomly selected *E. coli* isolates using primer-specific polymerase chain reaction (PCR). The presence of *mcr-1* gene was confirmed using PCR and sequencing analysis in six out of ten *E. coli* isolates. Furthermore, sequencing and phylogenetic analyses revealed a similarity between the catalytic domain of *Neisseria meningitidis* lipooligosaccharide phosphoethanolamine transferase A (LptA) and MCR proteins, indicating that the six tested isolates were colistin resistant. Finally, the findings of the present study showed that *E. coli* isolated from chicken harbored *mcr-1* gene, and multidrug and colistin resistance. These findings accentuate the need to implement strict measures to limit the imprudent use of antibiotics, particularly colistin, in agriculture and poultry farms.

## 1. Introduction

Antimicrobial resistance (AMR) is a significant global health problem of increasing concerns. Antimicrobial use, on the other hand, leads to the development and consequent spread of AMR, which is a major global health issue [1]. In recent years, ample evidence has emerged demonstrating a correlation between the use of antibiotics and AMR in animals as a contributing factor to the overall burden of AMR [2]. Because of the intensification of agricultural practices in most developing countries, the extent of animal consumption is projected to rise dramatically in the coming years [3]. Over 60% of all antibiotics produced are currently used in livestock production, including poultry [4].

Poultry is one of the most widely consumed animal proteins, with chicken being the most widely farmed species. In Bangladesh, the poultry industry is a significant sub-sector in terms of economic growth and job creation [5]. The use of antibiotics in poultry and livestock production systems has increased poultry industry productivity [1]. Antimicrobial agents are used in livestock production systems in many countries [3,6,7]. Antibiotics are used for a variety of purposes, including disease prevention, infection treatment, and increased productivity in food animals [3,8]. The widespread use of important antimicrobials in animal production is likely to escalate the emergence of antimicrobial resistance in foodborne pathogens and commensal species. *E. coli* is present in the gut of humans and warm-blooded animals. The majority of *E. coli* strains are nonpathogenic; however, certain strains, on the other hand, may cause serious foodborne illnesses. *E. coli* is a common cause of human urinary tract infections and septicemia. *E. coli* is a major pathogen with a wide range of importance in commercially raised poultry, causing significant economic losses [9]. *E. coli*, on the other hand, is a highly versatile bacterium that has been used as a model microorganism for detecting antimicrobial resistance [10,11]. According to a recent study, the prevalence of multidrug-resistant *E. coli* in food-producing animals increased over the last decade. *E. coli* from chickens was found to be more resistant to numerous antimicrobials than *E. coli* from other food-producing animals [10]. There is growing evidence linking poultry antimicrobial consumption to AMR in humans [12]. Food-producing animals, especially poultry, have been suggested as a potential source for the transmission of extended-spectrum beta-lactamase (ESBL)-producing bacteria to humans, either through direct contact or through consumption of contaminated meat products, resulting in colonization of the intestine and, potentially, causing severe and difficult-to-treat infections with antibiotic-resistant pathogens. Antimicrobial resistance is now recognized as a major threat to human and animal health around the world [13]. Due to the widespread and indiscriminate use of antibiotics in livestock production systems, the rate of multidrug resistance (MDR) in *Enterobacteriaceae* is alarmingly escalating. Owing to the rise in MDR, colistin, a polymyxin antibiotic, has been regarded as a last-resort antibiotic for treating Gram-negative bacterial infections, either alone or in conjunction with other medications. Furthermore, colistin has been used in livestock and poultry for decades all over the world [14]. The emergence of colistin resistance was previously reported [15,16,17]. As a result, it’s not surprising that widespread use of colistin in food-producing animals has aided the spread of colistin resistance [14]. In 2015, China reported the acquisition of the plasmid-mediated colistin *mcr-1* resistance gene [18]. Because of its low fitness cost and ability to be spread to a variety of bacteria, plasmid-borne colistin resistance *mcr-1* poses a significant threat [19,20]. MCR-1 is a phosphoethanolamine (PEA)-lipid A transferase in the bacterial outer membrane that catalyzes the attachment of its moiety to lipid A. In *Neisseria* LptA (EptA), the plasmid-mediated MCR-1 is classified as a subclade adjacent to the chromosome-encoded colistin-resistance mechanism [19,20]. The presence of *mcr* genes in bacterial isolates from animals is higher than in isolates from other sources, raising concerns about the effects of colistin use in food animals and the spread of colistin resistance. Furthermore, the resistance of *Enterobacteriaceae* to multiple antibiotics [21] makes the study of the antibiotic susceptibility profile and antimicrobial resistance gene detection a priority [22]. So far, *mcr-1* positive *Enterobacteriaceae* have been detected in animals, food, humans, and the environment in more than 25 countries across four continents [18,23,24,25,26,27].

In Bangladesh, however, very few studies are available on the detection of *mcr-1* gene in animals or humans [28,29]. To the best of our knowledge, very few systematic investigations on multidrug and colistin *mcr-1* resistance have yet been performed in *E. coli* of chicken origin. Therefore, this study aimed to explore the phenotypic and genotypic features *of E. coli* isolated from chickens using several tests. We examined the phylogenetic relationships between these isolates and compared the results with other published datasets. Additionally, we investigated the phosphatidylethanolamine (PE) substrate molecular docking with MCR-1 and LptA. We focused on the antimicrobial resistance *mcr-1* gene which causes colistin resistance.

## 2. Materials and Methods

### 2.1. Ethics Statement

The sampling protocol and procedures were carried out in compliance with Bangladesh law (Cruelty to Animals Act 1920, Government of the People’s Republic of Bangladesh Act No. I of 1920). The Animal Experimentation Ethics Committee (approval number #AUP2018004) of the Sylhet Agricultural University in Bangladesh reviewed and approved the sampling and experimental procedures.

### 2.2. Study Area and Sampling

**Figure 1:**
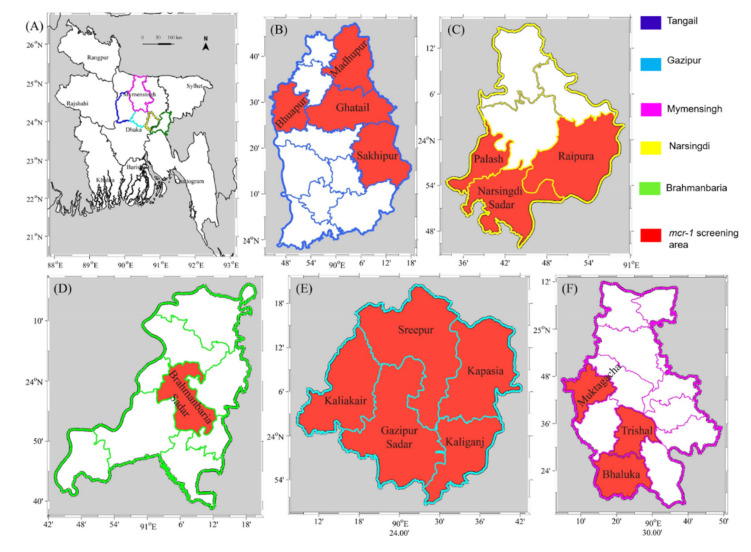
Geographical map of (**A**) Bangladesh showing Mymensingh division and the districts (**B**) Tangail (**C**) Narsingdi (**D**) Brahmanbaria (**E**) Gazipur and (**F**) Mymensingh of the sampling areas in the present study. Sampling areas where the *mcr-1* gene was screened are highlighted in red.

The study sites were selected based on their high density of poultry farms. The selected areas are commonly known as poultry (e.g., broiler and layer) zones, namely the Gazipur, Tangail, Mymensing, Narsingdi, and Brahmanbaria districts of Bangladesh, as shown in Figure 1. Sample collection was conducted between January and June in 2019. Dead and sick chickens (at least 5 chickens per farm) were collected and immediately transported to the laboratory. Based on the full and complete anamnesis, fecal samples were collected following standard procedures and immediately sent for further laboratory analysis.

### 2.3. Isolation and Biochemical Identification of E. coli

Samples were initially screened based on clinical history, symptoms, and postmortem examination. A total of one hundred (100) suspected samples were randomly selected. Samples were pre-enriched in buffered peptone water (Oxoid^®^, Hampshire, UK) at a dilution ratio of 1:10 and were incubated overnight at 37 °C. A loopful of each culture was streaked on Eosin Methylene Blue (EMB) (Hi Media, India) agar and MacConkey’s agar (Oxoid^®^, Hampshire, UK) and plates were incubated at 37 °C for 24 h. Characteristic metallic sheen colonies on EMB agar were selected and subsequently sub-cultured on nutrient agar (NA) (Oxoid^®^, Hampshire, UK), and biochemical tests were performed for further confirmation, including triple sugar iron (TSI), motility indole urease, oxidase and catalase tests, and Gram staining. Single *E. coli* colonies were stored in Brain Heart Infusion broth (Oxoid^®^, Hampshire, UK) in the presence of 15–20% glycerol and kept at −20 °C for further use.

### 2.4. Antimicrobial Susceptibility Testing

The phenotypic antibiogram profiles of *E. coli* isolates were determined using the Kirby–Bauer disk diffusion method, as was previously described [30]. Data were presented by measuring the zones of inhibition for a panel of 20 antimicrobial drugs and scoring them according to CLSI guidelines, as was previously described [31]. MICs for 10 selected isolates were determined using the VITEK 2 compact AST card N280. *E. coli* ATCC 25922 strain was used as the quality control strain. Based on CLSI-EUCAST plus natural resistance guidelines, the susceptibility breakpoints of the tested antimicrobials were interpreted [32,33]. Multidrug-resistant (MDR) isolates were described as those isolates which were found to be resistant to at least three different classes of antibiotics [34].

### 2.5. Extraction of Bacterial DNA

Genomic bacterial DNA was extracted for ten E. coli isolates out of a total of 94 isolates using the boiling-centrifugation process, as was previously described [35].

### 2.6. Molecular Detection of E. coli Pathotype and Colistin mcr-1 Resistance Gene

To amplify pathotype genes in the 10 selected *E. coli* isolates, multiplex PCR reactions were performed. Specific primers targeting pathotype genes (Table 1) with the expected amplicons were used to identify *E. coli* isolates. Nine primers sets were designed using the Primer3 program by retrieving *E. coli* sequences from NCBI, and a multiplex kit was developed by AddBio Inc. (AddBio Inc. Ltd., Daejeon, Korea). DNA amplification was performed according to manufacturer’s instructions (AddBio Inc. Ltd., Daejeon, Korea). The PCR reaction volume of 20 µL consisted of 10 µL of 2× master mix with uracil–DNA glycosylase (UDG), 5 μL of primer mix, and 5 μL of genomic DNA was added. Primers were used at a concentration of 5 pmole per reaction. Positive control consisting of *E. coli* positive plasmid DNA (5 µL) and negative control consisting of sterile molecular grade water were used. To optimize the PCR reaction, an internal control (IC) was used as lambda DNA amplification (1000 bp). The PCR was carried out on a DLAB TC100-G machine (DLAB Scientific Co., Ltd., Beijing, China) using the following reaction conditions: 3 min at 50 °C for UDG reaction, at 95 °C for initial denaturation for 10 min, 30 s at 95 °C for 35 cycles denaturation, 45 s at 68 °C for primer annealing and extension, and 5 min at 72 °C for final elongation. From multiplex PCR reactions, only *eaeA* gene was detected in target samples. Ten indicative samples of *eaeA* gene fragments were sequenced by SolGent (Daejeon, Korea) and confirmed by BLAST (Supplementary File S1).

The presence of the colistin *mcr-1* resistance gene in ten *E. coli* isolates was detected using a PCR assay. Using the Primer3 program, two targeting primer (Table 2) sets were designed (NeoProbe Daejeon, Korea) to cover the coding sequence of 1626 bp full-length gene *mcr-1* with 1197 bp and 799 bp amplicon sizes, respectively. BioEdit software (version 7.2) was used to align all available *mcr-1* gene sequences obtained from NCBI. A volume of 20 µL PCR reaction volume consisted of 10 µL of 2× master mix with uracil–DNA glycosylase (UDG), 5 μL of primer mix, and 5 μL of genomic DNA. Forward and reverse primers were used at 5 pmole each per reaction. The PCR was carried out using a DLAB TC100-G machine (DLAB Scientific Co., Ltd., Beijing, China), with the following reaction conditions: initial denaturation takes 10 min at 95 °C, followed by 30 s at 95 °C for 40 cycles of denaturation, 45 s at 68 °C for primer annealing and extension, and a final elongation stage of 5 min at 72 °C. The amplified PCR products were separated and visualized on a 1.5% agarose gel electrophoresis, and images were viewed using UV transillumination in a gel documentation (Bio-Rad Laboratories Inc., Hercules, CA, USA).

The PCR products were purified (Addbio Inc., Daejeon, Korea) and were subjected to Sanger sequencing (SolGent, Daejeon, Korea). The sequences of each sample were assembled and annotated in GenBank.

### 2.7. Sequences Analysis

Different methods were used to determine the sequence similarity, structure of gene *mcr-1*, and phylogenetic relationship. The MCR-1 and MCR-1-like proteins’ homologous sequences were extracted from NCBI through *BLASTp* search (https://blast.ncbi.nlm.nih.gov/Blast.cgi, accessed on 16 March 2021), with six SAUVM MCR-1 proteins rendered from the sequencing data of *mcr-1* genes of *E. coli.* Aligned sequences of MCR-1 from ClustalW [36] were used to construct a phylogenetic tree through the Maximum Likelihood Method of MEGA X [37]. To confirm the results, 500 bootstrap repetitions were used.

### 2.8. Structural Analysis and Validation

MCR-1 proteins’ transmembrane helices were predicted using the TMHMM server (http://www.cbs.dtu.dk/services/TMHMM/ accessed on 16 March 2021) and standard parameters, as reported recently [17]. When the loop was on the inside or outside, the topology was given as the location of the transmembrane helices differentiated by I and ‘o’ [38]. To determine 3D (three-dimensional) modeling of SAUVM MCR-1, I-TASSER was used. On the basis of pair-wise structural similarity, I-TASSER simulations generate a broad assembly of structural conformations. C-score [39] is a quantitative indicator of each model’s trust. ModRefiner [40], accompanied by the FG-MD refinement server [41], is used to improve the accuracy of the expected structures. Finally, Verified 3D, ERRAT, and RAMPAGE (Ramachandran Plot Assessment server) were used to validate the refined structures [42].

### 2.9. Molecular Docking

To investigate the molecular docking of the phosphatidylethanolamine (PE) substrate, the chemical structure of PE (ZINC identification number (ID): ZINC32837869) was retrieved from the ZINC database [43]. The RCSB Protein Data Bank (PDB) server was used to extract the 3D structure of LptA (formerly termed EptA; PDB ID: 5FGN; Organism: *Neisseria meningitidis*), which is the best template of SAUVM MCR-1 [44]. To perform molecular docking to investigate binding interactions of the PE in the MCR-1 LptA, the Autodock Vina algorithm in PyRx software was used [45]. Using OpenBabel (version 2.3.1), the output PDBQT files were converted into PDB format. To optimize and visualize the protein structures and ligand-binding interaction patterns, the PyMol and Discovery Studio tools were used [46].

## 3. Results

### 3.1. Identification of E. coli Isolates

Conventional microbiological and biochemical methods were used to isolate and identify *E. coli* isolates. A total of 100 fecal samples obtained from dead and sick chickens were collected from different poultry zones. Ninety-four samples yielded isolates identified as *E. coli.* Morphologically, isolates appeared as dry, donut-shaped, dark pink color colonies on MacConkey agar. Further, using selective media, *E. coli* produced greenish metallic sheen colonies on EMB agar. Gram stain smears from suspected colonies showed Gram-negative, rod-shaped bacteria. The bacterial isolates were also positive by motility, catalase, and Kovac’s indole test. The biochemical reactions on TSI agar slants were typical of *E. coli* (displayed both butt and slant in yellow without H_2_S production).

### 3.2. Antimicrobial Susceptibility Testing

The disk diffusion method was used to determine antimicrobial susceptibility of all ninety-four isolates of *E. coli* (*n* = 94) (Table 3). According to the CLSI breakpoints, a significant percentage of resistance to the tested antimicrobials was observed [32]. The resistance rates of colistin (88.30%), ciprofloxacin (77.66%), trimethoprim/sulfamethoxazole (76.60%), tigecycline (75.53%), and enrofloxacin (71.28%) were detected. The tested *E. coli* isolates were found to be susceptible to some antibiotics, with susceptibility rates for ampicillin (84.04%), piperacillin/tazobactam (76.60%), and gentamicin (73.17%).

### 3.3. Detection of Multidrug Resistance E. coli Isolates Carrying the Colistin Resitance mcr-1 Gene

Molecular characterization revealed that all 10 randomly selected isolates harbored *eaeA* (*E. coli* attaching and effacing) virulence gene, which is accountable for enteropathogenic *E. coli* infections. As a confirmatory detection tool, we used a multiplex PCR, and the results showed that the *eaeA* gene was found in *E. coli* isolates, with the predicted amplicon size of 291 bp.

In addition, all ten *eaeA*-positive *E. coli* isolates were examined for the existence of the *mcr-1* gene using PCR with specific primers.

Six *E. coli* isolates were found to carry colistin resistance *mcr-1* gene. The *mcr-1* genes obtained from chicken *E. coli* strains were submitted to the GenBank database and have accession numbers MN879255, MN879256, MN879257, MN879258, MN879259, and MN879260 for SAUVM E1, SAUVM E2, SAUVM E3, SAUVM E5, SAUVM E6, and SAUVM E7, respectively.

**Figure 2:**
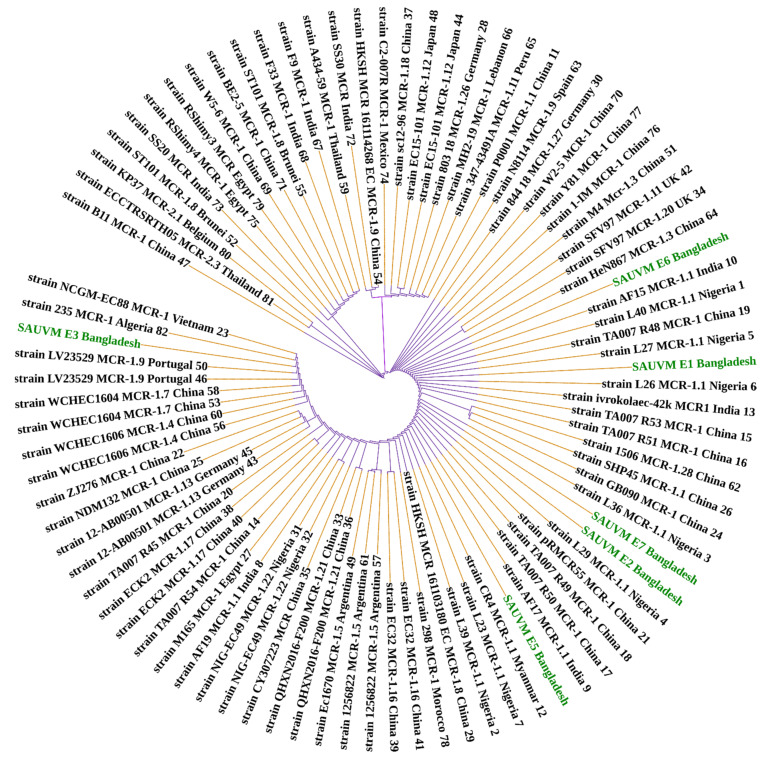
Phylogeny of 91 MCR-1 proteins of *E. coli* origin retrieved from NCBI database including the six sequenced SAUVM MCR-1 genes from this study. Phylogenetic study of MCR-1 and MCR-1-like proteins reveals ancestral origins and diversification. Using amino acid sequences from six SAUVM MCR-1 proteins, the BLAST search tool (https://blast.ncbi.nlm.nih.gov/Blast.cgi accessed on 16 March 2021) was used to retrieve homologous sequences of MCR-1 and MCR-1-like proteins from the NCBI database. MCR-1 and MCR-1-like proteins of *Salmonella*, *E. coli*, strains containing LptA (formerly known as EptA), and other sequences were categorized. Using aligned MCR-1 sequences from CLUSTALW, the maximum likelihood method of MEGA X was used to create a phylogenetic tree.

In the *mcr-1*-positive isolates, Sanger sequencing revealed 100% sequence identity between isolate SAUVM E1 and SAUVM E5, and SAUVM E2 and SAUVM E7 sequences accessed in the NCBI database. Moreover, *mcr-1*-positive isolate SAUVM E3 (mcr-1.24 allele) and SAUVM E6 (mcr-1.25 allele) contained a new allele of the MCR-1 family, compared to others retrieved from the NCBI database ( Figure 2).

Moreover, MIC testing was conducted to detect multidrug resistance in the *mcr-1*-positive *E. coli*. In MIC testing, multidrug resistance (MDR) patterns were observed in all six *mcr-1*-positive *E. coli*, as they were resistant to at least three (3) antimicrobial classes (colistin, ciprofloxacin, and trimethoprim/sulfamethoxazole) (Table 4). Antibiotic resistance was found in eight (*n* = 2 isolates), seven (*n* = 2 isolates), five (*n* = 1 isolate), and four (*n* = 1 isolate) of the tested isolates, as shown in Table 4.

### 3.4. Phylogeny and Structural Analysis

**Figure 3:**
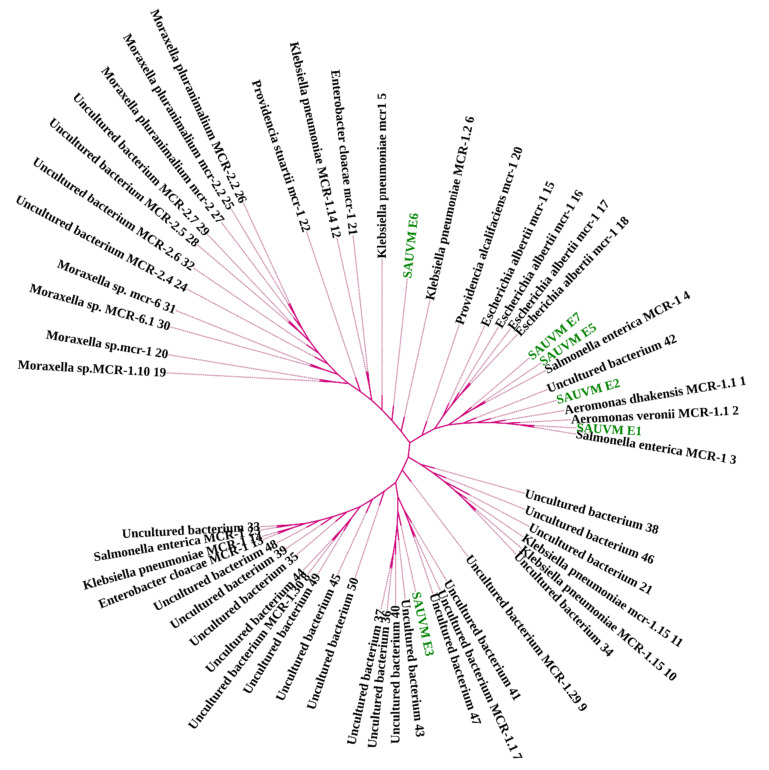
Phylogeny of 52 MCR-1 proteins retrieved from NCBI database, except *E. coli,* including sequenced SAUVM MCR-1. Phylogenetic study of MCR-1 and MCR-1-like proteins reveals ancestral origins and diversification. Using amino acid sequences from six SAUVM MCR-1 proteins, the BLAST search tool (https://blast.ncbi.nlm.nih.gov/Blast.cgi accessed on 16 March 2021) was used to retrieve homologous sequences of MCR-1 and MCR-1-like proteins from the NCBI database. MCR-1 and MCR-1-like proteins of *E. coli*, *Salmonella*, and strains containing LptA (formerly known as EptA) and others were among the sequences categorized. Using aligned MCR-1 sequences from CLUSTALW, the maximum likelihood method of MEGA X was used to create a phylogenetic tree.

Different computational analyses were conducted to determine the phylogeny, to sequence similarities, and to get structural visions of *mcr-1* gene and the particular translated MCR-1 proteins. A total of 91 MCR-1 proteins originated from *E. coli* (Supplementary File S2), and 52 proteins of MCR-1 gene (Supplementary File S3) obtained from bacteria other than *E. coli* were categorized for further analysis. In the case of protein acquisition from the NCBI database, above 30% of query coverage was set as the screening point. These two sets of proteins (Supplementary Files S2 and S3), including SAUVM MCR-1, were used for phylogenetic analysis. The phylogenetic analysis is shown in Figure 2 and Figure 3. All of the sequenced SAUVM MCR-1 clearly showed its genomic confirmation as *mcr-1* genes by highly aligning with *mcr-1* genes of *E. coli* as well as other origins. In addition, the phylogenetic tree showed that all of the SAUVM MCR-1 were mostly of Asian origin and that they were closely related.

### 3.5. Transmembrane Topology Analysis, Structural Modelling, Refinement, and Validation

**Figure 4:**
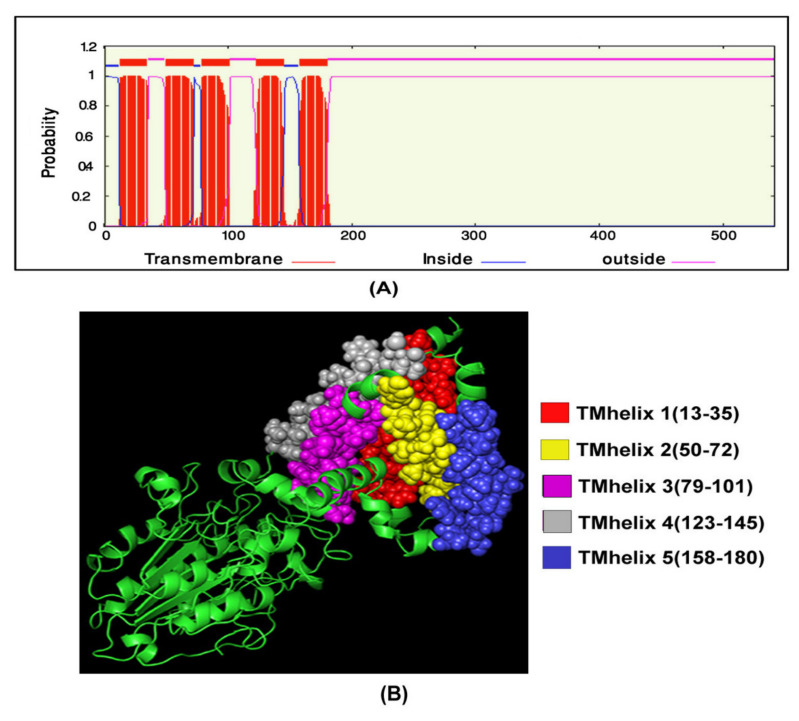
SAUVM MCR-1 protein transmembrane topology prediction. The TMHMM server (http://www.cbs.dtu.dk/services/TMHMM/ accessed on 16 March 2021) was used to predict the (**A**) transmembrane helices of MCR-1 proteins. (**B**) The topology was defined as the relative positions of the transmembrane helices, denoted by the letters I and ‘o’ when the loop is on the inside or outside, respectively.

**Figure 5:**
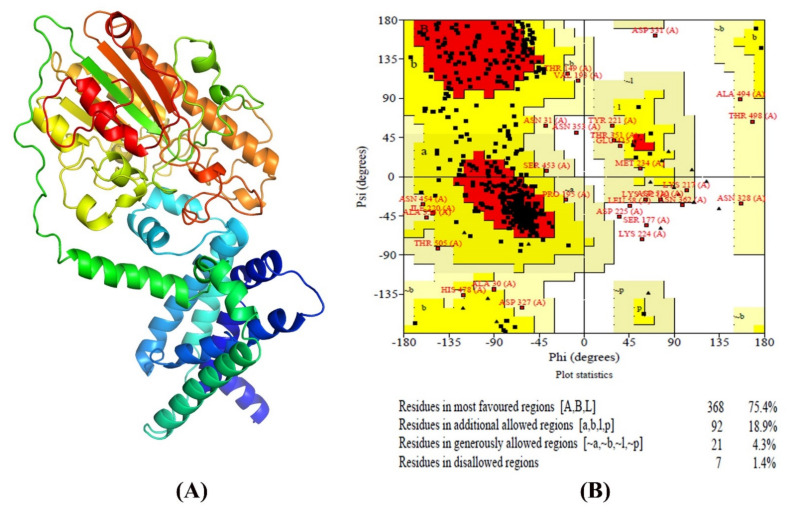
Validation of SAUVM MCR-1 protein modeled structures. (**A**) I-TASSER was used for three-dimensional (3D) modelling of SAUVM MCR-1 proteins, which functions by identifying structure templates from the PDB library. The C-score is a quantitative indicator of each model. SAUVM E1 model was chosen at random from these MCR-1 protein models and (**B**) analyzed, and structures were validated using Ramachandran Plot Assessment server (PROCHECK).

For transmembrane helices prediction in *mcr-1* genes of *E. coli* isolates, as it is an essential analysis of proteins, the TMHMM server was used. TMhelix1 (13–35), TMhelix 2 (50–72), TMhelix 3 (79–101), TMhelix 4 (123–145), and TMhelix 5 (158–180) were the five transmembrane domains in the SAUVM MCR-1 proteins, which spanned the inner membrane region (Figure 4). The I-TASSER server was used to model SAUVM MCR-1 proteins, with the structural template *N. meningitidis* EptA (PDB ID: 5FGN). When compared to EptA, SAUVM MCR-1 proteins displayed 35.4% (35.6%) identity, and their modelled structure had a coverage score of 96%. Refinement was done to enrich the quality of the structure. Refinement Ramachandran plot analysis revealed that 75.4% of residues were in the most preferred region, 18.9% residues in the additional permitted region, and 4.3% residues were in the generously allowed region (Figure 5). In addition, ERRAT showed 94.4% quality factor (Supplementary Figure S1A,B). Finally, Verify3D showed that 94.74% of the residues had averaged 3D-1D score >= 0.2 (Supplementary Figure S2).

### 3.6. Molecular Docking

**Figure 6:**
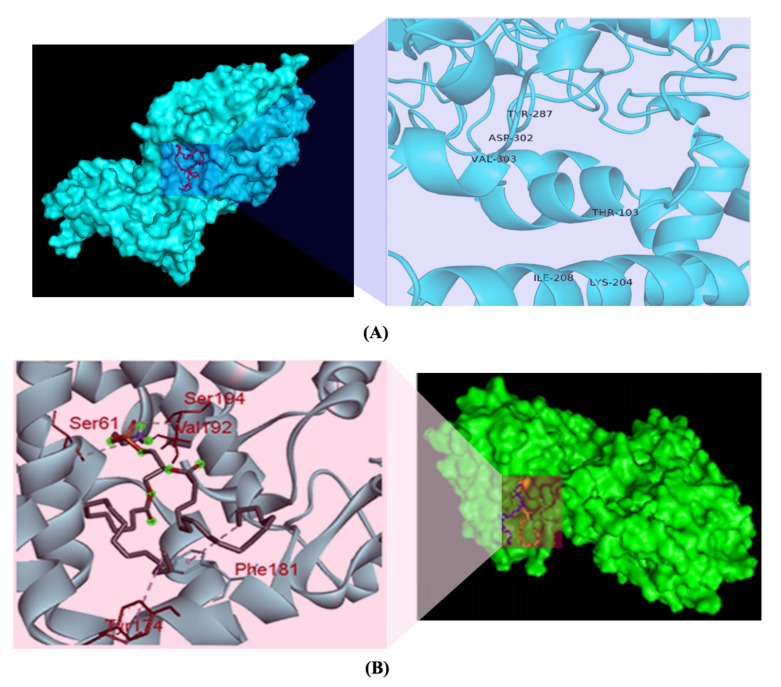
PE substrate ligand-binding interaction pattern with colistin resistance MCR-1 and LptA. The PE substrate with MCR-1 protein as a modelled cartoon structure. PyMol software was used to build the cartoon framework. The substrates bind in the groove of MCR-1 and LptA mainly stretching from 175 to 195, in which Phe, Tyr, and Ser residues were abundant in the substrate-binding region for PE interaction in both (**A**) and (**B**). (**B**) Ligand-binding interaction showed that PE-binding sites were distributed differently in MCR-1 and LptA proteins (SAUVM MCR-1: Thr-103, Lys-204, Ile-208, Tyr-287, Asp-302, Val-303; LptA: Ser 61, Tyr 174, Phe 181, Val 192, Ser 194).

The ligand-binding interaction pattern of PE substrate was identified using various tools. The grid box was set to 85.545° × 80.742° × 83.745° (x, y, and z) with 1 A° spacing between the grid points, and all other parameters were left at their normal settings. Although molecular docking of PE substrate with SAUVM MCR-1 and LptA generated five docking binding confirmations, the binding pattern with the lowest energy was selected (PE & MCR-1: −4.9 kcal/mol and PE & LptA: −3.6 kcal/mol). It was demonstrated that Thr-103, Lys-204, Ile-208, Tyr-287, Asp-302, and Val-303 were the key interactive molecules in the PE-binding cavity of SAUVM MCR-1, whereas Ser 61, Tyr 174, Phe 181, Val 192, and Ser 194 were the vital interactive molecules for LptA (Figure 6).

## 4. Discussion

*E. coli* is transmitted to humans from animals primarily through contaminated foods [47]. *E. coli* of zoonotic origin may directly cause serious food poisoning or gastrointestinal infections [12,13], or be part of the colonizing human flora and later cause urinary tract infections or septicemia. A wide range of foods of animal origin, including poultry, have been linked to such diseases, and this is of large concern. The severity of disease could be reduced by antimicrobial therapy in humans [48] and poultry [49,50]. Nevertheless, the emergence of antimicrobial-resistant *Enterobacteriace* has become a public health problem in several parts of the world [13,51,52]. Particularly, antimicrobial agents commonly prescribed to treat bacterial infections show a reduced susceptibility [53,54] leading to treatment failure [48,55]. Antibiotic misuse has been identified as a major contributor to the rise of MDR bacteria, both in human medicine as well as in the veterinary practices [56]. Therefore, antimicrobial resistance and its association to animals is of major concern and is challenging governments and health authorities. To date, Bangladesh lacks baseline data on the incidence of resistant *E. coli* and their resistance genes identified from different sources (e.g., poultry) in order to establish successful antimicrobial resistance and public health risk management strategies.

In the present study, samples were collected from broiler and layer farms from clinically sick chickens. Samples were tested using morphological, biochemical, and molecular methods to detect antimicrobial-resistant *E. coli* isolates and detect *mcr-1* colistin resistance genes in a subset of them. The isolation rate of *E. coli* strains in sick chickens was found to be 94% from suspected samples. A recent study found that Shiga-toxin-producing *E. coli* isolates may pose a health risk to humans and could play a role in the colonization and transmission of life-threatening *E. coli* strains [57]. Molecular methods allow for the rapid detection of *E. coli* isolates and their resistance genes in poultry. A multiplex PCR method was used to detect *E. coli* isolates from chicken followed by amplification, nucleotide sequencing, and phylogenetic analysis. The pathogenicity of *E. coli* is related to a number of factors, including the *eaeA* virulence gene, which encodes intimin, a pathogenic *E. coli*-binding protein [58]. In the current study, the detected virulence *eaeA* gene (10/10 of tested) in the identified *E. coli* designates the disease-causing characteristics [58,59]. Moreover, apparently healthy animals, especially commercial chickens, and their surroundings carry *Enterobacteriace* in Bangladesh [60,61].

Resistance of *E. coli* against antimicrobial agents is an emerging threat in both developing and developed countries [62]. It was therefore important to investigate the resistance patterns of the isolates to understand the resistance threat they could pose. In tested *E. coli* isolates, we detected high levels of resistance to colistin, ciprofloxacin, and trimethoprim/sulfamethoxazole. These high resistance rates are most likely due to the widespread use or misuse of these antibiotics in veterinary medicine, which has resulted in the accumulation of antimicrobial-resistant bacteria that can infect humans and contribute to acquired infectious diseases [18,62,63]. In addition, resistance to trimethoprim/sulfamethoxazole and enrofloxacin in 75.53%, and 71.28%, respectively, of chicken isolates deserves our attention [50,64]. A recent study on antimicrobial resistance profiling reported tetracycline resistance (95.0%) in *Enterobacteriace* isolated from chickens [65]. Possibly, the resistance rates for these antibiotics are lower due to their lower use in veterinary medicine. Moreover, this finding was in line with previous reports [63,66]. The quinolone-class antibiotic ciprofloxacin showed high resistance to screened *E. coli* isolates previously [67]. Currently, ciprofloxacin is extensively used in the poultry industries; it has been introduced during the last decade in Bangladesh for this use. However, a previous study reported that *E. coli* isolates were sensitive to ciprofloxacin [60,68]. Additionally, antibiotics from the same class may cause cross-resistance, which contributes to high resistance rates. Antibiotics such as tetracyclines and trimethoprim/sulfamethoxazole are widely used for poultry care and used as growth promoters in feed. The majority of the time, poultry farmers use these medications without a veterinarian’s prescription [69]. A recent study reported that 94% of MDR *E. coli* in poultry environments in Bangladesh had colistin MIC of ≥4 µg/mL and 13.5% of them were *mcr-1*-positive [70]. Surprisingly, the present study found that all of the tested *mcr-1*-positive *E. coli* isolates were MDR. MDR *E. coli* isolates from our country and the rest of the world have been found to have similar reports [53,71,72]. MDR strains appear to be common, most likely as a result of the indiscriminate use of antimicrobial agents. Given the risk of resistant isolates being transmitted directly from animals to humans, this is a serious problem for both veterinary medicine and human health [2].

Colistin has been extensively used in the agricultural production system in China [18,73] since the early 1980s, causing the initial emergence with global spread of *mcr-1* in recent years [14,18,28,72,74]. In this analysis, the presence of the colistin resistance *mcr-1* gene (6/10) in *E. coli* strains isolated from chicken feces was identified using a multiplex PCR-based detection system. This high rate of *mcr-1* in chicken *E. coli* isolates was unexpected, and it indicates that *mcr-1* is already prevalent in Bangladeshi food animals. These results support a recent study where 61.7% of *E. coli* isolates from poultry guts showed colistin resistance and 36.4% were positive for *mcr* genes [75]. While we were unable to obtain actual field data on uses of antibiotics, the existence of the *mcr-1* gene suggests that colistin was used frequently in the poultry industry in this area, possibly in combination with other antimicrobials. A recent study has stated that 28% of chicken samples harbored *mcr-1* in China, and it has been linked to both humans and animals [18]. A study in the Netherlands found an unexpectedly high incidence of *mcr-1* (24.8%) in chicken samples [76]. Other studies reported the existence of *mcr-1* in *E. coli* isolates recovered from poultry and livestock. [59,64,76,77,78]. The higher presence of the *mcr-1* gene in chicken *Enterobacteriace* is concerning, especially in Bangladesh, where antibiotic usage in humans and animals is poorly regulated. Hence, the recent emergence of colistin resistance has prompted international guidelines for colistin usage restrictions in agricultural production systems [72,79].

While the detailed molecular mechanisms of the resistant *E. coli* isolates tested in the current study are lacking, we used integrative approaches ranging from nucleotide sequence analysis, bioinformatics, and structural modeling to bacterial genetics to resolve them. The revealing of new mcr-1-harbouring *E. coli* isolates enhances our understanding of the newly emerging field of colistin resistance *mcr-1* genes. It increases our knowledge of the homology, structure, and validation of mcr-1 genes present in *E. coli* isolates. It was reported that the mcr-1 colistin resistance gene has previously been found in a multidrug-resistant plasmid [62,80]. We also found that the identified colistin resistance *mcr-1* genes were closely similar to a plasmid isolated from China [18].

These facts exemplify that colistin resistance in MDR bacteria can develop over time, a finding that should be taken seriously. It is therefore of great interest to determine the multiple sequence alignments for *mcr-1* genes in *E. coli* isolated from poultry. In order to elude hits from very closely associated species, retrieved sequences of *E. coli* species were excluded from the phylogeny study, and those were only aligned with SAUVM MCR-1 proteins. From the multiple sequence alignments of SAUVM MCR-1 proteins, it has been found that they possess putative conserved sites and belong to the Mobilized Colistin Resistance (MCR) protein family. The study demonstrated that the newly identified *mcr-1* genes had five transmembrane helices: TMhelix1 (13–35), TMhelix 2 (50–72), TMhelix 3 (79–101), TMhelix 4 (123–145), and TMhelix 5 (158–180), which spanned the inner membrane region [74,81]. The phylogenetic analysis indicated that the sequenced *mcr-1* genes of *E. coli* are homologous to previously reported *mcr-1* genes from *E. coli* and other origins. The phylogeny of the *mcr-1* identified in our study is identical to that of the *mcr-1* previously identified from *Escherichia coli* and *Salmonella* spp. [81,82,83]. Moreover, SAUVM MCR-1 showed an evolutionary relation with the Asian origin of *mcr-1,* and it had its closest ancestral relationship with *Salmonella enterica* strains. The phylogenetic tree demonstrates a close relation among some studied *mcr-1* strains, such as SAUVM E1, SAUVM E2, and SAUVM E7, and an evolutionary relationship to other *mcr-1* genes like strain L26 MCR1.1, L26 MCR1.1, strain L29, and strain L36. From a similar study, the antimicrobial susceptibility phenotype of *E. coli* LV23529 showed resistance to colistin, and the phylogenetic tree of this study also showed that SAUVM E3 is closely linked to it [82]. Since the discovery of *mcr-1*, other variations of the gene have been discovered, including mcr-1.2, mcr-2, mcr-1.3, mcr-1.4, mcr-1.5, mcr-1.6, mcr-1.7, and mcr-1.8. The phylogenetic tree revealed that SAUVM MCR-1 is related to many MCR-1 variants. SAUVM E3, for example, is closely related to the mcr-1.9 strain, and its spread could result in widespread resistance to colistin [84]. To increase the structural insight, 3D homology modelling of six SAUVM MCR-1 proteins was constructed, and *Neisseria meningitides* LptA was used as a structural template. Further, molecular interactions between Phosphatidylethanolamine (PE) substrate with MCR-1 and LptA was investigated. The ligand-binding interaction pattern of PE substrate with SAUVM MCR-1 and LptA revealed that PE-binding sites were localized differently in SAUVM MCR-1 and LptA. In addition, Phe 181, Tyr 174, Val 192, Ser 61, and Ser 194 were the prime interactive molecules in the PE-binding cavity of LptA while Thr-103, Lys-204, Ile-208, Tyr-287, Asp-302, and Val-303 were for SAUVM MCR-1.

The use of the disk diffusion method for assessing susceptibility to colistin is a weakness of the work. Due to limited resources, out of 94 samples, we randomly selected 10 isolates only for *eaeA* gene and *mcr-1* gene detection in *E. coli isolates* from poultry samples. This is another limitation of the study, but the 10 characterized isolates still give important information regarding *mcr-1,* as well as other molecular characteristics.

## 5. Conclusions

The present study demonstrates the presence of *mcr-1* gene mediated colistin resistance in multidrug-resistant *E. coli* isolated from chickens in Bangladesh. Our data, although limited, represents a significant snapshot of colistin resistance *mcr-1* genes and highlights the increasing issue of transferable colistin resistance in Bangladesh. The results also demonstrated in silico functional analysis and the phylogenetic relationships of colistin-resistance MCR-1 proteins. In addition, phylogenetic analysis showed an evolutionary linkage between the catalytic domain of the phosphatidylethanolamine substrate LptA and MCR proteins, indicating that they are colistin resistant. The present study highlights the urgent need to incorporate risk management strategies and to investigate the imprudent use of colistin and other antimicrobial agents in food-chain animals in the country.

## Figures and Tables

**Table 1:** Sequences of primers used for the detection of virulence genes in *Escherichia coli* isolates in the present study.

Target Gene	Pathotype	Primer Name	Primer Sequence (5′-3′)	Amplicon Size (bp)
Lambda	Internal Control	LF	CAGATCTCCAGCACGGAACTATTGAGTACGAACG	1000
		LR	GCATAAAATGCGGGGATTCACTGGCTGC	
ipaH	EIEC	Ip-F	GATGCCGTGACAGCATGGTTCC	718
		Ip-R	CAGCCGGTCAGCCACCCTCTG	
Vt1	EHEC	V1-F	GTCATTCGCCCTGCAATAGGTACTCC	599
		V1-R	AGTCTTGTCCATGATAGTCAGGCAGGAC	
vt2	EHEC	V2-F	CGGACAGAGATATCGACCCCTCTTGAAC	481
		V2-R	CCTGACGAAATTCTCTCTGTATCTGCCTGAAG	
bfpA	EPEC	Bf-F	CAGAATGCTATTTCAGAAGTAATGAGCGCAAC	400
		Bf-R	CAGTTGCCGCTTCAGCAGGAGTAATAG	
aggR	EAEC	Ag-F	GGTCAAAAGGAATAATTGTAGCTGATGCTGACGAT	341
		Ag-R	GCTGCTTTGCTCATTCTTGATTGCATAAGGATCTGG	
eaeA	EPEC	Ea-F	CCAGGCTTCGTCACAGTTGCAGGC	291
		Ea-R	CGCCACCAATACCTAAACGGGTATTATCACC	
stp	ETEC	Sp-F	CCGTGAAACAACATGACGGGAGGTAACATGAAAAAGC	234
		Sp-R	GCACAGGCAGGATTACAACAAAGTTCACAGCAG	
sth	ETEC	Sh-F	TTCACCTTTCGCTCAGGATGCTAAACCAG	171
		Sh-R	AGCACCCGGTACAAGCAGGATTACAACAC	
lt	ETEC	Lt-F	GCAGGTTTCCCACCGGATCACCAAGC	127
		Lt-R	CAGATTCTGGGTCTCCTCATTACAAGTATCACCTG	

**Table 2:** Sequences of primers used for the detection of *mcr-1* gene in *E. coli* isolates.

Target Gene	Primer Name	Primer Sequence	Size (bp)
MCR1	MCR1-P1F MCR1-P1R	F: CAGTATGGGATTGCGCAATGATT R: TTATCCATCACGCCTTTTGAGTC	1197
MCR1	MCR1-P2F MCR1-P2R	F: TGTCGATACCGCCAAATACCAAG R: GGAGTGTGCGGTGGGTTTG	799

**Table 3:** Antimicrobial susceptibility profiles of *E coli* isolates (*n* = 94) in the present study.

Antimicrobial Agents	Susceptible (S)		Intermediate (I)		Resistance (R)		I + R
	Number of Isolates	%	Number of Isolates	%	Number of Isolates	%	
**Penicillins**							
Ampicillin (AMP, 10 μg)	79	84.04	6	6.38	9	9.57	15.96
Amoxycillin/clavulanate (AMC, 20/10 µg)	55	58.51	27	28.72	12	12.77	41.49
Piperacillin/tazobactam (PTZ, 100/10 μg)	72	76.60	14	14.89	8	8.51	23.40
**Aminoglycosides**							
Amikacin (AMK, 30 μg)	58	61.70	19	20.21	17	18.09	38.30
Gentamicin (GEN, 10 μg)	60	73.17	20	26.83	14	17.07	43.90
**Cephalosporins**							
Cefuroxime (CFX, 30 μg)	53	56.38	23	24.47	18	19.15	43.62
Cefuroxime axetil (CFA, 30 μg)	59	62.77	23	24.47	12	12.77	37.23
Ceftriaxone (CTR, 30 μg)	62	75.61	11	11.34	24	24.74	36.08
Cefoperazone/sulbactam (CFS, 75/30 μg)	53	56.38	30	31.91	11	11.70	43.62
Cefepime (CFP, 30 μg)	51	54.26	17	18.09	26	27.66	45.74
**Carbapenems**							
Ertapenem (ETP, 10 μg)	62	65.96	23	24.47	9	9.57	34.04
Imipenem (IMP, 10 μg)	62	65.96	20	21.28	12	12.77	34.04
Meropenem (MPM, 10 μg)	63	67.02	22	23.40	9	9.57	32.98
**Tetracyclines**							
Tigecycline (TIG, 15 μg)	9	9.57	14	14.89	71	75.53	90.43
**Quinolones & Fluoroquinolones**							
Ciprofloxacin (CIP, 5 μg)	14	14.89	7	7.45	73	77.66	85.11
Enrofloxacin (ENR, 10 μg)	23	24.47	4	4.26	67	71.28	75.53
Nalidixic acid (NAL, 30 μg)	15	15.96	10	10.64	69	73.40	84.04
**Nitrofurans**							
Nitrofurantoin (NIT, 300 µg)	58	61.70	17	18.09	19	20.21	38.30
**Polymyxins**							
Colistin (COL, 10 μg)	3	3.19	8	8.51	83	88.30	96.81
**Folate Pathway Inhibitors**							
Trimethoprim/Sulfamethoxazole (SXT, 1.25/23.75 µg)	9	9.57	13	13.83	72	76.60	90.43

% = percentage (number of isolates/total number of isolates tested). CLSI Zone Diameter Interpretive Criteria (mm).

**Table 4:** Multidrug resistance patterns among *mcr-1*-positive *E. coli* isolates in the present study.

*E. coli*	COL	SXT	CIP	ENR	TIG	AMC	CTR	GEN	AMK	IMP	MPM	NIT	Antibiotic
Isolate Number													Resistance (*n*=)
SAUVM E1	R	R	R	R	R	S	S	R	R	S	R	S	8
SAUVM E2	R	R	R	S	S	R	R	R	R	S	S	S	7
SAUVM E3	R	R	R	R	R	S	S	S	S	S	S	S	5
SAUVM E5	R	R	R	R	R	S	S	S	S	R	R	R	8
SAUVM E6	R	S	R	R	R	S	S	S	S	S	S	S	4
SAUVM E7	R	R	R	R	R	S	S	S	S	R	R	S	7

S = susceptible; R = resistance; COL = colistin; SXT = trimethoprim/sulfamethoxazole; CIP = ciprofloxacin; ENR = Enrofloxacin; TIG = tigecycline; AMC = amoxicillin/clavulanic acid; CTR = ceftriaxone; GEN = gentamicin; AMK = amikacin; IMP = imipenem; MPM = meropenem; NIT = nitrofurantoin; resistant if, colistin: ≥4; trimethoprim/sulfamethoxazole: ≥80; amoxicillin/clavulanic acid: ≥32; ceftriaxone: ≥4; ciprofloxacin: ≥1; gentamicin: ≥0.5; amikacin: ≥0.5; imipenem: ≥4; meropenem: ≥4; nitrofurantoin: ≥128. VITEK 2 systems version: 08.01; control: *E. coli* (ATCC 25922). MIC interpretation guideline/parameter set: copy of global CLSI based + natural resistance. AES parameter set: copy of global CLSI based + natural resistance.

## Data Availability

Data generated and analyzed in the present study were included in the manuscript and in the supplementary files. The sequence data of the six *mcr-1* genes obtained from *E. coli* strains in the present study were deposited in GenBank (https://www.ncbi.nlm.nih.gov/genbank/) (accessed on 15 March 2021) under accession numbers MN879255, MN879256, MN879257, MN879258, MN879259 and MN879260 for SAUVM E1, SAUVM E2, SAUVM E3, SAUVM E5, SAUVM E6, and SAUVM E7, respectively.

## Supplementary Materials

The following are available online at https://www.mdpi.com/article/10.3390/antibiotics11010097/s1. Supplementary Figure S1A,B: Structure validation by ERRAT; Supplementary Figure S2: Structure validation by Verify3D server; Supplementary File S1: Amplicons of 291bp *eaeA* gene sequence of *E. coli* isolates (E1–E10). Supplementary File S2: A total of 91 MCR-1 proteins of *E. coli* origin retrieved from NCBI database. Supplementary File S3: A total of 52 MCR-1 proteins retrieved from NCBI database except *E. coli*.

## References

[1] Roth N., Käsbohrer A., Mayrhofer S., Zitz U., Hofacre C., Domig K.J. (2019). The application of antibiotics in broiler production and the resulting antibiotic resistance in *Escherichia coli*: A global overview. Poult. Sci..

[2] Marshall B.M., Levy S.B. (2011). Food animals and antimicrobials: Impacts on human health. Clin. Microbiol. Rev..

[3] Van Boeckel T.P., Brower C., Gilbert M., Grenfell B.T., Levin S.A., Robinson T.P., Teillant A., Laxminarayan R. (2015). Global trends in antimicrobial use in food animals. Proc. Natl. Acad. Sci. USA.

[4] Van Boeckel T.P., Gandra S., Ashok A., Caudron Q., Grenfell B.T., Levin S.A., Laxminarayan R. (2014). Global antibiotic consumption 2000 to 2010: An analysis of national pharmaceutical sales data. Lancet Infect. Dis..

[5] Hamid M.A., Rahman M.A., Ahmed S., Hossain K.M. (2016). Status of Poultry Industry in Bangladesh and the Role of Private Sector for its Development. Asian J. Poult. Sci..

[6] Boamah V.E., Agyare C., Odoi H., Dalsgaard A. (2016). Antibiotic Practices and Factors Influencing the Use of Antibiotics in Selected Poultry Farms in Ghana. J. Antimicrob. Agents.

[7] Landers T.F., Cohen B., Wittum T.E., Larson E.L. (2012). A review of antibiotic use in food animals: Perspective, policy, and potential. Public Health Rep..

[8] Castanon J.I.R. (2007). History of the use of antibiotic as growth promoters in European poultry feeds. Poult. Sci..

[9] Hammerum A.M., Heuer O.E. (2009). Human Health Hazards from Antimicrobial-Resistant *Escherichia coli* of Animal Origin. Clin. Infect. Dis..

[10] Radimersky T., Frolkova P., Janoszowska D., Dolejska M., Svec P., Roubalova E., Cikova P., Cizek A., Literak I. (2010). Antibiotic resistance in faecal bacteria (*Escherichia coli*, *Enterococcus* spp.) in feral pigeons. J. Appl. Microbiol..

[11] Benameur Q., Tali-Maamar H., Assaous F., Guettou B., Rahal K., Ben-Mahdi M.H. (2019). Detection of multidrug resistant *Escherichia coli* in the ovaries of healthy broiler breeders with emphasis on extended-spectrum β-lactamases producers. Comp. Immunol. Microbiol. Infect. Dis..

[12] Lazarus B., Paterson D.L., Mollinger J.L., Rogers B.A. (2015). Do human extraintestinal *Escherichia coli* infections resistant to expanded-spectrum cephalosporins originate from food-producing animals? A systematic review. Clin. Infect. Dis..

[13] Falgenhauer L., Imirzalioglu C., Oppong K., Akenten C.W., Hogan B., Krumkamp R., Poppert S., Levermann V., Schwengers O., Sarpong N. (2019). Detection and characterization of ESBL-producing *Escherichia coli* from humans and poultry in Ghana. Front. Microbiol..

[14] Kempf I., Jouy E., Chauvin C. (2016). Colistin use and colistin resistance in bacteria from animals. Int. J. Antimicrob. Agents.

[15] Zhang J., Chen L., Wang J., Yassin A.K., Butaye P., Kelly P., Gong J., Guo W., Li J., Li M. (2018). Molecular detection of colistin resistance genes (mcr-1, mcr-2 and mcr-3) in nasal/oropharyngeal and anal/cloacal swabs from pigs and poultry. Sci. Rep..

[16] Yin W., Li H., Shen Y., Liu Z., Wang S., Shen Z., Zhang R., Walsh T.R., Shen J., Wang Y. (2017). Novel plasmid-mediated colistin resistance gene mcr-3 in *Escherichia coli*. mBio.

[17] Uddin M.B., Hossain S.B., Hasan M., Alam M.N., Debnath M., Begum R., Roy S., Harun-Al-rashid A., Chowdhury M.S.R., Rahman M.M. (2021). Multidrug antimicrobial resistance and molecular detection of MCR-1 gene in *Salmonella* species isolated from chicken. Animals.

[18] Liu Y.Y., Wang Y., Walsh T.R., Yi L.X., Zhang R., Spencer J., Doi Y., Tian G., Dong B., Huang X. (2016). Emergence of plasmid-mediated colistin resistance mechanism MCR-1 in animals and human beings in China: A microbiological and molecular biological study. Lancet Infect. Dis..

[19] Zhang H., Hou M., Xu Y., Srinivas S., Huang M., Liu L., Feng Y. (2019). Action and mechanism of the colistin resistance enzyme MCR-4. Commun. Biol..

[20] Gao R., Hu Y., Li Z., Sun J., Wang Q., Lin J., Ye H., Liu F., Srinivas S., Li D. (2016). Dissemination and Mechanism for the MCR-1 Colistin Resistance. PLoS Pathog..

[21] Antunes P., Mourão J., Campos J., Peixe L. (2016). Salmonellosis: The role of poultry meat. Clin. Microbiol. Infect..

[22] Elkenany R., Elsayed M.M., Zakaria A.I., El-Sayed S.A.E.S., Rizk M.A. (2019). Antimicrobial resistance profiles and virulence genotyping of *Salmonella enterica* serovars recovered from broiler chickens and chicken carcasses in Egypt. BMC Vet. Res..

[23] Wang Y., Zhang R., Li J., Wu Z., Yin W., Schwarz S., Tyrrell J.M., Zheng Y., Wang S., Shen Z. (2017). Comprehensive resistome analysis reveals the prevalence of NDM and MCR-1 in Chinese poultry production. Nat. Microbiol..

[24] Gad A.H., Abo-Shama U.H., Harclerode K.K., Fakhr M.K. (2018). Prevalence, serotyping, molecular typing, and antimicrobial resistance of *Salmonella* isolated from conventional and organic retail ground poultry. Front. Microbiol..

[25] Clothier K.A., Kim P., Mete A., Hill A.E. (2018). Frequency, serotype distribution, and antimicrobial susceptibility patterns of *Salmonella* in small poultry flocks in California. J. Vet. Diagn. Investig..

[26] Baron S., Hadjadj L., Rolain J.M., Olaitan A.O. (2016). Molecular mechanisms of polymyxin resistance: Knowns and unknowns. Int. J. Antimicrob. Agents.

[27] Rolain J.M., Olaitan A.O. (2016). Plasmid-mediated colistin resistance: The final blow to colistin?. Int. J. Antimicrob. Agents.

[28] Sobur M.A., Ievy S., Haque Z.F., Nahar A., Zaman S.B., Rahman M.T. (2019). Emergence of colistin-resistant *Escherichia coli* in poultry, house flies, and pond water in Mymensingh, Bangladesh. J. Adv. Vet. Anim. Res..

[29] Islam A., Rahman Z., Monira S., Rahman M.A., Camilli A., George C.M., Ahmed N., Alam M. (2017). Colistin resistant Escherichia coli carrying mcr-1 in urban sludge samples: Dhaka, Bangladesh. Gut Pathog..

[30] Bayer A.W., Kirby W.M.M., Sherris J.C., Turck M. (2018). Antibiotic Susceptibility Testing by a Standardized Single Disk Method. Am. J. Clin. Pathol..

[31] Clinical and Laboratory Standards Institute (2014). Supplement M100: Performance Standards for Antimicrobial Susceptibility Testing.

[32] Clinical and Laboratory Standards Institute (2016). Performance Standards for Antimicrobial Susceptibility Testing Supplement M100S.

[33] Satlin M.J., Weinstein M.P., Patel J., Romney M., Kahlmeter G., Giske C.G., Turnidge J. (2020). Clinical and Laboratory Standards Institute and European Committee on Antimicrobial Susceptibility Testing Position Statements on Polymyxin B and Colistin Clinical Breakpoints. Clin. Infect. Dis..

[34] Sweeney M.T., Lubbers B.V., Schwarz S., Watts J.L. (2018). Applying definitions for multidrug resistance, extensive drug resistance and pandrug resistance to clinically significant livestock and companion animal bacterial pathogens. J. Antimicrob. Chemother..

[35] Dashti A.A., Jadaon M.M., Abdulsamad A.M., Dashti H.M. (2009). Heat treatment of bacteria: A simple method of DNA extraction for molecular techniques. Kuwait Med. J..

[36] Larkin M.A., Blackshields G., Brown N.P., Chenna R., Mcgettigan P.A., McWilliam H., Valentin F., Wallace I.M., Wilm A., Lopez R. (2007). Clustal W and Clustal X version 2.0. Bioinformatics.

[37] Kumar S., Stecher G., Li M., Knyaz C., Tamura K. (2018). MEGA X: Molecular evolutionary genetics analysis across computing platforms. Mol. Biol. Evol..

[38] Krogh A., Larsson B., Von Heijne G., Sonnhammer E.L.L. (2001). Predicting transmembrane protein topology with a hidden Markov model: Application to complete genomes. J. Mol. Biol..

[39] Zhang Y. (2008). I-TASSER server for protein 3D structure prediction. BMC Bioinform..

[40] Xu D., Zhang Y. (2011). Improving the physical realism and structural accuracy of protein models by a two-step atomic-level energy minimization. Biophys. J..

[41] Zhang J., Liang Y., Zhang Y. (2011). Atomic-level protein structure refinement using fragment-guided molecular dynamics conformation sampling. Structure.

[42] Lovell S.C., Davis I.W., Adrendall W.B., de Bakker P.I.W., Word J.M., Prisant M.G., Richardson J.S., Richardson D.C. (2003). Structure validation by Calpha geometry: Phi,psi and Cbeta deviation. Proteins-Struct. Funct. Genet..

[43] Irwin J.J., Shoichet B.K. (2005). ZINC—A free database of commercially available compounds for virtual screening. J. Chem. Inf. Model..

[44] Deshpande N., Addess K.J., Bluhm W.F., Merino-Ott J.C., Townsend-Merino W., Zhang Q., Knezevich C., Xie L., Chen L., Feng Z. (2005). The RCSB Protein Databa Bank: A redesigned query system and relational database based on the mmCIF schema. Nucleic Acids Res..

[45] Dallakyan S., Olson A.J. (2014). Small-molecule library screening by docking with PyRx. Chemical Biology.

[46] Temml V., Kaserer T., Kutil Z., Landa P., Vanek T., Schuster D. (2014). Pharmacophore modeling for COX-1 and-2 inhibitors with LigandScout in comparison to Discovery Studio. Future Med. Chem..

[47] Barlaam A., Parisi A., Spinelli E., Caruso M., Di Taranto P., Normanno G. (2019). Global emergence of colistin-resistant *Escherichia coli* in food chains and associated food safety implications: A review. J. Food Prot..

[48] Bythwood T.N., Soni V., Lyons K., Hurley-Bacon A., Lee M.D., Hofacre C., Sanchez S., Maurer J.J. (2019). Antimicrobial Resistant *Salmonella enterica* Typhimurium Colonizing Chickens: The Impact of Plasmids, Genotype, Bacterial Communities, and Antibiotic Administration on Resistance. Front. Sustain. Food Syst..

[49] World Organisation for Animal Health (2019). Terrestrial Animal Health Code. Prevention, Detection, and Control of Salmonella in Poultry.

[50] Clifford K., Desai D., da Costa C.P., Meyer H., Klohe K., Winkler A., Rahman T., Islam T., Zaman M.H. (2018). Antimicrobial resistance in livestock and poor quality veterinary medicines. Bull. World Health Organ..

[51] Ahmed I., Rabbi M.B., Sultana S. (2019). Antibiotic resistance in Bangladesh: A systematic review. Int. J. Infect. Dis..

[52] Islam K.B.M.S., Shiraj-um-mahmuda S. (2016). Antibiotic Usage Patterns in Selected Broiler Farms of Bangladesh and their Public Health Implications. J. Public Health Dev. Ctries.

[53] Wasyl D., Kern-Zdanowicz I., Domańska-Blicharz K., Zajac M., Hoszowski A. (2015). High-level fluoroquinolone resistant *Salmonella enterica* serovar Kentucky ST198 epidemic clone with IncA/C conjugative plasmid carrying blaCTX-M-25 gene. Vet. Microbiol..

[54] Iwamoto M., Reynolds J., Karp B.E., Tate H., Fedorka-Cray P.J., Plumblee J.R., Hoekstra R.M., Whichard J.M., Mahon B.E. (2017). Ceftriaxone-resistant nontyphoidal salmonella from humans, retail meats, and food animals in the United States, 1996–2013. Foodborne Pathog. Dis..

[55] Tribble D.R. (2017). Resistant pathogens as causes of traveller’s diarrhea globally and impact(s) on treatment failure and recommendations. J. Travel Med..

[56] Chantziaras I., Boyen F., Callens B., Dewulf J. (2014). Correlation between veterinary antimicrobial use and antimicrobial resistance in food-producing animals: A report on seven countries. J. Antimicrob. Chemother..

[57] Havelaar A.H., Kirk M.D., Torgerson P.R., Gibb H.J., Hald T., Lake R.J., Praet N., Bellinger D.C., de Silva N.R., Gargouri N. (2015). World Health Organization Global Estimates and Regional Comparisons of the Burden of Foodborne Disease in 2010. PLoS Med..

[58] Fitzgerald S.F., Beckett A.E., Palarea-Albaladejo J., McAteer S., Shaaban S., Morgan J., Ahmad N.I., Young R., Mabbott N.A., Morrison L. (2019). Shiga toxin sub-type 2a increases the efficiency of *Escherichia coli* O157 transmission between animals and restricts epithelial regeneration in bovine enteroids. PLoS Pathog..

[59] Abd El-Mongy M., Abd-El-Moneam G.M., Moawad A.A., Mohammed A.B.A. (2017). Serotyping and virulence genes detection in *Escherichia coli* isolated from broiler chickens. J. Biol. Sci..

[60] Sary K., Fairbrother J.M., Arsenault J., De Lagarde M., Boulianne M. (2019). Antimicrobial Resistance and Virulence Gene Profiles among *Escherichia coli* Isolates from Retail Chicken Carcasses in Vietnam. Foodborne Pathog. Dis..

[61] Parvej M.S., Rahman M., Uddin M.F., Nazir K.N.H., Jowel M.S., Khan M.F.R., Rahman M.B. (2016). Isolation and Characterization of *Salmonella enterica* serovar Typhimurium Circulating Among Healthy Chickens of Bangladesh. Turk. J. Agric. Food Sci. Technol..

[62] Rahaman M.T., Rahman M., Rahman M.B., Khan M.F.R., Hossen M.L., Parvej M.S., Ahmed S. (2014). Poultry *Salmonella* Specific Bacteriophage Isolation and Characterization. Bangladesh J. Vet. Med..

[63] Kamal Niaz F. (2016). Emerging Issue of *Escherichia coli* Resistance: A Threat to Public Health. Health Sci. J..

[64] Zhang L., Fu Y., Xiong Z., Ma Y., Wei Y., Qu X., Zhang H., Zhang J., Liao M. (2018). Highly prevalent multidrug-resistant *Salmonella* from chicken and pork meat at retail markets in Guangdong, China. Front. Microbiol..

[65] Jahantigh M., Samadi K., Dizaji R.E., Salari S. (2020). Antimicrobial resistance and prevalence of tetracycline resistance genes in *Escherichia coli* isolated from lesions of colibacillosis in broiler chickens in Sistan, Iran. BMC Vet. Res..

[66] Sinwat N., Angkittitrakul S., Chuanchuen R. (2015). Characterization of Antimicrobial Resistance in *Salmonella enterica* Isolated from Pork, Chicken Meat, and Humans in Northeastern Thailand. Foodborne Pathog. Dis..

[67] Pormohammad A., Nasiri M.J., Azimi T. (2019). Prevalence of antibiotic resistance in escherichia coli strains simultaneously isolated from humans, animals, food, and the environment: A systematic review and meta-analysis. Infect. Drug Resist..

[68] Asif M., Rahman H., Qasim M., Khan T.A., Ullah W., Jie Y. (2017). Molecular detection and antimicrobial resistance profile of zoonotic *Salmonella enteritidis* isolated from broiler chickens in Kohat, Pakistan. J. Chin. Med. Assoc..

[69] Sarker M.S., Mannan M.S., Ali M.Y., Bayzid M., Ahad A., Bupasha Z.B. (2019). Antibiotic resistance of *Escherichia coli* isolated from broilers sold at live bird markets in Chattogram, Bangladesh. J. Adv. Vet. Anim. Res..

[70] Amin M.B., Sraboni A.S., Hossain M.I., Roy S., Mozmader T.A.U., Unicomb L., Rousham E.K., Islam M.A. (2020). Occurrence and genetic characteristics of mcr-1-positive colistin-resistant E. coli from poultry environments in Bangladesh. J. Glob. Antimicrob. Resist..

[71] Al Azad M.A.R., Rahman M.M., Amin R., Begum M.I.A., Fries R., Husna A., Khairalla A.S., Badruzzaman A.T.M., El Zowalaty M.E., Lampang K.N. (2019). Susceptibility and multidrug resistance patterns of *Escherichia coli* isolated from cloacal swabs of live broiler chickens in Bangladesh. Pathogens.

[72] Maciuca I.E., Cummins M.L., Cozma A.P., Rimbu C.M., Guguianu E., Panzaru C., Licker M., Szekely E., Flonta M., Djordjevic S.P. (2019). Genetic Features of mcr-1 Mediated Colistin Resistance in CMY-2-Producing *Escherichia coli* From Romanian Poultry. Front. Microbiol..

[73] Wang Y., Tian G.B., Zhang R., Shen Y., Tyrrell J.M., Huang X., Zhou H., Lei L., Li H.Y., Doi Y. (2017). Prevalence, risk factors, outcomes, and molecular epidemiology of mcr-1-positive Enterobacteriaceae in patients and healthy adults from China: An epidemiological and clinical study. Lancet Infect. Dis..

[74] Wang R., Van Dorp L., Shaw L.P., Bradley P., Wang Q., Wang X., Jin L., Zhang Q., Liu Y., Rieux A. (2018). The global distribution and spread of the mobilized colistin resistance gene mcr-1. Nat. Commun..

[75] Islam S., Urmi U.L., Rana M., Sultana F., Jahan N., Hossain B., Iqbal S., Hossain M.M., Mosaddek A.S.M., Nahar S. (2020). High abundance of the colistin resistance gene mcr-1 in chicken gut bacteria in Bangladesh. Sci. Rep..

[76] Schrauwen E.J.A., Huizinga P., van Spreuwel N., Verhulst C., Kluytmans-van den Bergh M.F.Q., Kluytmans J.A.J.W. (2017). High prevalence of the mcr-1 gene in retail chicken meat in the Netherlands in 2015. Antimicrob. Resist. Infect. Control.

[77] Quesada A., Ugarte-Ruiz M., Iglesias M.R., Porrero M.C., Martínez R., Florez-Cuadrado D., Campos M.J., García M., Píriz S., Sáez J.L. (2016). Detection of plasmid mediated colistin resistance (MCR-1) in *Escherichia coli* and *Salmonella enterica* isolated from poultry and swine in Spain. Res. Vet. Sci..

[78] Shafiq M., Huang J., Ur Rahman S., Shah J.M., Chen L., Gao Y., Wang M., Wang L. (2019). High incidence of multidrug-resistant *Escherichia coli* coharboring mcr-1 and blaCTX-M-15 recovered from pigs. Infect. Drug Resist..

[79] Liu Y., Liu J.H. (2018). Monitoring Colistin Resistance in Food Animals, An Urgent Threat. Expert Rev. Anti-Infect. Ther..

[80] Huang X., Yu L., Chen X., Zhi C., Yao X., Liu Y., Wu S., Guo Z., Yi L., Zeng Z. (2017). High prevalence of colistin resistance and mcr-1 gene in *Escherichia coli* isolated from food animals in China. Front. Microbiol..

[81] Xu Y., Wei W., Lei S., Lin J., Srinivas S., Feng Y. (2018). An evolutionarily conserved mechanism for intrinsic and transferable polymyxin resistance. mBio.

[82] Manageiro V., Clemente L., Romão R., Silva C., Vieira L., Ferreira E., Caniça M. (2019). IncX4 plasmid carrying the new mcr-1.9 gene variant in a CTX-M-8-producing *Escherichia coli* isolate recovered from swine. Front. Microbiol..

[83] Fan J., Zhang L., He J., Zhao M., Loh B., Leptihn S., Yu Y., Hua X. (2020). Plasmid Dynamics of *mcr-1*-Positive *Salmonella* spp. in a General Hospital in China. Front. Microbiol..

[84] Liu H., Zhu B., Liang B., Xu X., Qiu S., Jia L., Li P., Yang L., Li Y., Xiang Y. (2018). A novel *mcr-1* variant carried by an IncI2-type plasmid identified from a multidrug resistant enterotoxigenic *Escherichia coli*. Front. Microbiol..

