# Supplementary material for: Molecular Detection of Colistin Resistance mcr-1 Gene in Multidrug-Resistant Escherichia coli Isolated from Chicken
Source: Antibiotics (Basel). 2022 Jan 13;11(1):97. doi: 10.3390/antibiotics11010097 (PMC8772701; doi:10.3390/antibiotics11010097)
Supplement: Supplementary file 1 [file antibiotics-11-00097-s001.zip › Supplementary File S1.pdf]

Supplementary File S1: Amplicons of *eaeA* gene sequence of *Escherichia coli* isolates (E1~E10).

>*Escherichia coli*_E1

CCAGGCTTCGTCACAGTTGCAGGCCTGGTTACAACATTATGGAACGGCAGAGGTTAATC
TGCAGAGTGGTAATAACTTTGACGGTAGTTCACTGGACTTCTTATTACCGTTCTATGATT
CCGAAAACATGCTGGCATTTGGTCAGGTCGGGGCGCGTTACATTGACTCCCGCTTTACG
GCAAATTTAGGTGCTGGCCAGCGTTTTTTCCTTCCTGAAAATATGTTGGGCTATAACGTC
TTCATTGATCAGGATTTTTCTGGTGATAATACCCGTTTAGGTATTGGTGGCG

>*Escherichia coli*_E2

CCAGGCTTCGTCACAGTTGCAGGCCTGGTTACAACATTATGGAACGGCAGAGGTTAATC
TGCAGAGTGGTAATAACTTTGACGGTAGTTCACTGGACTTCTTATTACCGTTCTATGATT
CCGAAAACATGCTGGCATTTGGTCAGGTCGGGGCGCGTTACATTGACTCCCGCTTTACG
GCAAATTTAGGTGCTGGCCAGCGTTTTTTCCTTCCTGAAAATATGTTGGGCTATAACGTC
TTCATTGATCAGGATTTTTCTGGTGATAATACCCGTTTAGGTATTGGTGGCG

>*Escherichia coli*_E3

CCAGGCTTCGTCACAGTTGCAGGCCTGGTTACAACATTATGGAACGGCAGAGGTTAATC
TGCAGAGTGGTAATAACTTTGACGGTAGTTCACTGGACTTCTTATTACCGTTCTATGATT
CCGAAAACATGCTGGCATTTGGTCAGGTCGGGGCGCGTTACATTGACTCCCGCTTTACG
GCAAATTTAGGTGCTGGCCAGCGTTTTTTCCTTCCTGAAAATATGTTGGGCTATAACGTC
TTCATTGATCAGGATTTTTCTGGTGATAATACCCGTTTAGGTATTGGTGGCG

>*Escherichia coli*_E4

CCAGGCTTCGTCACAGTTGCAGGCCTGGTTACAACATTATGGAACGGCAGAGGTTAATC
TGCAGAGTGGTAATAACTTTGACGGTAGTTCACTGGACTTCTTATTACCGTTCTATGATT
CCGAAAACATGCTGGCATTTGGTCAGGTCGGGGCGCGTTACATTGACTCCCGCTTTACG
GCAAATTTAGGTGCTGGCCAGCGTTTTTTCCTTCCTGAAAATATGTTGGGCTATAACGTC
TTCATTGATCAGGATTTTTCTGGTGATAATACCCGTTTAGGTATTGGTGGCG

>*Escherichia coli*_E5

CCAGGCTTCGTCACAGTTGCAGGCCTGGTTACAACATTATGGAACGGCAGAGGTTAATC
TGCAGAGTGGTAATAACTTTGACGGTAGTTCACTGGACTTCTTATTACCGTTCTATGATT
CCGAAAACATGCTGGCATTTGGTCAGGTCGGGGCGCGTTACATTGACTCCCGCTTTACG
GCAAATTTAGGTGCTGGCCAGCGTTTTTTCCTTCCTGAAAATATGTTGGGCTATAACGTC
TTCATTGATCAGGATTTTTCTGGTGATAATACCCGTTTAGGTATTGGTGGCG

>*Escherichia coli*_E6

CCAGGCTTCGTCACAGTTGCAGGCCTGGTTACAACATTATGGAACGGCAGAGGTTAATC
TGCAGAGTGGTAATAACTTTGACGGTAGTTCACTGGACTTCTTATTACCGTTCTATGATT
CCGAAAACATGCTGGCATTTGGTCAGGTCGGGGCGCGTTACATTGACTCCCGCTTTACG
GCAAATTTAGGTGCTGGCCAGCGTTTTTTCCTTCCTGAAAATATGTTGGGCTATAACGTC
TTCATTGATCAGGATTTTTCTGGTGATAATACCCGTTTAGGTATTGGTGGCG

>*Escherichia coli*_E7

CCAGGCTTCGTCACAGTTGCAGGCCTGGTTACAACATTATGGAACGGCAGAGGTTAATC
TGCAGAGTGGTAATAACTTTGACGGTAGTTCACTGGACTTCTTATTACCGTTCTATGATT
CCGAAAACATGCTGGCATTTGGTCAGGTCGGAGCGCGTTACATTGACTCCCGCTTTACG
GCAAATTTAGGTGCGGGTCAGCGTTTTTTCCTTCCTGAAAATATGTTGGGCTATAACGTC
TTCATTGATCAGGATTTTTCTGGTGATAATACCCGTTTAGGTATTGGTGGCG

>*Escherichia coli*_E8

CCAGGCTTCGTCACAGTTGCAGGCCTGGTTACAACATTATGGAACGGCAGAGGTTAATC
TGCAGAGTGGTAATAACTTTGACGGTAGTTCACTGGACTTCTTATTACCGTTCTATGATT
CCGAAAACATGCTGGCATTTGGTCAGGTCGGGGCGCGTTACATTGACTCCCGCTTTACG
GCAAATTTAGGTGCTGGCCAGCGTTTTTTCCTTCCTGAAAATATGTTGGGCTATAACGTC
TTCATTGATCAGGATTTTTCTGGTGATAATACCCGTTTAGGTATTGGTGGCG

>*Escherichia coli*_E9

CCAGGCTTCGTCACAGTTGCAGGCCTGGTTACAACATTATGGAACGGCAGAGGTTAATC
TGCAGAGTGGTAATAACTTTGACGGTAGTTCACTGGACTTCTTATTACCGTTCTATGATT
CCGAAAACATGCTGGCATTTGGTCAGGTCGGGGCGCGTTACATTGACTCCCGCTTTACG
GCAAATTTAGGTGCTGGCCAGCGTTTTTTCCTTCCTGAAAATATGTTGGGCTATAACGTC
TTCATTGATCAGGATTTTTCTGGTGATAATACCCGTTTAGGTATTGGTGGCG

>*Escherichia coli*_E10

CCAGGCTTCGTCACAGTTGCAGGCCTGGTTACAACATTATGGAACGGCAGAGGTTAATC
TGCAGAGTGGTAATAACTTTGACGGTAGTTCACTGGACTTCTTATTACCGTTCTATGATT
CCGAAAACATGCTGGCATTTGGTCAGGTCGGGGCGCGTTACATTGACTCCCGCTTTACG
GCAAATTTAGGTGCTGGCCAGCGTTTTTTCCTTCCTGAAAATATGTTGGGCTATAACGTC
TTCATTGATCAGGATTTTTCTGGTGATAATACCCGTTTAGGTATTGGTGGCG
