# Supplementary material for: Molecular Detection of Colistin Resistance mcr-1 Gene in Multidrug-Resistant Escherichia coli Isolated from Chicken
Source: Antibiotics (Basel). 2022 Jan 13;11(1):97. doi: 10.3390/antibiotics11010097 (PMC8772701; doi:10.3390/antibiotics11010097)
Supplement: Supplementary file 1 [file antibiotics-11-00097-s001.zip › Supplementary File S2.pdf]

Supplementary File S2: A total of 91 MCR-1 proteins of *E. coli* origin retrieved from NCBI database.

>strain_L40_MCR-1.1_Nigeria_1

ATGATGCAGCATACTTCTGTGTGGTACCGACGCTCGGTCAGTCCGTTTGTTCTTGTGGCGAGTGTTGCCGTTTTCTTGAC

CGCGACCGCCAATCTTACCTTTTTTGATAAAATCAGCCAAACCTATCCCATCGCGGACAATCTCGGCTTTGTGCTGACGA

TCGCTGTCGTGCTCTTTGGCGCGATGCTACTGATCACCACGCTGTTATCATCGTATCGCTATGTGCTAAAGCCTGTGTTG

ATTTTGCTATTAATCATGGGCGCGGTGACCAGTTATTTTACTGACACTTATGGCACGGTCTATGATACGACCATGCTCCA

AAATGCCCTACAGACCGACCAAGCCGAGACCAAGGATCTATTAAACGCAGCGTTTATCATGCGTATCATTGGTTTGGGTG

TGCTACCAAGTTTGCTTGTGGCTTTTGTTAAGGTGGATTATCCGACTTGGGGCAAGGGTTTGATGCGCCGATTGGGCTTG

ATCGTGGCAAGTCTTGCGCTGATTTTACTGCCTGTGGTGGCGTTCAGCAGTCATTATGCCAGTTTCTTTCGCGTGCATAA

GCCGCTGCGTAGCTATGTCAATCCGATCATGCCAATCTACTCGGTGGGTAAGCTTGCCAGTATTGAGTATAAAAAAGCCA

GTGCGCCAAAAGATACCATTTATCACGCCAAAGACGCGGTACAAGCAACCAAGCCTGATATGCGTAAGCCACGCCTAGTG

GTGTTCGTCGTCGGTGAGACGGCACGCGCCGATCATGTCAGCTTCAATGGCTATGAGCGCGATACTTTCCCACAGCTTGC

CAAGATCGATGGCGTGACCAATTTTAGCAATGTCACATCGTGCGGCACATCGACGGCGTATTCTGTGCCGTGTATGTTCA

GCTATCTGGGCGCGGATGAGTATGATGTCGATACCGCCAAATACCAAGAAAATGTGCTGGATACGCTGGATCGCTTGGGC

GTAAGTATCTTGTGGCGTGATAATAATTCGGACTCAAAAGGCGTGATGGATAAGCTGCCAAAAGCGCAATTTGCCGATTA

TAAATCCGCGACCAACAACGCCATCTGCAACACCAATCCTTATAACGAATGCCGCGATGTCGGTATGCTCGTTGGCTTAG

ATGACTTTGTCGCTGCCAATAACGGCAAAGATATGCTGATCATGCTGCACCAAATGGGCAATCACGGGCCTGCGTATTTT

AAGCGATATGATGAAAAGTTTGCCAAATTCACGCCAGTGTGTGAAGGTAATGAGCTTGCCAAGTGCGAACATCAGTCCTT

GATCAATGCTTATGACAATGCCTTGCTTGCCACCGATGATTTCATCGCTCAAAGTATCCAGTGGCTGCAGACGCACAGCA

ATGCCTATGATGTCTCAATGCTGTATGTCAGCGATCATGGCGAAAGTCTGGGTGAGAACGGTGTCTATCTACATGGTATG

CCAAATGCCTTTGCACCAAAAGAACAGCGCAGTGTGCCTGCATTTTTCTGGACGGATAAGCAAACTGGCATCACGCCAAT

GGCAACCGATACCGTCCTGACCCATGACGCGATCACGCCGACATTATTAAAGCTGTTTGATGTCACCGCGGACAAAGTCA

AAGACCGCACCGCATTCATCCGCTGA

>strain_L39_MCR-1.1_Nigeria_2

ATGATGCAGCATACTTCTGTGTGGTACCGACGCTCGGTCAGTCCGTTTGTTCTTGTGGCGAGTGTTGCCGTTTTCTTGAC

CGCGACCGCCAATCTTACCTTTTTTGATAAAATCAGCCAAACCTATCCCATCGCGGACAATCTCGGCTTTGTGCTGACGA

TCGCTGTCGTGCTCTTTGGCGCGATGCTACTGATCACCACGCTGTTATCATCGTATCGCTATGTGCTAAAGCCTGTGTTG

ATTTTGCTATTAATCATGGGCGCGGTGACCAGTTATTTTACTGACACTTATGGCACGGTCTATGATACGACCATGCTCCA

AAATGCCCTACAGACCGACCAAGCCGAGACCAAGGATCTATTAAACGCAGCGTTTATCATGCGTATCATTGGTTTGGGTG

TGCTACCAAGTTTGCTTGTGGCTTTTGTTAAGGTGGATTATCCGACTTGGGGCAAGGGTTTGATGCGCCGATTGGGCTTG

ATCGTGGCAAGTCTTGCGCTGATTTTACTGCCTGTGGTGGCGTTCAGCAGTCATTATGCCAGTTTCTTTCGCGTGCATAA

GCCGCTGCGTAGCTATGTCAATCCGATCATGCCAATCTACTCGGTGGGTAAGCTTGCCAGTATTGAGTATAAAAAAGCCA

GTGCGCCAAAAGATACCATTTATCACGCCAAAGACGCGGTACAAGCAACCAAGCCTGATATGCGTAAGCCACGCCTAGTG

GTGTTCGTCGTCGGTGAGACGGCACGCGCCGATCATGTCAGCTTCAATGGCTATGAGCGCGATACTTTCCCACAGCTTGC

CAAGATCGATGGCGTGACCAATTTTAGCAATGTCACATCGTGCGGCACATCGACGGCGTATTCTGTGCCGTGTATGTTCA

GCTATCTGGGCGCGGATGAGTATGATGTCGATACCGCCAAATACCAAGAAAATGTGCTGGATACGCTGGATCGCTTGGGC

GTAAGTATCTTGTGGCGTGATAATAATTCGGACTCAAAAGGCGTGATGGATAAGCTGCCAAAAGCGCAATTTGCCGATTA

TAAATCCGCGACCAACAACGCCATCTGCAACACCAATCCTTATAACGAATGCCGCGATGTCGGTATGCTCGTTGGCTTAG

ATGACTTTGTCGCTGCCAATAACGGCAAAGATATGCTGATCATGCTGCACCAAATGGGCAATCACGGGCCTGCGTATTTT

AAGCGATATGATGAAAAGTTTGCCAAATTCACGCCAGTGTGTGAAGGTAATGAGCTTGCCAAGTGCGAACATCAGTCCTT

GATCAATGCTTATGACAATGCCTTGCTTGCCACCGATGATTTCATCGCTCAAAGTATCCAGTGGCTGCAGACGCACAGCA

ATGCCTATGATGTCTCAATGCTGTATGTCAGCGATCATGGCGAAAGTCTGGGTGAGAACGGTGTCTATCTACATGGTATG

CCAAATGCCTTTGCACCAAAAGAACAGCGCAGTGTGCCTGCATTTTTCTGGACGGATAAGCAAACTGGCATCACGCCAAT

GGCAACCGATACCGTCCTGACCCATGACGCGATCACGCCGACATTATTAAAGCTGTTTGATGTCACCGCGGACAAAGTCA

AAGACCGCACCGCATTCATCCGCTGA

>MT070408.1 Escherichia coli strain L36 phosphoethanolamine--lipid A transferase MCR-1.1 (mcr-1) gene, mcr-1.1 allele, complete cds

ATGATGCAGCATACTTCTGTGTGGTACCGACGCTCGGTCAGTCCGTTTGTTCTTGTGGCGAGTGTTGCCGTTTTCTTGAC

CGCGACCGCCAATCTTACCTTTTTTGATAAAATCAGCCAAACCTATCCCATCGCGGACAATCTCGGCTTTGTGCTGACGA

TCGCTGTCGTGCTCTTTGGCGCGATGCTACTGATCACCACGCTGTTATCATCGTATCGCTATGTGCTAAAGCCTGTGTTG

ATTTTGCTATTAATCATGGGCGCGGTGACCAGTTATTTTACTGACACTTATGGCACGGTCTATGATACGACCATGCTCCA

AAATGCCCTACAGACCGACCAAGCCGAGACCAAGGATCTATTAAACGCAGCGTTTATCATGCGTATCATTGGTTTGGGTG

TGCTACCAAGTTTGCTTGTGGCTTTTGTTAAGGTGGATTATCCGACTTGGGGCAAGGGTTTGATGCGCCGATTGGGCTTG

ATCGTGGCAAGTCTTGCGCTGATTTTACTGCCTGTGGTGGCGTTCAGCAGTCATTATGCCAGTTTCTTTCGCGTGCATAA

GCCGCTGCGTAGCTATGTCAATCCGATCATGCCAATCTACTCGGTGGGTAAGCTTGCCAGTATTGAGTATAAAAAAGCCA

GTGCGCCAAAAGATACCATTTATCACGCCAAAGACGCGGTACAAGCAACCAAGCCTGATATGCGTAAGCCACGCCTAGTG

GTGTTCGTCGTCGGTGAGACGGCACGCGCCGATCATGTCAGCTTCAATGGCTATGAGCGCGATACTTTCCCACAGCTTGC

CAAGATCGATGGCGTGACCAATTTTAGCAATGTCACATCGTGCGGCACATCGACGGCGTATTCTGTGCCGTGTATGTTCA

GCTATCTGGGCGCGGATGAGTATGATGTCGATACCGCCAAATACCAAGAAAATGTGCTGGATACGCTGGATCGCTTGGGC

GTAAGTATCTTGTGGCGTGATAATAATTCGGACTCAAAAGGCGTGATGGATAAGCTGCCAAAAGCGCAATTTGCCGATTA

TAAATCCGCGACCAACAACGCCATCTGCAACACCAATCCTTATAACGAATGCCGCGATGTCGGTATGCTCGTTGGCTTAG

ATGACTTTGTCGCTGCCAATAACGGCAAAGATATGCTGATCATGCTGCACCAAATGGGCAATCACGGGCCTGCGTATTTT

AAGCGATATGATGAAAAGTTTGCCAAATTCACGCCAGTGTGTGAAGGTAATGAGCTTGCCAAGTGCGAACATCAGTCCTT

GATCAATGCTTATGACAATGCCTTGCTTGCCACCGATGATTTCATCGCTCAAAGTATCCAGTGGCTGCAGACGCACAGCA

ATGCCTATGATGTCTCAATGCTGTATGTCAGCGATCATGGCGAAAGTCTGGGTGAGAACGGTGTCTATCTACATGGTATG

CCAAATGCCTTTGCACCAAAAGAACAGCGCAGTGTGCCTGCATTTTTCTGGACGGATAAGCAAACTGGCATCACGCCAAT

GGCAACCGATACCGTCCTGACCCATGACGCGATCACGCCGACATTATTAAAGCTGTTTGATGTCACCGCGGACAAAGTCA

AAGACCGCACCGCATTCATCCGCTGA

>MT070407.1 Escherichia coli strain L29 phosphoethanolamine--lipid A transferase MCR-1.1 (mcr-1) gene, mcr-1.1 allele, complete cds

ATGATGCAGCATACTTCTGTGTGGTACCGACGCTCGGTCAGTCCGTTTGTTCTTGTGGCGAGTGTTGCCGTTTTCTTGAC

CGCGACCGCCAATCTTACCTTTTTTGATAAAATCAGCCAAACCTATCCCATCGCGGACAATCTCGGCTTTGTGCTGACGA

TCGCTGTCGTGCTCTTTGGCGCGATGCTACTGATCACCACGCTGTTATCATCGTATCGCTATGTGCTAAAGCCTGTGTTG

ATTTTGCTATTAATCATGGGCGCGGTGACCAGTTATTTTACTGACACTTATGGCACGGTCTATGATACGACCATGCTCCA

AAATGCCCTACAGACCGACCAAGCCGAGACCAAGGATCTATTAAACGCAGCGTTTATCATGCGTATCATTGGTTTGGGTG

TGCTACCAAGTTTGCTTGTGGCTTTTGTTAAGGTGGATTATCCGACTTGGGGCAAGGGTTTGATGCGCCGATTGGGCTTG

ATCGTGGCAAGTCTTGCGCTGATTTTACTGCCTGTGGTGGCGTTCAGCAGTCATTATGCCAGTTTCTTTCGCGTGCATAA

GCCGCTGCGTAGCTATGTCAATCCGATCATGCCAATCTACTCGGTGGGTAAGCTTGCCAGTATTGAGTATAAAAAAGCCA

GTGCGCCAAAAGATACCATTTATCACGCCAAAGACGCGGTACAAGCAACCAAGCCTGATATGCGTAAGCCACGCCTAGTG

GTGTTCGTCGTCGGTGAGACGGCACGCGCCGATCATGTCAGCTTCAATGGCTATGAGCGCGATACTTTCCCACAGCTTGC

CAAGATCGATGGCGTGACCAATTTTAGCAATGTCACATCGTGCGGCACATCGACGGCGTATTCTGTGCCGTGTATGTTCA

GCTATCTGGGCGCGGATGAGTATGATGTCGATACCGCCAAATACCAAGAAAATGTGCTGGATACGCTGGATCGCTTGGGC

GTAAGTATCTTGTGGCGTGATAATAATTCGGACTCAAAAGGCGTGATGGATAAGCTGCCAAAAGCGCAATTTGCCGATTA

TAAATCCGCGACCAACAACGCCATCTGCAACACCAATCCTTATAACGAATGCCGCGATGTCGGTATGCTCGTTGGCTTAG

ATGACTTTGTCGCTGCCAATAACGGCAAAGATATGCTGATCATGCTGCACCAAATGGGCAATCACGGGCCTGCGTATTTT

AAGCGATATGATGAAAAGTTTGCCAAATTCACGCCAGTGTGTGAAGGTAATGAGCTTGCCAAGTGCGAACATCAGTCCTT

GATCAATGCTTATGACAATGCCTTGCTTGCCACCGATGATTTCATCGCTCAAAGTATCCAGTGGCTGCAGACGCACAGCA

ATGCCTATGATGTCTCAATGCTGTATGTCAGCGATCATGGCGAAAGTCTGGGTGAGAACGGTGTCTATCTACATGGTATG

CCAAATGCCTTTGCACCAAAAGAACAGCGCAGTGTGCCTGCATTTTTCTGGACGGATAAGCAAACTGGCATCACGCCAAT

GGCAACCGATACCGTCCTGACCCATGACGCGATCACGCCGACATTATTAAAGCTGTTTGATGTCACCGCGGACAAAGTCA

AAGACCGCACCGCATTCATCCGCTGA

>MT070406.1 Escherichia coli strain L27 phosphoethanolamine--lipid A transferase MCR-1.1 (mcr-1) gene, mcr-1.1 allele, complete cds

ATGATGCAGCATACTTCTGTGTGGTACCGACGCTCGGTCAGTCCGTTTGTTCTTGTGGCGAGTGTTGCCGTTTTCTTGAC

CGCGACCGCCAATCTTACCTTTTTTGATAAAATCAGCCAAACCTATCCCATCGCGGACAATCTCGGCTTTGTGCTGACGA

TCGCTGTCGTGCTCTTTGGCGCGATGCTACTGATCACCACGCTGTTATCATCGTATCGCTATGTGCTAAAGCCTGTGTTG

ATTTTGCTATTAATCATGGGCGCGGTGACCAGTTATTTTACTGACACTTATGGCACGGTCTATGATACGACCATGCTCCA

AAATGCCCTACAGACCGACCAAGCCGAGACCAAGGATCTATTAAACGCAGCGTTTATCATGCGTATCATTGGTTTGGGTG

TGCTACCAAGTTTGCTTGTGGCTTTTGTTAAGGTGGATTATCCGACTTGGGGCAAGGGTTTGATGCGCCGATTGGGCTTG

ATCGTGGCAAGTCTTGCGCTGATTTTACTGCCTGTGGTGGCGTTCAGCAGTCATTATGCCAGTTTCTTTCGCGTGCATAA

GCCGCTGCGTAGCTATGTCAATCCGATCATGCCAATCTACTCGGTGGGTAAGCTTGCCAGTATTGAGTATAAAAAAGCCA

GTGCGCCAAAAGATACCATTTATCACGCCAAAGACGCGGTACAAGCAACCAAGCCTGATATGCGTAAGCCACGCCTAGTG

GTGTTCGTCGTCGGTGAGACGGCACGCGCCGATCATGTCAGCTTCAATGGCTATGAGCGCGATACTTTCCCACAGCTTGC

CAAGATCGATGGCGTGACCAATTTTAGCAATGTCACATCGTGCGGCACATCGACGGCGTATTCTGTGCCGTGTATGTTCA

GCTATCTGGGCGCGGATGAGTATGATGTCGATACCGCCAAATACCAAGAAAATGTGCTGGATACGCTGGATCGCTTGGGC

GTAAGTATCTTGTGGCGTGATAATAATTCGGACTCAAAAGGCGTGATGGATAAGCTGCCAAAAGCGCAATTTGCCGATTA

TAAATCCGCGACCAACAACGCCATCTGCAACACCAATCCTTATAACGAATGCCGCGATGTCGGTATGCTCGTTGGCTTAG

ATGACTTTGTCGCTGCCAATAACGGCAAAGATATGCTGATCATGCTGCACCAAATGGGCAATCACGGGCCTGCGTATTTT

AAGCGATATGATGAAAAGTTTGCCAAATTCACGCCAGTGTGTGAAGGTAATGAGCTTGCCAAGTGCGAACATCAGTCCTT

GATCAATGCTTATGACAATGCCTTGCTTGCCACCGATGATTTCATCGCTCAAAGTATCCAGTGGCTGCAGACGCACAGCA

ATGCCTATGATGTCTCAATGCTGTATGTCAGCGATCATGGCGAAAGTCTGGGTGAGAACGGTGTCTATCTACATGGTATG

CCAAATGCCTTTGCACCAAAAGAACAGCGCAGTGTGCCTGCATTTTTCTGGACGGATAAGCAAACTGGCATCACGCCAAT

GGCAACCGATACCGTCCTGACCCATGACGCGATCACGCCGACATTATTAAAGCTGTTTGATGTCACCGCGGACAAAGTCA

AAGACCGCACCGCATTCATCCGCTGA

>MT070405.1 Escherichia coli strain L26 phosphoethanolamine--lipid A transferase MCR-1.1 (mcr-1) gene, mcr-1.1 allele, complete cds

ATGATGCAGCATACTTCTGTGTGGTACCGACGCTCGGTCAGTCCGTTTGTTCTTGTGGCGAGTGTTGCCGTTTTCTTGAC

CGCGACCGCCAATCTTACCTTTTTTGATAAAATCAGCCAAACCTATCCCATCGCGGACAATCTCGGCTTTGTGCTGACGA

TCGCTGTCGTGCTCTTTGGCGCGATGCTACTGATCACCACGCTGTTATCATCGTATCGCTATGTGCTAAAGCCTGTGTTG

ATTTTGCTATTAATCATGGGCGCGGTGACCAGTTATTTTACTGACACTTATGGCACGGTCTATGATACGACCATGCTCCA

AAATGCCCTACAGACCGACCAAGCCGAGACCAAGGATCTATTAAACGCAGCGTTTATCATGCGTATCATTGGTTTGGGTG

TGCTACCAAGTTTGCTTGTGGCTTTTGTTAAGGTGGATTATCCGACTTGGGGCAAGGGTTTGATGCGCCGATTGGGCTTG

ATCGTGGCAAGTCTTGCGCTGATTTTACTGCCTGTGGTGGCGTTCAGCAGTCATTATGCCAGTTTCTTTCGCGTGCATAA

GCCGCTGCGTAGCTATGTCAATCCGATCATGCCAATCTACTCGGTGGGTAAGCTTGCCAGTATTGAGTATAAAAAAGCCA

GTGCGCCAAAAGATACCATTTATCACGCCAAAGACGCGGTACAAGCAACCAAGCCTGATATGCGTAAGCCACGCCTAGTG

GTGTTCGTCGTCGGTGAGACGGCACGCGCCGATCATGTCAGCTTCAATGGCTATGAGCGCGATACTTTCCCACAGCTTGC

CAAGATCGATGGCGTGACCAATTTTAGCAATGTCACATCGTGCGGCACATCGACGGCGTATTCTGTGCCGTGTATGTTCA

GCTATCTGGGCGCGGATGAGTATGATGTCGATACCGCCAAATACCAAGAAAATGTGCTGGATACGCTGGATCGCTTGGGC

GTAAGTATCTTGTGGCGTGATAATAATTCGGACTCAAAAGGCGTGATGGATAAGCTGCCAAAAGCGCAATTTGCCGATTA

TAAATCCGCGACCAACAACGCCATCTGCAACACCAATCCTTATAACGAATGCCGCGATGTCGGTATGCTCGTTGGCTTAG

ATGACTTTGTCGCTGCCAATAACGGCAAAGATATGCTGATCATGCTGCACCAAATGGGCAATCACGGGCCTGCGTATTTT

AAGCGATATGATGAAAAGTTTGCCAAATTCACGCCAGTGTGTGAAGGTAATGAGCTTGCCAAGTGCGAACATCAGTCCTT

GATCAATGCTTATGACAATGCCTTGCTTGCCACCGATGATTTCATCGCTCAAAGTATCCAGTGGCTGCAGACGCACAGCA

ATGCCTATGATGTCTCAATGCTGTATGTCAGCGATCATGGCGAAAGTCTGGGTGAGAACGGTGTCTATCTACATGGTATG

CCAAATGCCTTTGCACCAAAAGAACAGCGCAGTGTGCCTGCATTTTTCTGGACGGATAAGCAAACTGGCATCACGCCAAT

GGCAACCGATACCGTCCTGACCCATGACGCGATCACGCCGACATTATTAAAGCTGTTTGATGTCACCGCGGACAAAGTCA

AAGACCGCACCGCATTCATCCGCTGA

>MT070404.1 Escherichia coli strain L23 phosphoethanolamine--lipid A transferase MCR-1.1 (mcr-1) gene, mcr-1.1 allele, complete cds

ATGATGCAGCATACTTCTGTGTGGTACCGACGCTCGGTCAGTCCGTTTGTTCTTGTGGCGAGTGTTGCCGTTTTCTTGAC

CGCGACCGCCAATCTTACCTTTTTTGATAAAATCAGCCAAACCTATCCCATCGCGGACAATCTCGGCTTTGTGCTGACGA

TCGCTGTCGTGCTCTTTGGCGCGATGCTACTGATCACCACGCTGTTATCATCGTATCGCTATGTGCTAAAGCCTGTGTTG

ATTTTGCTATTAATCATGGGCGCGGTGACCAGTTATTTTACTGACACTTATGGCACGGTCTATGATACGACCATGCTCCA

AAATGCCCTACAGACCGACCAAGCCGAGACCAAGGATCTATTAAACGCAGCGTTTATCATGCGTATCATTGGTTTGGGTG

TGCTACCAAGTTTGCTTGTGGCTTTTGTTAAGGTGGATTATCCGACTTGGGGCAAGGGTTTGATGCGCCGATTGGGCTTG

ATCGTGGCAAGTCTTGCGCTGATTTTACTGCCTGTGGTGGCGTTCAGCAGTCATTATGCCAGTTTCTTTCGCGTGCATAA

GCCGCTGCGTAGCTATGTCAATCCGATCATGCCAATCTACTCGGTGGGTAAGCTTGCCAGTATTGAGTATAAAAAAGCCA

GTGCGCCAAAAGATACCATTTATCACGCCAAAGACGCGGTACAAGCAACCAAGCCTGATATGCGTAAGCCACGCCTAGTG

GTGTTCGTCGTCGGTGAGACGGCACGCGCCGATCATGTCAGCTTCAATGGCTATGAGCGCGATACTTTCCCACAGCTTGC

CAAGATCGATGGCGTGACCAATTTTAGCAATGTCACATCGTGCGGCACATCGACGGCGTATTCTGTGCCGTGTATGTTCA

GCTATCTGGGCGCGGATGAGTATGATGTCGATACCGCCAAATACCAAGAAAATGTGCTGGATACGCTGGATCGCTTGGGC

GTAAGTATCTTGTGGCGTGATAATAATTCGGACTCAAAAGGCGTGATGGATAAGCTGCCAAAAGCGCAATTTGCCGATTA

TAAATCCGCGACCAACAACGCCATCTGCAACACCAATCCTTATAACGAATGCCGCGATGTCGGTATGCTCGTTGGCTTAG

ATGACTTTGTCGCTGCCAATAACGGCAAAGATATGCTGATCATGCTGCACCAAATGGGCAATCACGGGCCTGCGTATTTT

AAGCGATATGATGAAAAGTTTGCCAAATTCACGCCAGTGTGTGAAGGTAATGAGCTTGCCAAGTGCGAACATCAGTCCTT

GATCAATGCTTATGACAATGCCTTGCTTGCCACCGATGATTTCATCGCTCAAAGTATCCAGTGGCTGCAGACGCACAGCA

ATGCCTATGATGTCTCAATGCTGTATGTCAGCGATCATGGCGAAAGTCTGGGTGAGAACGGTGTCTATCTACATGGTATG

CCAAATGCCTTTGCACCAAAAGAACAGCGCAGTGTGCCTGCATTTTTCTGGACGGATAAGCAAACTGGCATCACGCCAAT

GGCAACCGATACCGTCCTGACCCATGACGCGATCACGCCGACATTATTAAAGCTGTTTGATGTCACCGCGGACAAAGTCA

AAGACCGCACCGCATTCATCCGCTGA

>MN879260.1 Escherichia coli strain SAUVM_E7 phosphoethanolamine--lipid A transferase MCR-1.1 (mcr-1) gene, mcr-1.1 allele, partial cds

ATGATGCAGCATACTTCTGTGTGGTACCGACGCTCGGTCAGTCCGTTTGTTCTTGTGGCGAGTGTTGCCGTTTTCTTGAC

CGCGACCGCCAATCTTACCTTTTTTGATAAAATCAGCCAAACCTATCCCATCGCGGACAATCTCGGCTTTGTGCTGACGA

TCGCTGTCGTGCTCTTTGGCGCGATGCTACTGATCACCACGCTGTTATCATCGTATCGCTATGTGCTAAAGCCTGTGTTG

ATTTTGCTATTAATCATGGGCGCGGTGACCAGTTATTTTACTGACACTTATGGCACGGTCTATGATACGACCATGCTCCA

AAATGCCCTACAGACCGACCAAGCCGAGACCAAGGATCTATTAAACGCAGCGTTTATCATGCGTATCATTGGTTTGGGTG

TGCTACCAAGTTTGCTTGTGGCTTTTGTTAAGGTGGATTATCCGACTTGGGGCAAGGGTTTGATGCGCCGATTGGGCTTG

ATCGTGGCAAGTCTTGCGCTGATTTTACTGCCTGTGGTGGCGTTCAGCAGTCATTATGCCAGTTTCTTTCGCGTGCATAA

GCCGCTGCGTAGCTATGTCAATCCGATCATGCCAATCTACTCGGTGGGTAAGCTTGCCAGTATTGAGTATAAAAAAGCCA

GTGCGCCAAAAGATACCATTTATCACGCCAAAGACGCGGTACAAGCAACCAAGCCTGATATGCGTAAGCCACGCCTAGTG

GTGTTCGTCGTCGGTGAGACGGCACGCGCCGATCATGTCAGCTTCAATGGCTATGAGCGCGATACTTTCCCACAGCTTGC

CAAGATCGATGGCGTGACCAATTTTAGCAATGTCACATCGTGCGGCACATCGACGGCGTATTCTGTGCCGTGTATGTTCA

GCTATCTGGGCGCGGATGAGTATGATGTCGATACCGCCAAATACCAAGAAAATGTGCTGGATACGCTGGATCGCTTGGGC

GTAAGTATCTTGTGGCGTGATAATAATTCGGACTCAAAAGGCGTGATGGATAAGCTGCCAAAAGCGCAATTTGCCGATTA

TAAATCCGCGACCAACAACGCCATCTGCAACACCAATCCTTATAACGAATGCCGCGATGTCGGTATGCTCGTTGGCTTAG

ATGACTTTGTCGCTGCCAATAACGGCAAAGATATGCTGATCATGCTGCACCAAATGGGCAATCACGGGCCTGCGTATTTT

AAGCGATATGATGAAAAGTTTGCCAAATTCACGCCAGTGTGTGAAGGTAATGAGCTTGCCAAGTGCGAACATCAGTCCTT

GATCAATGCTTATGACAATGCCTTGCTTGCCACCGATGATTTCATCGCTCAAAGTATCCAGTGGCTGCAGACGCACAGCA

ATGCCTATGATGTCTCAATGCTGTATGTCAGCGATCATGGCGAAAGTCTGGGTGAGAACGGTGTCTATCTACATGGTATG

CCAAATGCCTTTGCACCAAAAGAACAGCGCAGTGTGCCTGCATTTTTCTGGACGGATAAGCAAACTGGCATCACGCCAAT

GGCAACCGATACCGTCCTGACCCATGACGCGATCACGCCGACATTATTAAAGCTGTTTGATGTCACCGCGGACAAAGTCA

AAGACCGCACCGCATTCATCCGCTGA

>MN879256.1 Escherichia coli strain SAUVM_E2 phosphoethanolamine--lipid A transferase MCR-1.1 (mcr-1) gene, mcr-1.1 allele, partial cds

ATGATGCAGCATACTTCTGTGTGGTACCGACGCTCGGTCAGTCCGTTTGTTCTTGTGGCGAGTGTTGCCGTTTTCTTGAC

CGCGACCGCCAATCTTACCTTTTTTGATAAAATCAGCCAAACCTATCCCATCGCGGACAATCTCGGCTTTGTGCTGACGA

TCGCTGTCGTGCTCTTTGGCGCGATGCTACTGATCACCACGCTGTTATCATCGTATCGCTATGTGCTAAAGCCTGTGTTG

ATTTTGCTATTAATCATGGGCGCGGTGACCAGTTATTTTACTGACACTTATGGCACGGTCTATGATACGACCATGCTCCA

AAATGCCCTACAGACCGACCAAGCCGAGACCAAGGATCTATTAAACGCAGCGTTTATCATGCGTATCATTGGTTTGGGTG

TGCTACCAAGTTTGCTTGTGGCTTTTGTTAAGGTGGATTATCCGACTTGGGGCAAGGGTTTGATGCGCCGATTGGGCTTG

ATCGTGGCAAGTCTTGCGCTGATTTTACTGCCTGTGGTGGCGTTCAGCAGTCATTATGCCAGTTTCTTTCGCGTGCATAA

GCCGCTGCGTAGCTATGTCAATCCGATCATGCCAATCTACTCGGTGGGTAAGCTTGCCAGTATTGAGTATAAAAAAGCCA

GTGCGCCAAAAGATACCATTTATCACGCCAAAGACGCGGTACAAGCAACCAAGCCTGATATGCGTAAGCCACGCCTAGTG

GTGTTCGTCGTCGGTGAGACGGCACGCGCCGATCATGTCAGCTTCAATGGCTATGAGCGCGATACTTTCCCACAGCTTGC

CAAGATCGATGGCGTGACCAATTTTAGCAATGTCACATCGTGCGGCACATCGACGGCGTATTCTGTGCCGTGTATGTTCA
GCTATCTGGGCGCGGATGAGTATGATGTCGATACCGCCAAATACCAAGAAAATGTGCTGGATACGCTGGATCGCTTGGGC
GTAAGTATCTTGTGGCGTGATAATAATTCGGACTCAAAAGGCGTGATGGATAAGCTGCCAAAAGCGCAATTTGCCGATTA
TAAATCCGCGACCAACAACGCCATCTGCAACACCAATCCTTATAACGAATGCCGCGATGTCGGTATGCTCGTTGGCTTAG
ATGACTTTGTCGCTGCCAATAACGGCAAAGATATGCTGATCATGCTGCACCAAATGGGCAATCACGGGCCTGCGTATTTT
AAGCGATATGATGAAAAGTTTGCCAAATTCACGCCAGTGTGTGAAGGTAATGAGCTTGCCAAGTGCGAACATCAGTCCTT
GATCAATGCTTATGACAATGCCTTGCTTGCCACCGATGATTTCATCGCTCAAAGTATCCAGTGGCTGCAGACGCACAGCA
ATGCCTATGATGTCTCAATGCTGTATGTCAGCGATCATGGCGAAAGTCTGGGTGAGAACGGTGTCTATCTACATGGTATG
CCAAATGCCTTTGCACCAAAAGAACAGCGCAGTGTGCCTGCATTTTTCTGGACGGATAAGCAAACTGGCATCACGCCAAT
GGCAACCGATACCGTCCTGACCCATGACGCGATCACGCCGACATTATTAAAGCTGTTTGATGTCACCGCGGACAAAGTCA
AAGACCGCACCGCATTCATCCGCTGA

>MN879255.1 Escherichia coli strain SAUVM_E1 MCR-1 family phosphoethanolamine--lipid A transferase (mcr-1) gene, partial cds

ATGATGCAGCATACTTCTGTGTGGTACCGACGCTCGGTCAGTCCGTTTGTTCTTGTGGCGAGTGTTGCCGTTTTCTTGAC
CGCGACCGCCAATCTTACCTTTTTTGATAAAATCAGCCAAACCTATCCCATCGCGGACAATCTCGGCTTTGTGCTGACGA
TCGCTGTCGTGCTCTTTGGCGCGATGCTACTGATCACCACGCTGTTATCATCGTATCGCTATGTGCTAAAGCCTGTGTTG
ATTTTGCTATTAATCATGGGCGCGGTGACCAGTTATTTTACTGACACTTATGGCACGGTCTATGATACGACCATGCTCCA
AAATGCCCTACAGACCGACCAAGCCGAGACCAAGGATCTATTAAACGCAGCGTTTATCATGCGTATCATTGGTTTGGGTG
TGCTACCAAGTTTGCTTGTGGCTTTTGTTAANGTGGATTATCCGACTTGGGGCAAGGGTTTGATGCGCCGATTGGGCTTG
ATCGTGGCAAGTCTTGCGCTGATTTTACTGCCTGTGGTGGCGTTCAGCAGTCATTATGCCAGTTTCTTTCGCGTGCATAA

GCCGCTGCGTAGCTATGTCAATCCGATCATGCCAATCTACTCGGTGGGTAAGCTTGCCAGTATTGAGTATAAAAAAGCCA

GTGCGCCAAAAGATACCATTTATCACGCCAAAGACGCGGTACAAGCAACCAAGCCTGATATGCGTAAGCCACGCCTAGTG

GTGTTCGTCGTCGGTGAGACGGCACGCGCCGATCATGTCAGCTTCAATGGCTATGAGCGCGATACTTTCCCACAGCTTGC

CAAGATCGATGGCGTGACCAATTTTAGCAATGTCACATCGTGCGGCACATCGACGGCGTATTCTGTGCCGTGTATGTTCA

GCTATCTGGGCGCGGATGAGTATGATGTCGATACCGCCAAATACCAAGAAAATGTGCTGGATACGCTGGATCGCTTGGGC

GTAAGTATCTTGTGGCGTGATAATAATTCGGACTCAAAAGGCGTGATGGATAAGCTGCCAAAAGCGCAATTTGCCGATTA

TAAATCCGCGACCAACAACGCCATCTGCAACACCAATCCTTATAACGAATGCCGCGATGTCGGTATGCTCGTTGGCTTAG

ATGACTTTGTCGCTGCCAATAACGGCAAAGATATGCTGATCATGCTGCACCAAATGGGCAATCACGGGCCTGCGTATTTT

AAGCGATATGATGAAAAGTTTGCCAAATTCACGCCAGTGTGTGAAGGTAATGAGCTTGCCAAGTGCGAACATCAGTCCTT

GATCAATGCTTATGACAATGCCTTGCTTGCCACCGATGATTTCATCGCTCAAAGTATCCAGTGGCTGCAGACGCACAGCA

ATGCCTATGATGTCTCAATGCTGTATGTCAGCGATCATGGCGAAAGTCTGGGTGAGAACGGTGTCTATCTACATGGTATG

CCAAATGCCTTTGCACCAAAAGAACAGCGCAGTGTGCCTGCATTTTTCTGGACGGATAAGCAAACTGGCATCACGCCAAT

GGCAACCGATACCGTCCTGACCCATGACGCGATCACGCCGACATTATTAAAGCTGTTTGATGTCACCGCGGACAAAGTCA

AAGACCGCACCGCATTCATCCGCTGA

>MN367316.1 Escherichia coli strain AF19 phosphoethanolamine--lipid A transferase MCR-1.1 (mcr-1) gene, mcr-1.1 allele, complete cds

ATGATGCAGCATACTTCTGTGTGGTACCGACGCTCGGTCAGTCCGTTTGTTCTTGTGGCGAGTGTTGCCGTTTTCTTGAC

CGCGACCGCCAATCTTACCTTTTTTGATAAAATCAGCCAAACCTATCCCATCGCGGACAATCTCGGCTTTGTGCTGACGA

TCGCTGTCGTGCTCTTTGGCGCGATGCTACTGATCACCACGCTGTTATCATCGTATCGCTATGTGCTAAAGCCTGTGTTG

ATTTTGCTATTAATCATGGGCGCGGTGACCAGTTATTTTACTGACACTTATGGCACGGTCTATGATACGACCATGCTCCA

AAATGCCCTACAGACCGACCAAGCCGAGACCAAGGATCTATTAAACGCAGCGTTTATCATGCGTATCATTGGTTTGGGTG

TGCTACCAAGTTTGCTTGTGGCTTTTGTTAAGGTGGATTATCCGACTTGGGGCAAGGGTTTGATGCGCCGATTGGGCTTG

ATCGTGGCAAGTCTTGCGCTGATTTTACTGCCTGTGGTGGCGTTCAGCAGTCATTATGCCAGTTTCTTTCGCGTGCATAA

GCCGCTGCGTAGCTATGTCAATCCGATCATGCCAATCTACTCGGTGGGTAAGCTTGCCAGTATTGAGTATAAAAAAGCCA

GTGCGCCAAAAGATACCATTTATCACGCCAAAGACGCGGTACAAGCAACCAAGCCTGATATGCGTAAGCCACGCCTAGTG

GTGTTCGTCGTCGGTGAGACGGCACGCGCCGATCATGTCAGCTTCAATGGCTATGAGCGCGATACTTTCCCACAGCTTGC

CAAGATCGATGGCGTGACCAATTTTAGCAATGTCACATCGTGCGGCACATCGACGGCGTATTCTGTGCCGTGTATGTTCA

GCTATCTGGGCGCGGATGAGTATGATGTCGATACCGCCAAATACCAAGAAAATGTGCTGGATACGCTGGATCGCTTGGGC

GTAAGTATCTTGTGGCGTGATAATAATTCGGACTCAAAAGGCGTGATGGATAAGCTGCCAAAAGCGCAATTTGCCGATTA

TAAATCCGCGACCAACAACGCCATCTGCAACACCAATCCTTATAACGAATGCCGCGATGTCGGTATGCTCGTTGGCTTAG

ATGACTTTGTCGCTGCCAATAACGGCAAAGATATGCTGATCATGCTGCACCAAATGGGCAATCACGGGCCTGCGTATTTT

AAGCGATATGATGAAAAGTTTGCCAAATTCACGCCAGTGTGTGAAGGTAATGAGCTTGCCAAGTGCGAACATCAGTCCTT

GATCAATGCTTATGACAATGCCTTGCTTGCCACCGATGATTTCATCGCTCAAAGTATCCAGTGGCTGCAGACGCACAGCA

ATGCCTATGATGTCTCAATGCTGTATGTCAGCGATCATGGCGAAAGTCTGGGTGAGAACGGTGTCTATCTACATGGTATG

CCAAATGCCTTTGCACCAAAAGAACAGCGCAGTGTGCCTGCATTTTTCTGGACGGATAAGCAAACTGGCATCACGCCAAT

GGCAACCGATACCGTCCTGACCCATGACGCGATCACGCCGACATTATTAAAGCTGTTTGATGTCACCGCGGACAAAGTCA

AAGACCGCACCGCATTCATCCGCTGA

>MN367315.1 Escherichia coli strain AF17 phosphoethanolamine--lipid A transferase MCR-1.1 (mcr-1) gene, mcr-1.1 allele, complete cds

ATGATGCAGCATACTTCTGTGTGGTACCGACGCTCGGTCAGTCCGTTTGTTCTTGTGGCGAGTGTTGCCGTTTTCTTGAC

CGCGACCGCCAATCTTACCTTTTTTGATAAAATCAGCCAAACCTATCCCATCGCGGACAATCTCGGCTTTGTGCTGACGA
TCGCTGTCGTGCTCTTTGGCGCGATGCTACTGATCACCACGCTGTTATCATCGTATCGCTATGTGCTAAAGCCTGTGTTG
ATTTTGCTATTAATCATGGGCGCGGTGACCAGTTATTTTACTGACACTTATGGCACGGTCTATGATACGACCATGCTCCA
AAATGCCCTACAGACCGACCAAGCCGAGACCAAGGATCTATTAAACGCAGCGTTTATCATGCGTATCATTGGTTTGGGTG
TGCTACCAAGTTTGCTTGTGGCTTTTGTTAAGGTGGATTATCCGACTTGGGGCAAGGGTTTGATGCGCCGATTGGGCTTG
ATCGTGGCAAGTCTTGCGCTGATTTTACTGCCTGTGGTGGCGTTCAGCAGTCATTATGCCAGTTTCTTTCGCGTGCATAA
GCCGCTGCGTAGCTATGTCAATCCGATCATGCCAATCTACTCGGTGGGTAAGCTTGCCAGTATTGAGTATAAAAAAGCCA
GTGCGCCAAAAGATACCATTTATCACGCCAAAGACGCGGTACAAGCAACCAAGCCTGATATGCGTAAGCCACGCCTAGTG
GTGTTCGTCGTCGGTGAGACGGCACGCGCCGATCATGTCAGCTTCAATGGCTATGAGCGCGATACTTTCCCACAGCTTGC
CAAGATCGATGGCGTGACCAATTTTAGCAATGTCACATCGTGCGGCACATCGACGGCGTATTCTGTGCCGTGTATGTTCA
GCTATCTGGGCGCGGATGAGTATGATGTCGATACCGCCAAATACCAAGAAAATGTGCTGGATACGCTGGATCGCTTGGGC
GTAAGTATCTTGTGGCGTGATAATAATTCGGACTCAAAAGGCGTGATGGATAAGCTGCCAAAAGCGCAATTTGCCGATTA
TAAATCCGCGACCAACAACGCCATCTGCAACACCAATCCTTATAACGAATGCCGCGATGTCGGTATGCTCGTTGGCTTAG
ATGACTTTGTCGCTGCCAATAACGGCAAAGATATGCTGATCATGCTGCACCAAATGGGCAATCACGGGCCTGCGTATTTT
AAGCGATATGATGAAAAGTTTGCCAAATTCACGCCAGTGTGTGAAGGTAATGAGCTTGCCAAGTGCGAACATCAGTCCTT
GATCAATGCTTATGACAATGCCTTGCTTGCCACCGATGATTTCATCGCTCAAAGTATCCAGTGGCTGCAGACGCACAGCA
ATGCCTATGATGTCTCAATGCTGTATGTCAGCGATCATGGCGAAAGTCTGGGTGAGAACGGTGTCTATCTACATGGTATG
CCAAATGCCTTTGCACCAAAAGAACAGCGCAGTGTGCCTGCATTTTTCTGGACGGATAAGCAAACTGGCATCACGCCAAT
GGCAACCGATACCGTCCTGACCCATGACGCGATCACGCCGACATTATTAAAGCTGTTTGATGTCACCGCGGACAAAGTCA

AAGACCGCACCGCATTCATCCGCTGA

>MN367313.1 Escherichia coli strain AF15 phosphoethanolamine--lipid A transferase MCR-1.1 (mcr-1) gene, mcr-1.1 allele, complete cds

ATGATGCAGCATACTTCTGTGTGGTACCGACGCTCGGTCAGTCCGTTTGTTCTTGTGGCGAGTGTTGCCGTTTTCTTGAC

CGCGACCGCCAATCTTACCTTTTTTGATAAAATCAGCCAAACCTATCCCATCGCGGACAATCTCGGCTTTGTGCTGACGA

TCGCTGTCGTGCTCTTTGGCGCGATGCTACTGATCACCACGCTGTTATCATCGTATCGCTATGTGCTAAAGCCTGTGTTG

ATTTTGCTATTAATCATGGGCGCGGTGACCAGTTATTTTACTGACACTTATGGCACGGTCTATGATACGACCATGCTCCA

AAATGCCCTACAGACCGACCAAGCCGAGACCAAGGATCTATTAAACGCAGCGTTTATCATGCGTATCATTGGTTTGGGTG

TGCTACCAAGTTTGCTTGTGGCTTTTGTTAAGGTGGATTATCCGACTTGGGGCAAGGGTTTGATGCGCCGATTGGGCTTG

ATCGTGGCAAGTCTTGCGCTGATTTTACTGCCTGTGGTGGCGTTCAGCAGTCATTATGCCAGTTTCTTTCGCGTGCATAA

GCCGCTGCGTAGCTATGTCAATCCGATCATGCCAATCTACTCGGTGGGTAAGCTTGCCAGTATTGAGTATAAAAAAGCCA

GTGCGCCAAAAGATACCATTTATCACGCCAAAGACGCGGTACAAGCAACCAAGCCTGATATGCGTAAGCCACGCCTAGTG

GTGTTCGTCGTCGGTGAGACGGCACGCGCCGATCATGTCAGCTTCAATGGCTATGAGCGCGATACTTTCCCACAGCTTGC

CAAGATCGATGGCGTGACCAATTTTAGCAATGTCACATCGTGCGGCACATCGACGGCGTATTCTGTGCCGTGTATGTTCA

GCTATCTGGGCGCGGATGAGTATGATGTCGATACCGCCAAATACCAAGAAAATGTGCTGGATACGCTGGATCGCTTGGGC

GTAAGTATCTTGTGGCGTGATAATAATTCGGACTCAAAAGGCGTGATGGATAAGCTGCCAAAAGCGCAATTTGCCGATTA

TAAATCCGCGACCAACAACGCCATCTGCAACACCAATCCTTATAACGAATGCCGCGATGTCGGTATGCTCGTTGGCTTAG

ATGACTTTGTCGCTGCCAATAACGGCAAAGATATGCTGATCATGCTGCACCAAATGGGCAATCACGGGCCTGCGTATTTT

AAGCGATATGATGAAAAGTTTGCCAAATTCACGCCAGTGTGTGAAGGTAATGAGCTTGCCAAGTGCGAACATCAGTCCTT

GATCAATGCTTATGACAATGCCTTGCTTGCCACCGATGATTTCATCGCTCAAAGTATCCAGTGGCTGCAGACGCACAGCA

ATGCCTATGATGTCTCAATGCTGTATGTCAGCGATCATGGCGAAAGTCTGGGTGAGAACGGTGTCTATCTACATGGTATG

CCAAATGCCTTTGCACCAAAAGAACAGCGCAGTGTGCCTGCATTTTTCTGGACGGATAAGCAAACTGGCATCACGCCAAT

GGCAACCGATACCGTCCTGACCCATGACGCGATCACGCCGACATTATTAAAGCTGTTTGATGTCACCGCGGACAAAGTCA

AAGACCGCACCGCATTCATCCGCTGA

>MH484519.1 Escherichia coli strain P0001 plasmid pP0001 insertion sequence ISApl1 transposase (tnpA) gene, complete cds; and phosphoethanolamine--lipid A transferase MCR-1.1 (mcr-1) and hypothetical protein genes, complete cds

CTTAATCACGGATTCACTCGTGAAATGGCAGAAGATGCTCTTGGAAAACAATTTGTCCAGGCCAGAGAAGAACATCTGGA

ATTGCAGCAACAGCGTGACAATTCACAGGACATGCACTAATTAAGAAAAAAGCCCGGTTTTCGGGCTTTTTAAGAGCTGA

ATTTACAATCCAAGTGCAACAAAAAAAGAAGTACTCATCAACTAGAATATAATTTTGTTTCCACACAAAAACCAATTCCA

AATGATGAGTACTTCCTACCGACATCTTACAATAAACGAGCGAGAAAAGATAATGATTTTACTCGCACAGGGCAAAAAAC

AAGCAGAAATTGCCAAAGCACTGGGACGTAGCTCCAGCACCATTTCTCGCGAGCTGAAACGACACGCTCTAGAAAGCTAC

AGTGCAACGAACGCACAAAACAGCTATTTGAAGCATCGTCAAAATAGCAAAGCACAGCGCAAATTAGAGCAGCCTGAATA

TTTCAATTTGGTGCAAGAAAAGTTTCTGACAGAAAACTGGTCGCCCGAACAAATCAGCGCACGATTAAAATTGGAAAAAT

CTGAATTATCCATTAGTTATTCAACCATTTATCGTGGTATTTATTCAGGGTTGTTTGATATAGGCGAACGCAAAGCCAGT

CGCAAACTGCGCCACAAAGGCAAAACACGGCATACAAAAAATCATCATGAAAAACGTGGCAAAATTCAGATATCCAACC
A

TTTGAACGACCGTCCCATTTCGGCGCAAAATCGCAGTCGCTTTGGACATTGGGAAGCCGATACCGTACTGGGTAAAGCGG

GTGGAGCTTGTTTGCTGACGCTGACGGAACGCAAAAGTCGTTTTGAGTTGGTGAAGAAAATTCCTGCCAAAAAAGCCGAA

GCAGTCCAAAAAGCCATGATTGAATTGCTGGATTCACATATATTGCGGTCAATTACGCCAGACCGTGGTAAAGAATTTGC

CCAACATCGTTTGGTAACAGAAGCACTGGGTGTAGAATTTTACTTCCCCGAGCCGCATCAACCGTGGACACGGGGAACGA

ATGAAAATACAAATGGGTTACTTCGTGAATACTTTCCGAAGCACCAAGACATCAATCAGTGGAGCGAAGTTGATATTCAA

CAGGTGATCAATAAACTGAATTTACGACCACGTAAATGTTTAGGTTGGAAAACACCTTATGAAGTTTACTTCAAAAAATC

GTTGCACTTGGTTTGACAATTCAAGATACAAATTATAAATACTCTCAAGTGTATATTCAGTATGGGATTGCGCAATGATT

GCCTAATAAAATTTCTGAAATATTTCTGTATCGCATAATTTTTTATATCAGATAAATTGTACTGGATTTCTTAAAAAATT

GCAGTATAATTGCCGCAATTATCCCACCGTTTATTTTTTGAGTAGTTTCTCATGATGCAGCATACTTCTGTGTGGTACCG

ACGCTCGGTCAGTCCGTTTGTTCTTGTGGCGAGTGTTGCCGTTTTCTTGACCGCGACCGCCAATCTTACCTTTTTGATA

AAATCAGCCAAACCTATCCCATCGCGGACAATCTCGGCTTTGTGCTGACGATCGCTGTCGTGCTCTTTGGCGCGATGCTA

CTGATCACCACGCTGTTATCATCGTATCGCTATGTGCTAAAGCCTGTGTTGATTTTGCTATTAATCATGGGCGCGGTGAC

CAGTTATTTTACTGACACTTATGGCACGGTCTATGATACGACCATGCTCCAAAATGCCCTACAGACCGACCAAGCCGAGA

CCAAGGATCTATTAAACGCAGCGTTTATCATGCGTATCATTGGTTTGGGTGTGCTACCAAGTTTGCTTGTGGCTTTTGTT

AAGGTGGATTATCCGACTTGGGGCAAGGGTTTGATGCGCCGATTGGGCTTGATCGTGGCAAGTCTTGCGCTGATTTTACT

GCCTGTGGTGGCGTTCAGCAGTCATTATGCCAGTTTCTTTCGCGTGCATAAGCCGCTGCGTAGCTATGTCAATCCGATCA

TGCCAATCTACTCGGTGGGTAAGCTTGCCAGTATTGAGTATAAAAAAGCCAGTGCGCCAAAAGATACCATTTATCACGCC

AAAGACGCGGTACAAGCAACCAAGCCTGATATGCGTAAGCCACGCCTAGTGGTGTTCGTCGTCGGTGAGACGGCACGCGC

CGATCATGTCAGCTTCAATGGCTATGAGCGCGATACTTTCCCACAGCTTGCCAAGATCGATGGCGTGACCAATTTTAGCA

ATGTCACATCGTGCGGCACATCGACGGCGTATTCTGTGCCGTGTATGTTCAGCTATCTGGGCGCGGATGAGTATGATGTC

GATACCGCCAAATACCAAGAAAATGTGCTGGATACGCTGGATCGCTTGGGCGTAAGTATCTTGTGGCGTGATAATAATTC

GGACTCAAAAGGCGTGATGGATAAGCTGCCAAAAGCGCAATTTGCCGATTATAAATCCGCGACCAACAACGCCATCTGCA

ACACCAATCCTTATAACGAATGCCGCGATGTCGGTATGCTCGTTGGCTTAGATGACTTTGTCGCTGCCAATAACGGCAAA

GATATGCTGATCATGCTGCACCAAATGGGCAATCACGGGCCTGCGTATTTTAAGCGATATGATGAAAAGTTTGCCAAATT
CACGCCAGTGTGTGAAGGTAATGAGCTTGCCAAGTGCGAACATCAGTCCTTGATCAATGCTTATGACAATGCCTTGCTTG
CCACCGATGATTTCATCGCTCAAAGTATCCAGTGGCTGCAGACGCACAGCAATGCCTATGATGTCTCAATGCTGTATGTC
AGCGATCATGGCGAAAGTCTGGGTGAGAACGGTGTCTATCTACATGGTATGCCAAATGCCTTTGCACCAAAAGAACAGCG
CAGTGTGCCTGCATTTTTCTGGACGGATAAGCAAACTGGCATCACGCCAATGGCAACCGATACCGTCCTGACCCATGACG
CGATCACGCCGACATTATTAAAGCTGTTTGATGTCACCGCGGACAAAGTCAAAGACCGCACCGCATTCATCCGCTGATTT
CTCCCTGTATTTTTTCCAAACCCACCGCACACTCCATTCGTATTATGGGCGGTGGGGTGGGGTTTGTTATGCCGTATTTA
TCAAATAAACGCCTACTTGCTGAGATGAGTATCGCTCTTGTCATGGCGATCGTTGCCACGCTGACCCTTGAGCACAGTCA
GATTGATCTGATGGTCGCTGATTGGTTTTATCTGGGTATGGGGCATTGGATGGTTGCCAAGCAAGCTTTTTTGCCAGATT
TGCTACTGTATTCTGGACTAAAAAAGCTGCTGATGGCGATGCTGATCTACTTGCTGGTTGCGACCATTTGCCGTGCTTAT
CATGAGAAAAAGGGCAATGCTATCACTGCCAAGTGGCTTGTCCCAGTGACAAAATTTCGCGTGCGTGAGCTTGCGTATCT
GGTGCTGACTTTGATCCTAGTGCCGACAGTTGTCGCGTCATTGAAGGCATATACTCATGTGGTCTGCCCTGTGCATTTGA
CGATTTTTGATGGTACGCTGCCGTATTTGCCGATGCTTGATAGTATGCGTAACACCATTCCTGATAAGTGCTTTCCTGCG
GCGCATGCCAGTAGCGGATTTGCGCTGTTTGCCTTTGCGTTTGCGCCAAGTTTGCGCCGCCGTCGTGGTGCGATCATCAT
CGTGGTGATGGCATTGGGCTGGGCGATGGGCTGCTATAAGATGATTATTGGCGATCATTTTTTGAGCCATACGGTGGTGT
CGATGATGCTTGCGTGGGCGATGTCGGCAGGGCTTGCGTGGGTGTTTTTTAAGAAGGGTGAACAAGTTCAAGGAAAAAAA
ATCAACCCCAAACAGAAATAACAGCTACAAAAATGGACAGTAATAATGCAATAAAAGTAATGTTGAGCCATATGTTGGAC
GAGA

>MK405590.1 Escherichia coli strain CR4 plasmid phosphoethanolamine--lipid A transferase MCR-1.1 (mcr-1) gene, mcr-1.1 allele, complete cds

ATGATGCAGCATACTTCTGTGTGGTACCGACGCTCGGTCAGTCCGTTTGTTCTTGTGGCGAGTGTTGCCGTTTTCTTGAC
CGCGACCGCCAATCTTACCTTTTTTGATAAAATCAGCCAAACCTATCCCATCGCGGACAATCTCGGCTTTGTGCTGACGA
TCGCTGTCGTGCTCTTTGGCGCGATGCTACTGATCACCACGCTGTTATCATCGTATCGCTATGTGCTAAAGCCTGTGTTG
ATTTTGCTATTAATCATGGGCGCGGTGACCAGTTATTTTACTGACACTTATGGCACGGTCTATGATACGACCATGCTCCA
AAATGCCCTACAGACCGACCAAGCCGAGACCAAGGATCTATTAAACGCAGCGTTTATCATGCGTATCATTGGTTTGGGTG
TGCTACCAAGTTTGCTTGTGGCTTTTGTTAAGGTGGATTATCCGACTTGGGGCAAGGGTTTGATGCGCCGATTGGGCTTG
ATCGTGGCAAGTCTTGCGCTGATTTTACTGCCTGTGGTGGCGTTCAGCAGTCATTATGCCAGTTTCTTTCGCGTGCATAA
GCCGCTGCGTAGCTATGTCAATCCGATCATGCCAATCTACTCGGTGGGTAAGCTTGCCAGTATTGAGTATAAAAAAGCCA
GTGCGCCAAAAGATACCATTTATCACGCCAAAGACGCGGTACAAGCAACCAAGCCTGATATGCGTAAGCCACGCCTAGTG
GTGTTCGTCGTCGGTGAGACGGCACGCGCCGATCATGTCAGCTTCAATGGCTATGAGCGCGATACTTTCCCACAGCTTGC
CAAGATCGATGGCGTGACCAATTTTAGCAATGTCACATCGTGCGGCACATCGACGGCGTATTCTGTGCCGTGTATGTTCA
GCTATCTGGGCGCGGATGAGTATGATGTCGATACCGCCAAATACCAAGAAAATGTGCTGGATACGCTGGATCGCTTGGGC
GTAAGTATCTTGTGGCGTGATAATAATTCGGACTCAAAAGGCGTGATGGATAAGCTGCCAAAAGCGCAATTTGCCGATTA
TAAATCCGCGACCAACAACGCCATCTGCAACACCAATCCTTATAACGAATGCCGCGATGTCGGTATGCTCGTTGGCTTAG
ATGACTTTGTCGCTGCCAATAACGGCAAAGATATGCTGATCATGCTGCACCAAATGGGCAATCACGGGCCTGCGTATTTT
AAGCGATATGATGAAAAGTTTGCCAAATTCACGCCAGTGTGTGAAGGTAATGAGCTTGCCAAGTGCGAACATCAGTCCTT
GATCAATGCTTATGACAATGCCTTGCTTGCCACCGATGATTTCATCGCTCAAAGTATCCAGTGGCTGCAGACGCACAGCA
ATGCCTATGATGTCTCAATGCTGTATGTCAGCGATCATGGCGAAAGTCTGGGTGAGAACGGTGTCTATCTACATGGTATG
CCAAATGCCTTTGCACCAAAAGAACAGCGCAGTGTGCCTGCATTTTTCTGGACGGATAAGCAAACTGGCATCACGCCAAT

GGCAACCGATACCGTCCTGACCCATGACGCGATCACGCCGACATTATTAAAGCTGTTTGATGTCACCGCGGACAAAGTCA

AAGACCGCACCGCATTCATCCGCTGA

>LC427672.1 Escherichia coli plasmid pIVRIKOL-2 mcr-1 gene for phosphoethanolamine transferase MCR1, complete cds

ATGATGCAGCATACTTCTGTGTGGTACCGACGCTCGGTCAGTCCGTTTGTTCTTGTGGCGAGTGTTGCCGTTTTCTTGAC

CGCGACCGCCAATCTTACCTTTTTTGATAAAATCAGCCAAACCTATCCCATCGCGGACAATCTCGGCTTTGTGCTGACGA

TCGCTGTCGTGCTCTTTGGCGCGATGCTACTGATCACCACGCTGTTATCATCGTATCGCTATGTGCTAAAGCCTGTGTTG

ATTTTGCTATTAATCATGGGCGCGGTGACCAGTTATTTTACTGACACTTATGGCACGGTCTATGATACGACCATGCTCCA

AAATGCCCTACAGACCGACCAAGCCGAGACCAAGGATCTATTAAACGCAGCGTTTATCATGCGTATCATTGGTTTGGGTG

TGCTACCAAGTTTGCTTGTGGCTTTTGTTAAGGTGGATTATCCGACTTGGGGCAAGGGTTTGATGCGCCGATTGGGCTTG

ATCGTGGCAAGTCTTGCGCTGATTTTACTGCCTGTGGTGGCGTTCAGCAGTCATTATGCCAGTTTCTTTCGCGTGCATAA

GCCGCTGCGTAGCTATGTCAATCCGATCATGCCAATCTACTCGGTGGGTAAGCTTGCCAGTATTGAGTATAAAAAAGCCA

GTGCGCCAAAAGATACCATTTATCACGCCAAAGACGCGGTACAAGCAACCAAGCCTGATATGCGTAAGCCACGCCTAGTG

GTGTTCGTCGTCGGTGAGACGGCACGCGCCGATCATGTCAGCTTCAATGGCTATGAGCGCGATACTTTCCCACAGCTTGC

CAAGATCGATGGCGTGACCAATTTTAGCAATGTCACATCGTGCGGCACATCGACGGCGTATTCTGTGCCGTGTATGTTCA

GCTATCTGGGCGCGGATGAGTATGATGTCGATACCGCCAAATACCAAGAAAATGTGCTGGATACGCTGGATCGCTTGGGC

GTAAGTATCTTGTGGCGTGATAATAATTCGGACTCAAAAGGCGTGATGGATAAGCTGCCAAAAGCGCAATTTGCCGATTA

TAAATCCGCGACCAACAACGCCATCTGCAACACCAATCCTTATAACGAATGCCGCGATGTCGGTATGCTCGTTGGCTTAG

ATGACTTTGTCGCTGCCAATAACGGCAAAGATATGCTGATCATGCTGCACCAAATGGGCAATCACGGGCCTGCGTATTTT

AAGCGATATGATGAAAAGTTTGCCAAATTCACGCCAGTGTGTGAAGGTAATGAGCTTGCCAAGTGCGAACATCAGTCCTT

GATCAATGCTTATGACAATGCCTTGCTTGCCACCGATGATTTCATCGCTCAAAGTATCCAGTGGCTGCAGACGCACAGCA

ATGCCTATGATGTCTCAATGCTGTATGTCAGCGATCATGGCGAAAGTCTGGGTGAGAACGGTGTCTATCTACATGGTATG

CCAAATGCCTTTGCACCAAAAGAACAGCGCAGTGTGCCTGCATTTTTCTGGACGGATAAGCAAACTGGCATCACGCCAAT

GGCAACCGATACCGTCCTGACCCATGACGCGATCACGCCGACATTATTAAAGCTGTTTGATGTCACCGCGGACAAAGTCA

AAGACCGCACCGCATTCATCCGCTGA

>MH602243.1 Escherichia coli strain TA007 isolate R54 plasmid phosphoethanolamine transferase MCR-1 (mcr-1) gene, complete cds

ATGATGCAGCATACTTCTGTGTGGTACCGACGCTCGGTCAGTCCGTTTGTTCTTGTGGCGAGTGTTGCCGTTTTCTTGAC

CGCGACCGCCAATCTTACCTTTTTTGATAAAATCAGCCAAACCTATCCCATCGCGGACAATCTCGGCTTTGTGCTGACGA

TCGCTGTCGTGCTCTTTGGCGCGATGCTACTGATCACCACGCTGTTATCATCGTATCGCTATGTGCTAAAGCCTGTGTTG

ATTTTGCTATTAATCATGGGCGCGGTGACCAGTTATTTTACTGACACTTATGGCACGGTCTATGATACGACCATGCTCCA

AAATGCCCTACAGACCGACCAAGCCGAGACCAAGGATCTATTAAACGCAGCGTTTATCATGCGTATCATTGGTTTGGGTG

TGCTACCAAGTTTGCTTGTGGCTTTTGTTAAGGTGGATTATCCGACTTGGGGCAAGGGTTTGATGCGCCGATTGGGCTTG

ATCGTGGCAAGTCTTGCGCTGATTTTACTGCCTGTGGTGGCGTTCAGCAGTCATTATGCCAGTTTCTTTCGCGTGCATAA

GCCGCTGCGTAGCTATGTCAATCCGATCATGCCAATCTACTCGGTGGGTAAGCTTGCCAGTATTGAGTATAAAAAAGCCA

GTGCGCCAAAAGATACCATTTATCACGCCAAAGACGCGGTACAAGCAACCAAGCCTGATATGCGTAAGCCACGCCTAGTG

GTGTTCGTCGTCGGTGAGACGGCACGCGCCGATCATGTCAGCTTCAATGGCTATGAGCGCGATACTTTCCCACAGCTTGC

CAAGATCGATGGCGTGACCAATTTTAGCAATGTCACATCGTGCGGCACATCGACGGCGTATTCTGTGCCGTGTATGTTCA

GCTATCTGGGCGCGGATGAGTATGATGTCGATACCGCCAAATACCAAGAAAATGTGCTGGATACGCTGGATCGCTTGGGC

GTAAGTATCTTGTGGCGTGATAATAATTCGGACTCAAAAGGCGTGATGGATAAGCTGCCAAAAGCGCAATTTGCCGATTA

TAAATCCGCGACCAACAACGCCATCTGCAACACCAATCCTTATAACGAATGCCGCGATGTCGGTATGCTCGTTGGCTTAG

ATGACTTTGTCGCTGCCAATAACGGCAAAGATATGCTGATCATGCTGCACCAAATGGGCAATCACGGGCCTGCGTATTTT

AAGCGATATGATGAAAAGTTTGCCAAATTCACGCCAGTGTGTGAAGGTAATGAGCTTGCCAAGTGCGAACATCAGTCCTT

GATCAATGCTTATGACAATGCCTTGCTTGCCACCGATGATTTCATCGCTCAAAGTATCCAGTGGCTGCAGACGCACAGCA

ATGCCTATGATGTCTCAATGCTGTATGTCAGCGATCATGGCGAAAGTCTGGGTGAGAACGGTGTCTATCTACATGGTATG

CCAAATGCCTTTGCACCAAAAGAACAGCGCAGTGTGCCTGCATTTTTCTGGACGGATAAGCAAACTGGCATCACGCCAAT

GGCAACCGATACCGTCCTGACCCATGACGCGATCACGCCGACATTATTAAAGCTGTTTGATGTCACCGCGGACAAAGTCA

AAGACCGCACCGCATTCATCCGCTGA

>MH602242.1 Escherichia coli strain TA007 isolate R53 plasmid phosphoethanolamine transferase MCR-1 (mcr-1) gene, complete cds

ATCCCACCGTTTATTTTTGAGTAGTTTCTCATGATGCAGCATACTTCTGTGTGGTACCGACGCTCGGTCAGTCCGTTTG

TTCTTGTGGCGAGTGTTGCCGTTTTCTTGACCGCGACCGCCAATCTTACCTTTTTTGATAAAATCAGCCAAACCTATCCC

ATCGCGGACAATCTCGGCTTTGTGCTGACGATCGCTGTCGTGCTCTTTGGCGCGATGCTACTGATCACCACGCTGTTATC

ATCGTATCGCTATGTGCTAAAGCCTGTGTTGATTTTGCTATTAATCATGGGCGCGGTGACCAGTTATTTTACTGACACTT

ATGGCACGGTCTATGATACGACCATGCTCCAAAATGCCCTACAGACCGACCAAGCCGAGACCAAGGATCTATTAAACGCA

GCGTTTATCATGCGTATCATTGGTTTGGGTGTGCTACCAAGTTTGCTTGTGGCTTTTGTTAAGGTGGATTATCCGACTTG

GGGCAAGGGTTTGATGCGCCGATTGGGCTTGATCGTGGCAAGTCTTGCGCTGATTTTACTGCCTGTGGTGGCGTTCAGCA

GTCATTATGCCAGTTTCTTTCGCGTGCATAAGCCGCTGCGTAGCTATGTCAATCCGATCATGCCAATCTACTCGGTGGGT

AAGCTTGCCAGTATTGAGTATAAAAAAGCCAGTGCGCCAAAAGATACCATTTATCACGCCAAAGACGCGGTACAAGCAAC

CAAGCCTGATATGCGTAAGCCACGCCTAGTGGTGTTCGTCGTCGGTGAGACGGCACGCGCCGATCATGTCAGCTTCAATG

GCTATGAGCGCGATACTTTCCCACAGCTTGCCAAGATCGATGGCGTGACCAATTTTAGCAATGTCACATCGTGCGGCACA

TCGACGGCGTATTCTGTGCCGTGTATGTTCAGCTATCTGGGCGCGGATGAGTATGATGTCGATACCGCCAAATACCAAGA

AAATGTGCTGGATACGCTGGATCGCTTGGGCGTAAGTATCTTGTGGCGTGATAATAATTCGGACTCAAAAGGCGTGATGG

ATAAGCTGCCAAAAGCGCAATTTGCCGATTATAAATCCGCGACCAACAACGCCATCTGCAACACCAATCCTTATAACGAA

TGCCGCGATGTCGGTATGCTCGTTGGCTTAGATGACTTTGTCGCTGCCAATAACGGCAAAGATATGCTGATCATGCTGCA

CCAAATGGGCAATCACGGGCCTGCGTATTTTAAGCGATATGATGAAAAGTTTGCCAAATTCACGCCAGTGTGTGAAGGTA

ATGAGCTTGCCAAGTGCGAACATCAGTCCTTGATCAATGCTTATGACAATGCCTTGCTTGCCACCGATGATTTCATCGCT

CAAAGTATCCAGTGGCTGCAGACGCACAGCAATGCCTATGATGTCTCAATGCTGTATGTCAGCGATCATGGCGAAAGTCT

GGGTGAGAACGGTGTCTATCTACATGGTATGCCAAATGCCTTTGCACCAAAAGAACAGCGCAGTGTGCCTGCATTTTTCT

GGACGGATAAGCAAACTGGCATCACGCCAATGGCAACCGATACCGTCCTGACCCATGACGCGATCACGCCGACATTATTA

AAGCTGTTTGATGTCACCGCGGACAAAGTCAAAGACCGCACCGCATTCATCCGCTGATTTCTCCCTGTATTTTTCCAAA

CCCACCGCACACTCCATTCGTAT

>MH602241.1 Escherichia coli strain TA007 isolate R51 plasmid phosphoethanolamine transferase MCR-1 (mcr-1) gene, complete cds

TCCCACCGTTTATTTTTGAGTAGTTTCTCATGATGCAGCATACTTCTGTGTGGTACCGACGCTCGGTCAGTCCGTTTGT

TCTTGTGGCGAGTGTTGCCGTTTTCTTGACCGCGACCGCCAATCTTACCTTTTTTGATAAAATCAGCCAAACCTATCCCA

TCGCGGACAATCTCGGCTTTGTGCTGACGATCGCTGTCGTGCTCTTTGGCGCGATGCTACTGATCACCACGCTGTTATCA

TCGTATCGCTATGTGCTAAAGCCTGTGTTGATTTTGCTATTAATCATGGGCGCGGTGACCAGTTATTTTACTGACACTTA

TGGCACGGTCTATGATACGACCATGCTCCAAAATGCCCTACAGACCGACCAAGCCGAGACCAAGGATCTATTAAACGCAG

CGTTTATCATGCGTATCATTGGTTTGGGTGTGCTACCAAGTTTGCTTGTGGCTTTTGTTAAGGTGGATTATCCGACTTGG

GGCAAGGGTTTGATGCGCCGATTGGGCTTGATCGTGGCAAGTCTTGCGCTGATTTTACTGCCTGTGGTGGCGTTCAGCAG

TCATTATGCCAGTTTCTTTCGCGTGCATAAGCCGCTGCGTAGCTATGTCAATCCGATCATGCCAATCTACTCGGTGGGTA

AGCTTGCCAGTATTGAGTATAAAAAAGCCAGTGCGCCAAAAGATACCATTTATCACGCCAAAGACGCGGTACAAGCAACC

AAGCCTGATATGCGTAAGCCACGCCTAGTGGTGTTCGTCGTCGGTGAGACGGCACGCGCCGATCATGTCAGCTTCAATGG

CTATGAGCGCGATACTTTCCCACAGCTTGCCAAGATCGATGGCGTGACCAATTTTAGCAATGTCACATCGTGCGGCACAT

CGACGGCGTATTCTGTGCCGTGTATGTTCAGCTATCTGGGCGCGGATGAGTATGATGTCGATACCGCCAAATACCAAGAA

AATGTGCTGGATACGCTGGATCGCTTGGGCGTAAGTATCTTGTGGCGTGATAATAATTCGGACTCAAAAGGCGTGATGGA

TAAGCTGCCAAAAGCGCAATTTGCCGATTATAAATCCGCGACCAACAACGCCATCTGCAACACCAATCCTTATAACGAAT

GCCGCGATGTCGGTATGCTCGTTGGCTTAGATGACTTTGTCGCTGCCAATAACGGCAAAGATATGCTGATCATGCTGCAC

CAAATGGGCAATCACGGGCCTGCGTATTTTAAGCGATATGATGAAAAGTTTGCCAAATTCACGCCAGTGTGTGAAGGTAA

TGAGCTTGCCAAGTGCGAACATCAGTCCTTGATCAATGCTTATGACAATGCCTTGCTTGCCACCGATGATTTCATCGCTC

AAAGTATCCAGTGGCTGCAGACGCACAGCAATGCCTATGATGTCTCAATGCTGTATGTCAGCGATCATGGCGAAAGTCTG

GGTGAGAACGGTGTCTATCTACATGGTATGCCAAATGCCTTTGCACCAAAAGAACAGCGCAGTGTGCCTGCATTTTTCTG

GACGGATAAGCAAACTGGCATCACGCCAATGGCAACCGATACCGTCCTGACCCATGACGCGATCACGCCGACATTATTAA

AGCTGTTTGATGTCACCGCGGACAAAGTCAAAGACCGCACCGCATTCATCCGCTGATTTCTCCCTGTATTTTTCCAAAC

CCACCGCACACTCCATT

>MH602240.1 Escherichia coli strain TA007 isolate R50 plasmid phosphoethanolamine transferase MCR-1 (mcr-1) gene, complete cds

TATCCCACCGTTTATTTTTTGAGTAGTTTCTCATGATGCAGCATACTTCTGTGTGGTACCGACGCTCGGTCAGTCCGTTT

GTTCTTGTGGCGAGTGTTGCCGTTTTCTTGACCGCGACCGCCAATCTTACCTTTTTTGATAAAATCAGCCAAACCTATCC

CATCGCGGACAATCTCGGCTTTGTGCTGACGATCGCTGTCGTGCTCTTTGGCGCGATGCTACTGATCACCACGCTGTTAT

CATCGTATCGCTATGTGCTAAAGCCTGTGTTGATTTTGCTATTAATCATGGGCGCGGTGACCAGTTATTTTACTGACACT

TATGGCACGGTCTATGATACGACCATGCTCCAAAATGCCCTACAGACCGACCAAGCCGAGACCAAGGATCTATTAAACGC

AGCGTTTATCATGCGTATCATTGGTTTGGGTGTGCTACCAAGTTTGCTTGTGGCTTTTGTTAAGGTGGATTATCCGACTT

GGGGCAAGGGTTTGATGCGCCGATTGGGCTTGATCGTGGCAAGTCTTGCGCTGATTTTACTGCCTGTGGTGGCGTTCAGC

AGTCATTATGCCAGTTTCTTTCGCGTGCATAAGCCGCTGCGTAGCTATGTCAATCCGATCATGCCAATCTACTCGGTGGG

TAAGCTTGCCAGTATTGAGTATAAAAAAGCCAGTGCGCCAAAAGATACCATTTATCACGCCAAAGACGCGGTACAAGCAA

CCAAGCCTGATATGCGTAAGCCACGCCTAGTGGTGTTCGTCGTCGGTGAGACGGCACGCGCCGATCATGTCAGCTTCAAT

GGCTATGAGCGCGATACTTTCCCACAGCTTGCCAAGATCGATGGCGTGACCAATTTTAGCAATGTCACATCGTGCGGCAC

ATCGACGGCGTATTCTGTGCCGTGTATGTTCAGCTATCTGGGCGCGGATGAGTATGATGTCGATACCGCCAAATACCAAG

AAAATGTGCTGGATACGCTGGATCGCTTGGGCGTAAGTATCTTGTGGCGTGATAATAATTCGGACTCAAAAGGCGTGATG

GATAAGCTGCCAAAAGCGCAATTTGCCGATTATAAATCCGCGACCAACAACGCCATCTGCAACACCAATCCTTATAACGA

ATGCCGCGATGTCGGTATGCTCGTTGGCTTAGATGACTTTGTCGCTGCCAATAACGGCAAAGATATGCTGATCATGCTGC

ACCAAATGGGCAATCACGGGCCTGCGTATTTTAAGCGATATGATGAAAAGTTTGCCAAATTCACGCCAGTGTGTGAAGGT

AATGAGCTTGCCAAGTGCGAACATCAGTCCTTGATCAATGCTTATGACAATGCCTTGCTTGCCACCGATGATTTCATCGC

TCAAAGTATCCAGTGGCTGCAGACGCACAGCAATGCCTATGATGTCTCAATGCTGTATGTCAGCGATCATGGCGAAAGTC

TGGGTGAGAACGGTGTCTATCTACATGGTATGCCAAATGCCTTTGCACCAAAAGAACAGCGCAGTGTGCCTGCATTTTTC

TGGACGGATAAGCAAACTGGCATCACGCCAATGGCAACCGATACCGTCCTGACCCATGACGCGATCACGCCGACATTATT

AAAGCTGTTTGATGTCACCGCGGACAAAGTCAAAGACCGCACCGCATTCATCCGCTGATTTCTCCCTGTATTTTTTCCAA

ACCCACCGCACACTCCATTCGTATT

>MH602239.1 Escherichia coli strain TA007 isolate R49 plasmid phosphoethanolamine transferase MCR-1 (mcr-1) gene, complete cds

ATCCCACCGTTTATTTTTGAGTAGTTTCTCATGATGCAGCATACTTCTGTGTGGTACCGACGCTCGGTCAGTCCGTTTG

TTCTTGTGGCGAGTGTTGCCGTTTTCTTGACCGCGACCGCCAATCTTACCTTTTTGATAAAATCAGCCAAACCTATCCC

ATCGCGGACAATCTCGGCTTTGTGCTGACGATCGCTGTCGTGCTCTTTGGCGCGATGCTACTGATCACCACGCTGTTATC

ATCGTATCGCTATGTGCTAAAGCCTGTGTTGATTTTGCTATTAATCATGGGCGCGGTGACCAGTTATTTTACTGACACTT

ATGGCACGGTCTATGATACGACCATGCTCCAAAATGCCCTACAGACCGACCAAGCCGAGACCAAGGATCTATTAAACGCA

GCGTTTATCATGCGTATCATTGGTTTGGGTGTGCTACCAAGTTTGCTTGTGGCTTTTGTTAAGGTGGATTATCCGACTTG

GGGCAAGGGTTTGATGCGCCGATTGGGCTTGATCGTGGCAAGTCTTGCGCTGATTTTACTGCCTGTGGTGGCGTTCAGCA

GTCATTATGCCAGTTTCTTTCGCGTGCATAAGCCGCTGCGTAGCTATGTCAATCCGATCATGCCAATCTACTCGGTGGGT

AAGCTTGCCAGTATTGAGTATAAAAAAGCCAGTGCGCCAAAAGATACCATTTATCACGCCAAAGACGCGGTACAAGCAAC

CAAGCCTGATATGCGTAAGCCACGCCTAGTGGTGTTCGTCGTCGGTGAGACGGCACGCGCCGATCATGTCAGCTTCAATG

GCTATGAGCGCGATACTTTCCCACAGCTTGCCAAGATCGATGGCGTGACCAATTTTAGCAATGTCACATCGTGCGGCACA

TCGACGGCGTATTCTGTGCCGTGTATGTTCAGCTATCTGGGCGCGGATGAGTATGATGTCGATACCGCCAAATACCAAGA

AAATGTGCTGGATACGCTGGATCGCTTGGGCGTAAGTATCTTGTGGCGTGATAATAATTCGGACTCAAAAGGCGTGATGG

ATAAGCTGCCAAAAGCGCAATTTGCCGATTATAAATCCGCGACCAACAACGCCATCTGCAACACCAATCCTTATAACGAA

TGCCGCGATGTCGGTATGCTCGTTGGCTTAGATGACTTTGTCGCTGCCAATAACGGCAAAGATATGCTGATCATGCTGCA

CCAAATGGGCAATCACGGGCCTGCGTATTTTAAGCGATATGATGAAAAGTTTGCCAAATTCACGCCAGTGTGTGAAGGTA

ATGAGCTTGCCAAGTGCGAACATCAGTCCTTGATCAATGCTTATGACAATGCCTTGCTTGCCACCGATGATTTCATCGCT

CAAAGTATCCAGTGGCTGCAGACGCACAGCAATGCCTATGATGTCTCAATGCTGTATGTCAGCGATCATGGCGAAAGTCT

GGGTGAGAACGGTGTCTATCTACATGGTATGCCAAATGCCTTTGCACCAAAAGAACAGCGCAGTGTGCCTGCATTTTTCT

GGACGGATAAGCAAACTGGCATCACGCCAATGGCAACCGATACCGTCCTGACCCATGACGCGATCACGCCGACATTATTA

AAGCTGTTTGATGTCACCGCGGACAAAGTCAAAGACCGCACCGCATTCATCCGCTGATTTCTCCCTGTATTTTTTCCAAA

CCCACCGCACACTCCATTCGTA

>MH602238.1 Escherichia coli strain TA007 isolate R48 plasmid phosphoethanolamine transferase MCR-1 (mcr-1) gene, complete cds

TATCCCACCGTTTATTTTTTGAGTAGTTTCTCATGATGCAGCATACTTCTGTGTGGTACCGACGCTCGGTCAGTCCGTTT

GTTCTTGTGGCGAGTGTTGCCGTTTTCTTGACCGCGACCGCCAATCTTACCTTTTTTGATAAAATCAGCCAAACCTATCC

CATCGCGGACAATCTCGGCTTTGTGCTGACGATCGCTGTCGTGCTCTTTGGCGCGATGCTACTGATCACCACGCTGTTAT

CATCGTATCGCTATGTGCTAAAGCCTGTGTTGATTTTGCTATTAATCATGGGCGCGGTGACCAGTTATTTTACTGACACT

TATGGCACGGTCTATGATACGACCATGCTCCAAAATGCCCTACAGACCGACCAAGCCGAGACCAAGGATCTATTAAACGC

AGCGTTTATCATGCGTATCATTGGTTTGGGTGTGCTACCAAGTTTGCTTGTGGCTTTTGTTAAGGTGGATTATCCGACTT

GGGGCAAGGGTTTGATGCGCCGATTGGGCTTGATCGTGGCAAGTCTTGCGCTGATTTTACTGCCTGTGGTGGCGTTCAGC

AGTCATTATGCCAGTTTCTTTCGCGTGCATAAGCCGCTGCGTAGCTATGTCAATCCGATCATGCCAATCTACTCGGTGGG

TAAGCTTGCCAGTATTGAGTATAAAAAAGCCAGTGCGCCAAAAGATACCATTTATCACGCCAAAGACGCGGTACAAGCAA

CCAAGCCTGATATGCGTAAGCCACGCCTAGTGGTGTTCGTCGTCGGTGAGACGGCACGCGCCGATCATGTCAGCTTCAAT

GGCTATGAGCGCGATACTTTCCCACAGCTTGCCAAGATCGATGGCGTGACCAATTTTAGCAATGTCACATCGTGCGGCAC

ATCGACGGCGTATTCTGTGCCGTGTATGTTCAGCTATCTGGGCGCGGATGAGTATGATGTCGATACCGCCAAATACCAAG

AAAATGTGCTGGATACGCTGGATCGCTTGGGCGTAAGTATCTTGTGGCGTGATAATAATTCGGACTCAAAAGGCGTGATG

GATAAGCTGCCAAAAGCGCAATTTGCCGATTATAAATCCGCGACCAACAACGCCATCTGCAACACCAATCCTTATAACGA

ATGCCGCGATGTCGGTATGCTCGTTGGCTTAGATGACTTTGTCGCTGCCAATAACGGCAAAGATATGCTGATCATGCTGC

ACCAAATGGGCAATCACGGGCCTGCGTATTTTAAGCGATATGATGAAAAGTTTGCCAAATTCACGCCAGTGTGTGAAGGT

AATGAGCTTGCCAAGTGCGAACATCAGTCCTTGATCAATGCTTATGACAATGCCTTGCTTGCCACCGATGATTTCATCGC

TCAAAGTATCCAGTGGCTGCAGACGCACAGCAATGCCTATGATGTCTCAATGCTGTATGTCAGCGATCATGGCGAAAGTC

TGGGTGAGAACGGTGTCTATCTACATGGTATGCCAAATGCCTTTGCACCAAAAGAACAGCGCAGTGTGCCTGCATTTTTC

TGGACGGATAAGCAAACTGGCATCACGCCAATGGCAACCGATACCGTCCTGACCCATGACGCGATCACGCCGACATTATT

AAAGCTGTTTGATGTCACCGCGGACAAAGTCAAAGACCGCACCGCATTCATCCGCTGATTTCTCCCTGTATTTTTTCCAA

ACCCACCGCACACTCCATTCGTATTA

>MH602237.1 Escherichia coli strain TA007 isolate R45 plasmid phosphoethanolamine transferase MCR-1 (mcr-1) gene, complete cds

ATGATGCAGCATACTTCTGTGTGGTACCGACGCTCGGTCAGTCCGTTTGTTCTTGTGGCGAGTGTTGCCGTTTTCTTGAC

CGCGACCGCCAATCTTACCTTTTTTGATAAAATCAGCCAAACCTATCCCATCGCGGACAATCTCGGCTTTGTGCTGACGA

TCGCTGTCGTGCTCTTTGGCGCGATGCTACTGATCACCACGCTGTTATCATCGTATCGCTATGTGCTAAAGCCTGTGTTG

ATTTTGCTATTAATCATGGGCGCGGTGACCAGTTATTTTACTGACACTTATGGCACGGTCTATGATACGACCATGCTCCA

AAATGCCCTACAGACCGACCAAGCCGAGACCAAGGATCTATTAAACGCAGCGTTTATCATGCGTATCATTGGTTTGGGTG

TGCTACCAAGTTTGCTTGTGGCTTTTGTTAAGGTGGATTATCCGACTTGGGGCAAGGGTTTGATGCGCCGATTGGGCTTG

ATCGTGGCAAGTCTTGCGCTGATTTTACTGCCTGTGGTGGCGTTCAGCAGTCATTATGCCAGTTTCTTTCGCGTGCATAA

GCCGCTGCGTAGCTATGTCAATCCGATCATGCCAATCTACTCGGTGGGTAAGCTTGCCAGTATTGAGTATAAAAAAGCCA

GTGCGCCAAAAGATACCATTTATCACGCCAAAGACGCGGTACAAGCAACCAAGCCTGATATGCGTAAGCCACGCCTAGTG

GTGTTCGTCGTCGGTGAGACGGCACGCGCCGATCATGTCAGCTTCAATGGCTATGAGCGCGATACTTTCCCACAGCTTGC

CAAGATCGATGGCGTGACCAATTTTAGCAATGTCACATCGTGCGGCACATCGACGGCGTATTCTGTGCCGTGTATGTTCA

GCTATCTGGGCGCGGATGAGTATGATGTCGATACCGCCAAATACCAAGAAAATGTGCTGGATACGCTGGATCGCTTGGGC

GTAAGTATCTTGTGGCGTGATAATAATTCGGACTCAAAAGGCGTGATGGATAAGCTGCCAAAAGCGCAATTTGCCGATTA

TAAATCCGCGACCAACAACGCCATCTGCAACACCAATCCTTATAACGAATGCCGCGATGTCGGTATGCTCGTTGGCTTAG

ATGACTTTGTCGCTGCCAATAACGGCAAAGATATGCTGATCATGCTGCACCAAATGGGCAATCACGGGCCTGCGTATTTT

AAGCGATATGATGAAAAGTTTGCCAAATTCACGCCAGTGTGTGAAGGTAATGAGCTTGCCAAGTGCGAACATCAGTCCTT

GATCAATGCTTATGACAATGCCTTGCTTGCCACCGATGATTTCATCGCTCAAAGTATCCAGTGGCTGCAGACGCACAGCA

ATGCCTATGATGTCTCAATGCTGTATGTCAGCGATCATGGCGAAAGTCTGGGTGAGAACGGTGTCTATCTACATGGTATG

CCAAATGCCTTTGCACCAAAAGAACAGCGCAGTGTGCCTGCATTTTTCTGGACGGATAAGCAAACTGGCATCACGCCAAT

GGCAACCGATACCGTCCTGACCCATGACGCGATCACGCCGACATTATTAAAGCTGTTTGATGTCACCGCGGACAAAGTCA

AAGACCGCACCGCATTCATCCGCTGA

>MH395740.1 Escherichia coli plasmid pRMCR55 phosphoethanolamine transferase MCR-1 (mcr-1) gene, complete cds

GCCGCATTATCCCACCGTTTATTTTTTGAGTAGTTTCTCATGATGCAGCATACTTCTGTGTGGTACCGACGCTCGGTCAG

TCCGTTTGTTCTTGTGGCGAGTGTTGCCGTTTTCTTGACCGCGACCGCCAATCTTACCTTTTTTGATAAAATCAGCCAAA

CCTATCCCATCGCGGACAATCTCGGCTTTGTGCTGACGATCGCTGTCGTGCTCTTTGGCGCGATGCTACTGATCACCACG

CTGTTATCATCGTATCGCTATGTGCTAAAGCCTGTGTTGATTTTGCTATTAATCATGGGCGCGGTGACCAGTTATTTTAC

TGACACTTATGGCACGGTCTATGATACGACCATGCTCCAAAATGCCCTACAGACCGACCAAGCCGAGACCAAGGATCTAT

TAAACGCAGCGTTTATCATGCGTATCATTGGTTTGGGTGTGCTACCAAGTTTGCTTGTGGCTTTTGTTAAGGTGGATTAT

CCGACTTGGGGCAAGGGTTTGATGCGCCGATTGGGCTTGATCGTGGCAAGTCTTGCGCTGATTTTACTGCCTGTGGTGGC

GTTCAGCAGTCATTATGCCAGTTTCTTTCGCGTGCATAAGCCGCTGCGTAGCTATGTCAATCCGATCATGCCAATCTACT

CGGTGGGTAAGCTTGCCAGTATTGAGTATAAAAAAGCCAGTGCGCCAAAAGATACCATTTATCACGCCAAAGACGCGGTA

CAAGCAACCAAGCCTGATATGCGTAAGCCACGCCTAGTGGTGTTCGTCGTCGGTGAGACGGCACGCGCCGATCATGTCAG

CTTCAATGGCTATGAGCGCGATACTTTCCCACAGCTTGCCAAGATCGATGGCGTGACCAATTTTAGCAATGTCACATCGT

GCGGCACATCGACGGCGTATTCTGTGCCGTGTATGTTCAGCTATCTGGGCGCGGATGAGTATGATGTCGATACCGCCAAA

TACCAAGAAAATGTGCTGGATACGCTGGATCGCTTGGGCGTAAGTATCTTGTGGCGTGATAATAATTCGGACTCAAAAGG

CGTGATGGATAAGCTGCCAAAAGCGCAATTTGCCGATTATAAATCCGCGACCAACAACGCCATCTGCAACACCAATCCTT

ATAACGAATGCCGCGATGTCGGTATGCTCGTTGGCTTAGATGACTTTGTCGCTGCCAATAACGGCAAAGATATGCTGATC

ATGCTGCACCAAATGGGCAATCACGGGCCTGCGTATTTTAAGCGATATGATGAAAAGTTTGCCAAATTCACGCCAGTGTG

TGAAGGTAATGAGCTTGCCAAGTGCGAACATCAGTCCTTGATCAATGCTTATGACAATGCCTTGCTTGCCACCGATGATT

TCATCGCTCAAAGTATCCAGTGGCTGCAGACGCACAGCAATGCCTATGATGTCTCAATGCTGTATGTCAGCGATCATGGC

GAAAGTCTGGGTGAGAACGGTGTCTATCTACATGGTATGCCAAATGCCTTTGCACCAAAAGAACAGCGCAGTGTGCCTGC

ATTTTTCTGGACGGATAAGCAAACTGGCATCACGCCAATGGCAACCGATACCGTCCTGACCCATGACGCGATCACGCCGA

CATTATTAAAGCTGTTTGATGTCACCGCGGACAAAGTCAAAGACCGCACCGCATTCATCCGCTGATTTCTCCCTGTATTT

TTTCCAAACCCACCGCACACTCCATTCGTATTATGG

>KX580711.1 Escherichia coli strain ZJ276 plasmid transposase, phosphoethanolamine transferase (mcr-1), and hypothetical protein genes, complete cds

TTCCAAATGATGAGTACTTCCTACCGACATCTTACAATAAACGAGCGAGAAAAGATAATGATTTTACTCGCACAGGGCAA

AAAACAAGCAGAAATTGCCAAAGCACTGGGACGTAGCTCCAGCACCATTTCTCGCGAGCTGAAACGACACGCTCTAGAAA

GCTACAGTGCAACGAACGCACAAAACAGCTATTTGAAGCATCGTCAAAATAGCAAAGCACAGCGCAAATTAGAGCAGCCT
GAATATTTCAATTTGGTGCAAGAAAAGTTTCTGACAGAAAACTGGTCGCCCGAACAAATCAGCGCACGATTAAAATTGGA
AAAATCTGAATTATCCATTAGTTATTCAACCATTTATCGTGGTATTTATTCAGGGTTGTTTGATATAGGCGAACGCAAAG
CCAGTCGCAAACTGCGCCACAAAGGCAAAACACGGCATACAAAAAATCATCATGAAAAACGTGGCAAAATTCAGATATCC
AACCATTTGAACGACCGTCCCATTTCGGCGCAAAATCGCAGTCGCTTTGGACATTGGGAAGCCGATACCGTACTGGGTAA
AGCGGGTGGAGCTTGTTTGCTGACGCTGACGGAACGCAAAAGTCGTTTTGAGTTGGTGAAGAAAATTCCTGCCAAAAAAG
CCGAAGCAGTCCAAAAAGCCATGATTGAATTGCTGGATTCACATATATTGCGGTCAATTACGCCAGACCGTGGTAAAGAA
TTTGCCCAACATCGTTTGGTAACAGAAGCACTGGGTGTAGAATTTTACTTCCCCGAGCCGCATCAACCGTGGACACGGGG
AACGAATGAAAATACAAATGGGTTACTTCGTGAATACTTTCCGAAGCACCAAGACATCAATCAGTGGAGCGAAGTTGATA
TTCAACAGGTGATCAATAAACTGAATTTACGACCACGTAAATGTTTAGGTTGGAAAACACCTTATGAAGTTTACTTCAAA
AAATCGTTGCACTTGGTTTGACAATTCAAGATACAAATTATAAATACTCTCAAGTGTATATTCAGTATGGGATTGCGCAA
TGATTGCCTAATAAAATTTCTGAAATATTTCTGTATCGCATAATTTTTTATATCAGATAAATTGTACTGGATTTCTTAAA
AAATTGCAGTATAATTGCCGCAATTATCCCACCGTTTATTTTTTGAGTAGTTTCTCATGATGCAGCATACTTCTGTGTGG
TACCGACGCTCGGTCAGTCCGTTTGTTCTTGTGGCGAGTGTTGCCGTTTTCTTGACCGCGACCGCCAATCTTACCTTTTT
TGATAAAATCAGCCAAACCTATCCCATCGCGGACAATCTCGGCTTTGTGCTGACGATCGCTGTCGTGCTCTTTGGCGCGA
TGCTACTGATCACCACGCTGTTATCATCGTATCGCTATGTGCTAAAGCCTGTGTTGATTTTGCTATTAATCATGGGCGCG
GTGACCAGTTATTTTACTGACACTTATGGCACGGTCTATGATACGACCATGCTCCAAAATGCCCTACAGACCGACCAAGC
CGAGACCAAGGATCTATTAAACGCAGCGTTTATCATGCGTATCATTGGTTTGGGTGTGCTACCAAGTTTGCTTGTGGCTT
TTGTTAAGGTGGATTATCCGACTTGGGGCAAGGGTTTGATGCGCCGATTGGGCTTGATCGTGGCAAGTCTTGCGCTGATT

TTACTGCCTGTGGTGGCGTTCAGCAGTCATTATGCCAGTTTCTTTCGCGTGCATAAGCCGCTGCGTAGCTATGTCAATCC

GATCATGCCAATCTACTCGGTGGGTAAGCTTGCCAGTATTGAGTATAAAAAAGCCAGTGCGCCAAAAGATACCATTTATC

ACGCCAAAGACGCGGTACAAGCAACCAAGCCTGATATGCGTAAGCCACGCCTAGTGGTGTTCGTCGTCGGTGAGACGGCA

CGCGCCGATCATGTCAGCTTCAATGGCTATGAGCGCGATACTTTCCCACAGCTTGCCAAGATCGATGGCGTGACCAATTT

TAGCAATGTCACATCGTGCGGCACATCGACGGCGTATTCTGTGCCGTGTATGTTCAGCTATCTGGGCGCGGATGAGTATG

ATGTCGATACCGCCAAATACCAAGAAAATGTGCTGGATACGCTGGATCGCTTGGGCGTAAGTATCTTGTGGCGTGATAAT

AATTCGGACTCAAAAGGCGTGATGGATAAGCTGCCAAAAGCGCAATTTGCCGATTATAAATCCGCGACCAACAACGCCAT

CTGCAACACCAATCCTTATAACGAATGCCGCGATGTCGGTATGCTCGTTGGCTTAGATGACTTTGTCGCTGCCAATAACG

GCAAAGATATGCTGATCATGCTGCACCAAATGGGCAATCACGGGCCTGCGTATTTTAAGCGATATGATGAAAAGTTTGCC

AAATTCACGCCAGTGTGTGAAGGTAATGAGCTTGCCAAGTGCGAACATCAGTCCTTGATCAATGCTTATGACAATGCCTT

GCTTGCCACCGATGATTTCATCGCTCAAAGTATCCAGTGGCTGCAGACGCACAGCAATGCCTATGATGTCTCAATGCTGT

ATGTCAGCGATCATGGCGAAAGTCTGGGTGAGAACGGTGTCTATCTACATGGTATGCCAAATGCCTTTGCACCAAAAGAA

CAGCGCAGTGTGCCTGCATTTTTCTGGACGGATAAGCAAACTGGCATCACGCCAATGGCAACCGATACCGTCCTGACCCA

TGACGCGATCACGCCGACATTATTAAAGCTGTTTGATGTCACCGCGGACAAAGTCAAAGACCGCACCGCATTCATCCGCT

GATTTCTCCCTGTATTTTTTCCAAACCCACCGCACACTCCATTCGTATTATGGGCGGTGGGGTGGGGTTTGTTATGCCGT

ATTTATCAAATAAACGCCTACTTGCTGAGATGAGTATCGCTCTTGTCATGGCGATCGTTGCCACGCTGACCCTTGAGCAC

AGTCAGATTGATCTGATGGTCGCTGATTGGTTTTATCTGGGTATGGGGCATTGGATGGTTGCCAAGCAAGCTTTTTTGCC

AGATTTGCTACTGTATTCTGGACTAAAAAAGCTGCTGATGGCGATGCTGATCTACTTGCTGGTTGCGACCATTTGCCGTG

CTTATCATGAGAAAAAGGGCAATGCTATCACTGCCAAGTGGCTTGTCCCAGTGACAAAATTTCGCGTGCGTGAGCTTGCG

TATCTGGTGCTGACTTTGATCCTAGTGCCGACAGTTGTCGCGTCATTGAAGGCATATACTCATGTGGTCTGCCCTGTGCA

TTTGACGATTTTTGATGGTACGCTGCCGTATTTGCCGATGCTTGATAGTATGCGTAACACCATTCCTGATAAGTGCTTTC

CTGCGGCGCATGCCAGTAGCGGATTTGCGCTGTTTGCCTTTGCGTTTGCGCCAAGTTTGCGCCGCCGTCGTGGTGCGATC

ATCATCGTGGTGATGGCATTGGGCTGGGCGATGGGCTGCTATAAGATGATTATTGGCGATCATTTTTTGAGCCATACGGT

GGTGTCGATGATGCTTGCGTGGGCGATGTCGGCAGGGCTTGCGTGGGTGTTTTTTAAGAAGGGTGAACAAGTTTAATCGG

CTGAATTTACAATCCAAGTGCAACAAAAAAAGAAGTACTCATCAACTAGAATATAATTTTGTTTCCACACAAAAACCACT

TCCAAATGATGAGTAC

>LC193130.1 Escherichia coli tnpA, mcr-1 genes for transposase, phosphoethanolamine transferase, hypothetical protein, complete cds, strain: NCGM-EC88

ATACGCTGAATTTACAATCCAAGTGCAACAAAAAAAGAAGGACTAATCAACTAGAATAGAATTTTGTTTCCACAAAAAAA

CCAATTAAAAAAGATGAGTAAAAACTACCGACATCTTACAATAAACGAGCGAGAAAAGATAATGATTTTACTCGCACAGG

GCAAAAAACAAGCAGAAATTGCCAAAGCACTGGGACGTAGCTCCAGCACCATTTCTCGCGAGCTGAAACGACACGCTCTA

GAAAGCTACAGTGCAACGAACGCACAAAACAGCTATTTGAAGCATCGTCAAAATAGCAAAGCACAGCGCAAATTAGAGC
A

GCCTGAATATTTCAATTTGGTGCAAGAAAAGTTTCTGACAGAAAACTGGTCGCCCGAACAAATCAGCGCACGATTAAAAT

TGGAAAAATCTGAATTATCCATTAGTTATTCAACCATTTATCGTGGTATTTATTCAGGGTTGTTTGATATAGGCGAACGC

AAAGCCAGTCGCAAACTGCGCCACAAAGGCAAAACACGGCATACAAAAAATCATCATGAAAAACGTGGCAAAATTCAGA
T

ATCCAACCATTTGAACGACCGTCCCATTTCGGCGCAAAATCGCAGTCGCTTTGGACATTGGGAAGCCGATACCGTACTGG

GTAAAGCGGGTGGAGCTTGTTTGCTGACGCTGACGGAACGCAAAAGTCGTTTTGAGTTGGTGAAGAAAATTCCTGCCAAA

AAAGCCGAAGCAGTCCAAAAAGCCATGATTGAATTGCTGGATTCACATATATTGCGGTCAATTACGCCAGACCGTGGTAA

AGAATTTGCCCAACATCGTTTGGTAACAGAAGCACTGGGTGTAGAATTTTACTTCCCCGAGCCGCATCAACCGTGGACAC

GGGGAACGAATGAAAATACAAATGGGTTACTTCGTGAATACTTTCCGAAGCACCAAGACATCAATCAGTGGAGCGAAGTT

GATATTCAACAGGTGATCAATAAACTGAATTTACGACCACGTAAATGTTTAGGTTGGAAAACACCTTATGAAGTTTACTT

CAAAAAATCGTTGCACTTGGTTTGACAATTCAAGATACAAATTATAAATACTCTCAAGTGTATATTCAGTATGGGATTGC

GCAATGATTGCCTAATAAAATTTCTGAAATATTTCTGTATCGCATAATTTTTTATATCAGATAAATTGTACTGGATTTCT

TAAAAAATTGCAGTATAATTGCCGCAATTATCCCACCGTTTATTTTTTGAGTAGTTTCTCATGATGCAGCATACTTCTGT

GTGGTACCGACGCTCGGTCAGTCCGTTTGTTCTTGTGGCGAGTGTTGCCGTTTTCTTGACCGCGACCGCCAATCTTACCT

TTTTTGATAAAATCAGCCAAACCTATCCCATCGCGGACAATCTCGGCTTTGTGCTGACGATCGCTGTCGTGCTCTTTGGC

GCGATGCTACTGATCACCACGCTGTTATCATCGTATCGCTATGTGCTAAAGCCTGTGTTGATTTTGCTATTAATCATGGG

CGCGGTGACCAGTTATTTTACTGACACTTATGGCACGGTCTATGATACGACCATGCTCCAAAATGCCCTACAGACCGACC

AAGCCGAGACCAAGGATCTATTAAACGCAGCGTTTATCATGCGTATCATTGGTTTGGGTGTGCTACCAAGTTTGCTTGTG

GCTTTTGTTAAGGTGGATTATCCGACTTGGGGCAAGGGTTTGATGCGCCGATTGGGCTTGATCGTGGCAAGTCTTGCGCT

GATTTTACTGCCTGTGGTGGCGTTCAGCAGTCATTATGCCAGTTTCTTTCGCGTGCATAAGCCGCTGCGTAGCTATGTCA

ATCCGATCATGCCAATCTACTCGGTGGGTAAGCTTGCCAGTATTGAGTATAAAAAAGCCAGTGCGCCAAAAGATACCATT

TATCACGCCAAAGACGCGGTACAAGCAACCAAGCCTGATATGCGTAAGCCACGCCTAGTGGTGTTCGTCGTCGGTGAGAC

GGCACGCGCCGATCATGTCAGCTTCAATGGCTATGAGCGCGATACTTTCCCACAGCTTGCCAAGATCGATGGCGTGACCA

ATTTTAGCAATGTCACATCGTGCGGCACATCGACGGCGTATTCTGTGCCGTGTATGTTCAGCTATCTGGGCGCGGATGAG

TATGATGTCGATACCGCCAAATACCAAGAAAATGTGCTGGATACGCTGGATCGCTTGGGCGTAAGTATCTTGTGGCGTGA

TAATAATTCGGACTCAAAAGGCGTGATGGATAAGCTGCCAAAAGCGCAATTTGCCGATTATAAATCCGCGACCAACAACG
CCATCTGCAACACCAATCCTTATAACGAATGCCGCGATGTCGGTATGCTCGTTGGCTTAGATGACTTTGTCGCTGCCAAT
AACGGCAAAGATATGCTGATCATGCTGCACCAAATGGGCAATCACGGGCCTGCGTATTTTAAGCGATATGATGAAAAGTT
TGCCAAATTCACGCCAGTGTGTGAAGGTAATGAGCTTGCCAAGTGCGAACATCAGTCCTTGATCAATGCTTATGACAATG
CCTTGCTTGCCACCGATGATTTCATCGCTCAAAGTATCCAGTGGCTGCAGACGCACAGCAATGCCTATGATGTCTCAATG
CTGTATGTCAGCGATCATGGCGAAAGTCTGGGTGAGAACGGTGTCTATCTACATGGTATGCCAAATGCCTTTGCACCAAA
AGAACAGCGCAGTGTGCCTGCATTTTTCTGGACGGATAAGCAAACTGGCATCACGCCAATGGCAACCGATACCGTCCTGA
CCCATGACGCGATCACGCCGACATTATTAAAGCTGTTTGATGTCACCGCGGACAAAGTCAAAGACCGCACCGCATTCATC
CGCTGATTTCTCCCTGTATTTTTTCCAAACCCACCGCACACTCCATTCGTATTATGGGCGGTGGGGTGGGGTTTGTTATG
CCGTATTTATCAAATAAACGCCTACTTGCTGAGATGAGTATCGCTCTTGTCATGGCGATCGTTGCCACGCTGACCCTTGA
GCACAGTCAGATTGATCTGATGGTCGCTGATTGGTTTTATCTGGGTATGGGGCATTGGATGGTTGCCAAGCAAGCTTTTT
TGCCAGATTTGCTACTGTATTCTGGACTAAAAAAGCTGCTGATGGCGATGCTGATCTACTTGCTGGTTGCGACCATTTGC
CGTGCTTATCATGAGAAAAAGGGCAATGCTATCACTGCCAAGTGGCTTGACGTATAGGAAATTGAAAAACCAGTCGATAC
CATTGTCCACGCCTTATGCCACGTGAGCCTTTCTGCTTTTCCCACCACTCCGGTGGTTTCCCTCTCTGTATGGTCGATTT
TTATACCGTAACAGGCTGTTTTTTATACTTTCACCGATTCCCGGGCACACTTAGCCTGTGTCAGGCCGGCATATACCTCA
GTAGACTGATATCCCGCGCGTTTTTCTTCAGCCCTGATACATTCAGCCCCGCAATGGCCCGGCGGTATACCATCCGGCAG
CCACACAGCACGCATTCGAACGGGTCACGACTCAGGAACTGTTTCACCATTTGTGCATAGCACACTTTCGCCACTGGTTC
CGGTTTATCCATCCCCAGTGCACGGTACACCTGCGGCAGCTTCTCTCCACACACACGGTTGGCAAGGAACCCGAAGTACC
TCACCATCTTAAAAAACTTCTCCGGGATGTGCTGTTTCAGCCTCGCGACCAGCTCACGCTGTGTCAGCGTTTCCGTCGCC

GTTTCTCCCGTTTTGTGGTCCAGGTAACGGAAGCTCAGGCTTGCCCCTCCGTTGTAATGAGCCAGTCGGGAAGCCGCTAT

TGGTGGCTTCTTCAGATAACGACCCAGGTAGCGTGCCGTATTCCGCCCTCCGGCTGTCTTCTTCGACATGTACACATGCC

AGTATTTTCCGCCGGATTTCAGCACCAGGCTTCTCCACTGTGATTCCGTCGTGATATGTGACAACGACTCCGGCATTGCC

ATCCCCTCTGACCACGCTTTCAGAAGCAGCTGCCGCATATTCCACATCCACCGTGAACGCATCGCGTCTTTCAGGAAGCT

CAGCTTTTTCCACTGACCATGCTTATTCAGACCTCCACAGGTTACAGACACATGTACATGCGGATGCCAGTTGAGACGAC

GGCCATACGTGTGGATGGCGCAGAAGATACCGGGTTCCAGCCCCCGTTTTCGGGCGGCATACAGCAGATTCTCCACCGCC

AGACGGCACACGTCATTCAGCAGCCAGCGGTTGCTTTCGAACACCGGCCACAGCGTGTCCGGCAGGGTGAAGACCAGATG

TACCCAGTCGCAGTCAGGAAGACGATTCAGCTGTGTTGCTGTCCACAGGTCTGTGGCCTTCTTTCCGCAGGACGGGCAGG

CACGGCTGCCGCATGAGTTGGTCAGGTACTTTACGTGCTGACAGTCCGGGTTATCACACCCGAACTCTTTTACACCCAGT

ATCCGTGTGCCGCAGGCCAGCATTTTGGTGACGGCTTCAACCTCGATATCGCGCAGACCGCCCGCATCCAGGAAGGACGT

CCAGCACTGGTTGGCTGTGAACAGACGTTTCAGAGGGCGGGGAGTAAAACCGGACAACATGAGGATGGATTAAACCCCGG

TATCCTGTAGCAGGGTAAGGGCTGCTGTATCGCCACGGCGCTTCAGGATGGAGGTCGTCACCATCGCAGGTGGTGGGTCC

ATGGGTTTGAGCATTTTCATCCAGTCCCATTCTCGTTGTGAGAGCTTCTCAGCACCAACAGAGAGCAGAACTGTATCGAT

ACTTTTGCGGGTCAACATAAGGGGATGAGTCCTTAATAAAACAGGTCCTCAGAGCATACATATTCTGCGGGGCGTGCGGC

AACAACAGGACGAACCGGGGCGGTTTTCAATTTGCAGCCGCCAGGCTGCCGTGGTTCTCCCAGTGACAAAATTTCGCGTG

CGTGAGCTTGCGTATCTGGTGCTGACTTTGATCCTAGTGCCGACAGTTGTCGCGTCATTGAAGGCATATACTCATGTGGT

CTGCCCTGTGCATTTGACGATTTTTGATGGTACGCTGCCGTATTTGCCGATGCTTGATAGTATGCGTAACACCATTCCTG

ATAAGTGCTTTCCTGCGGCGCATGCCAGTAGCGGATTTGCGCTGTTTGCCTTTGCGTTTGCGCCAAGTTTGCGCCGCCGT

CGTGGTGCGATCATCATCGTGGTGATGGCATTGGGCTGGGCGATGGGCTGCTATAAGATGATTATTGGCGATCATTTTTT

GAGCCATACGGTGGTGTCGATGATGCTTGCGTGGGCGATGTCGGCAGGGCTTGCGTGGGTGTTTTTAAGAAGGGTGAAC

AAGTTTAATCGGCTGAATTTACAATCCAAGTGCAACAAAAAAAGAAGTACTCATCAACTAGAATATAATTTTGTTTCCAC

ACAAAAACCACTTCCAAATGATGAGTACTT

>KX886345.1 Escherichia coli transposase (tnpA), MCR-1 (mcr-1), hypothetical protein, and transcriptional regulator (ydfA) genes, complete cds

ATCCTCTTATCCAGAGGGGGCATTAGTAACGTTTGATAGGCCATTTGAAATAATTGCCAATTCATATGAAGAATACCGTA

CTCCAGTGTATGCGGTTTCTTTTTCGAAGGATGAGTTTAGTGAAGATATTAAGACTTTTGAGTCATTGCACGACGCATCT

GAATATAAAAATAAGATGCTTCAGGAGCACGGGCTTAACCAGGATGATATCTTAATCACACCTGTAACAAGAGAAGAAAT

CGCCTTTAAAGGAATTAAGGACGCTGTTAATGACGCTAATATGGCAGTGATGGAACAGGCGGGGGATTCGTCCAGAGAAT

CACCAGAAGAGATACTCGCCAGTATTTCAGCTAACGAGCATATGATCTCCGGGCTTGAAAACTTCCTGGTTAAAGACCGT

TCTCAGTTCAGTTCCTGCAATGGGGATATCGTTGTGGAGGCGGAAATTACCAGGGGGGAAGGTGGTCTTTATCACCTTGC

GGTCGCTGGTAAGCATGGCTTAGAGCGTGGTGATGCAGTTGCCAGAGTGGATGTTACGGAGCAGCAATTTGCCGCGATCA

CTGGAAAAACACCTTCTGAAGTTTTAACGGGGGACCAAACATCGGCTCGTGTTCCGGTAATTACAGGTATCCATTTCAGT

ACAAGGGCTATTGAAAATGTGAATAAACTGGAACAACAGAAGGATTATGTCTATTTCTCAACACATGAAGGCCTGAATGC

TGAAATAAAAGATTTCAGCTCTCTCAAGGATGCTATTGAGTGGGGCCGGGTTGAATGCGAGCTACATGATCTCAATAAGC

GCGATACTGTCATTTATCGCGTCGAGTCTGAACATATTTCTCAGGGGATTGACGCTGTAATGAAAAATGCTGAACGCGTG

GAGCGTCATGAAATTGAGAAAGCACAGGGACGGGATTGCACGCCGGAAGACGGCAAGATTCTTGAGGCAATTGATCGCTT

TGAGGACAAGTTCAGAGGGGAAGGACTGAAGTTCGAAAGGGAGAAGGCCGAATCCGATTTGCTTAATCACGGATTCACTC

GTGAAATGGCAGAAGATGCTCTTGGAAAACAATTTGTCCAGGCCAGAGAAGAACATCTGGAATTGCAGCAACAGCGTGAC

AATTCACAGGACATGCACTAATTAAGAAAAAAGCCCGGTTTTCGGGCTTTTTAAGAGCTGAATTTACAATCCAAGTGCAA

CAAAAAAAGAAGTACTCATCAACTAGAATATAATTTTGTTTCCACACAAAAACCATTTCCAAATGATGAGTACTTCCTAC

CGACATCTTACAATAAACGAGCGAGAAAAGATAATGATTTTACTCGCACAGGGCAAAAAACAAGCAGAAATTGCCAAAGC

ACTGGGACGTAGCTCCAGCACCATTTCTCGCGAGCTGAAACGACACGCTCTAGAAAGCTACAGTGCAACGAACGCACAAA

ACAGCTATTTGAAGCATCGTCAAAATAGCAAAGCACAGCGCAAATTAGAGCAGCCTGAATATTTCAATTTGGTGCAAGAA

AAGTTTCTGACAGAAAACTGGTCGCCCGAACAAATCAGCGCACGATTAAAATTGGAAAAATCTGAATTATCCATTAGTTA

TTCAACCATTTATCGTGGTATTTATTCAGGGTTGTTTGATATAGGCGAACGCAAAGCCAGTCGCAAACTGCGCCACAAAG

GCAAAACACGGCATACAAAAAATCATCATGAAAAACGTGGCAAAATTCAGATATCCAACCATTTGAACGACCGTCCCATT

TCGGCGCAAAATCGCAGTCGCTTTGGACATTGGGAAGCCGATACCGTACTGGGTAAAGCGGGTGGAGCTTGTTTGCTGAC

GCTGACGGAACGCAAAAGTCGTTTTGAGTTGGTGAAGAAAATTCCTGCCAAAAAAGCCGAAGCAGTCCAAAAAGCCATGA

TTGAATTGCTGGATTCACATATATTGCGGTCAATTACGCCAGACCGTGGTAAAGAATTTGCCCAACATCGTTTGGTAACA

GAAGCACTGGGTGTAGAATTTTACTTCCCCGAGCCGCATCAACCGTGGACACGGGGAACGAATGAAAATACAAATGGGTT

ACTTCGTGAATACTTTCCGAAGCACCAAGACATCAATCAGTGGAGCGAAGTTGATATTCAACAGGTGATCAATAAACTGA

ATTTACGACCACGTAAATGTTTAGGTTGGAAAACACCTTATGAAGTTTACTTCAAAAAATCGTTGCACTTGGTTTGACAA

TTCAAGATACAAATTATAAATACTCTCAAGTGTATATTCAGTATGGGATTGCGCAATGATTGCCTAATAAAATTTCTGAA

ATATTTCTGTATCGCATAATTTTTTATATCAGATAAATTGTACTGGATTTCTTAAAAAATTGCAGTATAATTGCCGTAAT

TATCCCACCGTTTATTTTTTGAGTAGTTTCTCATGATGCAGCATACTTCTGTGTGGTACCGACGCTCGGTCAGTCCGTTT

GTTCTTGTGGCGAGTGTTGCCGTTTTCTTGACCGCGACCGCCAATCTTACCTTTTTTGATAAAATCAGCCAAACCTATCC

CATCGCGGACAATCTCGGCTTTGTGCTGACGATCGCTGTCGTGCTCTTTGGCGCGATGCTACTGATCACCACGCTGTTAT

CATCGTATCGCTATGTGCTAAAGCCTGTGTTGATTTTGCTATTAATCATGGGCGCGGTGACCAGTTATTTTACTGACACT

TATGGCACGGTCTATGATACGACCATGCTCCAAAATGCCCTACAGACCGACCAAGCCGAGACCAAGGATCTATTAAACGC

AGCGTTTATCATGCGTATCATTGGTTTGGGTGTGCTACCAAGTTTGCTTGTGGCTTTTGTTAAGGTGGATTATCCGACTT

GGGGCAAGGGTTTGATGCGCCGATTGGGCTTGATCGTGGCAAGTCTTGCGCTGATTTTACTGCCTGTGGTGGCGTTCAGC

AGTCATTATGCCAGTTTCTTTCGCGTGCATAAGCCGCTGCGTAGCTATGTCAATCCGATCATGCCAATCTACTCGGTGGG

TAAGCTTGCCAGTATTGAGTATAAAAAAGCCAGTGCGCCAAAAGATACCATTTATCACGCCAAAGACGCGGTACAAGCAA

CCAAGCCTGATATGCGTAAGCCACGCCTAGTGGTGTTCGTCGTCGGTGAGACGGCACGCGCCGATCATGTCAGCTTCAAT

GGCTATGAGCGCGATACTTTCCCACAGCTTGCCAAGATCGATGGCGTGACCAATTTTAGCAATGTCACATCGTGCGGCAC

ATCGACGGCGTATTCTGTGCCGTGTATGTTCAGCTATCTGGGCGCGGATGAGTATGATGTCGATACCGCCAAATACCAAG

AAAATGTGCTGGATACGCTGGATCGCTTGGGCGTAAGTATCTTGTGGCGTGATAATAATTCGGACTCAAAAGGCGTGATG

GATAAGCTGCCAAAAGCGCAATTTGCCGATTATAAATCCGCGACCAACAACGCCATCTGCAACACCAATCCTTATAACGA

ATGCCGCGATGTCGGTATGCTCGTTGGCTTAGATGACTTTGTCGCTGCCAATAACGGCAAAGATATGCTGATCATGCTGC

ACCAAATGGGCAATCACGGGCCTGCGTATTTTAAGCGATATGATGAAAAGTTTGCCAAATTCACGCCAGTGTGTGAAGGT

AATGAGCTTGCCAAGTGCGAACATCAGTCCTTGATCAATGCTTATGACAATGCCTTGCTTGCCACCGATGATTTCATCGC

TCAAAGTATCCAGTGGCTGCAGACGCACAGCAATGCCTATGATGTCTCAATGCTGTATGTCAGCGATCATGGCGAAAGTC

TGGGTGAGAACGGTGTCTATCTACATGGTATGCCAAATGCCTTTGCACCAAAAGAACAGCGCAGTGTGCCTGCATTTTTC

TGGACGGATAAGCAAACTGGCATCACGCCAATGGCAACCGATACCGTCCTGACCCATGACGCGATCACGCCGACATTATT

AAAGCTGTTTGATGTCACCGCGGACAAAGTCAAAGACCGCACCGCATTCATCCGCTGATTTCTCCCTGTATTTTTTCCAA

ACCCACCGCACACTCCATTCGTATTATGGGCGGTGGGGTGGGGTTTGTTATGCCGTATTTATCAAATAAACGCCTACTTG

CTGAGATGAGTATCGCTCTTGTCATGGCGATCGTTGCCACGCTGACCCTTGAGCACAGTCAGATTGATCTGATGGTCGCT

GATTGGTTTTATCTGGGTATGGGGCATTGGATGGTTGCCAAGCAAGCTTTTTTGCCAGATTTGCTACTGTATTCTGGACT

AAAAAAGCTGCTGATGGCGATGCTGATCTACTTGCTGGTTGCGACCATTTGCCGTGCTTATCATGAGAAAAAGGGCAATG

CTATCACTGCCAAGTGGCTTGTCCCAGTGACAAAATTTCGCGTGCGTGAGCTTGCGTATCTGGTGCTGACTTTGATCCTA

GTGCCGACAGTTGTCGCGTCATTGAAGGCATATACTCATGTGGTCTGCCCTGTGCATTTGACGATTTTTGATGGTACGCT

GCCGTATTTGCCGATGCTTGATAGTATGCGTAACACCATTCCTGATAAGTGCTTTCCTGCGGCGCATGCCAGTAGCGGAT

TTGCGCTGTTTGCCTTTGCGTTTGCGCCAAGTTTGCGCCGCCGTCGTGGTGCGATCATCATCGTGGTGATGGCATTGGGC

TGGGCGATGGGCTGCTATAAGATGATTATTGGCGATCATTTTTTGAGCCATACGGTGGTGTCGATGATGCTTGCGTGGGC

GATGTCGGCAGGGCTTGCGTGGGTGTTTTTTAAGAAGGGTGAACAAGTTCAAGGAAAAAAATCAACCCCAAACAGAAATA

ACAGCTACAAAAATGGACAGTAATAATGCAATAAAAGTAATGTTGAGCCATATGTTGGACGAGTCTTTTAAGACTAATGA

GGCTGTTAATGCCATTGTAATGAAACTAATGATAATAATGGCTGCAAATAAATATTGTACAATGTTTTTTATTGTACTTG

GTTGCTCCCCAATTCTTTCTCTGTAATTTGCATCAACATTAGATATCCATTGAAGTAATAGAGCAATAAAACAGCCTACT

CCGCAAACGGTGAATTGCACAGACGTCGGGTTTGAATCAAACAACGAGATCGCTAAGTTATATAAGTATACCGCGCCTGA

AATCACCCCGACAACTAAAATAGAGATGAGTATAATCCGAAGAACTAATGTATTGCTTTCCTTTAAGGAATATATTAAAG

CCGCTTTTTCGTATTCTTTCATGATTTAATCCAGTCTATAAGTATAAGAACCAACAAAACTGTCCAGGATTCATTTTCTG

TGAGCATATGAAATCCCATCCAGAGTGATGATACTGGCAGAAACACGCCCACAGCCGGAATTATTAGTCTGGCGGAGAAT

ATCCTTATCAGGAGTATCAATAGTGATAAGAACAGAAAAAGAGTGATCATGTGGAAAAACATTCACATCACCTATAACAC

TATTAAGATAGCCTGCCAGATATCCATTTCAGACAGGCTATATACAGGGTTCAATTCATTCTATGAAAGGTGTATAGGGA

TTGCTGATGTTTGTGTCGATCAAAAATCTAATATTAATAGATGAGAACTAATATACATATTTCCAGACGGATGGGGGAT

TTTATCGTAGAGTTTATTCATTGTGAGTTCTGCAAGCCTGTGATCGGCGGCGGCATTAAACGACTCAAGCTCAGAAGGAG

GGAGGCTGTTTTGGTTTTTTTCAATAATCTTTTCAAGTGTGTCGAGTGTTGAGCAACGTCTTAACTGAAAGAGCCAGTCT

TGTTTTGTTTTCATTGTACAATTCCTGAAGAACATCCTTGTCCTATAGTAGCTACAACGATGTTTGATTTCTACTGCTGG

ATTTATTTCTTACTTTTTTGTGTAAACTCAAATCCCAGTTTCCCGGTTGCTTTGTCAACCAGTTTTACTTTTGCTGAAAA

TTCTTTGTTTGTTTTTTGACTAATGAAACCTTTCAATTCACTTGTAACTCCTTTTGTTAT

>KX458104.1 Escherichia coli strain NDM132 plasmid pls1 insertion sequence ISApl1 integrase (int) gene, complete cds; mcr-1 (mcr-1) and hypothetical protein genes, complete cds; insertion sequence truncated ISApl1, complete sequence; insertion sequence ISKpn26 transposase (tnp) gene, complete cds; and insertion sequence truncated ISApl1, complete sequence

CAGCCAGAAGATGCTGGTGGCTCGATATAAAAATGGTTGGGTAATGGTGGATTGTTTTCTGTTTCATCATACCAAACCTA

AGAAAAAAGCAAAAAACAAAACTACTGTGGCTAAGCCTCAACTTAAAGAGGCTGAATTTACAATCCAAGTGCAACAAAAA

AAGAAGTACTCATCAACTAGAATATAATTTTGTTTCCACACAAAAACCATTTCCAAATGATGAGTACTTCCTACCGACAT

CTTACAATAAACGAGCGAGAAAAGATAATGATTTTACTCGCACAGGGCAAAAAACAAGCAGAAATTGCCAAAGCACTGGG

ACGTAGCTCCAGCACCATTTCTCGCGAGCTGAAACGACACGCTCTAGAAAGCTACAGTGCAACGAACGCACAAAACAGCT

ATTTGAAGCATCGTCAAAATAGCAAAGCACAGCGCAAATTAGAGCAGCCTGAATATTTCAATTTGGTGCAAGAAAAGTTT

CTGACAGAAAACTGGTCGCCCGAACAAATCAGCGCACGATTAAAATTGGAAAAATCTGAATTATCCATTAGTTATTCAAC

CATTTATCGTGGTATTTATTCAGGGTTGTTTGATATAGGCGAACGCAAAGCCAGTCGCAAACTGCGCCACAAAGGCAAAA

CACGGCATACAAAAAATCATCATGAAAAACGTGGCAAAATTCAGATATCCAACCATTTGAACGACCGTCCCATTTCGGCG

CAAAATCGCAGTCGCTTTGGACATTGGGAAGCCGATACCGTACTGGGTAAAGCGGGTGGAGCTTGTTTGCTGACGCTGAC

GGAACGCAAAAGTCGTTTTGAGTTGGTGAAGAAAATTCCTGCCAAAAAAGCCGAAGCAGTCCAAAAAGCCATGATTGAAT

TGCTGGATTCACATATATTGCGGTCAATTACGCCAGACCGTGGTAAAGAATTTGCCCAACATCGTTTGGTAACAGAAGCA

CTGGGTGTAGAATTTTACTTCCCCGAGCCGCATCAACCGTGGACACGGGGAACGAATGAAAATACAAATGGGTTACTTCG

TGAATACTTTCCGAAGCACCAAGACATCAATCAGTGGAGCGAAGTTGATATTCAACAGGTGATCAATAAACTGAATTTAC

GACCACGTAAATGTTTAGGTTGGAAAACACCTTATGAAGTTTACTTCAAAAAATCGTTGCACTTGGTTTGACAATTCAAG

ATACAAATTATAAATACTCTCAAGTGTATATTCAGTATGGGATTGCGCAATGATTGCCTAATAAAATTTCTGAAATATTT

CTGTATCGCATAATTTTTTATATCAGATAAATTGTACTGGATTTCTTAAAAAATTGCAGTATAATTGCCGCAATTATCCC

ACCGTTTATTTTTTTAATAGTTTCTCATGATGCAGCATACTTCTGTGTGGTACCGACGCTCGGTCAGTCCGTTTGTTCTT

GTGGCGAGTGTTGCCGTTTTCTTGACCGCGACCGCCAATCTTACCTTTTTTGATAAAATCAGCCAAACCTATCCCATCGC

GGACAATCTCGGCTTTGTGCTGACGATCGCTGTCGTGCTCTTTGGCGCGATGCTACTGATCACCACGCTGTTATCATCGT

ATCGCTATGTGCTAAAGCCTGTGTTGATTTTGCTATTAATCATGGGCGCGGTGACCAGTTATTTTACTGACACTTATGGC

ACGGTCTATGATACGACCATGCTCCAAAATGCCCTACAGACCGACCAAGCCGAGACCAAGGATCTATTAAACGCAGCGTT

TATCATGCGTATCATTGGTTTGGGTGTGCTACCAAGTTTGCTTGTGGCTTTTGTTAAGGTGGATTATCCGACTTGGGGCA

AGGGTTTGATGCGCCGATTGGGCTTGATCGTGGCAAGTCTTGCGCTGATTTTACTGCCTGTGGTGGCGTTCAGCAGTCAT

TATGCCAGTTTCTTTCGCGTGCATAAGCCGCTGCGTAGCTATGTCAATCCGATCATGCCAATCTACTCGGTGGGTAAGCT

TGCCAGTATTGAGTATAAAAAAGCCAGTGCGCCAAAAGATACCATTTATCACGCCAAAGACGCGGTACAAGCAACCAAGC

CTGATATGCGTAAGCCACGCCTAGTGGTGTTCGTCGTCGGTGAGACGGCACGCGCCGATCATGTCAGCTTCAATGGCTAT

GAGCGCGATACTTTCCCACAGCTTGCCAAGATCGATGGCGTGACCAATTTTAGCAATGTCACATCGTGCGGCACATCGAC

GGCGTATTCTGTGCCGTGTATGTTCAGCTATCTGGGCGCGGATGAGTATGATGTCGATACCGCCAAATACCAAGAAAATG

TGCTGGATACGCTGGATCGCTTGGGCGTAAGTATCTTGTGGCGTGATAATAATTCGGACTCAAAAGGCGTGATGGATAAG

CTGCCAAAAGCGCAATTTGCCGATTATAAATCCGCGACCAACAACGCCATCTGCAACACCAATCCTTATAACGAATGCCG

CGATGTCGGTATGCTCGTTGGCTTAGATGACTTTGTCGCTGCCAATAACGGCAAAGATATGCTGATCATGCTGCACCAAA

TGGGCAATCACGGGCCTGCGTATTTTAAGCGATATGATGAAAAGTTTGCCAAATTCACGCCAGTGTGTGAAGGTAATGAG

CTTGCCAAGTGCGAACATCAGTCCTTGATCAATGCTTATGACAATGCCTTGCTTGCCACCGATGATTTCATCGCTCAAAG

TATCCAGTGGCTGCAGACGCACAGCAATGCCTATGATGTCTCAATGCTGTATGTCAGCGATCATGGCGAAAGTCTGGGTG

AGAACGGTGTCTATCTACATGGTATGCCAAATGCCTTTGCACCAAAAGAACAGCGCAGTGTGCCTGCATTTTTCTGGACG

GATAAGCAAACTGGCATCACGCCAATGGCAACCGATACCGTCCTGACCCATGACGCGATCACGCCGACATTATTAAAGCT

GTTTGATGTCACCGCGGACAAAGTCAAAGACCGCACCGCATTCATCCGCTGATTTCTCCCTGTATTTTTCCAAACCCAC

CGCACACTCCATTCGTATTATGGGCGGTGGGGTGGGGTTTGTTATGCCGTATTTATCAAATAAACGCCTACTTGCTGAGA

TGAGTATCGCTCTTGTCATGGCGATCGTTGCCACGCTGACCCTTGAGCACAGTCAGATTGATCTGATGGTCGCTGATTGG

TTTTATCTGGGTATGGGGCATTGGATGGTTGCCAAGCAAGCTTTTTTGCCAGATTTGCTACTGTATTCTGGACTAAAAAA

GCTGCTGATGGCGATGCTGATCTACTTGCTGGTTGCGACCATTTGCCGTGCTTATCATGAGAAAAAGGGCAATGCTATCA

CTGCCAAGTGGCTTGTCCCAGTGACAAAATTTCGCGTGCGTGAGCTTGCGTATCTGGTGCTGACTTTGATCCTAGTGCCG

ACAGTTGTCGCGTCATTGAAGGCATATACTCATGTGGTCTGCCCTGTGCATTTGACGATTTTTGATGGTACGCTGCCGTA

TTTGCCGATGCTTGATAGTATGCGTAACACCATTCCTGATAAGTGCTTTCCTGCGGCGCATGCCAGTAGCGGATTTGCGC

TGTTTGCCTTTGCGTTTGCGCCAAGTTTGCGCCGCCGTCGTGGTGCGATCATCATCGTGGTGATGGCATTGGGCTGGGCG

ATGGGCTGCTATAAGATGATTATTGGCGATCATTTTTTGAGCCATACGGTGGTGTCGATGATGCTTGCGTGGGCGATGTC

GGCAGGGCTTGCGTGGGTGTTTTTTAAGAAGGGTGAACAAGTTTAATCGGCTGAATTTACAATCCAAGTGCAACAAAAAA

AGAAGTACTCATCAACTAGAATATAATTTTGTTTCCACACAAAAACCACTTCCAAATGATGAGTACTTCCTACCGACATC

TTACAATAAACGAGCGAGAAAAGATAATGATTTTACTCGCACAGGGCAAAAAACAAGCAGAAATTGCCAAAGCACTGGGA

CGTAGCTCCAGCACCATTTCTCGCGAGCTGAAACGACACGCTCTAGGGAAGGTGCGAACAAGTTCCTGATATGAGATCAT

CATATTCATCCGGAGCGCATCCCAGAGGGACATCATGAGCCATCAACTCACCTTCGCCGATAGTGAATTCAGCACTAAGC

GCCGTCAGACCCGAAAAGAGATTTTCCTCTCCCGCATGGAGCAGATTTTGCCATGGCAGAATATGACCGCTGTCATCGAG

CCGTTTTATCCCAAGGCGGGCAATGGCCGACGGCCCTATCCGCTGGAGACCATGCTGCGTATTCACTGCATGCAGCATTG

GTACAACCTGAGCGACGGTGCCATGGAAGATGCCCTGTACGAAATCGCCTCCATGCGCCTGTTTGCCCGATTATCCCTGG

ATAGCGCCCTGCCGGATCGCACCACCATCATGAATTTCCGCCACCTGCTCGAGCAGCATCAACTGGCCCGTCAATTGTTC

AAGACCATCAATCGCTGGCTGGCCGAAGCAGGCGTCATGATGACCCAAGGCACTTTGGTGGATGCCACCATCATTGAGGC

ACCCAGCTCTACCAAGAACAAAGAGCAGCAACGCGATCCGGAGATGCATCAGACCAAGAAAGGCAATCAGTGGCACTTTG

GCATGAAGGCCCACATTGGTGTCGATGCCAAGAGTGGCCTGACCCACAGCCTGGTCACCACCGCGGCCAACGAGCATGAC

CTCAATCAGCTGGGTAATCTGCTTCATGGAGAGGAGCAATTTGTCTCAGCCGATGCCGGCTACCAAGGAGCGCCACAGCG

CGAGGAGCTGGCCGAGGTGGATGTGGACTGGCTGATCGCCGAGCGTCCCGGCAGGGTAAAAACCTTGAAGCAGCATCCGC

GCAAGAACAAAACGGCCATCAACATCGAATACATGAAAGCCAGCATCCGTGCCAGGGTGGAGCACCCGTTTCGCATCATC

AAGCGGCAGTTCGGCTTCGTGAAAGCCAGATACAAGGGGCTGCTGAAAAACGATAACCAACTGGCGATGTTATTCACCCT

GGCCAACCTGTTTCGGGTGGACCAAATGATACGTCAGTGGGAGAGATCTCAGTAAAAACCGGAAATAACGCCAGAAATGG

TGGAAAAAATAGCCTAAATAGGCTGATTCGATGTGTTTGCGGAAAAAAATCGGCCCAGATCCGCGAAATTTTAATCAGC

GAGTCAGCTTGGGAAGAAATGACCTGCTTATTCGCACCTTCCCTAGAAAGCTACAGTGCAACGAACGCACAAAACAGCTA

TTTGAAGCATCGTCAAAATAGCAAAGCACAGCGCAAATTAGAGCAGCCTGAATATTTCAATTTGGTGCAAGAAAAGTTTC

TGACAGAAAACTGGTCGCCCGAACAAATCAGCGCACGATTAAAATTGGAAAAATCTGAATTATCCATTAGTTATTCAACC

ATTTATCGTGGTATTTATTCAGGGTTGTTTGATATAGGCGAACGCAAAGCCAGTCGCAAACTGCGCCACAAAGGCAAAAC

ACGGCATACAAAAAATCATCATGAAAAACGTGGCAAAATTCAGATATCCAACCATTTGAACGACCGTCCCATTTCGGCGC

AAAATCGCAGTCGCTTTGGACATTGGGAAGCCGATACCGTACTGGGTAAAGCGGGTGGAGCTTGTTTGCTGACGCTGACG

GAACGCAAAAGTCGTTTTGAGTTGGTGAAGAAAATTCCTGCCAAAAAAGCCGAAGCAGTCCAAAAAGCCATGATTGAATT

GCTGGATTCACATATATTGCGGTCAATTACGCCAGACCGTGGTAAAGAATTTGCCCAACATCGTTTGGTAACAGAAGCAC

TGGGTGTAGAATTTTACTTCCCCGAGCCGCATCAACCGTGGACACGGGGAACGAATGAAAATACAAATGGGTTACTTCGT

GAATACTTTCCGAAGCACCAAGACATCAATCAGTGGAGCGAAGTTGATATTCAACAGGTGATCAATAAACTGAATTTACG

ACCACGTAAATGTTTAGGTTGGAAAACACCTTATGAAGTTTACTTCAAAAAATCGTTGCACTTGGTTTGACAATTCAAGA

GACTAAAAGAGAAGGGAGTGAAAATGGAACTGGGCATTATGGTGTGCATCGTGTTGTTACTCTCCGTGGCGGCGGCGTGG

ATCAGTAATAAGACAGTTGATTTGCTCGAAAGATTCTATTTCAAGCGTCCGCTGAGCATGGAGTACGCGGCATGGCTACG

TGTAATGTGTGCAGGCTTGTTATTGGTTTTTATTCAGGTTGCTCCACAATTTACACTTCTGTATACCCTTAAACTTGTCA

CGCTTATTAATCTTGGAATCCAGACAATGAAATTAAATGGCGTGTACAAATATAAAAGAACGG

>NG_050417.1 Escherichia coli SHP45 pHNSHP45 mcr-1 gene for phosphoethanolamine--lipid A transferase MCR-1.1, complete CDS

CGCATAATTTTTTATATCAGATAAATTGTACTGGATTTCTTAAAAAATTGCAGTATAATTGCCGTAATTATCCCACCGTT

TATTTTTTGAGTAGTTTCTCATGATGCAGCATACTTCTGTGTGGTACCGACGCTCGGTCAGTCCGTTTGTTCTTGTGGCG

AGTGTTGCCGTTTTCTTGACCGCGACCGCCAATCTTACCTTTTTTGATAAAATCAGCCAAACCTATCCCATCGCGGACAA

TCTCGGCTTTGTGCTGACGATCGCTGTCGTGCTCTTTGGCGCGATGCTACTGATCACCACGCTGTTATCATCGTATCGCT

ATGTGCTAAAGCCTGTGTTGATTTTGCTATTAATCATGGGCGCGGTGACCAGTTATTTTACTGACACTTATGGCACGGTC

TATGATACGACCATGCTCCAAAATGCCCTACAGACCGACCAAGCCGAGACCAAGGATCTATTAAACGCAGCGTTTATCAT

GCGTATCATTGGTTTGGGTGTGCTACCAAGTTTGCTTGTGGCTTTTGTTAAGGTGGATTATCCGACTTGGGGCAAGGGTT

TGATGCGCCGATTGGGCTTGATCGTGGCAAGTCTTGCGCTGATTTTACTGCCTGTGGTGGCGTTCAGCAGTCATTATGCC

AGTTTCTTTCGCGTGCATAAGCCGCTGCGTAGCTATGTCAATCCGATCATGCCAATCTACTCGGTGGGTAAGCTTGCCAG

TATTGAGTATAAAAAAGCCAGTGCGCCAAAAGATACCATTTATCACGCCAAAGACGCGGTACAAGCAACCAAGCCTGATA

TGCGTAAGCCACGCCTAGTGGTGTTCGTCGTCGGTGAGACGGCACGCGCCGATCATGTCAGCTTCAATGGCTATGAGCGC

GATACTTTCCCACAGCTTGCCAAGATCGATGGCGTGACCAATTTTAGCAATGTCACATCGTGCGGCACATCGACGGCGTA

TTCTGTGCCGTGTATGTTCAGCTATCTGGGCGCGGATGAGTATGATGTCGATACCGCCAAATACCAAGAAAATGTGCTGG

ATACGCTGGATCGCTTGGGCGTAAGTATCTTGTGGCGTGATAATAATTCGGACTCAAAAGGCGTGATGGATAAGCTGCCA

AAAGCGCAATTTGCCGATTATAAATCCGCGACCAACAACGCCATCTGCAACACCAATCCTTATAACGAATGCCGCGATGT

CGGTATGCTCGTTGGCTTAGATGACTTTGTCGCTGCCAATAACGGCAAAGATATGCTGATCATGCTGCACCAAATGGGCA

ATCACGGGCCTGCGTATTTTAAGCGATATGATGAAAAGTTTGCCAAATTCACGCCAGTGTGTGAAGGTAATGAGCTTGCC

AAGTGCGAACATCAGTCCTTGATCAATGCTTATGACAATGCCTTGCTTGCCACCGATGATTTCATCGCTCAAAGTATCCA

GTGGCTGCAGACGCACAGCAATGCCTATGATGTCTCAATGCTGTATGTCAGCGATCATGGCGAAAGTCTGGGTGAGAACG

GTGTCTATCTACATGGTATGCCAAATGCCTTTGCACCAAAAGAACAGCGCAGTGTGCCTGCATTTTTCTGGACGGATAAG

CAAACTGGCATCACGCCAATGGCAACCGATACCGTCCTGACCCATGACGCGATCACGCCGACATTATTAAAGCTGTTTGA

TGTCACCGCGGACAAAGTCAAAGACCGCACCGCATTCATCCGCTGATTTCTCCCTGTATTTTTTCCAAACCCACCGCACA

CTCCATTCGTATTATGGGCGGTGGGGTGGGGTTTGTTATGCCGTATTTATCAAATAAACGCCTACT

>LC114017.1 Escherichia coli mcr-1 gene for phosphoethanolamine-lipid A transferase MCR-1, complete cds, strain: M165

ATGATGCAGCATACTTCTGTGTGGTACCGACGCTCGGTCAGTCCGTTTGTTCTTGTGGCGAGTGTTGCCGTTTTCTTGAC

CGCGACCGCCAATCTTACCTTTTTTGATAAAATCAGCCAAACCTATCCCATCGCGGACAATCTCGGCTTTGTGCTGACGA

TCGCTGTCGTGCTCTTTGGCGCGATGCTACTGATCACCACGCTGTTATCATCGTATCGCTATGTGCTAAAGCCTGTGTTG

ATTTTGCTATTAATCATGGGCGCGGTGACCAGTTATTTTACTGACACTTATGGCACGGTCTATGATACGACCATGCTCCA
AAATGCCCTACAGACCGACCAAGCCGAGACCAAGGATCTATTAAACGCAGCGTTTATCATGCGTATCATTGGTTTGGGTG
TGCTACCAAGTTTGCTTGTGGCTTTTGTTAAGGTGGATTATCCGACTTGGGGCAAGGGTTTGATGCGCCGATTGGGCTTG
ATCGTGGCAAGTCTTGCGCTGATTTTACTGCCTGTGGTGGCGTTCAGCAGTCATTATGCCAGTTTCTTTCGCGTGCATAA
GCCGCTGCGTAGCTATGTCAATCCGATCATGCCAATCTACTCGGTGGGTAAGCTTGCCAGTATTGAGTATAAAAAAGCCA
GTGCGCCAAAAGATACCATTTATCACGCCAAAGACGCGGTACAAGCAACCAAGCCTGATATGCGTAAGCCACGCCTAGTG
GTGTTCGTCGTCGGTGAGACGGCACGCGCCGATCATGTCAGCTTCAATGGCTATGAGCGCGATACTTTCCCACAGCTTGC
CAAGATCGATGGCGTGACCAATTTTAGCAATGTCACATCGTGCGGCACATCGACGGCGTATTCTGTGCCGTGTATGTTCA
GCTATCTGGGCGCGGATGAGTATGATGTCGATACCGCCAAATACCAAGAAAATGTGCTGGATACGCTGGATCGCTTGGGC
GTAAGTATCTTGTGGCGTGATAATAATTCGGACTCAAAAGGCGTGATGGATAAGCTGCCAAAAGCGCAATTTGCCGATTA
TAAATCCGCGACCAACAACGCCATCTGCAACACCAATCCTTATAACGAATGCCGCGATGTCGGTATGCTCGTTGGCTTAG
ATGACTTTGTCGCTGCCAATAACGGCAAAGATATGCTGATCATGCTGCACCAAATGGGCAATCACGGGCCTGCGTATTTT
AAGCGATATGATGAAAAGTTTGCCAAATTCACGCCAGTGTGTGAAGGTAATGAGCTTGCCAAGTGCGAACATCAGTCCTT
GATCAATGCTTATGACAATGCCTTGCTTGCCACCGATGATTTCATCGCTCAAAGTATCCAGTGGCTGCAGACGCACAGCA
ATGCCTATGATGTCTCAATGCTGTATGTCAGCGATCATGGCGAAAGTCTGGGTGAGAACGGTGTCTATCTACATGGTATG
CCAAATGCCTTTGCACCAAAAGAACAGCGCAGTGTGCCTGCATTTTTCTGGACGGATAAGCAAACTGGCATCACGCCAAT
GGCAACCGATACCGTCCTGACCCATGACGCGATCACGCCGACATTATTAAAGCTGTTTGATGTCACCGCGGACAAAGTCA
AAGACCGCACCGCATTCATCCGCTGA

>NG_068217.1 Escherichia coli 803_18 mcr-1 gene for phosphoethanolamine--lipid A transferase MCR-1.26, complete CDS

ATAATTTTTTATATCAGATAAATTGTACTGGATTTCTTAAAAAATTGCAGTATAATTGCCGCAATTATCCCACCGTTTAT

TTTTTGAGTAGTTTCTCACGATGCAGCATACTTCTGTGTGGTACCGACGCTCGGTCAGTCCGTTTGTTCTTGTGGCGAGT

GTTGCCGTTTTCTTGACCGCGACCGCCAATCTTACCTTTTTTGATAAAATCAGCCAAACCTATCCCATCGCGGACAATCT

CGGCTTTGTGCTGACGATCGCTGTCGTGCTCTTTGGCGCGATGCTACTGATCACCACGCTGTTATCATCGTATCGCTATG

TGCTAAAGCCTGTGTTGATTTTGCTATTAATCATGGGCGCGGTGACCAGTTATTTTACTGACACTTATGGCACGGTCTAT

GATACGACCATGCTCCAAAATGCCCTACAGACCGACCAAGCCGAGACCAAGGATCTATTAAACGCAGCGTTTATCATGCG

TATCATTGGTTTGGGTGTGCTACCAAGTTTGCTTGTGGCTTTTGTTAAGGTGGATTATCCGACTTGGGGCAAGGGTTTGA

TGCGCCGATTGGGCTTGATCGTGGCAAGTCTTGCGCTGATTTTACTGCCTGTGGTGGCGTTCAGCAGTCATTATGCCAGT

TTCTTTCGCGTGCATAAGCCGCTGCGTAGCTATGTCAATCCGATCATGCCAATCTACTCGGTGGGTAAGCTTGCCAGTAT

TGAGTATAAAAAAGCCAGTGCGCCAAAAGATACCATTTATCACGCCAAAGACGCGGTACAAGCAACCAAGCCTGATATGC

GTAAGCCACGCCTAGTGGTGTTCGTCGTCGGTGAGACGGCACGCGCCGATCATGTCAGCTTCAATGGCTATGAGCGCGAT

ACTTTCCCACAGCTTGCCAAGATCGATGGCGTGACCAATTTTAGCAATGTCACATCGTGCGGCACATCGACGGCGTATTC

TGTGCCGTGTATGTTCAGCTATCTGGGCGCGGATGAGTATGATGTCGATACCGCCAAATACCAAGAAAATGTGCTGGATA

CGCTGGATCGCTTGGGCGTAAGTATCTTGTGGCGTGATAATAATTCGGACTCAAAAGGCGTGATGGATAAGCTGCCAAAA

GCGCAATTTGCCGATTATAAATCCGCGACCAACAACGCCATCTGCAACACCAATCCTTATAACGAATGCCGCGATGTCGG

TATGCTCGTTGGCTTAGATGACTTTGTCGCTGCCAATAACGGCAAAGATATGCTGATCATGCTGCACCAAATGGGCAATC

ACGGGCCTGCGTATTTTAAGCGATATGATGAAAAGTTTGCCAAATTCACGCCAGTGTGTGAAGGTAATGAGCTTGCCAAG

TGCGAACATCAGTCCTTGATCAATGCTTATGACAATGCCTTGCTTGCCACCGATGATTTCATCGCTCAAAGTATCCAGTG

GCTGCAGACGCACAGCAATGCCTATGATGTCTCAATGCTGTATGTCAGCGATCATGGCGAAAGTCTGGGTGAGAACGGTG

TCTATCTACATGGTATGCCAAATGCCTTTGCACCAAAAGAACAGCGCAGTGTGCCTGCATTTTTCTGGACGGATAAGCAA

ACTGGCATCACGCCAATGGCAACCGATACCGTCCTGACCCATGACGCGATCACGCCGACATTATTAAAGCTGTTTGATGT

CACCGCGGACAAAGTCAAAGACCGCACCGCATTCATCCGCTGATTTCTCCCTGTATTTTTCCAAACCCACCGCACACTC

CATTCGTATTATGGGCGGTGGGGTGGGGTTTGTTATGCCGTATTTATCAAATAAACGCCTACT

>MN879258.1 Escherichia coli strain SAUVM_E5 MCR-1 family phosphoethanolamine--lipid A transferase (mcr-1) gene, partial cds

ATGATGCAGCATACTTCTGTGTGGTACCGACGCTCGGTCAGTCCGTTTGTTCTTGTGGCGAGTGTTGCCGTTTTCTTGAC

CGCGACCGCCAATCTTACCTTTTTTGATAAAATCAGCCAAACCTATCCCATCGCGGACAATCTCGGCTTTGTGCTGACGA

TCGCTGTCGTGCTCTTTGGCGCGATGCTACTGATCACCACGCTGTTATCATCGTATCGCTATGTGCTAAAGCCTGTGTTG

ATTTTGCTATTAATCATGGGCGCGGTGACCAGTTATTTTACTGACACTTATGGCACGGTCTATGATACGACCATGCTCCA

AAATGCCCTACAGACCGACCAAGCCGAGACCAAGGATCTATTAAACGCAGCGTTTATCATGCGTATCATTGGTTTGGGTG

TGCTACCAAGTTTGCTTGTGGCTTTTGTTAAGGTGGATTATCCGACTTGGGGCAAGGGTTTGATGCGCCGATTGGGCTTG

ATCGTGGCAAGTCTTGCGCTGATTTTACTGCCTGTGGTGGCGTTCAGCAGTCATTATGCCAGTTTCTTTCGCGTGCATAA

GCCGCTGCGTAGCTATGTCAATCCGATCATGCCAATCTACTCGGTGGGTAAGCTTGCCAGTATTGAGTATAAAAAAGCCA

GTGCGCCAAAAGATACCATTTATCACGCCAAAGACGCGGTACAAGCAACCAAGCCTGATATGCGTAAGCCACGCCTAGTG

GTGTTCGTCGTCGGTGAGACGGCACGCGCCGATCATGTCAGCTTCAATGGCTATGAGCGCGATACTTTCCCACAGCTTGC

CAAGATCGATGGCGTGACCAATTTTAGCAATGTCACATCGTGCGGCACATCGACGGCGTATTCTGTGCCGTGTATGTTCA

GCTATCTGGGCGCGGATGAGTATGATGTCGATACCGCCAAATACCAAGAAAATGTGCTGGATACGCTGGATCGCTTGGGC

GTAAGTATCTTGTGGCGTGATAATAATTCGGACTCAAAAGGCGTGATGGATAAGCTGCCAAAAGCGCAATTTGCCGATTA

TAAATCCGCGACCAACAACGCCATCTGCAACACCAATCCTTATAACGAATGCCGCGATGTCGGTATGCTCGTTGGCTTAG

ATGACTTTGTCGCTGCCAATAACGGCAAAGATATGCTGATCATGCTGCACCAAATGGGCAATCACGGGCCTGCGTATTTT

AAGCGATATGATGAAAAGTTTGCCAAATTCACGCCAGTGTGTGAAGGTAATGAGCTTGCCAAGTGCGAACATCAGTCCTT

GATCAATGCTTATGACAATGCCTTGCTTGCCACCGATGATTTCATCGCTCAAAGTATCCAGTGGCTGCAGACGCACAGCA

ATGCCTATGATGTCTCAATGCTGTATGTCAGCGATCATGGCGAAAGTCTGGGTGAGAACGGTGTCTATCTACATGGTATG

CCAAATGCCTTTGCACCAAAAGAACAGCGCAGTGTGCCTGCATTTTTCTGGACGGATAAGCAAACTGGCATCACGCCAAT

GGCAACCGATACCGTCCTGACCCATGACGCGATCACGCCGACATTATTAAAGCTGTTTGATGTCACCGCGGACAAAGTCA

AAGACCGCACCGCATNCATCCGCTGA

>KY685070.1 Escherichia coli strain HKSH_MCR_161103180_EC phosphoethanolamine lipid A transferase (mcr1) gene, mcr1.8 allele, complete cds

ATGATGCAGCATACTTCTGTGTGGTACCGACGCTCGGTCAGTCCGTTTGTTCTTGTGGCGAGTGTTGCCGTTTTCTTGAC

CGCGACCGCCAATCTTACCTTTTTTGATAAAATCAGCCAAACCTATCCCATCGCGGACAATCTCGGCTTTGTGCTGACGA

TCGCTGTCGTGCTCTTTGGCGCGATGCTACTGATCACCACGCTGTTATCATCGTATCGCTATGTGCTAAAGCCTGTGTTG

ATTTTGCTATTAATCATGGGCGCGGTGACCAGTTATTTTACTGACACTTATGGCACGGTCTATGATACGACCATGCTCCA

AAATGCCCTACAGACCGACCAAGCCGAGACCAAGGATCTATTAAACGCAGCGTTTATCATGCGTATCATTGGTTTGGGTG

TGCTACCAAGTTTGCTTGTGGCTTTTGTTAAGGTGGATTATCCGACTTGGGGCAAGGGTTTGATGCGCCGATTGGGCTTG

ATCGTGGCAAGTCTTGCGCTGATTTTACTGCCTGTGGTGGCGTTCAGCAGTCATTATGCCAGTTTCTTTCGCGTGCATAA

GCCGCTGCGTAGCTATGTCAATCCGATCATGCCAATCTACTCGGTGGGTAAGCTTGCCAGTATTGAGTATAAAAAAGCCA

GTGCGCCAAAAGATACCATTTATCACGCCAAAGACGCGGTACAAGCAACCAAGCCTGATATGCGTAAGCCACGCCTAGTG

GTGTTCGTCGTCGGTGAGACGGCACGCGCCGATCATGTCAGCTTCAATGGCTATGAGCGCGATACTTTCCCACAGCTTGC

CAAGATCGATGGCGTGACCAATTTTAGCAATGTCACATCGTGCGGCACATCGACGGCGTATTCTGTGCCGTGTATGTTCA

GCTATCTGGGCGCGGATGAGTATGATGTCGATACCGCCAAATACCAAGAAAATGTGCTGGATACGCTGGATCGCTTGGGC

GTAAGTATCTTGTGGCGTGATAATAATTCGGACTCAAAAGGCGTGATGGATAAGCTGCCAAAAGCGCAATTTGCCGATTA

TAAATCCGCGACCAACAACGCCATCTGCAACACCAATCCTTATAACGAATGCCGCGATGTCGGTATGCTCGTTGGCTTAG

ATGACTTTGTCGCTGCCAATAACGGCAAAGATATGCTGATCATGCTGCACCAAATGGGCAATCACGGGCCTGCGTATTTT

AAGCGATATGATGAAAAGTTTGCCAAATTCACGCCAGTGTGTGAAGGTAATGAGCTTGCCAARTGCGAACATCAGTCCTT

GATCAATGCTTATGACAATGCCTTGCTTGCCACCGATGATTTCATCGCTCAAAGTATCCAGTGGCTGCAGACGCACAGCA

ATGCCTATGATGTCTCAATGCTGTATGTCAGCGATCATGGCGAAAGTCTGGGTGAGAACGGTGTCTATCTACATGGTATG

CCAAATGCCTTTGCACCAAAAGAACAGCGCAGTGTGCCTGCATTTTTCTGGACGGATAAGCAAACTGGCATCACGCCAAT

GGCAACCGATACCGTCCTGACCCATGACGCGATCACGCCGACATTATTAAAGCTGTTTGATGTCACCGCGGACAAAGTCA

AAGACCGCACCGCATTCATCCGCTGA

>NG_068218.1 Escherichia coli 844_18 mcr-1 gene for phosphoethanolamine--lipid A transferase MCR-1.27, complete CDS

CGCATAATTTTTATATCAGATAAATTGTACTGGATTTCTTAAAAAATTGCAGTATAATTGCCGCAATTATCCCACCGTT

TATTTTTTGAGTAGTTTCTCATGATGCAGCATACTTCTGTGTGGTGCCGACGCTCGGTCAGTCCGTTTGTTCTTGTGGCG

AGTGTTGCCGTTTTCTTGACCGCGACCGCCAATCTTACCTTTTTTGATAAAATCAGCCAAACCTATCCCATCGCGGACAA

TCTCGGCTTTGTGCTGACGATCGCTGTCGTGCTCTTTGGCGCGATGCTACTGATCACCACGCTGTTATCATCGTATCGCT

ATGTGCTAAAGCCTGTGTTGATTTTGCTATTAATCATGGGCGCGGTGACCAGTTATTTTACTGACACTTATGGCACGGTC

TATGATACGACCATGCTCCAAAATGCCCTACAGACCGACCAAGCCGAGACCAAGGATCTATTAAACGCAGCGTTTATCAT

GCGTATCATTGGTTTGGGTGTGCTACCAAGTTTGCTTGTGGCTTTTGTTAAGGTGGATTATCCGACTTGGGGCAAGGGTT

TGATGCGCCGATTGGGCTTGATCGTGGCAAGTCTTGCGCTGATTTTACTGCCTGTGGTGGCGTTCAGCAGTCATTATGCC

AGTTTCTTTCGCGTGCATAAGCCGCTGCGTAGCTATGTCAATCCGATCATGCCAATCTACTCGGTGGGTAAGCTTGCCAG

TATTGAGTATAAAAAAGCCAGTGCGCCAAAAGATACCATTTATCACGCCAAAGACGCGGTACAAGCAACCAAGCCTGATA

TGCGTAAGCCACGCCTAGTGGTGTTCGTCGTCGGTGAGACGGCACGCGCCGATCATGTCAGCTTCAATGGCTATGAGCGC

GATACTTTCCCACAGCTTGCCAAGATCGATGGCGTGACCAATTTTAGCAATGTCACATCGTGCGGCACATCGACGGCGTA

TTCTGTGCCGTGTATGTTCAGCTATCTGGGCGCGGATGAGTATGATGTCGATACCGCCAAATACCAAGAAAATGTGCTGG

ATACGCTGGATCGCTTGGGCGTAAGTATCTTGTGGCGTGATAATAATTCGGACTCAAAAGGCGTGATGGATAAGCTGCCA

AAAGCGCAATTTGCCGATTATAAATCCGCGACCAACAACGCCATCTGCAACACCAATCCTTATAACGAATGCCGCGATGT

CGGTATGCTCGTTGGCTTAGATGACTTTGTCGCTGCCAATAACGGCAAAGATATGCTGATCATGCTGCACCAAATGGGCA

ATCACGGGCCTGCGTATTTTAAGCGATATGATGAAAAGTTTGCCAAATTCACGCCAGTGTGTGAAGGTAATGAGCTTGCC

AAGTGCGAACATCAGTCCTTGATCAATGCTTATGACAATGCCTTGCTTGCCACCGATGATTTCATCGCTCAAAGTATCCA

GTGGCTGCAGACGCACAGCAATGCCTATGATGTCTCAATGCTGTATGTCAGCGATCATGGCGAAAGTCTGGGTGAGAACG

GTGTCTATCTACATGGTATGCCAAATGCCTTTGCACCAAAAGAACAGCGCAGTGTGCCTGCATTTTTCTGGACGGATAAG

CAAACTGGCATCACGCCAATGGCAACCGATACCGTCCTGACCCATGACGCGATCACGCCGACATTATTAAAGCTGTTTGA

TGTCACCGCGGACAAAGTCAAAGACCGCACCGCATTCATCCGCTGATTTCTCCCTGTATTTTTCCAAACCCACCGCACA

CTCCATTCGTATTATGGGCGGTGGGGTGGGGTTTGTTATGCCGTATTTATCAAATAAACGCCTACT

>NG_065944.1 Escherichia coli NIG-EC49 mcr-1 gene for phosphoethanolamine--lipid A transferase MCR-1.22, complete CDS

ATGATGCAGCATACTTCTGTGTGGTACCGACGCTCGGTCAGTCCGTTTGTTCTTGTGGCGAGTGTTGCCGTTTTCTTGAC

CGCGACCGCCAATCTTACCTTTTTTGATAAAATCAGCCAAACCTATCCCATCGCGGACAATCTCGGCTTTGTGCTGACGA

TCGCTGTCGTGCTCTTTGGCGCGATGCTACTGATCACCACGCTGTTATCATCGTATCGCTATGTGCTAAAGCCTGTGTTG

ATTTTGCTATTAATCATGGGCGCGGTGACCAGTTATTTACTGACACTTATGGCACGGTCTATGATACGACCATGCTCCA

AAATGCCCTACAGACCGACCAAGCCGAGACCAAGGATCTATTAAACGCAGCGTTTATCATGCGTATCATTGGTTTGGGTG

TGCTACCAAGTTTGCTTGTGGCTTTTGTTAAGGTGGATTATCCGACTTGGGGCAAGGGTTTGATGCGCCGATTGGGCTTG

ATCGTGGCAAGTCTTGCGCTGATTTTACTGCCTGTGGTGGCGTTCAGCAGTCATTATGCCAGTTTCTTTCGCGTGCATAA

GCCGCTGCGTAGCTATGTCAATCCGATCATGCCAATCTACTCGGTGGGTAAGCTTGCCAGTATTGAGTATAAAAAAGCCA

GTGCGCCAAAAGATACCATTTATCACGCCAAAGACGCGGTACAAGCAACCAAGCCTGATATGCGTAAGCCACGCCTAGTG

GTGTTCGTCGTCGGTGAGACGGCACGCGCCGATCATGTCAGCTTCAATGGCTATGAGCGCGATACTTTCCCACAGCTTGC

CAAGATCGATGGCGTGACCAATTTTAGCAATGTCACATCGTGCGGCACATCGACGGCGTATTCTGTGCCGTGTATGTTCA

GCTATCTGGGCGCGGATGAGTATGATGTCGATACCGCCAAATACCAAGAAAATGTGCTGGATACGCTGGATCGCTTGGGC

GTAAGTATCTTGTGGCGTGATAATAATTCGGACTCAAAAGGCGTGATGGATAAGCTGCCAAAAGCGCAATTTGCCGATTA

TAAATCCGCGACCAACAACGCCATCTGCAACACCAATCCTTATAACGAATGCCGCGATGTCGGTATGCTCGTTGGCTTAG

ATGACTTTGTCGCTGCCAATAACGGCAAAGATATGCTGATCATGCTGCACCAAATGGGCAATCACGGGCCTGCGTATTTT

AAGCGATATGATGAAAAGTTTGCCAAATTCACGCCAGTGTGTGAAGGTAATGAGCTTGCCAAGTGCGAACATCAGTTCTT

GATCAATGCTTATGACAATGCCTTGCTTGCCACCGATGATTTCATCGCTCAAAGTATCCAGTGGCTGCAGACGCACAGCA

ATGCCTATGATGTCTCAATGCTGTATGTCAGCGATCATGGCGAAAGTCTGGGTGAGAACGGTGTCTATCTACATGGTATG

CCAAATGCCTTTGCACCAAAAGAACAGCGCAGTGTGCCTGCATTTTTCTGGACGGATAAGCAAACTGGCATCACGCCAAT

GGCAACCGATACCGTCCTGACCCATGACGCGATCACGCCGACATTATTAAAGCTGTTTGATGTCACCGCGGACAAAGTCA

AAGACCGCACCGCATTCATCCGCTGA

>MN017134.1 Escherichia coli strain NIG-EC49 phosphoethanolamine--lipid A transferase MCR-1.22 (mcr-1) gene, mcr-1.22 allele, complete cds

ATGATGCAGCATACTTCTGTGTGGTACCGACGCTCGGTCAGTCCGTTTGTTCTTGTGGCGAGTGTTGCCGTTTTCTTGAC

CGCGACCGCCAATCTTACCTTTTTTGATAAAATCAGCCAAACCTATCCCATCGCGGACAATCTCGGCTTTGTGCTGACGA

TCGCTGTCGTGCTCTTTGGCGCGATGCTACTGATCACCACGCTGTTATCATCGTATCGCTATGTGCTAAAGCCTGTGTTG

ATTTTGCTATTAATCATGGGCGCGGTGACCAGTTATTTTACTGACACTTATGGCACGGTCTATGATACGACCATGCTCCA

AAATGCCCTACAGACCGACCAAGCCGAGACCAAGGATCTATTAAACGCAGCGTTTATCATGCGTATCATTGGTTTGGGTG

TGCTACCAAGTTTGCTTGTGGCTTTTGTTAAGGTGGATTATCCGACTTGGGGCAAGGGTTTGATGCGCCGATTGGGCTTG

ATCGTGGCAAGTCTTGCGCTGATTTTACTGCCTGTGGTGGCGTTCAGCAGTCATTATGCCAGTTTCTTTCGCGTGCATAA

GCCGCTGCGTAGCTATGTCAATCCGATCATGCCAATCTACTCGGTGGGTAAGCTTGCCAGTATTGAGTATAAAAAAGCCA

GTGCGCCAAAAGATACCATTTATCACGCCAAAGACGCGGTACAAGCAACCAAGCCTGATATGCGTAAGCCACGCCTAGTG

GTGTTCGTCGTCGGTGAGACGGCACGCGCCGATCATGTCAGCTTCAATGGCTATGAGCGCGATACTTTCCCACAGCTTGC

CAAGATCGATGGCGTGACCAATTTTAGCAATGTCACATCGTGCGGCACATCGACGGCGTATTCTGTGCCGTGTATGTTCA

GCTATCTGGGCGCGGATGAGTATGATGTCGATACCGCCAAATACCAAGAAAATGTGCTGGATACGCTGGATCGCTTGGGC

GTAAGTATCTTGTGGCGTGATAATAATTCGGACTCAAAAGGCGTGATGGATAAGCTGCCAAAAGCGCAATTTGCCGATTA

TAAATCCGCGACCAACAACGCCATCTGCAACACCAATCCTTATAACGAATGCCGCGATGTCGGTATGCTCGTTGGCTTAG

ATGACTTTGTCGCTGCCAATAACGGCAAAGATATGCTGATCATGCTGCACCAAATGGGCAATCACGGGCCTGCGTATTTT

AAGCGATATGATGAAAAGTTTGCCAAATTCACGCCAGTGTGTGAAGGTAATGAGCTTGCCAAGTGCGAACATCAGTTCTT

GATCAATGCTTATGACAATGCCTTGCTTGCCACCGATGATTTCATCGCTCAAAGTATCCAGTGGCTGCAGACGCACAGCA

ATGCCTATGATGTCTCAATGCTGTATGTCAGCGATCATGGCGAAAGTCTGGGTGAGAACGGTGTCTATCTACATGGTATG

CCAAATGCCTTTGCACCAAAAGAACAGCGCAGTGTGCCTGCATTTTTCTGGACGGATAAGCAAACTGGCATCACGCCAAT

GGCAACCGATACCGTCCTGACCCATGACGCGATCACGCCGACATTATTAAAGCTGTTTGATGTCACCGCGGACAAAGTCA

AAGACCGCACCGCATTCATCCGCTGA

>NG_065451.1 Escherichia coli QHXN2016-F200 mcr-1 gene for phosphoethanolamine--lipid A transferase MCR-1.21, complete CDS

ATGATGCAGCATACTTCTGTGTGGTACCGACGCTCGGTCAGTCCGTTTGTTCTTGTGGCGAGTGTTGCCGTTTTCTTGAC

CGCGACCGCCAATCTTACCTTTTTTGATAAAATCAGCCAAACCTATCCCATCGCGGACAATCTCGGCTTTGTGCTGACGA

TCGCTGTCGTGCTCTTTGGCGCGATGCTACTGATCACCACGCTGTTATCATCGTATCGCTATGTGCTAAAGCCTGTGTTG

ATTTTGCTATTAATCATGGGCGCGGTGACCAGTTATTTTACTGACACTTATGGCACGGTCTATGATACGACCATGCTCCA

AAATGCCCTACAGACCGACCAAGCCGAGACCAAGGATCTATTAAACGCAGCGTTTATCATGCGTATCATTGGTTTGGGTG

TGCTACCAAGTTTGCTTGTGGCTTTTGTTAAGGTGGATTATCCGACTTGGGGCAAGGGTTTGATGCGCCGATTGGGCTTG

ATCGTGGCAAGTCTTGCGCTGATTTTACTGCCTGTGGTGGCGTTCAGCAGTCATTATGCCAGTTTCTTTCGCGTGCATAA

GCCGCTGCGTAGCTATGTCAATCCGATCATGCCAATCTACTCGGTGGGTAAGCTTGCCAGTATTGAGTATAAAAAAGCCA

GTGCGCCAAAAGATACCATTTATCACGCCAAAGACGCGGTACAAGCAACCAAGCCTGATATGCGTAAGCCACGCCTAGTG

GTGTTCGTCGTCGGTGAGACGGCACGCGCCGATCATGTCAGCTTCAATGGCTATGAGCGCGATACTTTCCCACAGCTTGC

CAAGATCGATGGCGTGACCAATTTTAGCAATGTCACATCGTGCGGCACATCGACGGCGTATTCTGTGCCGTGTATGTTCA

GCTATCTGGGCGCGGATGAGTATGATGTCGATACCGCCAAATACCAAGAAAATGTGCTGGATACGCTGGATCGCTTGGGC

GTAAGTATCTTGTGGCGTGATAATAATTCGGACTCAAAAGGCGTGATGGATAAGCTGCCAAAAGCGCAATTTGCCGATTA

TAAATCCGCGACCAACAACGCCATCTGCAACACCAATCCTTATAACGAATGCCGCGATGTCGGTATGCTCGTTGGCTTAG

ATGACTTTGTCGCTGCCAATAACGGCAAAGATATGCTGATCATGCTGCACCAAATGGGCAATCACGGGCCTGCGTATTTT

AAGCGATATGATGAAAAGTTTGCCAAATTCACGTCAGTGTGTGAAGGTAATGAGCTTGCCAAGTGCGAACATCAGTCCTT

GATCAATGCTTATGACAATGCCTTGCTTGCCACCGATGATTTCATCGCTCAAAGTATCCAGTGGCTGCAGACGCACAGCA
ATGCCTATGATGTCTCAATGCTGTATGTCAGCGATCATGGCGAAAGTCTGGGTGAGAACGGTGTCTATCTACATGGTATG
CCAAATGCCTTTGCACCAAAAGAACAGCGCAGTGTGCCTGCATTTTTCTGGACGGATAAGCAAACTGGCATCACGCCAAT
GGCAACCGATACCGTCCTGACCCATGACGCGATCACGCCGACATTATTAAAGCTGTTTGATGTCACCGCGGACAAAGTCA
AAGACCGCACCGCATTCATCCGCTGA

>NG_065450.1 Escherichia coli mcr-1 gene for phosphoethanolamine--lipid A transferase MCR-1.20, complete CDS

ATGATGCAGCATACTTCTGTGTGGTACCGACGCTCGGTCAGTCCGTTTGTTCTTGTGGCGAGTGTTGCCGTTTTCTTGAC
CGCGACCGCCAATCTTACCTTTTTTGATAAAATCAGCCAAACCTATCCCATCGCGGACAATCTCGGCTTTGTGCTGACGA
TCGCTGTCGTGCTCTTTGGCGCGCTGCTACTGATCACCACGCTGTTATCATCGTATCGCTATGTGCTAAAGCCTGTGTTG
ATTTTGCTATTAATCATGGGCGCGGTGACCAGTTATTTTACTGACACTTATGGCACGGTCTATGATACGACCATGCTCCA
AAATGCCCTACAGACCGACCAAGCCGAGACCAAGGATCTATTAAACGCAGCGTTTATCATGCGTATCATTGGTTTGGGTG
TGCTACCAAGTTTGCTTGTGGCTTTTGTTAAGGTGGATTATCCGACTTGGGGCAAGGGTTTGATGCGCCGATTGGGCTTG
ATCGTGGCAAGTCTTGCGCTGATTTTACTGCCTGTGGTGGCGTTCAGCAGTCATTATGCCAGTTTCTTTCGCGTGCATAA
GCCGCTGCGTAGCTATGTCAATCCGATCATGCCAATCTACTCGGTGGGTAAGCTTGCCAGTATTGAGTATAAAAAAGCCA
GTGCGCCAAAAGATACCATTTATCACGCCAAAGACGCGGTACAAGCAACCAAGCCTGATATGCGTAAGCCACGCCTAGTG
GTGTTCGTCGTCGGTGAGACGGCACGCGCCGATCATGTCAGCTTCAATGGCTATGAGCGCGATACTTTCCCACAGCTTGC
CAAGATCGATGGCGTGACCAATTTTAGCAATGTCACATCGTGCGGCACATCGACGGCGTATTCTGTGCCGTGTATGTTCA
GCTATCTGGGCGCGGATGAGTATGATGTCGATACCGCCAAATACCAAGAAAATGTGCTGGATACGCTGGATCGCTTGGGC
GTAAGTATCTTGTGGCGTGATAATAATTCGGACTCAAAAGGCGTGATGGATAAGCTGCCAAAAGCGCAATTTGCCGATTA

TAAATCCGCGACCAACAACGCCATCTGCAACACCAATCCTTATAACGAATGCCGCGATGTCGGTATGCTCGTTGGCTTAG

ATGACTTTGTCGCTGCCAATAACGGCAAAGATATGCTGATCATGCTGCACCAAATGGGCAATCACGGGCCTGCGTATTTT

AAGCGATATGATGAAAAGTTTGCCAAATTCACGCCAGTGTGTGAAGGTAATGAGCTTGCCAAGTGCGAACATCAGTCCTT

GATCAATGCTTATGACAATGCCTTGCTTGCCACCGATGATTTCATCGCTCAAAGTATCCAGTGGCTGCAGACGCACAGCA

ATGCCTATGATGTCTCAATGCTGTATGTCAGCGATCATGGCGAAAGTCTGGGTGAGAACGGTGTCTATCTACATGGTATG

CCAAATGCCTTTGCACCAAAAGAACAGCGCAGTGTGCCTGCATTTTTCTGGACGGATAAGCAAACTGGCATCACGCCAAT

GGCAACCGATACCGTCCTGACCCATGACGCGATCACGCCGACATTATTAAAGCTGTTTGATGTCACCGCGGACAAAGTCA

AAGACCGCACCGCATTCATCCGCTGA

>MK965884.1 Escherichia coli strain CY307223 truncated MCR family phosphoethanolamine--lipid A transferase (mcr) gene, complete cds

ATGATGCAGCATACTTCTGTGTGGTACCGACGCTCGGTCAGTCCGTTTGTTCTTGTGGCGAGTGTTGCCGTTTTCTTGAC

CGCGACCGCCAATCTTACCTTTTTTGATAAAATCAGCCAAACCTATCCCATCGCGGACAATCTCGGCTTTGTGCTGACGA

TCGCTGTCGTGCTCTTTGGCGCGATGCTACTGATCACCACGCTGTTATCATCGTATCGCTATGTGCTAAAGCCTGTGTTG

ATTTTGCTATTAATCATGGGCGCGGTGACCAGTTATTTTACTGACACTTATGGCACGGTCTATGATACGACCATGCTCCA

AAATGCCCTACAGACCGACCAAGCCGAGACCAAGGATCTATTAAACGCAGCGTTTATCATGCGTATCATTGGTTTGGGTG

TGCTACCAAGTTTGCTTGTGGCTTTTGTTAAGGTGGATTATCCGACTTGGGGCAAGGGTTTGATGCGCCGATTGGGCTTG

ATCGTGGCAAGTCTTGCGCTGATTTTACTGCCTGTGGTGGCGTTCAGCAGTCATTATGCCAGTTTCTTTCGCGTGCATAA

GCCGCTGCGTAGCTATGTCAATCCGATCATGCCAATCTACTCGGTGGGTAAGCTTGCCAGTATTGAGTATAAAAAAGCCA

GTGCGCCAAAAGATACCATTTATCACGCCAAAGACGCGGTACAAGCAACCAAGCCTGATATGCGTAAGCCACGCCTAGTG

GTGTTCGTCGTCGGTGAGACGGCACGCGCCGATCATGTCAGCTTCAATGGCTATGAGCGCGATACTTTCCCACAGCTTGC

CAAGATCGATGGCGTGACCAATTTTAGCAATGTCACATCGTGCGGCACATCGACGGCGTATTCTGTGCCGTGTATGTTCA

GCTATCTGGGCGCGGATGAGTATGATGTCGATACCGCCAAATACCAAGAAAATGTGCTGGATACGCTGGATCGCTTGGGC

GTAAGTATCTTGTGGCGTGATAATAATTCGGACTCAAAAGGCGTGATGGATAAGCTGCCAAAAGCGCAATTTGCCGATTA

TAAATCCGCGACCAACAACGCCATCTGCAACACCAATCCTTATAACGAATGCCGCGATGTCGGTATGCTCGTTGGCTTAG

ATGACTTTGTCGCTGCCAATAACGGCAAAGATATGCTGATCATGCTGCACCAAATGGGCAATCACGGGCCTGCGTATTTT

AAGCGATATGATGAAAAGTTTGCCAAATTCACGCCAGTGTGTGAAGGTAATGAGCTTGCCAAGTGCGAACATCAGTCCTT

GATCAATGCTTATGACAATGCCTTGCTTGCCACCGATGATTTCATCGCTCAAAGTATCCAGTGACTGCAGACGCACAGCA

ATGCCTATGATGTCTCAATGCTGTATGTCAGCGATCATGGCGAAAGTCTGGGTGAGAACGGTGTCTATCTACATGGTATG

CCAAATGCCTTTGCACCAAAAGAACAGCGCAGTGTGCCTGCATTTTTCTGGACGGATAAGCAAACTGGCATCACGCCAAT

GGCAACCGATACCGTCCTGACCCATGACGCGATCACGCCGACATTATTAAAGCTGTTTGATGTCACCGCGGACAAAGTCA

AAGACCGCACCGCATTCATCCGCTGA

>MK965883.1 Escherichia coli strain QHXN2016-F200 phosphoethanolamine--lipid A transferase MCR-1.21 (mcr-1) gene, mcr-1.21 allele, complete cds

ATGATGCAGCATACTTCTGTGTGGTACCGACGCTCGGTCAGTCCGTTTGTTCTTGTGGCGAGTGTTGCCGTTTTCTTGAC

CGCGACCGCCAATCTTACCTTTTTTGATAAAATCAGCCAAACCTATCCCATCGCGGACAATCTCGGCTTTGTGCTGACGA

TCGCTGTCGTGCTCTTTGGCGCGATGCTACTGATCACCACGCTGTTATCATCGTATCGCTATGTGCTAAAGCCTGTGTTG

ATTTTGCTATTAATCATGGGCGCGGTGACCAGTTATTTTACTGACACTTATGGCACGGTCTATGATACGACCATGCTCCA

AAATGCCCTACAGACCGACCAAGCCGAGACCAAGGATCTATTAAACGCAGCGTTTATCATGCGTATCATTGGTTTGGGTG

TGCTACCAAGTTTGCTTGTGGCTTTTGTTAAGGTGGATTATCCGACTTGGGGCAAGGGTTTGATGCGCCGATTGGGCTTG

ATCGTGGCAAGTCTTGCGCTGATTTTACTGCCTGTGGTGGCGTTCAGCAGTCATTATGCCAGTTTCTTTCGCGTGCATAA

GCCGCTGCGTAGCTATGTCAATCCGATCATGCCAATCTACTCGGTGGGTAAGCTTGCCAGTATTGAGTATAAAAAAGCCA

GTGCGCCAAAAGATACCATTTATCACGCCAAAGACGCGGTACAAGCAACCAAGCCTGATATGCGTAAGCCACGCCTAGTG

GTGTTCGTCGTCGGTGAGACGGCACGCGCCGATCATGTCAGCTTCAATGGCTATGAGCGCGATACTTTCCCACAGCTTGC

CAAGATCGATGGCGTGACCAATTTTAGCAATGTCACATCGTGCGGCACATCGACGGCGTATTCTGTGCCGTGTATGTTCA

GCTATCTGGGCGCGGATGAGTATGATGTCGATACCGCCAAATACCAAGAAAATGTGCTGGATACGCTGGATCGCTTGGGC

GTAAGTATCTTGTGGCGTGATAATAATTCGGACTCAAAAGGCGTGATGGATAAGCTGCCAAAAGCGCAATTTGCCGATTA

TAAATCCGCGACCAACAACGCCATCTGCAACACCAATCCTTATAACGAATGCCGCGATGTCGGTATGCTCGTTGGCTTAG

ATGACTTTGTCGCTGCCAATAACGGCAAAGATATGCTGATCATGCTGCACCAAATGGGCAATCACGGGCCTGCGTATTTT

AAGCGATATGATGAAAAGTTTGCCAAATTCACGTCAGTGTGTGAAGGTAATGAGCTTGCCAAGTGCGAACATCAGTCCTT

GATCAATGCTTATGACAATGCCTTGCTTGCCACCGATGATTTCATCGCTCAAAGTATCCAGTGGCTGCAGACGCACAGCA

ATGCCTATGATGTCTCAATGCTGTATGTCAGCGATCATGGCGAAAGTCTGGGTGAGAACGGTGTCTATCTACATGGTATG

CCAAATGCCTTTGCACCAAAAGAACAGCGCAGTGTGCCTGCATTTTTCTGGACGGATAAGCAAACTGGCATCACGCCAAT

GGCAACCGATACCGTCCTGACCCATGACGCGATCACGCCGACATTATTAAAGCTGTTTGATGTCACCGCGGACAAAGTCA

AAGACCGCACCGCATTCATCCGCTGA

>MK754161.1 Escherichia coli strain CTX148 plasmid pCTX148 phosphoethanolamine--lipid A transferase MCR-1.1 (mcr-1) gene, mcr-1.1 allele, complete cds

GTCAACATAAGGGGATGAGTCCTTAATAAAACAGGTCCTCAGAGCATACATATTCTGCGGGGCGTGCGGCAACAACAGGA

CGAACCGGGGCGGTTTTCAATTTGCAGCCGCCAGGCTGCCGTGGTTCCTTTCGGTGCGAGAGAAAGGAAAACAGGTGTTA

TGGTATCGTGGACCATATACATGTTTGCCAGACAACCAAAAACCAGTACGGATGGATAAATCATCGACATAACAAGAGTC

ATTGTCATTTCAGAACGACTTCTGACAATTTTTATGGCCTGAACTAACCCTCCCGCAAGGTCGCCGCGAATTTCACCGCT

GGATATTAATTGATATTCATCCTCCTTTATATATTTTTTTAATGCTGAAGATATTGCCCCTGTGTTATTCAGTGCATTAA

GGCACTCACAACAAATATAGTAGGTTGCAGAAGACCCCGTTTTCTTCCCGTAGTCTGTTTCTACATCAATCAGGTGTGTA

AGAGCACGCTTTACATTTATGCCATCATTTAACATTCCAGATAAATCTTCATATAAACTTATTCTGGAATACCTGAATGT

GTATTTTGCCAGCCAGTGGGATATTCCAGAGAAATCAATTTCTATCGAATTGAGTTTCTCAAATATATCTTTTATCGCCA

TGTCCCCTCCGGGAGCATTGAGTATTTATCTTCGTCAATGGGGGATATGAAGTGTGCGGACAGCGGATCTACCAGGCCAT

GATTAATCAGGTGTAATAAGTGCTGATTACGGGTAATTCCTCCAAGTTCATAGTGCCAGTATGATTTTGCTTCCAGACGC

CCGGACTGACGATATAACTGAAAAAAACGTGCGTCGGGTGAGACGACTTCTGCTATAACCGTTCTTCCCGTTACGCCTTT

ATAACAGTGCTCGCAACCGTCAAGATTGCGCAACCGGACCTGTTCCGGCAGGCAATTTTCCTCAATGAGTTTCCGTTCTT

CTGTACTCAGTTTGTCTGCCATTTCAGCATAAGTGCGCTTACAGTAAGGACAGAGTTTTTGTACCAGCCGTTGACTCAGC

AGACCGATAAACAGAAGAGGATCTGCAATTAGCCTGGCCTGAATATTCTCCATTTCAAGGCGCTCAATAATATTCAGGGC

ATCGTTGGCGTGTAAGGTCGTCAACAGAAGGTGGCCTGTCATCGAGGCTTTGATTGCTGCTAATGCCGATGCGTGATCAC

GAATTTCGCCGTTCAGTATCGCGTCTGGATCAAGTCGCATAGCGGATTTAATCGCGTCAACCCAGCCATCGACGGAATCC

CTGACTGCCGTCTGTATTGCGCCGGGAATGCGGCCCTCCGGCGGTGATTCAATGGTGAAAAGGCGTTTTCGGGGGAGACG

CATATTATCGTTATGGTTGAAACCAAACGTGCTCAGATAGGCTTCTGAAGCCGTTCTCAGAGTCGTTGACTTACCGGACC

CTGTAGGGCCGGATAAAACGATAATTCCTTCCGGGCGTTGTAACATTCGCCTGATTGTCTGGATTTGTTGTGGCATGTAG

CCAAGTTCTTCGAAAGTGGGAATATCCTCGCTGTCATCTTTAATCAGGCGGAAGACCGCGTACACACCACCCACGCACGG

ATAATGCGAATAGCGCGCCCCAAAAAGATTTACAGGCTTCAGAAAATTCTCTGCGATACGTGCATCCTGCGGTATTGCCG

GGTCAAACTGCGTACCTTTGATAACATCGCTCATTCCAGAATAGGCAGCACCCAGCAATTCCAGCCCTTCTTTACGCGGG

ATGACATCCAGAATTTCCAGTTCCCCATGCACGCGTGCTTCGACATAAGTGAACTCGGAACCGTCCCGACCAGGTGTGAT

GTGCAAATCGGAACTTCCCAGGTTGTTTGCTTTCGCCAGGTAGCTAATGACCTTTTCCTGGTTGCTGTTTAAGTGTTGTG

ATGAGTTTGAATCAGACCTTCTTTCGCTGTCCTGGTAGCGGGATGCGATAACATCAAGAGTGGTCCAGGTTATATTTACC

TTTCCGGGATATTTAACATTCAGAGTGCCAATATAAGCCTGAACAGCACGCTGTCCTTTACTGTTATCAGCGATATAAAC

ATGAACAGGTTTATCGTCTTTTTCAGATATACTGACGTATACAACATCAGAAATATCTTTTCCGGGATAGTCTGCGTCAG

AAAAAAACATTATGTGTCGTCTCTTTCAATAGCATTAAAGAATAAGCTGGCGTTTCTCTGTACCATTCTGAATCCAGACG

CCATTACCGGTGATTGATGCCACCTTATAAGAAGTACCAGGAATACTTTGCCCGACCTTGACATCAGTTACTGACCCGTC

CAGGAGAGAAATTCGTGCTGACATTTGTTGTGGTGTACCAAATATTTCAATAACACGAAGTGATTTAGCGGCTACACGTT

GCGCTGGTATATCGGTATCTGCAAGAGAAGAATACCCGGAAGAGGGCGTTATTGCTGGCGTTGCGGACGCTACACCATTC

AAATCAAGATTACGCAATTCACTGACAGCCTTCTGACGCGCCAGCTTTGCTTCGTAAATTACCGTTTCAGCCTGAATCTG

CTCTACCTGTAAAATGGTCGGAGATGAATATTTTATTTCGTCTTTTGGCGTGCTTTCAGATTCTGTTGCGTAAGCAAGTG

AACTGAAAAAACAGACAGAAATAATCGTTGGTTTAATTATTTTTAACATAGAATGCACCTTTAATCTCATAATTCAGTCT

TCCTTCTTCCAGTTTTATTCCTGCATACTGGAAGCGAACGGCAGGTTCATGAAAGTTAGCGAAAAGTATTGAAGGCGGGG

TGCTGGTCTGAATCATTAATTCATACTCGTTCCACGGGAGAATTATTGGACGGCCTTCTTCATCTGTTTTTGTGTTTTCA

ATTTCCTGCCAGGTGAGTTTTAACCTCATTTTTTGTGCAAATGTTGTCAGGCGTTCCTGAATATCTGTTGCCTGTGGCAA

TGTGTCCGGGGTAATTGGATCAGGTGAAACGTCAAAACTGACAGGAAGAGAAAATCCCCCGGCGCTGCCTTCTGGCAATA

CAAAAGTTGCTGTGGTTGTCCCCTGGAATATTTCTCGTATCCGTGTGCTGAAGTCCTCGACCGTAACGCCGCTCAGTTCC

TTATATGATGTGCGTAACAGCCCGTCATTACCGGATGTGCTGCATTCAGCAAGGTCAAAACGCCATCCGGCGATCGATAA

GGGCAGTGCATCCCATTTATCAATGCATTTATCAATAAATGGTTTTATTTCAGGTAAATGTTGCCACGGTGGGGCAATCT

GTATTGCCTGTTTGTCTGCCATTTCTTTTGCCAGACGAAGACGTGCAGCTTCTGCCGCAGCTTCTTTTTCCCGGTATTCC

TGGTACATGGTGATCCCGTTCCAGAGCAGTATGGCCAGAATGGCACTTCCGCCATAAATCATGAACTGACGTTTTCGGGA

TACGCGAGTAAACGCCGCCTCTTTCGGCGGTTTTTTTACGTCAATCAACGCAGACAGAGTGAGTGAGGGGAGTGCCTGGC

TGATATCCCATGATTCAGGCTGGTATAGTTTCCAGCCGGGTTCTGGTGTTTCATTAAACTCAAGAAATGTTGTCAGAGCA

CTCATGATCTGACTTTTGTTACCTACAACATCGTTGACGAGAACGTTATTGACGCAGGAAACAAAGCCATAAAGATCACC

GAGTTCGCAGATAGCGTATCCGTCAGGTTCGATGAGGGGTTTTATCATCAGTGCCAGTGACCAGAATGAGGACGCCCCTG

TGGGAAGATCCTCTTTTCTGATAAATGCCACCATCGTTTTGGTTTCATTTTCTGAATCCTGATACTGACAGGAGAGAAAA

TATTCAGCGTCGTTTTTTCTTGCGAATGTACGCGCATTCTTTTCTTCTGAAGGAAGCGTTTTCCAGTCCAGACCGGAGAA

AAAAGCACGCTTGTTTTTAATAACCGAAGTGAAATTCCGGTTATTCTGATTATTCTCAGACAAAGTGACCTCCAAAAAAA

CTCCCTGTGTTTTTTCAGGGAGTTTTCAGTTAATTAGTTCACATTTTTTATTACTGCACTGGAGCCGGAATTAAGCACAT

TTGGTGTAATCAAAACGACCAGAATCGTGTCGCCGTCTTCGCTGTTAATGCCACCCCCAAGTCCAAAGAAGGAGGGGGTT

ATTACACCTTGCTTACTGGTTTTCCGGTTTGATTGCTGGAATCCACTCAGAATAACTGTCTGGCCTGATTTCAGGTCAAT

AGTCTGATTAATTGTTTTCAAATCCACATTCGGTGTTTGTGCTTTACTGCCGCCACTTTCGAACACCTCAAAAGTAGGTT

TGTCAGACAGACTCATTGAGAACAGCAATTGCAGGTTTTGTGAATCCGGCTGAATGTATGGAAGCAGAGTCATATTGAAT

CCGGTCGTTATTGTTGCCGCATTCAGTGACGTGGATGATCCCACATTGGCGGTGGAATCGGTTGTGACACTTTCAATGAA

GCCCTGCTGATTGGCAATTTGCATAGGGACGGGCGTCAGGTTTTTAGTCACAGCGGAGTTTTGCGTTACGACACTGACGC

TGCCTTGTGACGACAGGGCTTTCAGAAATGCTTTTGAGCCAGCCAGCTTACCGTCCACGATGGATACACCACTGGTAATA

ACATTCTCGGCGGCATTACCAAATGAGCCACCAAGACTCAGACCAAGATGACCATCATTAAATACTGCGTTCCAGTCGAT

TCCCGCCTGTTCCTGGCTTGATTTCTTGATACTCAGGATTTCAACGTTCAGCACAACCTGACGACTCATTTCATTATTGC

GCTTGTTCACAATCTCCTGAACGCTGTCCAGTACATCCGGGGTATCTGTTACTGTCAGTGAACCAGTGGACAGATACATG

CGCCCTGTGCCTGGTGTCAGCATTGAGGAAACTTCGCTTTTCAGGTCGTTGTACAGCGATGATTTCATTTCCACGGTTGT

AGTTTGCGTATTTGATGCGTCGCCAGTCATACCGCCAGAGCTGGTAGAGCCGGTACTGCCGCTGGAAGACATTGTGCCGC

TCACAACTTTTGAGTTGTATGCCACATTGCTGTCCATATATGTGATCGGGAATGAGCGCGTTTCCAGATAGAAGAATGTG

ATACGACCTTTATCGTATTTCCAGGATATTCCCAGCCGTGAGGATACGGTTTGCAGTAGGGCAGGAAGCCCGGAGATATT

CAGTTCCGGGAGTGATGTACCGCCAGAAGAGACTTTTACTGGCTGACTGCCTACTGACGCCAGAGGAACCATTCCATTTG

CATCCGGTGCTGGAATTGATCCTGTCATTTGTTGTGTTGAGCCAGTGCCTGTACCGTTCAGATAGGTCCAGACATCGGCG

CTGATTCGTACCGGGATATGACAGGTTTGTGTGATGCGTTGTGCGATCTGGCGTAGCTCAATATCTGAACGAGTATTAAT

CACAATCGGGCAGGGCGGCGCTGATTGCTTTACCTGCTTTTCAATAATTGGCGTTGTATTAATCCATTGTTTTGTTGAGT

CCTGGATAACCGGGTTATTACGAATTTTTTTCGCGTATTCAGTCGCTTTTTGTGCGTCTTTTTCGGCTGCAAGTTCATTA

GTATTGATTTTTTTGAAGTTTGCACAGCCTGATAATAAAAAAGAGGATATTAATAAACAAAGCAATTTTTTATTCATATT

TAATTAACTCCGGTACTGGAATGATAACTGTGCATCCTATTTCATCGTGAGAGCGGGTTAACGATATAACTGTGTCGAAA

AGGTTTTGTATCTGCACGCCGCCATCTGAAGGAGTCGTGACAACAATAAGACGTTTGCCACGTTTCCTGGCTGTTTTAAT

AAATCGAACAATAAACTGAACAACTTTTGATTCCCGTAAGTTGTTACCGATGGCTACACTATTAAGAACCAACGTTTTTT

CAGGTGCAGATAAAAAGTCAAAATAATCAAAATTTTCATCGCAAAGATAATGGTGTTTTAGAAGGGGAAGGTGTTCATGG

CGTTTATAAATATCACAGAAATCAAAATATTTAACGTCAGTGTCATTGATTAGCAATTCTGCTAAAAGTGTTGATTTTCC

AGTTCCAGTTTCGCCTATAATCAGAATATGTTTTTCAGAGTTTATCAAAACATCAATGGCTTCTTTCATCATATTCATAT

TCATATTCATATTCATATTTATCATATTCGAGTGTTTTATTCTCATAATAGAATCCTGTCTTTGCTAATCAATAGTATCT

TTATTGCGAGGATTATTCAGATACGTCTGTTTTATCGTGATTATCAAGGTCTGGATTAGTAATCACATTGCCTCCGGCAA

CCATGACTTTTTTTCCTCCAGCTTTAGGGAGATAAATTTCTGCAATTTTTGCTATTTCATCCATGACTTCTTGTGTAAAC

AGGTTTGGAATGTCAACATGATTGTTACGGGCGATATCCACGCGGCGTTTAAACTCTGGTTCTTCAAACCAGATTATTTT

GTTGGCAATAAATGGTCGGCTAAATTCACTGGTTATAATTTCACGGATTGCAATATTTCTGGCTTTATTCTTCTTATCCC

GGAGAGACACAATTTCTTCAGGAAGAAGAACCGGGCGCTCTATTACATCATCATTCCGTGTACGTGAACTTCCTCCCTTA

CCACCACCACTGGATATTGAGCGTTTGGATATTTTCATATCACGCACCCCAATCTCTTCTGAAATCTTTTTAGCCAGAGC

ATTTTTTGATTTCGGCGGATAGTAAAGCACAACAGCACTGTTTTCAGTGAATGTGGTCCATCCTTCCTGACCGTACATAT

CGCTTTTTTGCCCTTGTCCTTCATTCTGAAGAATGAACATAAAGCGAAGGTTATAACCTGCTGTAAAACCAACAGCGCGT

TCAATGACCTCCGATTTACCCATTGATGTAAATTCATCCAGGAGAATAAGGCACTGGTATTTCAGGTCCGGGTTATGTTC

CGGCAGTTCACGACAGTTTTCATTAACCAGTAATGAGAAGAAAAGGTTTACAATTTTCTCATGAGTGATTAAGGCATCCG

GTGTCAGACCAAGATAAATTGACATGGGTTTTTTACGAATATCCCGGATATCAAAATCACTGAAATTCGTCGCTTCAGCA

GTAACAGGGTTGCTGAATATATTCAGTGGTGATGAAAAGTTCGTTTTTATACTTCCCCTTGTGCGATCTGGTGCTGACAT

GAACTCAAAGAAGAATGATTTTGTTTTATCGCTAATCCAGCTACGATTTTCAACTTCCTGTCCCATCCATGCGGCGAGAT

CTTTTCCATTATCGGGAATCGACGTTTTCAGTATGGCGCTGATGGAAACAAGGACGTCCGGTGCATTATGACCTTTGGCT

TTCTGGTCCAGGTGAAAACGCTCCTTATCCAGCAGGTAAAGCCCTAACCCGACAAAAAGGTTGCGTGCAGAATCAGACCA

TATAGGATCATCACTTGCCGGGATAAGGATTGCGGCAATTTTTGCCAGGTCCGTTTCACGAAGTAAATCACTGCGACTGA

CGCAATCCAGAGGATTCCAGCGGTGAGAGCGTATCTGTCCTTTAATTGCCTGGTCGATAGTTTCCGCGTATCCGGCGGGC

GCAAAGAGAAAGCATTTTTGCCCTAATTCTTTTTGCCTGAATCCGGCTGATAAAAACCAGTTTTCAAGTTTGATATCCAG

TATGACCATACTTCCTGGATAGTTTACGCAGTTGGGGATAACAATCCCCACCCCTTTACCAGAACGTGTCGGGGCATAAA

GAATTAAAAATTGTTGTCCGGCGAAATAAATAAATTGTTTTTTGTATCTCCCCTTGAACATTTTACCGATGAGTATTGGT

GGATGTTTGTAAGGTGATTTTTTATCCGGGAAAAAACCTGATTTACTCAGGTCCATATCAGTTGCGAGCCGGGCGTCACC

GTAGATGACTTTTTTAGGCATAATCCCAATCACGACAATTAACATGAATATGGCAAAGAATATTAATGGAACGCTGAATC

CAAACCATGCCATAAAAACGAGTTTTTTATATTGTGGGTTGCCAGAAAATGTTGAGATTACAGAATGGAATGTATCCCAC

TTGAGCAAAGACATATCAACGGATGAATATCTTAATGCTGTGTATCCACCGAGATAGTTTGAGGCTATAAGCACACCCAC

AAGAAGCAGTAACAGGAAGAGAATTAACCCCCCCATCTTTTTTGCATTCATAGTTATCTCCACAATTTACATTGTGCTCA

AAAAACGATTTTTTTCTTCGGGTTCGTAATACAGTTCAGTCATTCTGGTATGGTTGAAAAAAAGAATGACATCAATTGAT

GTTTTTATCGTGCGCATGATGAGGTCCATATCAACAGTCATACCAACATCAGATTGTTTTACAATACTTGCCAGTCGTGA

AAATGAGGCATAAGTATTATTAGCATGAATGGTACTGATTGAGCCTTCATGCCCTGTATTAAGCGCCTCCAGATAACTCC

ATGCTTCGTTACCCCTAAGTTCAGCAAGAAAGATGTGATCTGGCTTTAATCGCATACACGCTTCAATGATTTCCTTCGCT

TCAACGGTATTTTTTTTGTAGAAAAGCCGGATATGGTTTGGATGCAGCGGCAGAGACATTTCCGGTACATCTTCAACGGT

TATATAACGCCGTTCAGGTGGAAAGATATCTGCAATTGCCTTTGCTATAGTTGTCTTACCGGAGCCAGTCCCACCGACGA

TTAAAAAATTAAGACGGTCTTTAACTGCCTCTCTTAAAAATCGAGCTTTAGATTGACCATCTGGACGCCGGGATAATTCA

TACAGGTAGCGTTGCCGTTCAGTCAGGATGGCTTCGTGTTTCGTCGCAATCCTGACATTATCAAAACGCCCTGTTCGGAC

ATAATCATCTATAGTAAAGCGCGTTAATGATGGCTTACGGATAGATATCACAACGCTGTTATTTTCTGTTGCAGGTGGAA

TGATGATTTGTCCACGTTCCCCACCGGGAAGAACTACCGATGCAATGGGATTATCATGGCTAAGTGGTATTTTTATCTTT

GATAAGTTCGTCAGAGTTTTGGCAAGTGTCATTAAATTATCAAAAGTCGCGTCTGGCGCATCTTTAGATTCCCAGCCATT

TTTCCCTTCAAACCATATTGTTCCTGGCTTATTCACAGATATTTCTGTGACATGTTCAATATTAAGAACAGACTGGATAC

CCGTCATATCCAGATATCGTCTTGCGGTTTTACTGTTATCTCGTGGTTGTGATGTATTACTGGTATCTCTGATGTTATTC

ATATCAATTGCTTTACTGAATTACTGAAGTTCATATACGGATGAAAAGTCGATATTTCGCGGAACAATAACAGACAATAC

AGTCCCCTGATTAATATATGCCGTGGGGGAATATTTATCGTATTGTCCAGCGTTGTTTTTGCCATTTCTTTTGTTGCGT

CAGATGTGTTTTCGTAGGTGATATTGCTGTTTGAGCCTGTTTGTTGCGTGCTGTTTTTCAGAATATCTAAACCATCACCA

AGTAATGACAGCAGCAATGCACCACCAAAACGCTGACCAAAATGATTATCGACCCATGCAGGAAGGCCGGAAGCACCCAG

ACTGTCAGTGCCAAGTGCGCCAATGCGCACATTGACATTTTCATCTTTGAGCGTTGTCCAGTTAACGAACACACGGGCTA

CTCCCTGTGTCATGACTTTATTCTGCTCCCCGATCAGTAATGCTCCTTTACGGGCAAGAAGCACTTCGCCGTTATCCGAC

CAGACGTCTCGTGTTAACTGACACATTGTGATACCGGGATAAGAGGTTACGATTTTCGTTTTGAGCACACAGGATAATGC

TGTTCCCGCAGATAACAGATATCGGCGGTTCAGCACAGGGGATACCTTTCCGTCTGCGTACTGACTGCCCTGTAAATCAT

CTCTTTCCTGGTTTTTTTCGCCTTGTTCCTGATTGTCGATCTTCACCATTGTGTCGCCCATCAGTTGTCGGACGGATTTT

GGCAATGGTTGTGGTTGGTTTGGATCTCCACTGGCGGTTTGGGGAGTGCCGTTGGCTATCACATTGACGGCTGTTTGATC

GGCCTTTTGTGATGCTGGCGCTGACGGGGCATTGTCTGCCTGTTTCTGTTCTTGCTCCTCCTGTGCTTTCCTGCGGTTAG

CGGCATCGAGTTTTTCCTTTTCTTCGATGTTTTTCATCATTGTTGTCAGTGAAACGCCATTGTTGGTATTTGTGACCAGT

GTCTCGTCAGACTTCTCAAGAGGTGTTTGCTGAACGACCTGTTCTCGTGAAAGAACTTTAAACAAGATAATGATCACAAA

TAACGCGGCAATCAGTAATATGATGAGGATTAATATTTTTTTTGGTGCTGCTTTACCTTTTACTTCGATAATTCCGCGAT

TTCCCGCGTCATTATTTTTTTCATCATCTTTATTTTTCATAGCTGAACTTCTCTACTCGTGTTTTTCCGTTTGACGTACC

TTTTTTATTCCACGGACGATTAACGATTGATGATGTTCTGATATCTGCAACCTGATCACCCAGGCGCAAACGGAGATTTT

CAGCCACGTCATAGTAAACCATTGTGTTTTTATTTCTGTCTCCGTTAACCAGATGTTCATTTCCATCGGCATCAACACGA

TAAAGGACCGGAATTTCCTGTTTATTAGTCCATCTCATACATGTAAATTCGCCATTATCCCATACCTGATAAGGAAAAT

TGATTTGTCCCCCTTGAGTTGATAATGACCATTAATTATTCCACCATCAGTGCATGGAAATGGCTTTTCAGTCATTACGG

TAGCTGACGGTGGTTTCGGATAATTAAAACGCAGAATATACGTGGGTTGTTTCGTGGTTGATATCAGGTAAAGAGGATAT

ATATGCTTATTGGTCTGAACGGTAACGTTTGTATCCGGTTCTTCTGCTTTTGGACGCAGAAACAGAGTGTTGTCCCTGAC

CGATACTGACCAGGCTTCTGGATCACCAATTCCGACATCTTTTATTGTTTCGTCTTGTCCGAACTTAATTGTTGTTTGTG

CGCCTGCTTTTACGCGCACATTAAATACATCCTGCTCGTTATAATCTACGGTCTGAATACGCCCGTCTCGTTCCGATGGT

GTGCCGTACATAGCTGCTAAAGCACCAGGTACTTGTAGCATCGACAAAAGAACTATCGCAGTAGTGCGTTTAAGCATCAT

TTTATAACCTCTGGATCAGGTTGGTAGCTCAGAACTTGCAGTCCCAGCGGATTCACAAGGCGCTCTTTTTCTGTTTTGAT

ATCTTTATCCCAGTCAAAAGAAATCGTCGAAATCCACTCTGTTGCTCTGTATCCGGGGGCGGGTTTACCAGATAAATCCA

GTACCATTTTTTTGAAACGCACCTGCGCCATATCCTTCCTTAGAAGGATTACTGAATTAATCTGGACCTTAATTTTGTAG

TTGTTTTTCAGAATGTTCAGTGGTGAGGAATCAGCACGAATCATGGTGTCATAAGCTGAAAACACCTTTTGGGAAGACAT

TGTTTTTACTGTATCGGCCTGTTCCTGGATAGTCTGCCAGTCGTATGCTTCATAGTTAATGAGATATTTTGACAGGAAAT

ATTTAGTCTCAACATCCTGATAACTTTCCTTAGCATCAGATAATTGAGGGGCAATATCAGTATATCCAGTGCTGTTATCC

ACTCGCACAAGGAACGGTACAGCGGTTTTTAATGGAGCAAGGGAGGCTATTGCGAAACCAAGAATGCAAATTACAACAAA

TTCACCAATGGCGAGAATGGTAAATGTTTTTGCCTTTTTCTTTATGATTTCAATTTCACTACGCTCAAAGTCACGTATGG

CCTTGAAATAATCCTTTTTTTCGTTCTTTTCTTCAAATTTCTTTTTGGAGGCTTCTTTTTTTGAATGTTCATAATAGTTT

GTGTTTTCCATTAATAATCACCTGCTTTGATCTGTTCAATTGCTGTGTTTAACTCAAACCATCCCCCTTTTTTCTGCACA

GGATTGGGGGTGTGAGAACATGCACTAATTAATAGCGCGGAGGCAATTATGAGGGGGATTTTCATATTGTTTTCCGTTCT

GTATAAAACAGAGAAATTAACCGTTCAGGCGTTTCAGGAGTTCTGGTATCCAGATATCAGGAGCTACATCTCCTTTTTCA

GCAATAATCTCATCAAAAATAGACAGGCCAGTTTCAGAGCCAGAAAGCACGGGGATAAACTCATCGAATCCAAACAAATC

CATTTTGGCGAAAACAGAAGAGCCAGATTGCTTGATGAGCATGGTCCTTGACTCCTTCGTCAGTTCTTTGAGTTTTTCAA

ACTCTTTTTCAGTAAGCCCAATTTCTTTATATCCTTCCCATTTTGCATCGGGATTGGGCAGGAGAATTTTCGTTGCGGTT

TGTTGTACCAGCGCCGCAAAAATTGCGCAGTTTATGGCGTCTTCCGGGGACTGTGATGTTAACCACATCATTTCACCTTT

CATGCGCCCCGCTTTCAGTGCGCTTTTAATCATGCTTTGTGTCATGGGAAAGTTTGCTGGCATCCAGAACTCTTCAATAA

TGGAGAGCATCAGATTGCCGCCGCGTTGCATGATTTCTTTGTAGAAAAACAGGACCGCGAGTAAAGGTTCACAGGCAGGA

TGAATACCGTTTTTTGTGTCCAGGACAACGGTTGTATCAAATCCCACTTTTTTATGATGCAGTGGATTGAAGGTATTCCG

GGGGGAATCCACCGCCCAGGCATATTCGCCGTTGTCGCACCATTTGGCGAGACGCGTCTGAAGTTCAGGACTGACTATAT

CGAGAAGAAGGGCTGTCCGGCGTTCTTCGACAGGCATTCGCATAATGCTTTCCACGGCAGCGTTTAATTCAATGCCATGT

TCATCAGAACAGGGGTTGCCCTGATTGTCTCGTGCCAGAACCTGTGTCCAGCGTTTCAGAAATGCGAGAAGTCTGTGCCA

GACGGGAGATTCCGGGCCTTCTGCCAGTTGCCACGGATTGCAGCCTGTATAGATACCTTCCTGCAACGTGAAATAGCTCC

CTCCGTATGCGCGGACAAAAAGTTCGGTAGAACGGTTGTAATCCACAACAAACATTAAAGGGTCAAAGCGTTGAAGAAAG

CCGCTGGCTGTTGCTTCAAATGTTGTTTTCCCTGTGCCTGTTGCGCCAAGAAACATTGCGTGTCCGGCAATTTTTTGTCC

GGTAACATTTTTTTCCGGTGGGCTGTAATGTGTATTGAACCAGTAAACGCTGCCTGAATCCGTTTTTAAAGGCATTATGG

CGGAGCCATCACCAATAGGATTGCCTGATTTTTTCCCGGAGGAATAATTGTGAAAAGACATCAGACAGGCCAGGTTGGTA

ATTGTTCTTCGGGTATCCAGTGGACGCCTCTTATTCAGCGGCATATGACTGAAAAAAACAAAAGGTGATGCGAGTGAGGC

GCGACTGAAGCGGAATCCCTTACCCGAAGTAATAAACTCAGCAGAGAGCTTTATACCATTGCTTCTGGCCTGATCTGGTG

TATCACCGAATACCGTTAATGCGCAGTGTAGTGAACCAAACAACGTAATTCCGGCTGCGACCGCTTCTTTACCAGCTTCG

AGTTCTTCCTGCTGTATTTTGGCTGCATCATTAGCTGATTGTAGTTTGTTTAACTGTGAATCGATATTCTTCAGGGTTTT

TGTTGGTGACTCAAAAATAAAGGATTGAGTAAGAATAAATTCATAGGGTTGTTTGAGAAGGAAATCCCATTGTCCGGGGG

TTGTTTCTATCGGGAAATCCTTCAAAATATAATTCGTTGCGAATTTTTTACTATCCGATTCGTTGTTACGCAGTTCAAGA

ACATCAGCACCAAAATACCATTCAGAATCACAAATGCTGGATGATAAGGGGGTGGATGATAAAGGAATCATGTTGTGTTC

TGCATTTAGCAATAGTGATAGATAGTCTGCCACCTCACTAATATAAGTATTGTAAACGGATAATAAAGAAGCGTTAAATG

GTAGAAGTGCTTTTTGCGCCTGTTGTGTCATTTCATTCATGCGCTCGATTCCGGTATCAACATCATCGTAGGGTATCCCA

AATGTCAGATACCATGTTGATTTATAGAATGCGCTGGATGTGAACAGAGCAAGGTATTTTTTTGAAAACCTGCTTAGAAA

ATCATTGTCAAAATTCCATTCTCCATTGATGGTTGCTCTTTTCTTGATAAGATGAGTCCATAAATACAGGTCGCCTTCCT

TTCCTAACCCAATCAGGAAGTTTTTAACGCTGTTAAATAAATTTGTAAGAACATTATCGTTCTCTGTCTCAAATGGAATG

CCTGCTACAACAAGTGAAGCCAGCATTTTCCTGTCAGAAGTAAAGATAATACTGTCTGTTATATGGAAGCGATAATCGGG

ATATTCACGAGCAACATCCATATTGTCACTGTTTTTTAATTTGATTTTGTGCATATATGTTACCTGACAGATGAATAACC

AATAATTAAATCTCTGTGGCGGATTTTTAACAAAAGCCCCATAAGGTTTAAACGGATGACTCTTAGTGCGTTTGAGTCAT

TTTCACAGGCCAGTTTTACTATAAAAAGAAACAGCGCACATATGACAGGAATAATTATCCCTTTTATACCCCAAAGGAAA

ATAGCGGGGAAGCCTGTCAGAACAGCGAAAAAAGCCGTGAACAATAACAACATTAAGGGTATACCAGCAATTAACGCAG
G

ACGGTTAAACCCGTTATAAGTCATGAATGACTCACTGACATGTTCATTTACTTTGTCCATAACTCACCATGAAAAAAGCC

GCAATTCACACATAAGGTAAATATGCGGCACAGTTGTATATATGGATTACTTATTTGAAGAGTTCCAGCGCCCAGTTCCC

GGCGTAAAGTGCACCGCCAGCGAGGGAACAATAGACAAGCGCCATTCCTACATCAGACCAGCTTTTCTTCTCCATGAATG

CCATGATGACGTTATAGATCAGGTAACACAAAGCACAAACACCACCAAAGCTAAATGCCCATGTCTTAAGTTCTGTCATG

GTATTTGTTGCGGTGTCGAGTCCACCTGCATAGGCAACGGAGGCAGTTAGTGACAGGAAAAACGCCAGAATAAAATACTG

CAAAGTTGATTTGACTTTCATTAACTAAAATCTCCAAAAACATCCCAGTCCGGTGATGTTTTCTCTTCATCGTTCGGGGG

GGTTATTTCTGATTCATCAGTTTTTTCACGGATTGATGGCACGATATAGTTATTCGCGTTACGAATAACCCGTTCCAGAT

AAGAAGTGTTGTTAAATTTCTTTTCTTTTATAAATCCTGTTGATTCGTTACCCGAATAATACAAAGATAAAGCGCGGAGA

ATTTTTTGATTACTACTACCCTCCTTTTGTCTCAGATAATTGTCCGTCAGGACTGCCGCTCCTGCTTTTATATTTGTACA

CGGGTCAAAAACTGTTTTATTAGTCAGATTGAGTGATGGGAAATTTTTGCTGTATATCTGTGTCAGACCTGCACTAAACC

TTTTATTTTCTGCTGTTAATTTTTCTGCATATTCAATAGCTCCTTTTTCATCTTTAAAATACTTACTTGTTCCGTCAGAA

ACATTCGCTATTACATATGGATTAGCCCCTGATTCAGTCATAATCAGGGCATTCATCGTATCGGGTGATACATCAGGGGC

ACACTGACTTATCAGTGCTGCCAGAACAGCCGCTGATAACTGCATAAGGGATTTATCTCCTGATATTAAATTCGTGAAGA

TCGTTTTTTCCTGCGGGGGCGACGAACGATACTGTCCAGCTCATTAGCGCCGGAAAGAAGTTCTTTCAGCGAGACAATTT

TTTCCCTGACAGGTATAAGGGATTTTGCCCCCGGACATTGTTCAAAGTCTGATTTGTACAGGTCGTATAAAACAAGCGTA

TGTTTTACATCCTGTGACAGATAATTATGTTTATTCTGGATGTTACCTTGTTCGATATTTTTGATAACATCTGCTGAACG

ATGAATTAAATCTGTGTAGATTCGGGTGTTTTTAAGAGGATTTATTTTTATATATTCAGAAGCAAGCTGAATAAGATTGC

ATCGTAAGATAAGGACTTCACGAGTGGCAATATCAAGCTCGCTGACATCATCATTATCCTGCATGGGTATCTCCGGTAAA

GATTTTCTGGACTGAAGTCCGCGCCGTAGCGATATATCCGGCCCACATAATGATGTGTCGGATATGCAGACTCCAGTTCA

GAATCCCCTTGTCAGGGGATATTTAGTTAATGAGTACAATAGCCTCTTCTGGAATGGAGGAAGGAACGGGAACGCCCATA

TCATTCCCCTGATTATCGACAATCTTCTTATTTTTTACTTTTCCTAACATTGCTGAATGTTTTGTTTGCTGCTGCAAAGC

AGACATCAGACCTTCTGTATCGGCAGACCAGATATAAACTCGCTTACCGTAAATAATATGATGAATATCATAAACGGGTG

TTATACCGATTTGCTCAGATGTTAACGTTCCTCCTGAATTACGTTTGTCTGGATATTTATAAAGATAGTCATTGATAACA

TTTGCGAAATGGACAAATTCAATTGCTTCCTGTTTTGCAGGAAGCAACTTCATTCGCTGTATGGTTTGGTTATTGTTATC

TGTCAGATAGTAACTGCCTATTGACAGGAATATCAGGAGCAAGCTGAAGAACCAACCCATAGCGATTACTCCTGTTGTGT

GTCTTTAATTGTGATTACGCATTGTGCAATATTCCTGAATGCGTATAACGGTTTATCTGCTGAAAGATAAAGCATCAGCG

TATCTTTTAATGCAGATTCAAAGTTTCCCCGGAAAATAAGAGGAGCATCTATTCTGTAGTTCACAGGAGTATCCCATACA

AGATTCCAGGTTGAATTACCACACGGCGAACTTGACGCCCATTCTGTGAGGCCATCCCGCAGCGTCGAACCTTTTTTAAG

ATTCCAGGTGAGTTTTTCTGTCTGTTTTTGCGGAACGGTGACAGGCGTTAGCGATTTTTGAGGTAACTTGTCAGAGGAAA

CTGGTGTCCTGACAGGAGATGCAGGGGTTTTGTCAGGAGATACAGGTGCTTTTTGTGATGTTTTCCCTTTCTGTTGTTGA

AAATCAATCAGAAGATGTCTTTTTTCGGTGTTCTTAATTACCGTCAGACCGTAGTTGCGAAGCATTTTATCCAGGACATA

AGGCCATTGATCGTTGCCCTGCCAGGATATCGTGCGACGGAAAGTTTTCGTTACCTCCGGGGATATTTCAGCCGTCCAGT

CCGATGGGACAATTTCTTTTAATGCTTTTTGCAGCGAGCCTTTACCATCATGGCGAACAAAGTAAATTTGCTTTTCAGGT

GTTGATGGGCCTGTAAGCGTGGTGGCCGGAACAGCGTTGTTAGCGTTTTTTTTCAGAAAACCGCTTTTTGACATTAGCGG

TGGTTGTTCAGGACGCGGTTTTGTCTGCTGATCAGTCTTTTGTGCCGTTTGTGCCTGATGGTTGTTGCTACTGATTGGCT

TTGCGGGAGGGAGTGCTGGTTGTTCAGAATGCTGTTCAGTTGCTCCTTTGGCTGCTGTTTTTATCTCACCAGATGAAGTT

ATGCTGTTTGTTGTTGGCTTCAGAAAACTATTATTGCTGCCCTGGTTTTTTGGCGCTGTCGCTGATTGCTCAGAACGTGG

TTTTACGGTTTCTGCAATACGAGGCGCGGGAGTTACGTTTATGGCTTCCTTTTTGAGATAATTATCATTGCTGTATTGAA

CAGTGCTGTTTGGCTGTGACACGGGCTGCTGAAGGACCACCCCGTTATAGTATGTGTCTGCCCTTACCTGCTCAATTGTG

GTATTCAGTTCATACCAACCTCCTTTTGCCTGTGCGGGTTCAGGGGCATGGGAACACGCATTCAGCAATAATGTGGTGGG

AATAATAATGATCTTTTTCATGGTTAACCCCAGGAACGTCCGAACTTACTCATGATTTTTGAGATCGCCGCAGGGTCTGC

GGTCGTGCATTGATTAAGAAACGCTTTTCGGGCATCAAAGGTTTTGCTCCAGCGAATAGATCCCTTTTTCTTTTTGATGA

TATTAAAGAAGTCCTTTTCGGCGCTTTTGCATTCGCTCCCGCCGCCGCTACCGATAGCTTTACCGTACAGACAAAGAACG

GAAGCACACGGATCAGACGCATATACGGGGGCAGAGAGTGCGATAGCACTTGCGGCAACCATCGATAAAATGATGGATTT

CATGGTGTTCCTCCATATTTCAGGCAAAAGAAAACCCGCACGAGGCGGGTATGAATGAGCCATTAAAGGCGGAATTGCAC

TCTTTGTTTAGTTTTGATATCTATCCTGGTGCATCATCAGAACAGAATACTGGAGTTAAGTTGGCTATGGCGCGGTTTTA

TTTAAATGTTCCGTTTGAAGAAAAGGATTTGGCTAAACAAAAAGGGGCGCAATGGGACCAAGAACAACGGAAATGGTTTG

TTCCACAGGGGAAAAATCCAATTTATTTTATTCGGTGGATAAAAGAATTAAATGAACATGATTACAATGTTTTTTCGCAA

CGTTTTTATATTGCGGAAAGTCACCAGTCTTGTTGGCGCTGTAAGAAAACAACTCCTGTGTTTGGTATTTTTTTACCTCG

TTGGTATAAGTATAGAGACGTTATTTGGGGAGTTGATCCTGCCGAATGGGAAGATTGTATATTAGATGAATGGTATGAAA

CATCCTCCCCTAAGGGTATGGAGTATTTTGATTCAAAGAAAAATATGATTTATCGGTGGTTAACCAGTAGAGTATGGTGG

ACTGATTTAACGAAAATTGAAATAATTTCAACCTCTGCTCTCTCAAGGATTAATGAGTATTCAAAATTATATTATCCATC

CCATAGCAAAACAGCAAAAATGAAGTATTACGCGAATCATTGTTGTCATTGTAACGCAATGCAAGGTGATTTTATGATGT

TTAATGAGCCAGGGGGCGTGTTCTTCCCTGTAACATATGAACAGGCGGAGAAAATAAGATTCCATGAAGTCAATGAAACA

ATATTTGCCAAAGCAAGTTATAGTTTAATACCCGAAGCCGGAGGTTTCATAGATCTTTAAGCAAGATAAGACCCGCACGA

GGCGGGTATTCTTTTAAATAGAATCACCTTGCTATAGGGAAGGTGATTCTATTGTAAACGTTCCATTAGATCCCGCACTG

TCTTCTTGTACTCTTCTTTTTGTGCGTTGTTCTAATGCAGCACGAACATTAGAACTATTTATCAACTGGTCACTGCGTAC

AGCCAAAAAAAGCCCGCTTTGCGAGCTGTTTTTAATGAAGGATTATTGCATCATATTTTTAAATGCTTTACCCGATTGCT

CTTTTTGAATGGCTTCACGCGCATTTTCGCAGTTTTGACCAGACATATCCCCTTTGCTGCATTTTTCGACAACATCTTGG

GCTTGTTCAAGATGTTCAGTGTAGTAACTGACATCATAAACTTTGTCTTCACAAGCAGCTAAAAGGAAGCATGACAAGAT

AGTTAAAGTGCTTATGAATTTTTTCATTCGAACACCTATTGCATCATTAATTTAAACTTTTCTCGACTAACAGCCTCTTG

TCTTGCATTTTCAGACGCGATTAGCTGTCTCTCTGCAATATCATATTGCTTCAATTGTAGCTCCATTGTCAATTTTTCTG

TATTCAATTGGTTAGATAAGTCAGCTTTTTTTTGCGGAGTATCTGCTGACGCGAACTCCTTCTGTAACGTGGTTAATCTC

TTAGCCCTTTCCTGCATTTCATTGTTGAAATTTTCATAGAATTTGATCTTAGCCAGGATTGCATCATATTTTTGTTGCGT

CTCTGCAATGTTGCTTGATAAGTCATATTGCGATCGCAAAGAGCTAAGATCTGATTTTGCACTATTTGTTATTGTTGATA

GAGAATCCTTCATATAGCTGGATGCGCTATCAAAGCCATTGCTGAAATCAGTGAAGCCTTCGAGTCTTTTCTTCTGTTCT

TCAGCGTATCTTATGGTCTGTTCATATTGTTGCTTGAGTTGCTCAAGCTGTCTTGCCGCTTCAGCGGCACGATTTAGCCC

TTCTTCAACTGTTTTAGCTATCGCTGTAACGTCTACTACTGGAATACCAGATGCAAAGATTGATGGTGATGTTACGGTAA

TTATTCCAGCAAAGAGAAATGATAATACTTTATCTTTCATGTTAATCACCCCGCTTTAAGTTTACTTCCACGGTTTAATC

CCTGAACTAATGCTGATGCAGAACGACTTCCTGCATTAAAGAATGGATTTGGCATTTTGCTGGCAAATCCATTGGTGAAA

GATCGGAGTCCTGATGCTCGCATAAAACCAGATGCAACCTTACCTCCAAAGCCATTAGCGGCTGATGTAAGGCCATTTAT

ACCTACCCCACCTGTTAATGTAGAACAGAGTGTGCCAACTTGTTCTATAAGGAATATGGAAATAATAGTAATAAGGAAAA

AGTAAACTACAATAGATAGTTTTATATCTCCATTAACAGGGACAACTGATTCAACGAAACTAATTAAGAAACTAAATGAT

ATGCTATAGAATAAATTTAACAGTATATAGTTGAGACAACTGCCGACCCATGCAGTAAACATATTTCTGGTCGATGAAAA

TAGAGCGAAACAAATGAATAAAATGCCAGCGGAAAGAAGAATTCCAACCATGAAAGTTGATAGTGTCAAGAAAACAGTA
G

TGTAATAGATGAGAAGCATTCCGCCGATATAGCCAATCCCCCATACTAGATAAACCCCCATTTTCTCAGCAATTTGGAAC

TTTCCATAAATACTCATACTTGTATCAAGGAAGTTGTTTAAATTCGTAGATAGAGCGTTCCACAAATTATCAACACTGGT

TGCAGTTCCTGAACCTCCAGTAACAGCAGCAGAAAGGTCGGAGCCAGCGTGCATTACAAAAGGAATAACAAACTGGCTGT

AATAAGGCGCTGAATAAGTGAATGCACCAACCAGGGCGAATGCACCGATATTCTTCGTCACCTCGTTCATAAGGATGTCA

CGTTGGGCATACGTGATTTCATATATTAGATAAATAACGTATAAGGCTATAGCCGCAAAAAATGTAGGAGATATTGCCTG

TGCGACCTTAGCGGCGTTCGTCGCCGCTGTGGATTCAACAATGGTTGTTACTTTCTCAAGAAATTCTGAAACAATGTTCA

TAGCTTAGTCCTTCTGGCAGATGAAGAGTTCACCAGAGGCACTAATATTTGGCTTCAGCTTAATTACCAGGTCTGTGCCG

AAAAAAATTGCTTCTCCAATTTTTTGGAAATTTAATGTGGATTCTTCTTTATTGTAACTATAAACGTTGTGGGTCGCTGT

CGATGTTTTGGTTTTACGTGGTGTAAACGCTTTTTTAATGATGTAATCGAGAGCGTGGCTGTTATGAGATGGTAAAGTGA

TTAAATCGTCGTTTTTTTTCATAATGTAGTTCTGATTTTTGAGGTTGAAGATAATTGTCGGTCAATGCTTTTGATTTTTT

TTCGTATCTCAATTGGATTCGCCTCAGATAAGTTAGCAATGGATAAAGCCAGTTCGATTTGTAAAATTTTCCTTGTTTCG

ATGAGATTGATTTTTACTTGTGGTATATCACGGAAGACACCACAGCCGGTAATTGCTTTTGAAACGACACGTTCTGTATT
CGCTATGCTTTTCTTTACTGTTCGAGGGGGGACATAACGTGCACGGCGAAGTTTTCTCAGATTAGCTTTTGCTTGAGTCA
TATTTGTATGCCATGAATAAATAAATGCTTTCTTCAAAGCCTGAATAAGCATAAACCAGTAATATGGTGGCTAAAGGCAA
AGAAAAGGTGAAATGAATAACGGCATTCAGAACTGAACCGGTTAATGTATATAAGACAAACGGTGCTGAAAGTATGAAAG
CACCCAGCAGGAAAGAAAAAACATTGCACGCCATTTTTAATAAAAATATTTTTATTAGGTGATGTTTTTTTCTGTACATT
TCATCGTATCCTTCAGGGGGCTGTCACCTTCTCACGTTGTTGCTTCCATCGCGAGAGCTTCAACAAGTTCATTGATACAA
GTCCGTTCTGCCGGAACTTCCCGACCTGTAACCGGGTCGAAAACGATATGCTCATTTTCAACGTAACGCAGGCGATCGTA
GATTTTTTGACGAGCTTTTATCTTAATCACGTCCAGCTCTGCATGTTTTAACTTTTCTCGTCTATCGCCGATGAAAAATA
TTATCCATATGACAGCCATGATAATTGCCGTACAGGGGAGCATGTAGCTTAATACTTTTACAGCCACAGCAAATGTATCT
TTGGCCCTTCCTGCTGCTGATTCAAGGATAAAAAAAGCTACAGTTATTATTATGACTGTGTATGTCTCGTATTTATTTAC
ACCTCTAATACCAAATTTGCGTAATGTGGCTTTAGTTACAATCTCTGAAAGAGAAACACCTTCATTTGCGTTTCGCATTG
AATTTTCCTCATACTATTCCTGTGACACTTAACACCATCAATAAAATATGTAATGCAGTAATGTTTTTTAGCGTTTTTTT
GTAGGCTTCAATAAAATCCTTATCCAGAAGTCTGATAATAAAAATACATAGTGCATTCATCGCTGCTCCTGTAAGCGGAA
TTAACAACGAAAGATGCATGATTTATTCCTTGTTTTCACTCTTGTCTTCGATGGTCATTACTTTAAGAGTTATTCCTTGC
ATGTCTGTAACAATAACTCTTGTTCCCGCAGGGTAGTTTTCATTGTGTAACGTCTCAACAGGCCAGAATGTATCACCAAT
GAGTAATTGCCCCTTGCCGTTAACAATATCGTCACTGAGCGTAAATTCTTTTCCTTTAAAACGTGAATGCCCTGTGTTCA
CCAGGAGAGTGTCTAATTTCTTATGTTTTTTGTCATAGAAGAACTTAATTATCGACGCCAGTATGCTGATTGACGCAAAG
AAACATATATTGGCTTCTTGTGATATCGGAAGAAAAAAGCCCATAATGGCGCTTGATAATGCACCAAGACTGATTAAGAG
TAACCATCCCGTTCCGGTTATAATTTCAAGTGCAATACACAGAAGAAACAGTGTAAACCATAACATGGCTAATCTCCATG

ATAAAAGGCGGAGAGTGAATAAAGATAATAAAGGTGGTAACAAACGGCTAATACATTAAACAACAGACATAAACCGAAG
A

ATATGCCATTCAAACTCCATATAAGCAGAGGCATCGTTGCTATCATGAGCGTCGAAGATCCCCAGTCTAGCCATCTTGGA

AATATGGAGTCGATAATATGCTGTAGTCTTTCTCTTTCATTATTATCTCTTGTGATAGAAAGGAGAAAAAACCATGTTGA

CATGGGAATTATATACAAAATGATAATGATGCATGTATAAGTTTTCATTTTAATGGCTCACTCAGGAGTTAAGATAAGGA

CGGTGATTAAAATAATATTGTCACCGTCCGCTATCTTTTTTAAGACAAATATTGTTGAGCCTGTTTCATATCAACTTTGG

GAGTATCCTGTGATACGTTTTTTAGCAGTTCACTGATACCTGCAACCGAACTCACCAGGCTACTTGAATCAAGCGGCATC

ATAACCAGTTTACTGTTTGATGCTGTGCCAATTGCCTGGAGTGCTTCAGTGTATTTCTGCGCAATAAAGTAATTAACAGA

CTGAACATCACCTTCAGCGATTGCATCCGACACCAGTTTTGTTGCGCGAGCTTCCGCTTCTGCCTGACGTTCGCGTGCTT

CAGACTGAAGAAATGCTGATTGCCGTTCCCCTTCCGCTTTCAGTATTTGAGACTGTTTTTCCCCTTCTGCCTTCAGAATT

TCTGACTGGCGTATACCTTCCGCTTCAAGAATCCGGGCACGCTTAGTTCGTTCCGCTTTCATTTGCGCGTTCATCGCTTC

AGTCAGTTCTTTTGGTGGTTTAACATCCCGAATTTCAATACGTGTGACCTTAATTCCCCACGGGTCAGTCGCATAATCGA

CAACCGTTAACAGTTTAGAATTGATGCTGTCACGTTGAGAAAGCATATCGTCGAGATTCATTCCACCAACGACAGTTCGG

ATATTGGTCATAACAAGATTTGAAATAGCTGATGCAAGATTATCGACTTCATAAGCTGCCTTCGCTGCATCAATGACCTG

GACAAAACAAACAGCATCGATAGTTACGTTTGCATTGTCTTTCGAAATAACTTCCTGTTTAGGAATATCCAGGACGGTTT

CCATCATGTTGATCCGTTGACCTATGCGGTCCATAAACGGGATCAGGAAGTGAAGACCGGGGGAAAGCGTATGGGTATAT

TTACCAAAGCGTTCCACTGTCCAGGCATTACCTTGCGGCACGATTTTGACGGCAGATTTAACGAATATCAGCGCGACTAA

AATTAATAAGAGTAACGGAATAGATGTAACTATTGGCGTAATTATAGCGTCAATCATGATTAATCCTCTCCCCATTCATC

ACAATATGCTTCAACCGGAGTTTTTCCTGCTTCATAATCACCGCGCCATGCTTCCGCATCGGCGGCATTTCCACCGCGTA

ACTCTGCGTAATCCATTAATAGCTCGTGCCATTCTTCAAATGTTACGGTTTGTTTAGTGGAACCAAAATCAGCCATATGT

ATTTCCTGCATTTTGTGGAGGTATCTCTTTGCAAATGAAATCTGAAATATGAACATCAACAGAAAAACACTTTCCTGCTT

CTTTTTCTTTTGAGAAGTTTTTTCTGCATTCTTCAGCGGTCATAGGGAAGTCGCTGTAATGAGTCCATTTCACGGGGCCA

CCACCTGGTTTTTTGATGGTTGCGTTTATTTGGTACTTAGGCATTCAAAAATACCTCCACTCGATCACCTGTCTTTCTTG

AACACATCCAGCGGCGCAGTTCTGTAAAACTATCCTGGATCTCACAACGGGTACTTTTCATTGTGACAGTTGGTATATTG

TCGTAGCTGGCATATGCAATCTCTTTTTTGGGTGTGCGCAATATGCCTTTGTCAATATCAAGAATCCTGAACGCGCTAAT

TCTTCTTTTGTGCTCGTTAAATAGTGTTCGTTTAGTTGCTGTTATCTGCTTCTGTGCTTCCTGGTAAATGTAGGTAAGCA

CATCATAAGATTGTTGCTGTGTAATAAGGAAATTACGGCTAACATCCGTTGTTGTGAGCGTTTTATTATTTCTTAATCCC

CAATAGGCCACGATGATATAGAGTGGATATCCTTTCAAATCTGCCACACATTCAGGAATGACAGGATTAATTTTTAACTT

ACGCGCCATATTTTCTCCCGGTTGTTTGTCTGAAATAGAAACATCCCCGGAATACAGCGTCATAAGCTAATTATGAAAAG

TTGCAGCCACTCACTACAGGCTCTTTATCTGATTGTTCATCATCATGCATAGCTAAACCCCCTTACCTGGAGTGGAGACT

GGCAAATCTGGTGTACTTCTGCGTTGCAGCCTGTACGACTTGCTCAGGGAGAGAGTGAGTGAGATTAACTCAACGCGAGT

TCACCAAATAAGGGGGTTTAAGTATGCAGAATGATATTTGCGGATAAAGAGTCGTTACCTGTGTAGATGGCGCGGCACGT

CACTCTGTTACGTAGCCCGTTGCTGACAGATGCCAGCGGTGCTTGAAATGCACGAATAATAACCTCCTTATGCCAGGGCT

TTAGGCAATGACCGCTGTCCGGGGTTGTTAAAGAGCATGAGATGACAAGCGTTCTAACGGGCGTCAAAGAGTGCTCATTT

TCCAGGCAATGAGCACAAAAAACGCGATGAACGAGCTTTTTACAATCATCTGGATAGTATTGTGTGTGCGACATTTAAAA

CCTGTCAATGCCACAAGAGCGATCTAATGATCTGGATAACATCTAAAAAACAGAATAATATTCTGTTTGTTAAATGATGT

ACGATCTAAAAATCTAGATAATACTGTTTGTGTGGTTTATTCGATCGTGTGAGTGGGTGTATGCAGTCAAAAAAACTTTT

TTTGAATATACAAAATTCTGAATTTCTGATTTTGTAAGGTTTTGTATGGGGAACGTCAGTAAAGAAGGGGCTTCAGTAGC

TGTTCTGAAAACGATTTTGAAGCAGTCAGAGATGTGTGCTGAATCAAAAAGGAAACGAGCCAGGCTTGAACATCGTTGAT

CGATTGCGCCCATGCTGCTAAATTTGCAGGCAAGTTTTCCGCGATTTGTAAATGCAGAAAACGAGGTGCTGAAATTTTGT

TCAAGAACGTAAATTTCAGATAAAGAAAACCCCCGTAATCTCGTGTTTTCCAGGCAGCGAAGATTAGCGGGGGCTGACAT

CAAGGCAGAGCCTTGAGCTTATTTATTTCAGGTATGCTTGTTACCTGAAATGGAGTTTAATTCCATGAGAAGGAAATATC

AACTCTTACAGTGCAAGCTAAGTGCAGCTTGCTGTGATTAGCAAACGTCACTGGTCTGAACTATCACCGGAAGAGCAGGT

CCGTTTCTGGCAGGACTACGAAGCGGGAATTGAAAGCTCATTCCTGGTCCCCCAGGAAAACAAAGGCGGAACAACAAAAC

GTCGCCGGGGTGAACATTCCACTAAGCCAAAGTGTGAGAATCCAGCCTGGTTCCGTCCCGACAGCTATAAGGCGCTGGGC

GGACAGTTGGGCCACGCTTACAATCGTCTCGTTAAAAAAGACCCGGTAACTGGTCAGTACACACTCAGAATGCACATGTC

GTTGCATCCATTTTATGTGCTGAAACGTCAGAGTGTCGGTCGCAAATATAAGTTCCGACCGGAAAAACAGCGGCTGCTCG

ACGCAATATGGGTTGTACTTGTGAGTTTTTGTGATCGTGGATTGCACACTGTCGGCATGTCTGTCTCCCGCCTGGCAGAA

GAAATAAGTCCAAAAGACAGTAAGGGAAACGTTATTCCTGAAACCGCAGTCACAGTATCGCGCCTTTCCCGTCTGCTGGC

AGAGCAGGTTTGCTTTGGCACGTTGGGAACGTCAGAAAAGACGATATGGGATCGTGAATCCCGCCAGCGACTGCCGAAAT

ACGTCTGGATCACCGAAACTGGCTGGAAAATGCTGGGAGTTGATCTGGTGAAACTTCAGGAACAGCAACGTAAACGCCTT

GCAGAAAGTGAAATCCGTCTGCAACTGATTAAAGAAGGCGTTATCCGCGAGGGCGAGGAAATCTCTGTTCACTCGGCTCG

TAAACGCTGGTATGCGCAGCGTTCTCTCGATGCCATCAAGTTCCGACGAGAGAAAGCAGCAAAGCGCAAACGTGCCAATC

GCCTTGCAAAACTGCCGTATGATGAACAGCGAAACGAAATTGCACGGTTTATTCTGAAACGTATGCCGCCGGACGAAGCG

TACTGGTGCACTAAAGAAAGATTAGAGCAACTGGTAGCCAGGGATCTACGTCAGCTTGAACTGGCCCTGACAGCTTCGCC

ACCCCACTAGTTTAGCTGCCTGATACAATCGGCACTCTCTTCAGCCCCTTCGGGGCTTTTCGTGTTGCCTGCATTTCATG

AAATTCCTCATTTTTGACGACCGTTTTTCATTTTGTCCCGCTCTGACAGTTTTTTGATGGCCACAATCGGGTATCGGAGC

TAAACCGCCTTCGGTTATTAACAAAAACCCGCAAACGAATAACTTCAGAAAGCAGCAAACGTAGAAGATCAAACCCGCAA

TATATTCTTAACCCCTGTTCTTTAATCCCCTGCGTTGCTTCGCCGCAGGGAAAATATTTATCTTTGAAACAACTGTGGAT

AATTACAAAAATGCCTTCGCTTGCAGCGGCTAACGCCGCGCCGCTCAGAATATAAAAGTACCTCCCACCGCTTCGCGGCG

GGCATATGGACTTCTGCCTAATGAAAAACAAAGCCCCTCCAACCCTCCTACAAAAAAACGCTGCAAATACTCAGCCACAG

CGCCCAACCCATCACTGAAAAGCGCCGCCGCCCCCGCCCGAAGGGCGGGAACAACATCGCTTTCAATAATGGATGTTGTA

ACTAAGAAATTACATGGCTGTCAGTCTTCTGGCTGGAAGTACCAAGTACACGCTCGTAAGCGGTCCTGGCGGCCCGCTAA

CGCGGAGATACGCCCCGACTGCGGGTAAACCCTTGTCGGGACCACTCCGACCGCGCACAAAAGCTATTTCATGGCTGAAA

GCGGGTATGGCTTAGCAGGATGGGGATAGGTAAGGTGAAACCTATCAATCAGTACCGGCTTACGCCGGGCTTCGGCGGTT

TTACTCCAGTATCATATGTAACAACGGAGTGCCGCCTTCCATGCCGCTGGCGCGGCATCAAAAAAGAAAGCCCCCCAATG

ACGGGGGAAATAGCGTTTTGATCAAAATAATAATGCTAGTGTGCTTCTTGCGTGCCATTCCAGGCACAAACTAAATTTAA

CGTTTCGTTAACTACAAAGAAATCCGATGAGTTAATCACTTGCGAAATAAAAATCATATAGTAAATTACTATATGATTTT

GCGTTGTTTCGGAGAATTAAAAAATGCCTTTGATTGAATACATCAACAAGTTCTATCGGGGGAATCAGGCATCGTTCGCC

AGGCTGACCGGAGTTCAACCTGCGCAGGTAACACAGTGGATCAACAAAGGATTCATCGTGGTGAATCACACACTGTATAG

TCCACGCCGAAAGCTGGGAATTTAATTCCTGGTCAATATTTGACATCCTCCACGCCCTGAAGGACGGGGATATAAGGCGC

ACTGTATCCTGTTGTCTGGCAGAAAAAAGAAAGCCCCGTAGTTAATTTTTCATTAACCCACGAGGCGATCCCAATACTTC

AACAACACCAGGATAGACTTTTACCGCCCTTTACGCAATACCATGTATTTTCAAGTTTCTGGTCGATATATAATGTTGTT

GGCGCTTGAGGCTTTCTGCCTCATGGCGAAAAGGTGGTTTGTATCTTGTTGTGCGGCAGAAAGAAGAAAGCCCCGTAGTT

AATTTTTCATTAACCCACGAGGCGATCCCAATGCTTGAACAACATCAGGATAGCCTCTTACCGCGCGTTACGCAAGGAGA

AGAAGGCCATGAAACAGCCGAAAAACGCCCTGACCTGGTGTTTATTAATCGTGTGCTGCACATTGTTAATATTCACGTAT

CTGACCCGGAATCGTTTGTGCGAGGTCCGGCTGAAAGACGGAGACAGGGAGGTTACGGCAAGTCTGGCTTACGAATCCAA

CGGTAAGTAGCAACCCGGAGGCGGGCGAAAGCCCGCCTTTTTGGGGATGATGTGGTTCTGGCATTAAGCGCCTTTACAAA

GGGGGTATTCTGTACGGATACCCCCTTTTTCCATGTATTTTCAGGATATTAACTGGTGAATATGGTATAATTAGTCAATA

TGACAGCAATTATTGAGGCGAGTATGCAGCCCAAAAAAACACCCGTGATTGTGGTCAAAAAACGCCGCGTTCTGGTCATG

CCAGAAAATCCTGTGGTGAATGAAAAGCCGCAGGAAGTACAAAAATCAGCAGTTAATGAAAACAAGAAAGTACAGAAGA
A

AGATGCTGTAGCAGAGAAAACACGTAAAAAGCAGCCTCAGCCCTGGTATTTGAAAAAACAAATCACTTTTCCCCCAAAAT

ATCCGAAAGAATATTTTGAAAAGTGTTTCAATAAAGTTCGTGCTGTTTTTCCTGAACTATGGACAGACGAAAAAAAGAAC

TTGCCCCTGAAAAGCGGGATTCTCCAGGACGTTGAGAAATACCTGGCGGATAACCCGGATGTGGATCTGACGATTGAAGA

GTGGAATTGTGCGGTTCAGGTGATGACGTTCCGGTGGCAATATCTTCAGAATTGTACTGTACCAGGCGCAACACGTTACG

ACCTTTACGGGAAACCTGCTGGTACAGTAAAAAAAGCACACGCAACTTATGCGCAACTGGTTCTCGATGCCCGTAAGAAA

GCCAGCGAGAAGAAACAATTAAAACGTAAGGGATAAAGCCCCGAAGGGCTTTATCATTTCAGCCTGGCTGACTGTAGAAT

CCAGTGATTTTTTCCGTTTTCGTCCAGGCCGTGTTCGTAAGCTGCAAGAGCCGCTTTCATTATTGCTGTTTTTGTCTGGC

CTGTTCGCAGTGCTTCCCGTTCCAGAATATCTTCGTAAACGTCCCCAAGCCGGACATTGGTCACTTTAGTTGCTCCCGAT

TTTTTGCGGGTAGCTTCATTGATGAATCGGGCTGTGTCTGCATTGGTTTGTTCTGTTTTTTGTCCTGGTATTACCGGAGT

TTTCAAGTCAAGAGCCATTATATCACCTTTCGTGTGTTACATTAGCTTTACTGTGTAAAGCTAATGATGTAAAGCGATCG

TATTACATCCATTAGGGATTGATGCCTAACTCTTGCGCCAAAAGCTCTATTTGTGCTTTAGCTGTAGAAAGGCTGGATGC

GCGACGTACATCATGAACGCCAGCGCCTTCATTACAGGCCGCTTCAAAAATATCTAAATCTGAAATACGTGTACGAAGTG

GCTGTATCCACACCGGGTTCTCCCGGAGTAACTTGTCGAGATCAATCGCTGCTTTTCTGTGGCGTGGTTTTGTCGTGTTA

ATTCTCGTGAAAAGTACCCAGGGTTGCAGGTCTGGATTGATCTGTTGAGCTGTGCGAACTTTTTCTGTTACATGAGTCAG

CGTTTCTGCTTCGAAATCTGAAGACGGCTTAACGAGAGTAAGCAGAGTGTCAGCTACAGTTAAGGCGCTGCGAAATTCCT

TGCTGTCATGACCTGGACAGTCAATGATCAGCACTTCACATATTTTTTCAGGCGCTTAATGACTTCTGATACATCGCCA

TACTCTTCATAAACAGGAACCGGTAATAAGCCATTCTGCTTTCTTTTTTCGTTCCAGCTAAGAATATCATTGTTTTTATC

GGTCTTCAGAATGATGACCGTTCGTGAATTATTTACCAGATAAGAAGCCAGGTTTGTTGCTGTGGTGCTTTTTCCTACAC

CGCCTTTATCTGATGCGACAAGTAATGCTCTCCCCATAGAAACCTCGGATATACAGTGTAAAGGTTTATTTGTCTTTACA

TTGTATTCATTATAGTTACGTTGTAAAGATGTTTGTGTCTTTACAATGTAATTGCTTTTGTGCTTTACATTGTGTGTATG

TTACAGGGTAACTTTTCAGGATGCGATTTTGCCTAGTGTGTTGTTGCGCCCGTAGCTCGCTGACGCTCACACGCGCAGTA

CAGCGCCCCGCCCTGCTGCAATGCACCAGGACGGCGTAAACTCGCTGGCGCTCGCGCTAACTGCGGCATTCTGCGGCGCA

AATCAGCATTATCATGGCCTGTTGGTCTGTCCTGGCTTTGCAGGGCGAAGGGATGGCCCTGTGCATCCTGGTATGTTCGT

CAGCGGCTGCGCCGCGCTTCGGGGCGATGTGGCCCCGCTGCTCCCTGGTATTTTCTTTCGCCCCTTCTATCCGCTGGCGC

GGATTAAAAAATGAGCAAACCATCAAATAACTTGCTGTTTTCAGGCAATAAATCTTGATAATACATCTAAATAGATGTAT

TATTGTTTCATCGAAAGGGGATTGGCTCCTTTCCTTCTTAAGTCCAAACGGACAGGAGATTTAAAATGAAAACATTGACT

TTTAATAACGGCACTGTTTCTGTTGGCGATGTGTTTGTATCTTCCTGGGGATATGAGCAAACGAACGTTAATTTCTACCA

GGTTATTTCTGTTCATGGCAAAAAAACAGTAACTGTTCAGGAGATTCGCGCTTCAGTTCATCGGACTCATTCAATGAGCG

GATATAAGACTCCATTACTTAATGATTTCTGTGGTGAGCCATTAAAACGGCGAGTGCGTGATTATTACAGTTCGCCAGCA

ATTGAAATTGAGGAATTCGAAACAGCATATAAAGGATCACCGGAAGAAAAACACGAGTTCACATCATACTACTAAGAAAA

TAGTTGCAGGGGGTATCCCCTGCAACCATGTTAAAAATGTTAATAGTAGTATTTACCCAGGGGGTTTTAGTATGAATGAT

GGGGTAAAACTCCGTCCTTCAGGGCGTGGAGAATGCCGGGCTAATGCAGCAATGTAATGTTGCGCCCGTAGCTCGCTGAC

GCTCACACACGCAGTACAGCGCCGCGCTTCGGGGCGATGTGGCCCCGCTGTTCCCTGGTATTTCCTTTCGCCCCTTCTAT

CCGCTGACGCGGATTAAGAAACGAGCAAATCATCAAGTAAATTGCTGTTTTCAGGAAATAAATCTTGATAATACATCTAA

ATAGATGTATTATTGTTTCATCGAAAGGGGATTGGCTCCTTTCCTTCTTAAGTCCAAACGGACAGGAGATTTAAAATGAA

TATCCAGGAAGCATTAAACGTTTTTGGATTATCCGGCGAATTAACTGAAAAAGATATCAAAGCAGCATACAGAAAAGCCG

CTTTAAAATATCATCCAGATCGTAACCCGTTAGGGGCTGAACTGATGAAAGCGGTAAATGCAGCTTTTGATGTCTTGATG

GCAAATATTGATAAAATAAATCAGTTCCAGAGCGCTGATGAACATGCACGATATAATTACGGTGATGACCTGGAAAAAGT

ATTAAACGTTCTTTCTGGTTTATCTGGTCTGGTATTTGAAGTGATAGGTAACTGGGTATGGATTAGTGGAGAAACTATTA

CACATAAGGAAACTTTAAAAGAAATCGGGTGTAAATGGGCGGCAAAGAAAAAACAATGGTTTTATCGTCCAGACGAACAT

AAAAGTTACTGGAATCGTGAAGAACACACGATAGAAGAAATCCGCGCAAAATACGGTACAACCGGACAGCGCAGGGCGA
C

AGGGTGGCAACGCGTGGAAACCAGAGCGTAACCAGAACGGGGGCGAGAAGCCCCCGATATCATACCAGCCCGTCTTTTGG

GGCATCGAACCCAAAAAGACGGGAACAGCAGCCACGTAGCAAAAAGGGAAACAGATGAACTCTTTTTTTGAACAGTATCA

CCCTGTCTTTGAAGTTGTCTGCCGTATCCTGGGGAACGGCTGGCGCGTGAACAAACTTGATGATTGTCCGTCTCGTATAA

AACTGACGTCACCGCAGTTTAAAAATTACTCTGTGCATATTCGGATGGAAAAAGACCGATTTTCTGTTGTGGGGAGTGTG

GATAGTCGTTCCTGGAGTAGTCCGTATCATGTTTGTACGTTATCCAGGAAGCGGAATCCCGTTGATATAGCAGCCAATAT

CGAACGAAAAATTTTGCTAAATGCTTCGCAGGAGGTGTTACAGGCGATTGAGTATGAGAAACGCCAGGCGGCAAAAAAGG

ACGAAATTCTGATCCTGAAAGGTATGTTATCGCAACTTGTCCAGCTTGAAAGCTGGTATGGGGCATTGACAGGCTTTAAA

GCTGAAAATGGATTGAACGGTAAAGTCACCGAGCAGGGCGAGCGTTATGATTTGCAGATTAGGGGTTTGAGTATAGATCA

ACTTGTTAAAATTACAGGATATTTGAAACAGTTATGAGAAAAAACAAAGGGCGATTGACTTATTATCTTGAAGTGATTGA

TAAAAAATACCATTTTGTAAAAAAAATAAGCAGTTATTCAAAGGAGTTCACTGACGGAAAAACAAAAAGAACAAAGAGA
A

CGTTAAGTGAGCTGGTTTTTAATGAAAGTGAGGTCGAGGCAATAGACTTTACAAAAAATGGTTTAAGACCTGTTGATAAG

AATATTCTCTTAACTATGGTGAAAGAATATAAGGAGAGTGATGCATGACTATTGAAAATACACCGGAAAACATAAAGAAA

TTACGAAAAAAAATTGGCCTTACCCAGACAGAGTGCGGTGAGATTTTTGGCGTAGGCTTAAGTACATGGCAGAAAAAAGA

AGCTAAAACCCACAACCAGCTTAATTTATCGAAAGGTGAATTTGAGTACCTGTTATTACTTGCAGGGGAGCACCCTGAAT

ACGTCCTGCATAAAAAAGCTGAATCCCGCTCACCGGAGGAACCGTGAGCGAGAAGAAAAACACCACGACGCAGAAATCT
A

AGATATACAGTGTAAAGGTTTATTTGTCTTTACATTGTAATTGTTCTTTGTCTTTACACAGTGTGTGTTTGTAACAGGGC

GACTTTACATAATTGCGATTTTGCCTGGTGTGTTGTTGCGCCCGTAGCTCGCTGACGCTCACACGCGCAGTACAGCGCCC

CGCCCTGCTGCAATGCACCAGGACGGCGTGAACTCGCTGGCGCTCGCGCTAACTGCGGCATTCTGCGGCGCAAATCAGCA

TGATCATAGCCTGTTGGTCTGTTCTGGCTTTGCAGGGCGAAGGGATGGCCCTGTGCATCCTGGTATGTTCGTCAGCGGCT

GCGCCGCGCTTCGGGGCGATGTGGCCCCGCTGCTCCCTGGTATTTTTTTCGCCCCTTCTATCCGCTGGCGCGGATTAAA

AAATGAGCAAACCATCAAATAACTTGCTGTTTTCAGGCAATAAATCTTGATAATACATCTAAATAGATGTATTATTATTT

CATCGAAAGGGGATTGGCTCCTTTCCTTCTTAAGTCCAAACGGACAGGAGATTTAAAATGAAATTCACTTCAACCACGAA

TCATGTTTTCACATTCGAACGAGTAACGCTTTGCACGATTGTTCTTATTCATAAGGATACGGGACAACAGTATGTTGTCA

TATTTACGGATAACAATAAAATTCGTGACTATAAGACCGGAATTGTTTCTCAGTTTGGTGAACTTAAGCAGAGTGATATT

GATTTAATTCTTTTCTATCGGGACGAATATGAAAAATATTTTGACTCGTTAAATAATGGCGAGGAGTATTTGAGTTTTAA

AGAATATATCGGATGTATAAGGGGAAAATAAATTATGAAAGGTGTGTTCAGGTTGCTGGAACAACCTGAACACGTTATCG

ACAATCAAAACTGAATGAGGTTTTTAAATGTCAACACGACAGATCGTACAGCTTGCTGACTCCCTTGTCAAAGGCTGGGA

AACCAGAATTCCGGCAATGACGTGCACGGAGTGGGATCAATTCCGGTGGTGGTTAAACTACCTGCAAGGGTATGAAATGT

TTTAAATAACCGGGATAAATTCATATATGGGGAGAGTGTCATGCTCTCCCTTTCTTATTGGGTAGCGAGTTGTATTACAT

ATTTAGCTTTACGTTGTAAAGATTTTATTGTCTTTACAGTGTGGTGTGTTTTCCGGCGGTTGCGCTGCGCTTAGGGGTAT

GTGGCTTCGCTGCTCCCTGGTATTTTCTTTCACCCTCCTATTTGTCCGATGACTTTTGACAACAACGCTATTTGTAACTA

TAATTAGTTGCATGTTGGCAAGGGATGGGATTATAGCGTATGGGGAAAGCAGATAAGCTACTGGCAAAATTTTTAAACAG

TAAAAAAACGTTTGAATGGGATGAACTCGTTGTTTTGTTTTCTTCTCTAGGATATGTCAAAAAGGAAATGCAGGGATCAA

GAGTGCGATTTTTCAATGCTGAAATTAATCACACAATATTAATGCATCGCCCACATCCAGAAAGTTATATTAAAGGTGGA

ACGCTGAAAGCTATTAAACAGAACTTGAAAGAGGCTGGGGTTTTATGAAACATTTAAAATATAAAGGATATTTAGGTACG

GTTGAGCCGGATTTTGAAAATAATATCCTGTATGGAAAGCTGGCATTTATTCGTGATCTGGTGACTTATGAGGCAGAAAC

ATTGGCTGACCTGGAACGGGAATTTAAAACATCGGTTGATTTGTATTTACAGTCCTGTGTGGAGGACGGAAAGGAGCCTG

ACACGCCCTTTAAAGGTGTGTTTAACGTCAGGCTTGATCCAGAGCTGCATCGTCGGGTCGCTGAAATGGCGATGGAAGAA

GATTTGTCGCTGAATGCCTTTGTCAATAAGGCACTGGAAAAAGAAGTCAGTCATCATCGCGCAGGGGCTTAATCGCCCCT

TTTTGTTGCTCGCAGGCGTTGATGTTTCTTGGTATTACGGTGTAGTGTGTTGTATTTACAAACAATGCTTTACGGTGTAT

TACGATGCATACTCTTTAATTGCTTGCAATCTCTTTTCACGTTCGTTGCTTTGTTCAGCTTTTTTCAATTCACTGAATAT

GTACTCTTCATCTTTACCCGTGAGTTTTGAACCGGAGAGCAAGCGATTAACAACTTTACGTCGAATTGCAGGGTAATCTT

TGGTTTTATCGAGTATGGACAATATGCGGTCACTACTAAGAATCTGGCCTTTCCCTGATTCCACATAATGTAGAAAAAAT

GCGATTGCGGACACGCCCAAAAGCACTGTAAGGACGGCTACACCATCAAGACGAAACTTTGATATAAAGGAATACGCGAC

CACTCCAATAATTGCTAAGCAGAGACATAAATAAATTAAGAGGTTTTTGTTGCGTTGTTTTTTTTGTGCGCGATCATGAT

CATCTATGATTGCCAGTATCTTTTCTGGAGTTGCTTGAGCCATTTTTTTTCCTTATCAGGGAGACGGAGTGTTCAATCAT

CTGGCTTTACAATGTAAAGCTATGTTTGTCTTTACGGTGTAAATAATGAGATGTGCAGTTAAAAGTGTCAATCAGGTATC

ATATAGTAAAACACTATATGATTTTTCTGTAATAGGCTTAAAAAATTCTCGCTTACATTGTAAAGGTCTTTGTGTCTTTA

CAGTGCACCACGTTGTAAATCACTGCCTTCTGTGGAGATAATCATGTTTTTTTCTGTTGGTGTCGAAACACCGAAAGATG

ATCAGACTGCATACGGCATCACTGTTCCGGCTTTTGATTGTTTTGGTTTTGGATGTGTTTCTGCGGCTGATTCTCAGGCA

GAAATACCCGCTATGGCGCGTGAAGCGATATTGGCAATCGTGGAAGATATGGTATTAAGCGGTGCTTATTCTGTTGATGA

TATTCATGATGAAGGGTGTTTGACTTATTCGGATCACTCCAATTATAAGCACTGTGATAGCTGGTTTTTGGTTGATGTCG

ATTTAACATTAGACATGCCGTAGTCCGGCTATCGGGTCATGAGGACGCTGAAGAATATCGCAGGCATATATTTACAGTGT

AAAGGTGTTTCTGTCTTTACATTGTAAGGCTTATTGAATACTACACTGTAGTGCAATGTATGTGTTTTTATGACCTATCC

CCGCGTTGTGCGGGGATTTTTTTTGATAGCTAACCGCTCGCCGCAGTCTAGCGACCGAGCATAGCGAGCGAGTGACGAGG

AAGCGGAAGATCCACCGTTGCCAGGAGAAAAGCAGGTACGCACTATGCATTCTGCCCTAGTGATGCTTTCACAAACCACG

CGGCCTGAACTATTACGCCATGAATCCAGCGCAAATCGGCATCACGATTTTCTTTTTTCATTTTTTCGCCACTTAAAGCC

TTGCTTACGTGACTTTTTACCAGATTTTCGTGTAATTCTTTTGCTTCTTCAATTGTGAAACCACCAGGCAAATTTGGCGC

TTCTGCTTCCAGTGATGCCAGAGCAATTTCATAAGCCCGGCGCTCAATATTGTCTCGCACGTCCAGGCTGCCGATTCGCT

CCCTGATTTCTTTAATTAGTTTCTTGTCGTCGTCGGTAAATGTTGTCATCTCACTCTCCTTTGGCGTCAATGTTTACAGC

CTGGCAAGCCTCTTTGAGCACCCAGTCAATAGCGTCTTTCCATGCCCCTGTTTCGACGGGAGGATTCGTGTGCTTTACCT

GATCGTAGAAGCGTACTGCGTTTACCAGTCCTCTTGTTGGTGCTGCTATCGCAGGTGGTAAGTTGTAGAGTTCCTGAATT

TCATAGTTCGGCCTGTCGTTGCAGTCTTCTTTTGTCGGCACGTATTTCCAGTCACCAACCCACGTTTTACCCTGAGAGTC

CGTAACGTCTTTTTTCACGTAACGATACCTCCATGCAACAACCCCAGCTTCCAGCGATGCCAGTGCGATTTCATAAGCCC

GGCGTTCAATATTGTCTCGCACGTCCAAGCTGCCGATTCGCTCTCTGATTTCTTTAATCAGTCCCTTGTCGTCGTCGGTA

AAAGTTGTCATTAGTGACTCCTTTTCATATCTATGGTTTTCTTGAATCAATTTCGTCTAGTTTGAGTTTTATATTTTGCT

GGTCTGATTTTGGTAATTTTCTCATGCTCTCTTCAGTTATTTTTTTCATATCAAGAAAAGCTCTTGCATAGAAGCGCAGG

CTTGCCTCTTTTTCATGTTGTGCAACATACCAGGTTGCGCCAGAAAATGCCGCAGACAGGCATAATGTGCACAAGGCACA

AATTGCAATGGCTGTTCTTGTACTGAAGGGTTTTACAGCCTTAGATAATGACTTCAACTTTTCTTCATATAATTTGTTGT
TTTTTTCCTGGTTGCGTTTAATTCCAGATATGAGTTCAATAATATCATCTGTTAATTTTTCTTTTACCTCAACACTTGTT
TCTTTTAGATCGTTTTTTCCCTCTTCTACTTTTTCTTTTATTTCATTATCCAGATCGCCGCACGTCTCAACAATATTCTT
GAATCTTTGAATATAGTCATTTTCTACAGATGAAACGACCTGTCTGAAGCCCTCAGACAGGGCAGTGAAGTTAGCTTGTA
ACTCATTGCTGAATTTTTCCTGTATCTTTTGGTTCAGAAAAATTAATGAAATAACTGGATCATCTTTTGATATTTTATGC
CCTGTCTCCTTGAATACATCAACAATAAAATTATCAATATCTTCACTGGAAACAGCCTTTTCAGGCTGTTTTAATTCTGG
CTGATTAACTGTACTCATATTATTCACCGTGTTCATCGTCATAGATGCTATCTAATTGTTTGTAAATGTCATTGAAGACT
CGTTTTAATCGTGATTTAGCCATTAATCCAAAATGGTCGGACTCAAGAGCTTCTTTCAGTGTTAGTGATTTTTCTGTTAG
CTCTTTGATGTCAGAGGCAAAGGCATCACTTTTTCTGTCCTGAATAACCACAACGCCAGCGATTACATCCTTGTTTTTTT
CAATGAATTTAGTTTCTATTAATGGGATATTTTCAAGTGCTGGAATACCCTGAAATTCATTTATCCAGACAATAAGTTTT
ACCTTACTTCCTTTAACCAGTTCTTTCAGTTCTTCAAATCCCTGCAAGGTATCCGCAAGAGCTTGTCCACCAACGATAAC
AGTATGGATATAAACGTCTTGTTCTACATCTTCAAACATGTCCATAACACAGTTATCATTGAAATACTGAATTAACGGTA
AAAATGTAGATGCGCCATTGTCAATAACAAAAGTATTCTGACTATTGGTCAGCATTGATTCGAACATTGGATCGAATTTG
GATTGTATTACTTTGCTGTTTTCAGTGATTTGTATCAAATCTGCATTTAATCTTTTTACTTTAACCGTAGTTGTATTAAC
CGGGTCTGTGTCACCGACTACGATATTATCCATGTGATTTTCATCAATAAAGTATTGTGCCAGTATTGCGGTCGCGAATG
ATTTTCCAACACCACCTTTACCCTGAAGGATGAAATTGATTGAGTTTTTCATTTATAATGCTCCTTTACTTAAAGATATT
TATTTATGAATTTTTCATTCGTAGCACTTGGATCGTGCTCTATTTCTTTTTTGTTCTCACTAATGTTTGAAAAAAAACCT
TTTACCCCCCCTCTCGGTTCATCGCCTTTAGCTTCATTCTGATCAATTACATCTATGGTTTTTGCCTGTTCTTTTGGTTC
AGTGCATTTTTGTCTTTTTTTAGCCCTCTCTGTCATATTTATATAAGTCTTTTGGGCTAGTTCTGTCTTGGTGTTGTTGT

TTATGTAATCAGCTATTTGCTCCCATGTATAATATTTCTTTGCTTCAAGTATATTTTGCAAATACATATTGAAGACAGCC

TGTTTTGATTTCGATTTTGTTTCGTTTATAGCCTCGCATATTGGAGATAATAAACTCTCGATCTTTTTCATTTGTTACAT

TTCCTCTCTCTCTCTACCTCCTACATTGGCATAATACCTTATTTAAAGCAATTTGCTCGCCGTTTGTGTGGGTGATTATG

TGGGTTGTTTTGTGGGTTGTCAATGGGGGTGTGGTCTTTTTTGTGGGTTAAAGTGTGGGTGATTATGTGGGTTGTGTGTT

TTTGGTTGTGGGTTATTATGTGAGTCATTGTGTGGGCTGTATGCACGGCATTTTTTGTTCTTCTAAAACACAGGCACGAT

ACCGATTGTACACAAGACCGTTCCGGTCATGTTACAATCGTGTGCCAAAGGGGGACGCCCCTTTGAAACCCCGAACGATG

CAGCCGTTCTTATGCGGTTCTACGAACCTTGAGGACATATGAAGAAAAAAACCAACAAAAACGTTCATGTAACATTCAGA

CTTACCGAAGAAGAATATGCTCCGTTCGATAGGGCTATAAAAGAGCTTAATATTAGTAAGTCTGAATTTTTCAGGCTGCT

TACTATTGGTAAAATAAACACATATGCATCTGATAAACGTAACATACCAGAATACAAACGTTGTCTTTCTCAGTTGAGTT

GGGCAGGAAACAACATAAATCAAATAGCGCACCGATTAAATTCAGATCATTTAAAAGGTATTATATCAGAATCGCTTTAT

AAAAAGGTTTTAAATGGACTAATCGGTATTCGTGATCGCCTTCAGGAGATAGCTAAATGATTGTCAGATACGGTGGTGGT

AATGATGGTATCGTTGATTATCTGATAAATGGTCGCAAAGCAGAACGCCAGTACACGCGTGATGAACTTGATCATCGTGT

TGTTCTTGATGGTGATTTACAGACTACAGATAAGATTATTGATTCTATTGAAAACAAAAGTCAGGAACGTTATTTGCACA

TTACTCTGTCTTTTCATGAAAGTCATGTGTCGAATGAAGTGTTAAAGGCTGTTGTTGATGATTATAAAAAATTATTGATG

AATGCTTATCATCCTGATGAATACTCTTTTTATGCGGAAGCTCATTTGCCTAAAATTCGTCATATCCAGGATAATAGCAC

TGGTGAACTTGTTGAAAGAAAACCACATATTCATATTGTAATTCCTAAAGTAAATTTGATTACTGAAAAGTTCCTGAATC

CAGTGGGAGATGTTACCAAAGGGCATACAATTGAACAGCTTGATGCTATACAGGAATTTATTAATAATAAATATAATCTT

GATAGCCCTAAAGACTATCCGCGTAAAGATGCGGATTACGGAAAAATTATAAGTAGAGTTAAGGGGGATCTTTACAAAGA

GCATCACTCTGAATTGAAAGGTGAGTTGCTTTCGCGTATAGAGAACGAGAAAATTGAAAATTACTCTGTATTCAAAGATA

TTGTTGCAGAGTATGGTGAACTTCGTATAAGAAATGCTGGTAAAACCAATGAGTATCTGGCTGTAAAATTGCCTGGAGAT

AAAAAATACATAAACTTAAAAAGTCCTCTTTTTCGTCAGAATTACATTGAGACAAGGACATTAACACTTGAGAAACCAAC

ACACAAAGAGATAGAAAAGAGACTTAATACCTGGTTAAATAAAACAAGCCAAGAGATTAAGCATATTTTTAACCAGGCGG

AGAAAACAAGGGAACTTTACAAGACATTAAGCCCTTCACAACAAATTGATTTCCTGCAAGAGAGGATAAAAGAATATGAC

TCAAGAGAAAAACTTAACGAAAGAAATTCTCAGCAAACGTCAGGACGAGCGGGAGGTTACAAGTCGTGTCCTAAAAAGTT

TGCCAGAATCCGTCAATCTGAAGCAACAGTCGGATTGTCACGTATGCCCCAACGCGGTATGGTTTACGGAATCAATGGAT

TCACAAGACCCGACTCTGTCAGTGTATTGTCAGATATTTCGCAGCGTGATCTGGCAGAGCAATTATCGCAAAGAGAACAT

CCTGGTCAGGATGTGCGACGGGATTATGATAGACAATTCACAGAGTCAGGAATAAAAAGTCTTGAACGCTCTTCTTTTTT

GTGTGAAACGATGTTTCAGACTCTGAATGAGGCGGCAGAGAAAAACGAAATTGCAACGATGGCAGAAATTCGCAGAAATA

TTGATCCTGTCAGGTTTCTGTCTTCTGCCGCCGAGCGTTTTAATATTATTCCAGCGCAGCATAAAATCAGAACAGCAAAG

GACGGTTCCCCCCGGTTTTCTGTGGGGAATCGTAATATGAACGCATCTGATTTTCTGACAAAGCATATTAATCTTGCCTG

GAAAGATGCGAAATCCTTTCTGCTTGAAGTTTATTCACAGCAGTTAGAAAACACGCCATATACGCGCTATCCAACCTACA

GACGCCTTACGCATCATGAAGCCCGTGAGCGTCTTAACTCACTGAATTTATCGGAAAAAACATTACGAAATACTATCAGG

TTTGAGCGTGGCAAGCTGTATAATGATCTGCGAGAGATGAGACGCGAGTTGAAGTTAATACCACGTGAACAGCGTGATAT

TGCCGTGGGTGTGATTGTTTACAAAAAACTAACCACACTTGAGCGTCTTTCTGAACTCGACACAGAGGGGCGACACATAA

TTCGTCAATATCATGCTGACTGGCATAAGGATAAAGATGAAATGAAAGCCCTTGAACGTCTCAAAAGCTATCTCAACTTC

GATGAAATCAACGCCATTTCTGCTGACGAACCCGAACTTTCGCTTCAGAAAGCGGTGGATTCCCAGCGCCGCTTAGAAGA

GGCGAAAAAGGTTAACTCCAAACTAAAAGATCTTGTGATGGATAAGCAGGATTCCAGGATTGTTTATCGCGATCAGGAAT

CGGAAAAGCCTGTCTTTACCGACAAAGGGAACTTTGTCGTTGCGGGTAAAAATCCTTCAAAAGAAGAGATCGGAATAATG

CTTGAGTATTCCAGGGAAAAGTTTGGTGGTGTACTCAAACTTACCGGCTCTGAAGATTTCAAAAAAATGTGCGCTGAAGT

TGCCGCAGAGCAGGACATGAAGATTATTTTACGCCCGGAGCAGTATCAACAGATGATGCTGGAATTAAAAGCAGAACTTC

AGGGTAATAAGTTTGAGCAGGTGGAAACACAGGAAAATAGCCAGGAGTCCGAATCCAGAATAGAGAAAGGTGATGCATTG

AAGGAACAGGCCACCGAACAGGAACAGGCTACAGAGCAGGCACAGGCTACAGAGCAGGCACAGGCTACAGAACAGGCA
CA

GGCTACAGAGCAGGCACAGGCTACAGAGCAGGAACAGGCCACCGAACAGGAACAGGTCACCGAACAGGAACAGGCTACC
G

AACAGGAACAGGCTACCGAACAGGCACAGGCCACAGAGCAGGCACAGGCCACAGAGCAAGCACAGGTCGCAGCGCAGG
CC

ACATCTTCATATGATCCTGGTGTTATTACCAGGGCAAACACCCTGGATTCACAGATGCTTAGTAAAGGCACAAATGGTGA

ATTTGGCTATTTGAAATCTCTGGATAGCGATGAAAATGAGATTTGGGAAGTGCTGGGACATGTACCTGGAGACAGCGATG

ATATTTTTGACGTTGCCAGTTTTGATAATGAAAACGACGCAAAAGAATTTTGCAAAATTGTGAATGAGTTAGGTATTGAC

AGAACACAGGCTCTGATTCAGGAGCAGCTTACTACTCAACACGATCAGGCTACTGCGCAGGTGCATAATAAACAGGAAAT

TTATTGTATAAACTTTAGTCGCTTCCATGATTTGAATGAAGGTATCGTCTTTCATTCTAAAGATGCGGCTATTCAGTGTT

ATGAGGAAAGCAAATCTTCTGCAATAGAAAAATATGAAAGCAATTATTTAAATGGTGATGGTTTTGATACTGTTGTGCTT

ATGTCTAAAACTGTCTCAACCGATGAATTATCCTCTTATCCAGAGGGGGCATTAGTAACGTTTGATAGGCCATTTGAAAT

AATTGCCAATTCATATGAAGAATACCGTACTCCAGTGTATGCGGTTTCTTTTTCGAAGGATGAGTTTAGTGAAGATATTA

AGACTTTTGAGTCATTGCACGACGCATCTGAATATAAAAATAAGATGCTTCAGGAGCACGGGCTTAACCAGGATGATATC

TTAATCACACCTGTAACAAGAGAAGAAATCGCCTTTAAAGGAATTAAGGACGCTGTTAATGACGCTAATATGGCAGTGAT

GGAACAGGCGGGGGATTCGTCCAGAGAATCACCAGAAGAGATACTCGCCAGTATTTCAGCTAACGAGCATATGATCTCCG

GGCTTGAAAACTTCCTGGTTAAAGACCGTTCTCAGTTCAGTTCCTGCAATGGGGATATCGTTGTGGAGGCGGAAATTACC

AGGGGGGAAGGTGGTCTTTATCACCTTGCGGTCGCTGGTAAGCATGGCTTAGAGCGTGGTGATGCAGTTGCCAGAGTGGA

TGTTACGGAGCAGCAATTTGCCGCGATCACTGGAAAAACACCTTCTGAAGTTTTAACGGGGGACCAAACATCGGCTCGTG

TTCCGGTAATTACAGGTATCCATTTCAGTACAAGGGCTATTGAAAATGTGAATAAACTGGAACAACAGAAGGATTATGTC

TATTTCTCAACACATGAAGGCCTGAATGCTGAAATAAAAGATTTCAGCTCTCTCAAGGATGCTATTGAGTGGGGCCGGGT

TGAATGCGAGCTACATGATCTCAATAAGCGCGATACTGTCATTTATCGCGTCGAGTCTGAACATATTTCTCAGGGGATTG

ACGCTGTAATGAAAAATGCTGAACGCGTGGAGCGTCATGAAATTGAGAAAGCACAGGGACGGGATTGCACGCCGGAAGAC

GGCAAGATTCTTGAGGCAATTGATCGCTTTGAGGACAAGTTCAGAGGGGAAGGACTGAAGTTCGAAAGGGAGAAGGCCGA

ATCCGATTTGCTTAATCACGGATTCACTCGTGAAATGGCAGAAGATGCTCTTGGAAAACAATTTGTCCAGGCCAGAGAAG

AACATCTGGAATTGCAGCAACAGCGTGACAATTCACAGGACATGCACTAATTAAGAAAAAAGCCCGGTTTTCGGGCTTTT

TAAGATACAAATTATAAATACTCTCAAGTGTATATTCAGTATGGGATTGCGCAATGATTGCCTAATAAAATTTCTGAAAT

ATTTCTGTATCGCATAATTTTTTATATCAGATAAATTGTACTGGATTTCTTAAAAAATTGCAGTATAATTGCCGCAATTA

TCCCACCGTTTATTTTTTGATTAGTTTCTCATGATGCAGCATACTTCTGTGTGGTACCGACGCTCGGTCAGTCCGTTTGT

TCTTGTGGCGAGTGTTGCCGTTTTCTTGACCGCGACCGCCAATCTTACCTTTTTTGATAAAATCAGCCAAACCTATCCCA

TCGCGGACAATCTCGGCTTTGTGCTGACGATCGCTGTCGTGCTCTTTGGCGCGATGCTACTGATCACCACGCTGTTATCA

TCGTATCGCTATGTGCTAAAGCCTGTGTTGATTTTGCTATTAATCATGGGCGCGGTGACCAGTTATTTTACTGACACTTA

TGGCACGGTCTATGATACGACCATGCTCCAAAATGCCCTACAGACCGACCAAGCCGAGACCAAGGATCTATTAAACGCAG

CGTTTATCATGCGTATCATTGGTTTGGGTGTGCTACCAAGTTTGCTTGTGGCTTTTGTTAAGGTGGATTATCCGACTTGG

GGCAAGGGTTTGATGCGCCGATTGGGCTTGATCGTGGCAAGTCTTGCGCTGATTTTACTGCCTGTGGTGGCGTTCAGCAG

TCATTATGCCAGTTTCTTTCGCGTGCATAAGCCGCTGCGTAGCTATGTCAATCCGATCATGCCAATCTACTCGGTGGGTA

AGCTTGCCAGTATTGAGTATAAAAAAGCCAGTGCGCCAAAAGATACCATTTATCACGCCAAAGACGCGGTACAAGCAACC

AAGCCTGATATGCGTAAGCCACGCCTAGTGGTGTTCGTCGTCGGTGAGACGGCACGCGCCGATCATGTCAGCTTCAATGG

CTATGAGCGCGATACTTTCCCACAGCTTGCCAAGATCGATGGCGTGACCAATTTTAGCAATGTCACATCGTGCGGCACAT

CGACGGCGTATTCTGTGCCGTGTATGTTCAGCTATCTGGGCGCGGATGAGTATGATGTCGATACCGCCAAATACCAAGAA

AATGTGCTGGATACGCTGGATCGCTTGGGCGTAAGTATCTTGTGGCGTGATAATAATTCGGACTCAAAAGGCGTGATGGA

TAAGCTGCCAAAAGCGCAATTTGCCGATTATAAATCCGCGACCAACAACGCCATCTGCAACACAAATCCTTATAACGAAT

GCCGCGATGTCGGTATGCTCGTTGGCTTAGATGACTTTGTCGCTGCCAATAACGGCAAAGATATGCTGATCATGCTGCAC

CAAATGGGCAATCACGGGCCTGCGTATTTTAAGCGATATGATGAAAAGTTTGCCAAATTCACGCCAGTGTGTGAAGGTAA

TGAGCTTGCCAAGTGCGAACATCAGTCCTTGATCAATGCTTATGACAATGCCTTGCTTGCCACCGATGATTTCATCGCTC

AAAGTATCCAGTGGCTGCAGACGCACAGCAATGCCTATGATGTCTCAATGCTGTATGTCAGCGATCATGGCGAAAGTCTG

GGTGAGAACGGTGTCTATCTACATGGTATGCCAAATGCCTTTGCACCAAAAGAACAGCGCAGTGTGCCTGCATTTTTCTG

GACGGATAAGCAAACTGGCATCACGCCAATGGCAACCGATACCGTCCTGACCCATGACGCGATCACGCCGACATTATTAA

AGCTGTTTGATGTCACCGCGGACAAAGTCAAAGACCGCACCGCATTCATCCGCTGATTTCTCCCTGTATTTTTCCAAAC

CCACCGCACACTCCATTCGTATTATGGGCGGTGGGGTGGGGTTTGTTATGCCGTATTTATCAAATAAACGCCTACTTGCT

GAGATGAGTATCGCTCTTGTCATGGCGATCGTTGCCACGCTGACCCTTGAGCACAGTCAGATTGATCTGATGGTCGCTGA

TTGGTTTTATCTGGGTATGGGGCATTGGATGGTTGCCAAGCAAGCTTTTTTGCCAGATTTGCTACTGTATTCTGGACTAA

AAAAGCTGCTGATGGCGATGCTGATCTACTTGCTGGTTGCGACCATTTGCCGTGCTTATCATGAGAAAAAGGGCAATGCT

ATCACTGCCAAGTGGCTTGTCCCAGTGACAAAATTTCGCGTGCGTGAGCTTGCGTATCTGGTGCTGACTTTGATCCTAGT

GCCGACAGTTGTCGCGTCATTGAAGGCATATACTCATGTGGTCTGCCCTGTGCATTTGACGATTTTTGATGGTACGCTGC
CGTATTTGCCGATGCTTGATAGTATGCGTAACACCATTCCTGATAAGTGCTTTCCTGCGGCGCATGCCAGTAGCGGATTT
GCGCTGTTTGCCTTTGCGTTTGCGCCAAGTTTGCGCCGCCGTCGTGGTGCGATCATCATCGTGGTGATGGCATTGGGCTG
GGCGATGGGCTGCTATAAGATGATTATTGGCGATCATTTTTTGAGCCATACGGTGGTGTCGATGATGCTTGCGTGGGCGA
TGTCGGCAGGGCTTGCGTGGGTGTTTTTTAAGAAGGGTGAACAATTCAAGGAAAAAAAATCAACCCCAAACAGAAATAAC
AGCTACAAAAATGGACAGTAATAATGCAATAAAAGTAATGTTGAGCCATATGTTGGACGAGTCTTTTAAGACTAATGAGG
CTGTTAATGCCATTGTAATGAAACTAATGATAATAATGGCTGCAAATAAATATTGTACAATGTTTTTTATTGTACTTGGT
TGCTCCCCAATTCTTTCTCTGTAATTTGCATCAACATTAGATATCCATTGAAGTAATAGAGCAATAAAACAGCCTACTCC
GCAAACGGTGAATTGCACAGACGTCGGGTTTGAATCAAACAACGAGATCGCTAAGTTATATAAGTATACCGCGCCTGAAA
TCACCCCGACAACTAAAATAGAGATGAGTATAATCCGAAGAACTAATGTATTGCTTTCCTTTAAGGAATATATTAAAGCC
GCTTTTTCGTATTCTTTCATGATTTAATCCAGTCTATAAGTATAAGAACCAACAAAACTGTCCAGGATTCATTTTCTGTG
AGCATATGAAATCCCATCCAGAGTGATGATACTGGCAGAAACACGCCCACAGCCGGAATTATTAGTCTGGCGGAGAATAT
CCTTATCAGGAGTATCAATAGTGATAAGAACAGAAAAAGAGTGATCATGTGGAAAAACATTCACATCACCTATAACACTA
TTAAGATAGCCTGCCAGATATCCATTTCAGACAGGCTATATACAGGGTTCAATTCATTCTATGAAAGGTGTATAGGGATT
GCTGATGTTTGTGTCGATCAAAAATCTAATATTAATAGATGAGAACTAATATACATATTTCCAGACGGATGGGGGATTT
TATCGTAGAGTTTATTCATTGTGAGTTCTGCAAGCCTGTGATCGGCGGCGGCATTAAACGACTCAAGCTCAGAAGGAGGG
AGGCTGTTTTGGTTTTTTTCAATAATCTTTTCAAGTGTGTCGAGTGTTGAGCAACGTCTTAACTGAAAGAGCCAGTCTTG
TTTTGTTTTCATTGTACAATTCCTGAAGAACATCCTTGTCCTATAGTAGCTACAACGATGTTTGATTTCTACTGCTGGAT
TTATTTCTTACTTTTTTGTGTAAACTCAAATCCCAGTTTCCCGGTTGCTTTGTCAACCAGTTTTACTTTTGCTGAAAATT

CTTTGTTTGTTTTTTGACTAATGAAACCTTTCAATTCACTTGTAACTCCTTTTGTTATTAACGTTTCAATTTGTTTATCG

GTTAATGATTTGCCGCAGAAATCTTTCCATATCTTAAATTCACATCCTGTACAGAAATAACCTTTTGGTCTGATAACAAT

CTGTTTACCACAGGAAGGACAAGGAGAGGGCAGGCGTTGTGACTGACCAGACGGCGCGCTGGGGGCAACTTTTATTTCAG

CACTGTTCGCATTACTAATCATCGGTATCAGATCATTGTACAGTTCGTCAACGAACTGTTCGATGGTCATTTCTCCGTTT

TCAATGAGTGTCTGTTTTTCCGCCCATAATGCCGTCATATCTGGATTAACAGCTATATCCGGCAGAGCATCAATAAGCGC

ATACCCGGTATCAGTCGGAATAAGTTTTCCTTTTTCCAGGGTGATATAGTTCCGTTTTTTCAGTGTTTCCAATATGGAAG

CCCGCGTAGCTGGCGTACCAATACCGCCATGTTCGTCTTTTTTATCCCGATCTTTATCCTTCAGTAACTTTTTAATCACT

GGATCAGTGACAAAATCTGCGACACGAACAAGCGCGGCAAGTAAAGTGGCTTCGGTGAATAATGGCGGTGGCGTTGTTTT

CTTCTCATTCACAACCACTTCTTTTGTCGTCACTGTTTCTCCTGTGCGAATTTTACAGAGCAGATCAAAAGCGGAATCAT

CATTTTCGGCTTCTGATTCCTCATCCCCGGTATTTTCCTCACCAAGAAACGCTTCAAATCCGCTGTCTGTTGTTTTCCTG

GCACGGGCATAAAACGACTCATCACCACACTGAATGGCAACGGATACTTCCTGGTATGTTTTTTCAGGCATGAACTGAAC

AAGATAGTGTTGTGCGATTGCCAGGTAAACATTGCGCTCGTCACTGCTCAGCGCGTTTACATCCGGTACGTTAACGGTCG

GGATTATCGCCGTATGCGCAGTCACTTTCGCACTGTTAAACGCCTTGCTTTTACGTGTCGAATCAACGTCCAGTGGCTGA

TCGAATACTGACTTCAGGGCATCGAGAACCTGTGGTGCTTCGCTGAATTGTTCATCAGAAAGATATGAGCAATCTGAACG

GTTGTAAGTAATTGCTTTGTATTTTTCACGTAGCTGCTGTGTGATATCCAGCGTTTGTTGTGCCGTCATTTTGAACTTCT

TGTTCATGTACTGCTGAAGCCGGACCAGGTTAAAGGGCAATGGCGCAGCCGTTTTTTTATCGTCTGTTGCTGCTGCTTCA

ACTGTCGCTGGTTTTCCTGCAAGGGAAGCCGCTGTTCCATTCGCCCACGCCTTATCAAGTAATTTACGGTCTGTCAGTGG

TGCGAACTCACCAGGTTTCCAGTTCGCCCTGATAACATCAACGCCGCGTTGAAAATGCCCGGTCATGGTGTAGTAGAAAC

TGGATTTATGATTCTTGTTGGCACGGGTGCGATTTACTATCAGGCCAAGAATAGGCGTCTGAACACGCCCGACCGAAAGT

GTCCCTTTATAGCCTTTAGCCCGTGCCGGGATCGTATAAGCACGCGTCATGGATAAACCGTATATCGCGTCGGCTACAGA

ACGCGCCAGAGCCTTGAGATAGAGTCCTCTGAAGTCACGGTTATTTTTGGGATTTGCCAGTGCCTTTTTCACTGCCGGAA

GGGTGTTGTCGTTGATCAGAACGCGCTTAACGGGTTTTGTGTTGCCTGCATATTCCAGGACCTCATCGACAAGTAGCTGT

CCTTCGTCATCTGGATCGCCAGCATGAATAATTTCGGTCACATCGGCACGCCTGATAAGCTCAATAATCGTTTTAACTTG

TTTTGCGGCGCTTTCTACTGGCTGGTATTTTACGGGATAAAGACGCAAAGGGAGTGTTTCAATCTTCCACTCTTTGTATT

CAGGATTATAGTTTTCGGGGGGCTGTGATTCGATAATATGACCAAAACAGTTAGTCACAATAGTATTATCACTTTCAAAC

CAGCCATCATGACGGGTAAAATTGCCACCAAGCGCCTTAACAATATCATTCGCTACTGCGGGTTTTTCTGCGATAAAAAG

TTTCATTTTGTTATCCTTTACATTGAATTAATCTTTTGGCACAACGGATAGCACGTTCTTCTGATACTGCCTGTTGCCAG

TTATCTTCACTTACTGGATGTCTGAATCTGCGTTCACAGCCATTAAAAGCAAAAATCCCCTCACGGTTAAGTATATTAGT

TGCAATATCCTGGTCTTTAATTCTCAAAAAGGCTGTTACGGCAAAACCAGAATACGAAGAAATTTTTGAAATGATGCATT

CATCAGCTTTTATTAACGCGGCAAAAGGAACTATCTTTTGATGACCACACGAAATCAGAGGGAGTGTGTGAATATCATAA

TAAGCTATTGAATGACAATCCATGCGGGTATCATCAATAGCTGCCAGCCAGAATCCTGTCTGGTCTTTTATTTTTCTGCT

AATATCTGAATCTGAAACACCAGGATTTAATAAATATCCACCATAACAGACATGGAAGGGGTGAGCAGATTCATCAATAT

CAGGAAGTTCTTTTACCGGATTTTCTTTCCAGTCCTTCAGAACACCGAATTCAAGAGCATCCAGCAAAAACTTTCGTTCA

AATCCATCAAGCAATGCATATTGCATGTTAAACCTCTCAAAGAAGCTCTGATTTGTTGACCTCATGGTGAACCAGTTCAC

CATTGCGTAAGACAAACAGGTAAGAAAACTTTCTTTCCTTATCTTTCAGTCAGTTTTTGTATTCAGAAAGAATAAACACC

CAGTCATCGGTGCTGTAATGAGATGCGATGACTCATTATCTTTTTAGTGATGAGTTGATTATTTTTAAAATATGCCTGCC

TTTAATTAGCATGTTTCTGGAAACAGCCTGATTACGAATACCTCTCATCTGGTCAAACTGATAGGTTAAATCATGTTCTT

TTCGTGAAAGTCCAAGAGTGGCTCTTTCATTATCCATCAGGACTGAAATTGCAATATATTCTCCAGATGCACACCAGTCT

GCATAACTGGCAACGCAATGTTCCATCTCCTGACCTTCCTGACACAGAGCATCAAATGTAATTAATTCATTTACACCGTT

ATCAACTTTCTGCCAGTTTATCCCAGTTCCTTTCCATTTTGGGATTACGGGAATAACGTTATTCTTCACTTGTCGCGTCC

ATTCATCAGATAGTCTCCAGAAAGAAGGCCACTCCTGGTTTTTATGAATTAACCGTTCTTCTGCTAACAACCAGTCAATT

GCATGACACAACTGATTCGTTTCCATTTCCCAGCGTGATGTTTGCCTGGATTCAGTATTACGGTCATAAAAGCCAATGTT

TTTAAATAAATCACGATGATATTCTAGCCATTTTGAGCATATACGATAAATATCATTTTCTTTTTCTGAGTAAGCTCGAA

TGCTAATATAATCGATAATCCAGTAAATTGCTCTCACAGGATAGTTCTTGATAAGTGGATGGCGTAATAATCTGACTGTA

AATCTGAATGATGCATTATCATGTTTATCATTTAAAGCATATACTAATGACAAACTAACATGTCGTAAACTTCTGAACTC

ACCATAACTAAGTTTGCAGGGAAAACTGATTTTTTCATATAACTCTTTTGTCTTTCCAGATAGTATAAACTGACCATTTT

TATGTTGTTGTCTTAGACGACCAACCATAGGCCACAATCCGAAAAAATCCATATCATTCACAATATGGATTCGCTTTGAA

AAATCCAGATAGTCCTCAAATAATGCCTGACGAATTGAATTGTAAGCTATCGAACAATGTGAAGCTCTGGCATATTGTGC

AAATATCTTTTTATCTACCATTATCCATATAGCTTTACTCAATGCTTTTGAAGCATTAAATTCATTATCCACCCCTATAA

TTTTTAATGCTCTCTGATAAATTTTGTTTTTTATCTCTGCGAGTAAAATATGTCTGCTGATATATCGTGTATTATAGGGG

TAGTTGAATGATGCTATTTTACCAAGGCAATGAACAATGTCATGTATTCCATACTCATATGAGTAATATACATTGGATGG

ATATAGTGTGGAAATATAACGACTCTCACTGTTTTCCTCTTCCTCTCCAATGCGTAACAAATAGAGTTTGTCTTCAATTG

AATATCCAGCCTCAATCAATGATTTGATGATGTAGCTAATCGCATATTTTAACCAGTCGTCCATGTCGTATGCAGATTTT

TTAGACCAGAATAAAAAAATCTTACCAGGAAATGAATTTCTTCTGGTCCATACCCCAGTGTGCTTATCTCTTAGCTTGTA

TCCCCACAAAGTGATTTTGTTATTCTTTTTGTTTTTTCTAATACGAAAAACGCCACAAATGATAATGGAGATGTACTTTT

CATTCTCCTCTGTTTTTACAAGACGAAGCCCCCCTGTATTTTGATAGATTTTAAAAACATCATGCAACGTAAGAATATTA

TTGTCCCTTGTCTGTTGATTAATTACATACATGCCGCCCCCTGTTAAAAAGAAGCCTTTAATATTGCACAAAGATAAAAA

GACAGAATCATGGCAAACCAAAAGAAGAATGCATTTTCTTGCCCCCTTAATTTCTGATACGCCATTCCCACGGTGGAACC

GGATTTGCATCGGCCCACAAACCACGTTTTTGTTCCTGCGCTTCCCGTTGCAGAGCCGGTAATGACTCATCCACGTTGTA

ACGTTCATATACCCATGCAGCGCCAGACTGAACCATAAAGCGGCTGGCATCCGTGCCGTTCGTCGTGAACACATGACCGA

TAATGCGTCCGTAGCGGTCTTTATGTGAATAAGAGACTGTCACCTGTTTACCTGCAACCAGAGTTTTAAGCTGACTAGTT

GACCAGCGTCCAAAAGGTTGTTTCTTTTCAGGGGCATCGATGTTTATCAATCGGACTCGGATCGGAACTTCATACACAAC

AATTTTTGCGGGTAGTGTTTTAATTTCTATGGTGTCGCCATCGAGAACGCGAATAACTTTCCCCTGAAGAATTTTCTGGT

TAAATGTATAAGGTATTCCACCTGCATTTGCAGACTGAAAAGCGGTAAGCAGATAAATAAATATTGTTAATAAGATTTTC

ATAAAAGATTAACGTTAATTTGTCTGTGCAATGGATAGAGCGTTATTCTTATTTTCTTCCTGCCGCTTCATCGAAAAATG

ATGATTTTCCTACTGCTGCGGCATCATACTGAAATGAATATCCAAAATCATCGTCATGCTCAAAACCTGTATTTTCTCCA

TTTTTTAGTCTGGAAAGAATAATCTCAAGTTGACTGATTATATCTTCCCTTTCACTATGACGAGTGCCTTGTAAGAGAAT

AGTTACTTGTTGCAAAGGATATGCATGTTCAATCCATGCCTGGCTAGGTCCAGAGTGCGTTGTATATAAATTTTTTTGTG

CCGAAATATTATTCAGAAAACAGTTTAAGTCATCATTGGAATAAATTTCTTTAATATTGTTGATGTTGGATGTTAGTTTT

CCAAGTTGGAATGGTGGAGAATTTTTGTCTTCATTGTTGTCTCCTTTATCGTATTCTGGAAACACAATTAACAAAGGTGC

ACCATTAGTTCTCTTCATAAAACGGGTTACTAGCCGCCCAATATATTTATTGGACATATGCTGTTCAATGTCCAGGACAG

CATATTCCTCCGCTGGGAACATTTTGTTTTTTTCGATGGATTTAATTTTTCTGGTAAGATGAATGTTTCTGATAAGCAAA

CCTACTATCAGGAAGTAAATAAATATCCAGAATAAAAAATCACTCTGTAGTTGTTCTATCATAAGTGAATCCTCTAATTT

GTTAATGGACAAATCATTACCCGTTCATCAATGTTTGTAGGAAGTTCGCCAGGAGTAGGGACGCGTTCTCCTCGCTGGGC

GGCTTGTGCCAGTGTTCGTAGCAGCAACACGCTGGCATGAAATATGGCCTGTTCTTTTGTGGCTGCGCTTGTTTCCAGGT

CGAAATCACTCAGAGTCACAACGACTTGCCCGTTTCTGTTCTCGACCGTTGCCGGGTAGGGAACAAAATAGGGAGCGATT

TTGCTGGTTTGCTTGCGTCGGGCATCGAGTTTACTGACAAGTTGATAAGCCCGAAGGTGTGTGTATCGTTTCAACATGTT
CAGGGAGCGATGGCCTGAAATAGCGGCAACCTCAATGACGTTCAATGTACCAAGTTCGAAAAATCGGCTGATAGCCTCAT
GACGGAGGTCATGAAAATGCAAGTTTTCTATCTTCAGTTCCTGAAGTGCTGTTCGCCAGGCACTTTTGAATCCCGAAGAG
GTATAAGAAAAAATATTACCGTTAAGCTGTGTCGGCAGCATTTGCAGGTAATTACGGGCTTTCCTGGATAGCGGAACATC
GCGTGGAGCACCGTTTTTGGTTGTTGGAAGATGAGCTACCCCATGTTGCAGATCGACGTGTTCCCAACGCAATGACAAGA
TTTCACCTTGTCGCATTGCTGTTTCCAGCGCCAGATGAAAAATGACGTAAAGAGCCTGATTTTTTTCTTTGAAATACCGC
GAAAGTCGGCGTTCTTCTCCTGAAGTCAGGCGACGATCGCGCCCGCTACTGATTTTGGGTTTTCTGACCAGTTCGACAGG
ATTCATGCGGCATGTTCCCCATTCCACACGGGCGATATTGAATAATGATGACAACAGGGCAAGTTCAAGACGGACGGTGT
TTCCGGTTATTTGACGCCCCGTTCGTGGGTTAATTTGCGCCAACCGTTGATCCCGATAATTGGCAATATCAACAGTGGTA
ATGTCATCCATATAGCGTTCAGCAATAGGGTGACGTTTAATCACATTTACGCGATAAAACTCCTGTAGATGGCCTTTTTT
GTGGATGGATACTGTCTTCAGGTATTTGTCCAGCGCCCGGCTGATTGTCATTTTGCGAATTTTGATTTTCTGAAACATAT
CCCCTCCAGACAAAAAAGGGGGGAAGGTAGAGATTCTTGCTTCGAAATGCAATATCACCGTGCCAATCCGGTGTGTGGAA
AAAAGGAGGAGGCATAGGGAAAGTCTCCTCCTATCAGCTTAATTTCCCATACTCTCAGTCAAACTTGGGGCAGCATACTT
TTTGCGCATTATCCCATGTAAGAGCAACGGGTTTCCATAATTCAAACGATTATTCAGAATGCTCGTTATCTAAATCCGGA
TCAAACTGGCAACTAACAGCTTACCCACCTTCAATAGCTAGTTATCGTTGTGATGCAGTATGTATTGATTAATCATAGAA
ATAGGGGTGCCAATCCGGTACGTGGAGAAGGGCATCAGGTAGCACTGTTCTGACAGGAAAAATAGCAAATGGACAACAGA
TCCCATTGCCATCTGGTTTTTCTGCCAGCCAGTGTACCTGGAGTGTCAGTAATGCAGAAAATCCGCACGGGTGGAAGCCG
AATTATTTTGCGGGGTCAGTTGCAACCTATGATGCCAATCGAATTGTGAAATGCGGCTTCTATGACGAATACAACTTTTA
TGGCGGTACTCACCGAACTGACCTGTCGGGGAAATGTAGTTACATCGTGGTTTGCCAGTGATAAGTATCGTCACTGGCAA

ATGGCGTAAACTGTAATTGAACCATTTTGATCTTCGGTCGTGTCGCAACTGGCAGTCCATGCATTGTTGCCTGAAGGCAT
GGAACGTGTGAGCCATATAAATCCGGTATTTGATCTGCATTGACCGCCTCCACCAATGACTTTCTTGCCAGATGGACATG
TTGCCGTTGCTCCAGGCCAACGCCACGCAGTAGCTGAAGCGGTAACAATTTGGCTATCACCTGCGCCAATTTTTTTCCAC
ACACCGGATTGGCACGGAGGCCAGTGCCAATCCGGTATCTGGACAACGGCAAAAGTGAACTTCACTACCAGTACGTACAA
TATCGGGAAAAACACCCGAAATCTGAGCATTGGCGTACATGCGTACTGTTCGTGGACATACCTTAATGGTGCGCCTTTTG
GTGGGTTTCAGCAGGTATACTCCGACCAAAACAAGGTTTGGTATGTGAATAATTATGCGTGGGGAAATTATGAGTCCGGC
GGCACAATTACTGTTACATGCCTGAATCTTCCTGGTGCTGGGATTTAACCGAAGGGGCAACAATAAGCATCAACGTGTTC
CTCATCAACGCCATCACCAATGCCATACATGCGTGTACCTGTCATGATAAATCCGGTCGGGCATTGAATGGGTTTGTAGT
AGATAGATCCTCCACTTTTCCCGCCAATGAAGTGATTCATTGCTACAGATGATTGATACCACTTACAGGCGGAGTAGTTC
ACTTTATTGCCACCAGACCACCGACCGGATTGGCACTGGCCTCCGTGCCAATCCGGTGTGTGGAGAACTTCCGGTTCCTC
TAACGGTAGTTACAGTAATCTTGGATCACACCGAGGATCATTTACTGGTCGCAATACGGGAAGTGGTACGCTGTTTGTTT
ACGCCTCTGGAGGGAATGGTGGTTCCGCCGGGGGAGATTGTGCTAACACATCCAGATTGCAGGGATACGTTGCAGGGGCA
TTGATTAGCACAAATGCGAGCAACAACCCATCTTATGGGAAGACAGCATTCATCTCTTTTGCTGTCCCTGCTGGCGCAAC
TTATCAGATCACCTCTTATCCGGCCCAAAACTACTCTTGTGGCTCAGGGGTATTTTCAGTATTTGGATACCAAACTTAAT
TGAGAGTTACACAGGACGCAATACAACCTGTCTTGTGCTCGTATTTGTACCATTTTCGTGAACCAGATGGACTCTCGACT
ACCTGGCAGTAATGAGAATCCTCAGCATTTCCCATTCTGGCAATGGCACAAAAGTCAAATTGCCCCAAGTAACCAGTGCT
GGAAAGTTGAGTAACCTTGAGCTTTCCACCTAATGCCCTCCACACACCGGATTGGCACGGAGGCCAGTGCCAATCCGGTC
GGTGGTCTGGTGGCAATAAAGTGAACTACTCCGCCTGTAAGTGGTATCAATCATCTGTAGCAATGAATCACTTCATTGGC
GGGA

>NG_064789.1 Escherichia coli sc12-96 mcr-1 gene for phosphoethanolamine--lipid A transferase MCR-1.18, complete CDS

CGCATAATTTTTATATCAGATAAATTGTACTGGATTTCTTAAAAAATTGCAGTATAATTGCCGCAATTATCCCACCGTT

TATTTTTTGAGTAGTTTCTCATGATGCAGCATACTTCTGTGTGGGACCGACGCTCGGTCAGTCCGTTTGTTCTTGTGGCG

AGTGTTGCCGTTTTCTTGACCGCGACCGCCAATCTTACCTTTTTTGATAAAATCAGCCAAACCTATCCCATCGCGGACAA

TCTCGGCTTTGTGCTGACGATCGCTGTCGTGCTCTTTGGCGCGATGCTACTGATCACCACGCTGTTATCATCGTATCGCT

ATGTGCTAAAGCCTGTGTTGATTTTGCTATTAATCATGGGCGCGGTGACCAGTTATTTTACTGACACTTATGGCACGGTC

TATGATACGACCATGCTCCAAAATGCCCTACAGACCGACCAAGCCGAGACCAAGGATCTATTAAACGCAGCGTTTATCAT

GCGTATCATTGGTTTGGGTGTGCTACCAAGTTTGCTTGTGGCTTTTGTTAAGGTGGATTATCCGACTTGGGGCAAGGGTT

TGATGCGCCGATTGGGCTTGATCGTGGCAAGTCTTGCGCTGATTTTACTGCCTGTGGTGGCGTTCAGCAGTCATTATGCC

AGTTTCTTTCGCGTGCATAAGCCGCTGCGTAGCTATGTCAATCCGATCATGCCAATCTACTCGGTGGGTAAGCTTGCCAG

TATTGAGTATAAAAAAGCCAGTGCGCCAAAAGATACCATTTATCACGCCAAAGACGCGGTACAAGCAACCAAGCCTGATA

TGCGTAAGCCACGCCTAGTGGTGTTCGTCGTCGGTGAGACGGCACGCGCCGATCATGTCAGCTTCAATGGCTATGAGCGC

GATACTTTCCCACAGCTTGCCAAGATCGATGGCGTGACCAATTTTAGCAATGTCACATCGTGCGGCACATCGACGGCGTA

TTCTGTGCCGTGTATGTTCAGCTATCTGGGCGCGGATGAGTATGATGTCGATACCGCCAAATACCAAGAAAATGTGCTGG

ATACGCTGGATCGCTTGGGCGTAAGTATCTTGTGGCGTGATAATAATTCGGACTCAAAAGGCGTGATGGATAAGCTGCCA

AAAGCGCAATTTGCCGATTATAAATCCGCGACCAACAACGCCATCTGCAACACCAATCCTTATAACGAATGCCGCGATGT

CGGTATGCTCGTTGGCTTAGATGACTTTGTCGCTGCCAATAACGGCAAAGATATGCTGATCATGCTGCACCAAATGGGCA

ATCACGGGCCTGCGTATTTTAAGCGATATGATGAAAAGTTTGCCAAATTCACGCCAGTGTGTGAAGGTAATGAGCTTGCC

AAGTGCGAACATCAGTCCTTGATCAATGCTTATGACAATGCCTTGCTTGCCACCGATGATTTCATCGCTCAAAGTATCCA

GTGGCTGCAGACGCACAGCAATGCCTATGATGTCTCAATGCTGTATGTCAGCGATCATGGCGAAAGTCTGGGTGAGAACG
GTGTCTATCTACATGGTATGCCAAATGCCTTTGCACCAAAAGAACAGCGCAGTGTGCCTGCATTTTTCTGGACGGATAAG
CAAACTGGCATCACGCCAATGGCAACCGATACCGTCCTGACCCATGACGCGATCACGCCGACATTATTAAAGCTGTTTGA
TGTCACCGCGGACAAAGTCAAAGACCGCACCGCATTCATCCGCTGATTTCTCCCTGTATTTTTCCAAACCCACCGCACA
CTCCATTCGTATTATGGGCGGTGGGGTGGGGTTTGTTATGCCGTATTTATCAAATAAACGCCTACT

>NG_064788.1 Escherichia coli ECK2 mcr-1 gene for phosphoethanolamine--lipid A transferase MCR-1.17, complete CDS

ATGATGCAGCATACTTCTGTGTGGTACCGACGCTCGGTCAGTCCGTTTGTTCTTGTGGCGAGTGTTGCCGTTTTCTTGAC
CGCGACCGCCAATCTTACCTTTTTTGATAAAATCAGCCAAACCTATCCCATCGCGGACAATCTCGGCTTTGTGCTGACGA
TCGCTGTCGTGCTCTTTGGCGCGATGCTACTGATCACCACGCTGTTATCATCGTATCGCTATGTGCTAAAGCCTGTGTTG
ATTTTGCTATTAATCATGGGCGCGGTGACCAGTTATTTTACTGACACTTATGGCACGGTCTATGATACGACCATGCTCCA
AAATGCCCTACAGACCGACCAAGCCGAGACCAAGGATCTATTAAACGCAGCGTTTATCATGCGTATCATTGGTTTGGGTG
TGCTACCAACTTTGCTTGTGGCTTTTGTTAAGGTGGATTATCCGACTTGGGGCAAGGGTTTGATGCGCCGATTGGGCTTG
ATCGTGGCAAGTCTTGCGCTGATTTTACTGCCTGTGGTGGCGTTCAGCAGTCATTATGCCAGTTTCTTTCGCGTGCATAA
GCCGCTGCGTAGCTATGTCAATCCGATCATGCCAATCTACTCGGTGGGTAAGCTTGCCAGTATTGAGTATAAAAAAGCCA
GTGCGCCAAAAGATACCATTTATCACGCCAAAGACGCGGTACAAGCAACCAAGCCTGATATGCGTAAGCCACGCCTAGTG
GTGTTCGTCGTCGGTGAGACGGCACGCGCCGATCATGTCAGCTTCAATGGCTATGAGCGCGATACTTTCCCACAGCTTGC
CAAGATCGATGGCGTGACCAATTTTAGCAATGTCACATCGTGCGGCACATCGACGGCGTATTCTGTGCCGTGTATGTTCA
GCTATCTGGGCGCGGATGAGTATGATGTCGATACCGCCAAATACCAAGAAAATGTGCTGGATACGCTGGATCGCTTGGGC
GTAAGTATCTTGTGGCGTGATAATAATTCGGACTCAAAAGGCGTGATGGATAAGCTGCCAAAAGCGCAATTTGCCGATTA

TAAATCCGCGACCAACAACGCCATCTGCAACACCAATCCTTATAACGAATGCCGCGATGTCGGTATGCTCGTTGGCTTAG

ATGACTTTGTCGCTGCCAATAACGGCAAAGATATGCTGATCATGCTGCACCAAATGGGCAATCACGGGCCTGCGTATTTT

AAGCGATATGATGAAAAGTTTGCCAAATTCACGCCAGTGTGTGAAGGTAATGAGCTTGCCAAGTGCGAACATCAGTCCTT

GATCAATGCTTATGACAATGCCTTGCTTGCCACCGATGATTTCATCGCTCAAAGTATCCAGTGGCTGCAGACGCACAGCA

ATGCCTATGATGTCTCAATGCTGTATGTCAGCGATCATGGCGAAAGTCTGGGTGAGAACGGTGTCTATCTACATGGTATG

CCAAATGCCTTTGCACCAAAAGAACAGCGCAGTGTGCCTGCATTTTTCTGGACGGATAAGCAAACTGGCATCACGCCAAT

GGCAACCGATACCGTCCTGACCCATGACGCGATCACGCCGACATTATTAAAGCTGTTTGATGTCACCGCGGACAAAGTCA

AAGACCGCACCGCATTCATCCGCTGA

>NG_064787.1 Escherichia coli EC32 mcr-1 gene for phosphoethanolamine--lipid A transferase MCR-1.16, complete CDS

ATGATGCAGCATACTTCTGTGTGGTACCGACGCTCGGTCAGTCCGTTTGTTCTTGTGGCGAGTGTTGCCGTTTTCTTGAC

CGCGACCGCCAATCTTACCTTTTTTGATAAAATCAGCCAAACCTATCCCATCGCGGACAATCTCGGCTTTGTGCTGACGA

TCGCTGTCGTGCTCTTTGGCGCGATGCTACTGATCACCACGCTGTTATCATCGTATCGCTATGTGCTAAAGCCTGTGTTG

ATTTTGCTATTAATCATGGGCGCGGTGACCAGTTATTTTACTGACACTTATGGCACGGTCTATGATACGACCATGCTCCA

AAATGCCCTACAGACCGACCAAGCCGAGACCAAGGATCTATTAAACGCAGCGTTTATCATGCGTATCATTGGTTTGGGTG

TGCTACCAAGTTTGCTTGTGGCTTTTGTTAAGGTGGATTATCCGACTTGGGGCAAGGGTTTGATGCGCCGATTGGGCTTG

ATCGTGGCAAGTCTTGCGCTGATTTTACTGCCTGTGGTGGCGTTCAGCAGTCATTATGCCAGTTTCTTTCGCGTGCATAA

GCCGCTGCGTAGCTATGTCAATCCGATCATGCCAATCTACTCGGTGGGTAAGCTTGCCAGTATTGAGTATAAAAAAGCCA

GTGCGCCAAAAGATACCATTTATCACGCCAAAGACGCGGTACAAGCAACCAAGCCTGATATGCGTAAGCCACGCCTAGTG

GTGTTCGTCGTCGGTGAGACGGCACGCGCCGATCATGTCAGCTTCAATGGCTATGAGCGCGATACTTTCCCACAGCTTGC

CAAGATCGATGGCGTGACCAATTTTAGCAATGTCACATCGTGCGGCACATCGACGGCGTATTCTGTGCCGTGTATGTTCA

GCTATCTGGGCGCGGATGAGTATGATGTCGATACCGCCAAATACCAAGAAAATGTGCTGGATACGCTGGATAGCTTGGGC

GTAAGTATCTTGTGGCGTGATAATAATTCGGACTCAAAAGGCGTGATGGATAAGCTGCCAAAAGCGCAATTTGCCGATTA

TAAATCCGCGACCAACAACGCCATCTGCAACACCAATCCTTATAACGAATGCCGCGATGTCGGTATGCTCGTTGGCTTAG

ATGACTTTGTCGCTGCCAATAACGGCAAAGATATGCTGATCATGCTGCACCAAATGGGCAATCACGGGCCTGCGTATTTT

AAGCGATATGATGAAAAGTTTGCCAAATTCACGCCAGTGTGTGAAGGTAATGAGCTTGCCAAGTGCGAACATCAGTCCTT

GATCAATGCTTATGACAATGCCTTGCTTGCCACCGATGATTTCATCGCTCAAAGTATCCAGTGGCTGCAGACGCACAGCA

ATGCCTATGATGTCTCAATGCTGTATGTCAGCGATCATGGCGAAAGTCTGGGTGAGAACGGTGTCTATCTACATGGTATG

CCAAATGCCTTTGCACCAAAAGAACAGCGCAGTGTGCCTGCATTTTTCTGGACGGATAAGCAAACTGGCATCACGCCAAT

GGCAACCGATACCGTCCTGACCCATGACGCGATCACGCCGACATTATTAAAGCTGTTTGATGTCACCGCGGACAAAGTCA

AAGACCGCACCGCATTCATCCGCTGA

>MK568463.1 Escherichia coli strain ECK2 phosphoethanolamine--lipid A transferase MCR-1.17 (mcr-1) gene, mcr-1.17 allele, complete cds

ATGATGCAGCATACTTCTGTGTGGTACCGACGCTCGGTCAGTCCGTTTGTTCTTGTGGCGAGTGTTGCCGTTTTCTTGAC

CGCGACCGCCAATCTTACCTTTTTTGATAAAATCAGCCAAACCTATCCCATCGCGGACAATCTCGGCTTTGTGCTGACGA

TCGCTGTCGTGCTCTTTGGCGCGATGCTACTGATCACCACGCTGTTATCATCGTATCGCTATGTGCTAAAGCCTGTGTTG

ATTTTGCTATTAATCATGGGCGCGGTGACCAGTTATTTTACTGACACTTATGGCACGGTCTATGATACGACCATGCTCCA

AAATGCCCTACAGACCGACCAAGCCGAGACCAAGGATCTATTAAACGCAGCGTTTATCATGCGTATCATTGGTTTGGGTG

TGCTACCAACTTTGCTTGTGGCTTTTGTTAAGGTGGATTATCCGACTTGGGGCAAGGGTTTGATGCGCCGATTGGGCTTG

ATCGTGGCAAGTCTTGCGCTGATTTTACTGCCTGTGGTGGCGTTCAGCAGTCATTATGCCAGTTTCTTTCGCGTGCATAA

GCCGCTGCGTAGCTATGTCAATCCGATCATGCCAATCTACTCGGTGGGTAAGCTTGCCAGTATTGAGTATAAAAAAGCCA

GTGCGCCAAAAGATACCATTTATCACGCCAAAGACGCGGTACAAGCAACCAAGCCTGATATGCGTAAGCCACGCCTAGTG

GTGTTCGTCGTCGGTGAGACGGCACGCGCCGATCATGTCAGCTTCAATGGCTATGAGCGCGATACTTTCCCACAGCTTGC

CAAGATCGATGGCGTGACCAATTTTAGCAATGTCACATCGTGCGGCACATCGACGGCGTATTCTGTGCCGTGTATGTTCA

GCTATCTGGGCGCGGATGAGTATGATGTCGATACCGCCAAATACCAAGAAAATGTGCTGGATACGCTGGATCGCTTGGGC

GTAAGTATCTTGTGGCGTGATAATAATTCGGACTCAAAAGGCGTGATGGATAAGCTGCCAAAAGCGCAATTTGCCGATTA

TAAATCCGCGACCAACAACGCCATCTGCAACACCAATCCTTATAACGAATGCCGCGATGTCGGTATGCTCGTTGGCTTAG

ATGACTTTGTCGCTGCCAATAACGGCAAAGATATGCTGATCATGCTGCACCAAATGGGCAATCACGGGCCTGCGTATTTT

AAGCGATATGATGAAAAGTTTGCCAAATTCACGCCAGTGTGTGAAGGTAATGAGCTTGCCAAGTGCGAACATCAGTCCTT

GATCAATGCTTATGACAATGCCTTGCTTGCCACCGATGATTTCATCGCTCAAAGTATCCAGTGGCTGCAGACGCACAGCA

ATGCCTATGATGTCTCAATGCTGTATGTCAGCGATCATGGCGAAAGTCTGGGTGAGAACGGTGTCTATCTACATGGTATG

CCAAATGCCTTTGCACCAAAAGAACAGCGCAGTGTGCCTGCATTTTTCTGGACGGATAAGCAAACTGGCATCACGCCAAT

GGCAACCGATACCGTCCTGACCCATGACGCGATCACGCCGACATTATTAAAGCTGTTTGATGTCACCGCGGACAAAGTCA

AAGACCGCACCGCATTCATCCGCTGA

>MK568462.1 Escherichia coli strain EC32 phosphoethanolamine--lipid A transferase MCR-1.16 (mcr-1) gene, mcr-1.16 allele, complete cds

ATGATGCAGCATACTTCTGTGTGGTACCGACGCTCGGTCAGTCCGTTTGTTCTTGTGGCGAGTGTTGCCGTTTTCTTGAC

CGCGACCGCCAATCTTACCTTTTTTGATAAAATCAGCCAAACCTATCCCATCGCGGACAATCTCGGCTTTGTGCTGACGA

TCGCTGTCGTGCTCTTTGGCGCGATGCTACTGATCACCACGCTGTTATCATCGTATCGCTATGTGCTAAAGCCTGTGTTG

ATTTTGCTATTAATCATGGGCGCGGTGACCAGTTATTTTACTGACACTTATGGCACGGTCTATGATACGACCATGCTCCA

AAATGCCCTACAGACCGACCAAGCCGAGACCAAGGATCTATTAAACGCAGCGTTTATCATGCGTATCATTGGTTTGGGTG

TGCTACCAAGTTTGCTTGTGGCTTTTGTTAAGGTGGATTATCCGACTTGGGGCAAGGGTTTGATGCGCCGATTGGGCTTG

ATCGTGGCAAGTCTTGCGCTGATTTTACTGCCTGTGGTGGCGTTCAGCAGTCATTATGCCAGTTTCTTTCGCGTGCATAA

GCCGCTGCGTAGCTATGTCAATCCGATCATGCCAATCTACTCGGTGGGTAAGCTTGCCAGTATTGAGTATAAAAAAGCCA

GTGCGCCAAAAGATACCATTTATCACGCCAAAGACGCGGTACAAGCAACCAAGCCTGATATGCGTAAGCCACGCCTAGTG

GTGTTCGTCGTCGGTGAGACGGCACGCGCCGATCATGTCAGCTTCAATGGCTATGAGCGCGATACTTTCCCACAGCTTGC

CAAGATCGATGGCGTGACCAATTTTAGCAATGTCACATCGTGCGGCACATCGACGGCGTATTCTGTGCCGTGTATGTTCA

GCTATCTGGGCGCGGATGAGTATGATGTCGATACCGCCAAATACCAAGAAAATGTGCTGGATACGCTGGATAGCTTGGGC

GTAAGTATCTTGTGGCGTGATAATAATTCGGACTCAAAAGGCGTGATGGATAAGCTGCCAAAAGCGCAATTTGCCGATTA

TAAATCCGCGACCAACAACGCCATCTGCAACACCAATCCTTATAACGAATGCCGCGATGTCGGTATGCTCGTTGGCTTAG

ATGACTTTGTCGCTGCCAATAACGGCAAAGATATGCTGATCATGCTGCACCAAATGGGCAATCACGGGCCTGCGTATTTT

AAGCGATATGATGAAAAGTTTGCCAAATTCACGCCAGTGTGTGAAGGTAATGAGCTTGCCAAGTGCGAACATCAGTCCTT

GATCAATGCTTATGACAATGCCTTGCTTGCCACCGATGATTTCATCGCTCAAAGTATCCAGTGGCTGCAGACGCACAGCA

ATGCCTATGATGTCTCAATGCTGTATGTCAGCGATCATGGCGAAAGTCTGGGTGAGAACGGTGTCTATCTACATGGTATG

CCAAATGCCTTTGCACCAAAAGAACAGCGCAGTGTGCCTGCATTTTTCTGGACGGATAAGCAAACTGGCATCACGCCAAT

GGCAACCGATACCGTCCTGACCCATGACGCGATCACGCCGACATTATTAAAGCTGTTTGATGTCACCGCGGACAAAGTCA

AAGACCGCACCGCATTCATCCGCTGA

>MG198057.1 Escherichia coli strain SFV97 phosphoethanolamine transferase MCR-1.11 (mcr-1) gene, mcr-1.11 allele, complete cds

ATGATGCAGCATACTTCTGTGTGGTACCGACGCTCGGTCAGTCCGTTTGTTCTTGTGGCGAGTGTTGCCGTTTTCTTGAC

CGCGACCGCCAATCTTACCTTTTTTGATAAAATCAGCCAAACCTATCCCATCGCGGACAATCTCGGCTTTGTGCTGACGA

TCGCTGTCGTGCTCTTTGGCGCGCTGCTACTGATCACCACGCTGTTATCATCGTATCGCTATGTGCTAAAGCCTGTGTTG

ATTTTGCTATTAATCATGGGCGCGGTGACCAGTTATTTTACTGACACTTATGGCACGGTCTATGATACGACCATGCTCCA

AAATGCCCTACAGACCGACCAAGCCGAGACCAAGGATCTATTAAACGCAGCGTTTATCATGCGTATCATTGGTTTGGGTG

TGCTACCAAGTTTGCTTGTGGCTTTTGTTAAGGTGGATTATCCGACTTGGGGCAAGGGTTTGATGCGCCGATTGGGCTTG

ATCGTGGCAAGTCTTGCGCTGATTTTACTGCCTGTGGTGGCGTTCAGCAGTCATTATGCCAGTTTCTTTCGCGTGCATAA

GCCGCTGCGTAGCTATGTCAATCCGATCATGCCAATCTACTCGGTGGGTAAGCTTGCCAGTATTGAGTATAAAAAAGCCA

GTGCGCCAAAAGATACCATTTATCACGCCAAAGACGCGGTACAAGCAACCAAGCCTGATATGCGTAAGCCACGCCTAGTG

GTGTTCGTCGTCGGTGAGACGGCACGCGCCGATCATGTCAGCTTCAATGGCTATGAGCGCGATACTTTCCCACAGCTTGC

CAAGATCGATGGCGTGACCAATTTTAGCAATGTCACATCGTGCGGCACATCGACGGCGTATTCTGTGCCGTGTATGTTCA

GCTATCTGGGCGCGGATGAGTATGATGTCGATACCGCCAAATACCAAGAAAATGTGCTGGATACGCTGGATCGCTTGGGC

GTAAGTATCTTGTGGCGTGATAATAATTCGGACTCAAAAGGCGTGATGGATAAGCTGCCAAAAGCGCAATTTGCCGATTA

TAAATCCGCGACCAACAACGCCATCTGCAACACCAATCCTTATAACGAATGCCGCGATGTCGGTATGCTCGTTGGCTTAG

ATGACTTTGTCGCTGCCAATAACGGCAAAGATATGCTGATCATGCTGCACCAAATGGGCAATCACGGGCCTGCGTATTTT

AAGCGATATGATGAAAAGTTTGCCAAATTCACGCCAGTGTGTGAAGGTAATGAGCTTGCCAAGTGCGAACATCAGTCCTT

GATCAATGCTTATGACAATGCCTTGCTTGCCACCGATGATTTCATCGCTCAAAGTATCCAGTGGCTGCAGACGCACAGCA

ATGCCTATGATGTCTCAATGCTGTATGTCAGCGATCATGGCGAAAGTCTGGGTGAGAACGGTGTCTATCTACATGGTATG

CCAAATGCCTTTGCACCAAAAGAACAGCGCAGTGTGCCTGCATTTTTCTGGACGGATAAGCAAACTGGCATCACGCCAAT

GGCAACCGATACCGTCCTGACCCATGACGCGATCACGCCGACATTATTAAAGCTGTTTGATGTCACCGCGGACAAAGTCA

AAGACCGCACCGCATTCATCCGCTGA

>NG_057466.1 Escherichia coli 12-AB00501 mcr-1 gene for phosphoethanolamine--lipid A transferase MCR-1.13, complete CDS

ATGATGCAGCATACTTCTGTGTGGTACCGACGCTCGGTCAGTCCGTTTGTTCTTGTGGCGAGTGTTGCCGTTTTCTTGAC

CGCGACCGCCAATCTTACCTTTTTTGATAAAATCAGCCAAACCTATCCCATCGCGGACAATCTCGGCTTTGTGCTGACGA

TCGCTGTCGTGCTCTTTGGCGCGATGCTACTGATCACCACGCTGTTATCATCGTATCGCTATGTGCTAAAGCCTGTGTTG

ATTTTGCTATTAATCATGGGCGCGGTGACCAGTTATTTTACTGACACTTATGGCACGGTCTATGATACGACCATGCTCCA

AAATGCCCTACAGACCGACCAAGCCGAGACCAAGGATCTATTAAACGCAGCGTTTATCATGCGTATCATTGGTTTGGGTG

TGCTACCAAGTTTGCTTGTGGCTTTTGTTAAGGTGGATTATCCGACTTGGGGCAAGGGTTTGATACGCCGATTGGGCTTG

ATCGTGGCAAGTCTTGCGCTGATTTTACTGCCTGTGGTGGCGTTCAGCAGTCATTATGCCAGTTTCTTTCGCGTGCATAA

GCCGCTGCGTAGCTATGTCAATCCGATCATGCCAATCTACTCGGTGGGTAAGCTTGCCAGTATTGAGTATAAAAAAGCCA

GTGCGCCAAAAGATACCATTTATCACGCCAAAGACGCGGTACAAGCAACCAAGCCTGATATGCGTAAGCCACGCCTAGTG

GTGTTCGTCGTCGGTGAGACGGCACGCGCCGATCATGTCAGCTTCAATGGCTATGAGCGCGATACTTTCCCACAGCTTGC

CAAGATCGATGGCGTGACCAATTTTAGCAATGTCACATCGTGCGGCACATCGACGGCGTATTCTGTGCCGTGTATGTTCA

GCTATCTGGGCGCGGATGAGTATGATGTCGATACCGCCAAATACCAAGAAAATGTGCTGGATACGCTGGATCGCTTGGGC

GTAAGTATCTTGTGGCGTGATAATAATTCGGACTCAAAAGGCGTGATGGATAAGCTGCCAAAAGCGCAATTTGCCGATTA

TAAATCCGCGACCAACAACGCCATCTGCAACACCAATCCTTATAACGAATGCCGCGATGTCGGTATGCTCGTTGGCTTAG

ATGACTTTGTCGCTGCCAATAACGGCAAAGATATGCTGATCATGCTGCACCAAATGGGCAATCACGGGCCTGCGTATTTT

AAGCGATATGATGAAAAGTTTGCCAAATTCACGCCAGTGTGTGAAGGTAATGAGCTTGCCAAGTGCGAACATCAGTCCTT

GATCAATGCTTATGACAATGCCTTGCTTGCCACCGATGATTTCATCGCTCAAAGTATCCAGTGGCTGCAGACGCACAGCA

ATGCCTATGATGTCTCAATGCTGTATGTCAGCGATCATGGCGAAAGTCTGGGTGAGAACGGTGTCTATCTACATGGTATG

CCAAATGCCTTTGCACCAAAAGAACAGCGCAGTGTGCCTGCATTTTTCTGGACGGATAAGCAAACTGGCATCACGCCAAT

GGCAACCGATACCGTCCTGACCCATGACGCGATCACGCCGACATTATTAAAGCTGTTTGATGTCACCGCGGACAAAGTCA

AAGACCGCACCGCATTCATCCGCTGA

>NG_056412.1 Escherichia coli EC15-101 mcr-1 gene for phosphoethanolamine--lipid A transferase MCR-1.12, complete CDS

ATGATGCACCATACTTCTGTGTGGTACCGACGCTCGGTCAGTCCGTTTGTTCTTGTGGCGAGTGTTGCCGTTTTCTTGAC

CGCGACCGCCAATCTTACCTTTTTTGATAAAATCAGCCAAACCTATCCCATCGCGGACAATCTCGGCTTTGTGCTGACGA

TCGCTGTCGTGCTCTTTGGCGCGATGCTACTGATCACCACGCTGTTATCATCGTATCGCTATGTGCTAAAGCCTGTGTTG

ATTTTGCTATTAATCATGGGCGCGGTGACCAGTTATTTTACTGACACTTATGGCACGGTCTATGATACGACCATGCTCCA

AAATGCCCTACAGACCGACCAAGCCGAGACCAAGGATCTATTAAACGCAGCGTTTATCATGCGTATCATTGGTTTGGGTG

TGCTACCAAGTTTGCTTGTGGCTTTTGTTAAGGTGGATTATCCGACTTGGGGCAAGGGTTTGATGCGCCGATTGGGCTTG

ATCGTGGCAAGTCTTGCGCTGATTTTACTGCCTGTGGTGGCGTTCAGCAGTCATTATGCCAGTTTCTTTCGCGTGCATAA

GCCGCTGCGTAGCTATGTCAATCCGATCATGCCAATCTACTCGGTGGGTAAGCTTGCCAGTATTGAGTATAAAAAAGCCA

GTGCGCCAAAAGATACCATTTATCACGCCAAAGACGCGGTACAAGCAACCAAGCCTGATATGCGTAAGCCACGCCTAGTG

GTGTTCGTCGTCGGTGAGACGGCACGCGCCGATCATGTCAGCTTCAATGGCTATGAGCGCGATACTTTCCCACAGCTTGC

CAAGATCGATGGCGTGACCAATTTTAGCAATGTCACATCGTGCGGCACATCGACGGCGTATTCTGTGCCGTGTATGTTCA

GCTATCTGGGCGCGGATGAGTATGATGTCGATACCGCCAAATACCAAGAAAATGTGCTGGATACGCTGGATCGCTTGGGC

GTAAGTATCTTGTGGCGTGATAATAATTCGGACTCAAAAGGCGTGATGGATAAGCTGCCAAAAGCGCAATTTGCCGATTA

TAAATCCGCGACCAACAACGCCATCTGCAACACCAATCCTTATAACGAATGCCGCGATGTCGGTATGCTCGTTGGCTTAG

ATGACTTTGTCGCTGCCAATAACGGCAAAGATATGCTGATCATGCTGCACCAAATGGGCAATCACGGGCCTGCGTATTTT

AAGCGATATGATGAAAAGTTTGCCAAATTCACGCCAGTGTGTGAAGGTAATGAGCTTGCCAAGTGCGAACATCAGTCCTT

GATCAATGCTTATGACAATGCCTTGCTTGCCACCGATGATTTCATCGCTCAAAGTATCCAGTGGCTGCAGACGCACAGCA

ATGCCTATGATGTCTCAATGCTGTATGTCAGCGATCATGGCGAAAGTCTGGGTGAGAACGGTGTCTATCTACATGGTATG

CCAAATGCCTTTGCACCAAAAGAACAGCGCAGTGTGCCTGCATTTTTCTGGACGGATAAGCAAACTGGCATCACGCCAAT

GGCAACCGATACCGTCCTGACCCATGACGCGATCACGCCGACATTATTAAAGCTGTTTGATGTCACCGCGGACAAAGTCA

AAGACCGCACCGCATTCATCCGCTGA

>MG384739.1 Escherichia coli strain 12-AB00501 phosphoethanolamine--lipid A transferase MCR-1.13 (mcr-1) gene, mcr-1.13 allele, complete cds

ATGATGCAGCATACTTCTGTGTGGTACCGACGCTCGGTCAGTCCGTTTGTTCTTGTGGCGAGTGTTGCCGTTTTCTTGAC

CGCGACCGCCAATCTTACCTTTTTTGATAAAATCAGCCAAACCTATCCCATCGCGGACAATCTCGGCTTTGTGCTGACGA

TCGCTGTCGTGCTCTTTGGCGCGATGCTACTGATCACCACGCTGTTATCATCGTATCGCTATGTGCTAAAGCCTGTGTTG

ATTTTGCTATTAATCATGGGCGCGGTGACCAGTTATTTTACTGACACTTATGGCACGGTCTATGATACGACCATGCTCCA

AAATGCCCTACAGACCGACCAAGCCGAGACCAAGGATCTATTAAACGCAGCGTTTATCATGCGTATCATTGGTTTGGGTG

TGCTACCAAGTTTGCTTGTGGCTTTTGTTAAGGTGGATTATCCGACTTGGGGCAAGGGTTTGATACGCCGATTGGGCTTG

ATCGTGGCAAGTCTTGCGCTGATTTTACTGCCTGTGGTGGCGTTCAGCAGTCATTATGCCAGTTTCTTTCGCGTGCATAA

GCCGCTGCGTAGCTATGTCAATCCGATCATGCCAATCTACTCGGTGGGTAAGCTTGCCAGTATTGAGTATAAAAAAGCCA

GTGCGCCAAAAGATACCATTTATCACGCCAAAGACGCGGTACAAGCAACCAAGCCTGATATGCGTAAGCCACGCCTAGTG

GTGTTCGTCGTCGGTGAGACGGCACGCGCCGATCATGTCAGCTTCAATGGCTATGAGCGCGATACTTTCCCACAGCTTGC

CAAGATCGATGGCGTGACCAATTTTAGCAATGTCACATCGTGCGGCACATCGACGGCGTATTCTGTGCCGTGTATGTTCA

GCTATCTGGGCGCGGATGAGTATGATGTCGATACCGCCAAATACCAAGAAAATGTGCTGGATACGCTGGATCGCTTGGGC
GTAAGTATCTTGTGGCGTGATAATAATTCGGACTCAAAAGGCGTGATGGATAAGCTGCCAAAAGCGCAATTTGCCGATTA
TAAATCCGCGACCAACAACGCCATCTGCAACACCAATCCTTATAACGAATGCCGCGATGTCGGTATGCTCGTTGGCTTAG
ATGACTTTGTCGCTGCCAATAACGGCAAAGATATGCTGATCATGCTGCACCAAATGGGCAATCACGGGCCTGCGTATTTT
AAGCGATATGATGAAAAGTTTGCCAAATTCACGCCAGTGTGTGAAGGTAATGAGCTTGCCAAGTGCGAACATCAGTCCTT
GATCAATGCTTATGACAATGCCTTGCTTGCCACCGATGATTTCATCGCTCAAAGTATCCAGTGGCTGCAGACGCACAGCA
ATGCCTATGATGTCTCAATGCTGTATGTCAGCGATCATGGCGAAAGTCTGGGTGAGAACGGTGTCTATCTACATGGTATG
CCAAATGCCTTTGCACCAAAAGAACAGCGCAGTGTGCCTGCATTTTTCTGGACGGATAAGCAAACTGGCATCACGCCAAT
GGCAACCGATACCGTCCTGACCCATGACGCGATCACGCCGACATTATTAAAGCTGTTTGATGTCACCGCGGACAAAGTCA
AAGACCGCACCGCATTCATCCGCTGA

>KY780959.1 Escherichia coli strain LV23529 phosphoethanolamine-lipid A transferase MCR-1.9 (mcr-1.9) gene, complete cds

ATGATGCAGCATACTTCTGTGTGGTACCGACGCTCGGTCAGTCCGTTTGTTCTTGTGGCGAGTGTTGCCGTTTTCTTGAC
CGCGACCGCCAATCTTACCTTTTTTGATAAAATCAGCCAAACCTATCCCATCGCGGACAATCTCGGCTTTGTGCTGACGA
TCGCTGTCGTGCTCTTTGGCGCGATGCTACTGATCACCACGCTGTTATCATCGTATCGCTATGTGCTAAAGCCTGTGTTG
ATTTTGCTATTAATCATGGGCGCGGTGACCAGTTATTTTACTGACACTTATGGCACGGTCTATGATACGACCATGCTCCA
AAATGCCCTACAGACCGACCAAGCCGAGACCAAGGATCTATTAAACGCAGCGTTTATCATGCGTATCATTGGTTTGGGTG
TGCTACCAAGTTTGCTTGTGGCTTTTGTTAAGGTGGATTATCCGACTTGGGGCAAGGGTTTGATGCGCCGATTGGGCTTG
ATCGTGGCAAGTCTTGCGCTGATTTTACTGCCTGTGGTGGCGTTCAGCAGTCATTATGCCAGTTTCTTTCGCGTGCATAA
GCCGCTGCGTAGCTATGTCAATCCGATCATGCCAATCTACTCGGTGGGTAAGCTTGCCAGTATTGAGTATAAAAAAGCCA

GTGCGCCAAAAGATACCATTTATCACGCCAAAGACGCGGTACAAGCAACCAAGCCTGATATGCGTAAGCCACGCCTAGTG

GTGTTCGTCGTCGGTGAGACGGCACGCGCCGATCATGTCAGCTTCAATGGCTATGAGCGCGATACTTTCCCACAGCTTGC

CAAGATCGATGGCGTGACCAATTTTAGCAATGTCACATCGTGCGGCACATCGACGGCGTATTCTGTGCCGTGTATGTTCA

GCTATCTGGGCGCGGATGAGTATGATGTCGATACCGCCAAATACCAAGAAAATGTGCTGGATACGCTGGATCGCTTGGGC

GTAAGTATCTTGTGGCGTGATAATAATTCGGACTCAAAAGGCGTGATGGATAAGCTGCCAAAAGCGCAATTTGCCGATTA

TAAATCCGCGACCAACAACGCCATCTGCAACACCAATCCTTATAACGAATGCCGCGATGTCGGTATGCTCGTTGGCTTAG

ATGACTTTGTCGCTGCCAATAACGGCAAAGATATGCTGATCATGCTGCACCAAATGGGCAATCACGGGCCTGCGTATTTT

AAGCGATATGATGAAAAGTTTGCCAAATTCACGCCAGCGTGTGAAGGTAATGAGCTTGCCAAGTGCGAACATCAGTCCTT

GATCAATGCTTATGACAATGCCTTGCTTGCCACCGATGATTTCATCGCTCAAAGTATCCAGTGGCTGCAGACGCACAGCA

ATGCCTATGATGTCTCAATGCTGTATGTCAGCGATCATGGCGAAAGTCTGGGTGAGAACGGTGTCTATCTACATGGTATG

CCAAATGCCTTTGCACCAAAAGAACAGCGCAGTGTGCCTGCATTTTTCTGGACGGATAAGCAAACTGGCATCACGCCAAT

GGCAACCGATACCGTCCTGACCCATGACGCGATCACGCCGACATTATTAAAGCTGTTTGATGTCACCGCGGACAAAGTCA

AAGACCGCACCGCATTCATCCGCTGA

>MG517528.1 Escherichia coli strain B11 plasmid pBF123 phosphoethanolamine transferase (mcr-1) gene, partial cds

GCGGATGAATGCGGTGCGGTCTTTGACTTTGTCCGCGGTGACATCAAACAGCTTTAATAATGTCGGCGTGATCGCGTCAT

GGGTCAGGACGGTATCGGTTGCCATTGGCGTGATGCCAGTTTGCTTATCCGTCCAGAAAAATGCAGGCACACTGCGCTGT

TCTTTTGGTGCAAAGGCATTTGGCATACCATGTAGATAGACACCGTTCTCACCCAGACTTTCGCCATGATCGCTGACATA

CAGCATTGAGACATCATAGGCATTGCTGTGCGTCTGCAGCCACTGGATACTTTGAGCGATGAAATCATCGGTGGCAAGCA

AGGCATTGTCATAAGCATTGATCAAGGACTGATGTTCGCACTTGGCAAGCTCATTACCTTCACACACTGGCGTGAATTTG

GCAAACTTTTCATCATATCGCTTAAAATACGCAGGCCCGTGATTGCCCATTTGGTGCAGCATGATCAGCATATCTTTGCC

GTTATTGGCAGCGACAAAGTCATCTAAGCCAACGAGCATACCGACATCGCGGCATTCGTTATAAGGATTGGTGTTGCAGA

TGGCGTTGTTGGTCGCGGATTTATAATCGGCAAATTGCGCTTTTGGCAGCTTATCCATCACGCCTTTTGAGTCCGAATTA

TTATCACGCCACAAGATACTTACGCCCAAGCGATCCAGCGTATCCAGCACATTTTCTTGGTATTTGGCGGTATCGACATC

ATACTCATCCGCGCCCAGATAGCTGAACATACACGGCACAGAATACGCCGTCGATGTGCCGCACGATGTGACATTGCTAA

AATTGGTCACGCCATCGATCTTGGCAAGCTGTGGGAAAGTATCGCGCTCATAGCCATTGAAGCTGACATGATCGGCGCGT

GCCGTCTCACCGACGACGAACACCACTAGGCGTGGCTTACGCATATCAGGCTTGGTTGCTTGTACCGCGTCTTTGGCGTG

ATAAATGGTATCTTTTGGCGCACTGGCTTTTTTATACTCAATACTGGCAAGCTTACCCACCGAGTAGATTGGCATGATCG

GATTGACATAGCTACGCAGCGGCTTATGCACGCGAAAGAAACTGGCATAATGACTGCTGAACGCCACCACAGGCAGTAAA

ATCAGCGCAAGACTTGCCACGATCAAGCCCAATCGGCGCATCAAACCCTTGCCCCAAGTCGGATAATCCACCTTAACAAA

AGCCACAAGCAAACTTGGTAGCACACCCAAACCAATGATACGCATGATAAACGCTGCGTTTAATAGATCCTTGGTCTCGG

CTTGGTCGGTCTGTAGGGCATTTTGGAGCATGGTCGTATCATAGACCGTGCCATAAGTGTCAGTAAAATAACTGGTCACC

GCGCCCATGATTAATAGCAAAATCAACACAGGCTTTAGCACATAGCGATACGATGATAACAGCGTGGTGATCAGTAGCAT

CGCGCCAAAGAGCACGACAGCGATCGTCAGCACAAAGCCGAGATTGTCCGCGATGGGATAGGTTTGGCTGATTTTATCAA

AAAAGGTAAGATTGGCGGTCGCGGTCAAGAAAACGGCAACACTCGCCACAAGAACAAACGGACTGACCGAGCGTCGGTA
C

CACACAGAAGTATGCTGCATCAT

>LC337668.1 Escherichia coli EC15-101 mcr-1 gene for phosphoethanolamine--lipid A transferase MCR-1.12, complete cds

ATGATGCACCATACTTCTGTGTGGTACCGACGCTCGGTCAGTCCGTTTGTTCTTGTGGCGAGTGTTGCCGTTTTCTTGAC

CGCGACCGCCAATCTTACCTTTTTTGATAAAATCAGCCAAACCTATCCCATCGCGGACAATCTCGGCTTTGTGCTGACGA

TCGCTGTCGTGCTCTTTGGCGCGATGCTACTGATCACCACGCTGTTATCATCGTATCGCTATGTGCTAAAGCCTGTGTTG

ATTTTGCTATTAATCATGGGCGCGGTGACCAGTTATTTTACTGACACTTATGGCACGGTCTATGATACGACCATGCTCCA

AAATGCCCTACAGACCGACCAAGCCGAGACCAAGGATCTATTAAACGCAGCGTTTATCATGCGTATCATTGGTTTGGGTG

TGCTACCAAGTTTGCTTGTGGCTTTTGTTAAGGTGGATTATCCGACTTGGGGCAAGGGTTTGATGCGCCGATTGGGCTTG

ATCGTGGCAAGTCTTGCGCTGATTTTACTGCCTGTGGTGGCGTTCAGCAGTCATTATGCCAGTTTCTTTCGCGTGCATAA

GCCGCTGCGTAGCTATGTCAATCCGATCATGCCAATCTACTCGGTGGGTAAGCTTGCCAGTATTGAGTATAAAAAAGCCA

GTGCGCCAAAAGATACCATTTATCACGCCAAAGACGCGGTACAAGCAACCAAGCCTGATATGCGTAAGCCACGCCTAGTG

GTGTTCGTCGTCGGTGAGACGGCACGCGCCGATCATGTCAGCTTCAATGGCTATGAGCGCGATACTTTCCCACAGCTTGC

CAAGATCGATGGCGTGACCAATTTTAGCAATGTCACATCGTGCGGCACATCGACGGCGTATTCTGTGCCGTGTATGTTCA

GCTATCTGGGCGCGGATGAGTATGATGTCGATACCGCCAAATACCAAGAAAATGTGCTGGATACGCTGGATCGCTTGGGC

GTAAGTATCTTGTGGCGTGATAATAATTCGGACTCAAAAGGCGTGATGGATAAGCTGCCAAAAGCGCAATTTGCCGATTA

TAAATCCGCGACCAACAACGCCATCTGCAACACCAATCCTTATAACGAATGCCGCGATGTCGGTATGCTCGTTGGCTTAG

ATGACTTTGTCGCTGCCAATAACGGCAAAGATATGCTGATCATGCTGCACCAAATGGGCAATCACGGGCCTGCGTATTTT

AAGCGATATGATGAAAAGTTTGCCAAATTCACGCCAGTGTGTGAAGGTAATGAGCTTGCCAAGTGCGAACATCAGTCCTT

GATCAATGCTTATGACAATGCCTTGCTTGCCACCGATGATTTCATCGCTCAAAGTATCCAGTGGCTGCAGACGCACAGCA

ATGCCTATGATGTCTCAATGCTGTATGTCAGCGATCATGGCGAAAGTCTGGGTGAGAACGGTGTCTATCTACATGGTATG

CCAAATGCCTTTGCACCAAAAGAACAGCGCAGTGTGCCTGCATTTTTCTGGACGGATAAGCAAACTGGCATCACGCCAAT

GGCAACCGATACCGTCCTGACCCATGACGCGATCACGCCGACATTATTAAAGCTGTTTGATGTCACCGCGGACAAAGTCA

AAGACCGCACCGCATTCATCCGCTGA

>KY271416.1 Escherichia coli strain Ec1670 phosphoethanolamine--lipid A transferase MCR-1.5 (mcr-1) gene, mcr-1.5 allele, complete cds

ATGATGCAGCATACTTCTGTGTGGTACCGACGCTCGGTCAGTCCGTTTGTTCTTGTGGCGAGTGTTGCCGTTTTCTTGAC

CGCGACCGCCAATCTTACCTTTTTTGATAAAATCAGCCAAACCTATCCCATCGCGGACAATCTCGGCTTTGTGCTGACGA

TCGCTGTCGTGCTCTTTGGCGCGATGCTACTGATCACCACGCTGTTATCATCGTATCGCTATGTGCTAAAGCCTGTGTTG

ATTTTGCTATTAATCATGGGCGCGGTGACCAGTTATTTTACTGACACTTATGGCACGGTCTATGATACGACCATGCTCCA

AAATGCCCTACAGACCGACCAAGCCGAGACCAAGGATCTATTAAACGCAGCGTTTATCATGCGTATCATTGGTTTGGGTG

TGCTACCAAGTTTGCTTGTGGCTTTTGTTAAGGTGGATTATCCGACTTGGGGCAAGGGTTTGATGCGCCGATTGGGCTTG

ATCGTGGCAAGTCTTGCGCTGATTTTACTGCCTGTGGTGGCGTTCAGCAGTCATTATGCCAGTTTCTTTCGCGTGCATAA

GCCGCTGCGTAGCTATGTCAATCCGATCATGCCAATCTACTCGGTGGGTAAGCTTGCCAGTATTGAGTATAAAAAAGCCA

GTGCGCCAAAAGATACCATTTATCACGCCAAAGACGCGGTACAAGCAACCAAGCCTGATATGCGTAAGCCACGCCTAGTG

GTGTTCGTCGTCGGTGAGACGGCACGCGCCGATCATGTCAGCTTCAATGGCTATGAGCGCGATACTTTCCCACAGCTTGC

CAAGATCGATGGCGTGACCAATTTTAGCAATGTCACATCGTGCGGCACATCGACGGCGTATTCTGTGCCGTGTATGTTCA

GCTATCTGGGCGCGGATGAGTATGATGTCGATACCGCCAAATACCAAGAAAATGTGCTGGATACGCTGGATCGCTTGGGC

GTAAGTATCTTGTGGCGTGATAATAATTCGGACTCAAAAGGCGTGATGGATAAGCTGCCAAAAGCGCAATTTGCCGATTA

TAAATCCGCGACCAACAACGCCATCTGCAACACCAATCCTTATAACGAATGCCGCGATGTCGGTATGCTCGTTGGCTTAG

ATGACTTTGTCGCTGCCAATAACGGCAAAGATATGCTGATCATGCTGCACCAAATGGGCAATCACGGGCCTGCGTATTTT

AAGCGATATGATGAAAAGTTTGCCAAATTCACGCCAGTGTGTGAAGGTAATGAGCTTGCCAAGTGCGAACATCAGTCCTT

GATCAATGCTTATGACAATGCCTTGCTTGCCACCGATGATTTCATCGCTCAAAGTATCCAGTGGCTGCAGACGTACAGCA

ATGCCTATGATGTCTCAATGCTGTATGTCAGCGATCATGGCGAAAGTCTGGGTGAGAACGGTGTCTATCTACATGGTATG

CCAAATGCCTTTGCACCAAAAGAACAGCGCAGTGTGCCTGCATTTTTCTGGACGGATAAGCAAACTGGCATCACGCCAAT

GGCAACCGATACCGTCCTGACCCATGACGCGATCACGCCGACATTATTAAAGCTGTTTGATGTCACCGCGGACAAAGTCA

AAGACCGCACCGCATTCATCCGCTGA

>NG_055582.1 Escherichia coli LV23529 pLV23529-MCR-1.9 mcr-1 gene for phosphoethanolamine--lipid A transferase MCR-1.9, complete CDS

CGCATAATTTTTTATATCAGATAAATTGTACTGGATTTCTTAAAAAATTGCAGTATAATTGCCGCAATTATCCCACCGTT

TATTTTTTGAGTAGTTTCTCATGATGCAGCATACTTCTGTGTGGTACCGACGCTCGGTCAGTCCGTTTGTTCTTGTGGCG

AGTGTTGCCGTTTTCTTGACCGCGACCGCCAATCTTACCTTTTTTGATAAAATCAGCCAAACCTATCCCATCGCGGACAA

TCTCGGCTTTGTGCTGACGATCGCTGTCGTGCTCTTTGGCGCGATGCTACTGATCACCACGCTGTTATCATCGTATCGCT

ATGTGCTAAAGCCTGTGTTGATTTTGCTATTAATCATGGGCGCGGTGACCAGTTATTTTACTGACACTTATGGCACGGTC

TATGATACGACCATGCTCCAAAATGCCCTACAGACCGACCAAGCCGAGACCAAGGATCTATTAAACGCAGCGTTTATCAT

GCGTATCATTGGTTTGGGTGTGCTACCAAGTTTGCTTGTGGCTTTTGTTAAGGTGGATTATCCGACTTGGGGCAAGGGTT

TGATGCGCCGATTGGGCTTGATCGTGGCAAGTCTTGCGCTGATTTTACTGCCTGTGGTGGCGTTCAGCAGTCATTATGCC

AGTTTCTTTCGCGTGCATAAGCCGCTGCGTAGCTATGTCAATCCGATCATGCCAATCTACTCGGTGGGTAAGCTTGCCAG

TATTGAGTATAAAAAAGCCAGTGCGCCAAAAGATACCATTTATCACGCCAAAGACGCGGTACAAGCAACCAAGCCTGATA

TGCGTAAGCCACGCCTAGTGGTGTTCGTCGTCGGTGAGACGGCACGCGCCGATCATGTCAGCTTCAATGGCTATGAGCGC

GATACTTTCCCACAGCTTGCCAAGATCGATGGCGTGACCAATTTTAGCAATGTCACATCGTGCGGCACATCGACGGCGTA

TTCTGTGCCGTGTATGTTCAGCTATCTGGGCGCGGATGAGTATGATGTCGATACCGCCAAATACCAAGAAAATGTGCTGG

ATACGCTGGATCGCTTGGGCGTAAGTATCTTGTGGCGTGATAATAATTCGGACTCAAAAGGCGTGATGGATAAGCTGCCA

AAAGCGCAATTTGCCGATTATAAATCCGCGACCAACAACGCCATCTGCAACACCAATCCTTATAACGAATGCCGCGATGT

CGGTATGCTCGTTGGCTTAGATGACTTTGTCGCTGCCAATAACGGCAAAGATATGCTGATCATGCTGCACCAAATGGGCA

ATCACGGGCCTGCGTATTTTAAGCGATATGATGAAAAGTTTGCCAAATTCACGCCAGCGTGTGAAGGTAATGAGCTTGCC

AAGTGCGAACATCAGTCCTTGATCAATGCTTATGACAATGCCTTGCTTGCCACCGATGATTTCATCGCTCAAAGTATCCA

GTGGCTGCAGACGCACAGCAATGCCTATGATGTCTCAATGCTGTATGTCAGCGATCATGGCGAAAGTCTGGGTGAGAACG

GTGTCTATCTACATGGTATGCCAAATGCCTTTGCACCAAAAGAACAGCGCAGTGTGCCTGCATTTTTCTGGACGGATAAG

CAAACTGGCATCACGCCAATGGCAACCGATACCGTCCTGACCCATGACGCGATCACGCCGACATTATTAAAGCTGTTTGA

TGTCACCGCGGACAAAGTCAAAGACCGCACCGCATTCATCCGCTGATTTCTCCCTGTATTTTTCCAAACCCACCGCACA

CTCCATTCGTATTATGGGCGGTGGGGTGGGGTTTGTTATGCCGTATTTATCAAATAAACGCCTACT

>KY400027.1 Escherichia coli strain M4 Mcr-1.3 (mcr-1.3) gene, complete cds

ATGATGCAGCATACTTCTGTGTGGTACCGACGCTCGGTCAGTCCGTTGGTTCTTGTGGCGAGTGTTGCCGTTTTCTTGAC

CGCGACCGCCAATCTTACCTTTTTTGATAAAATCAGCCAAACCTATCCCATCGCGGACAATCTCGGCTTTGTGCTGACGA

TCGCTGTCGTGCTCTTTGGCGCGATGCTACTGATCACCACGCTGTTATCATCGTATCGCTATGTGCTAAAGCCTGTGTTG

ATTTTGCTATTAATCATGGGCGCGGTGACCAGTTATTTTACTGACACTTATGGCACGGTCTATGATACGACCATGCTCCA

AAATGCCCTACAGACCGACCAAGCCGAGACCAAGGATCTATTAAACGCAGCGTTTATCATGCGTATCATTGGTTTGGGTG

TGCTACCAAGTTTGCTTGTGGCTTTTGTTAAGGTGGATTATCCGACTTGGGGCAAGGGTTTGATGCGCCGATTGGGCTTG

ATCGTGGCAAGTCTTGCGCTGATTTTACTGCCTGTGGTGGCGTTCAGCAGTCATTATGCCAGTTTCTTTCGCGTGCATAA

GCCGCTGCGTAGCTATGTCAATCCGATCATGCCAATCTACTCGGTGGGTAAGCTTGCCAGTATTGAGTATAAAAAAGCCA

GTGCGCCAAAAGATACCATTTATCACGCCAAAGACGCGGTACAAGCAACCAAGCCTGATATGCGTAAGCCACGCCTAGTG

GTGTTCGTCGTCGGTGAGACGGCACGCGCCGATCATGTCAGCTTCAATGGCTATGAGCGCGATACTTTCCCACAGCTTGC

CAAGATCGATGGCGTGACCAATTTTAGCAATGTCACATCGTGCGGCACATCGACGGCGTATTCTGTGCCGTGTATGTTCA

GCTATCTGGGCGCGGATGAGTATGATGTCGATACCGCCAAATACCAAGAAAATGTGCTGGATACGCTGGATCGCTTGGGC

GTAAGTATCTTGTGGCGTGATAATAATTCGGACTCAAAAGGCGTGATGGATAAGCTGCCAAAAGCGCAATTTGCCGATTA

TAAATCCGCGACCAACAACGCCATCTGCAACACCAATCCTTATAACGAATGCCGCGATGTCGGTATGCTCGTTGGCTTAG

ATGACTTTGTCGCTGCCAATAACGGCAAAGATATGCTGATCATGCTGCACCAAATGGGCAATCACGGGCCTGCGTATTTT

AAGCGATATGATGAAAAGTTTGCCAAATTCACGCCAGTGTGTGAAGGTAATGAGCTTGCCAAGTGCGAACATCAGTCCTT

GATCAATGCTTATGACAATGCCTTGCTTGCCACCGATGATTTCATCGCTCAAAGTATCCAGTGGCTGCAGACGCACAGCA

ATGCCTATGATGTCTCAATGCTGTATGTCAGCGATCATGGCGAAAGTCTGGGTGAGAACGGTGTCTATCTACATGGTATG

CCAAATGCCTTTGCACCAAAAGAACAGCGCAGTGTGCCTGCATTTTTCTGGACGGATAAGCAAACTGGCATCACGCCAAT

GGCAACCGATACCGTCCTGACCCATGACGCGATCACGCCGACATTATTAAAGCTGTTTGATGTCACCGCGGACAAAGTCA

AAGACCGCACCGCATTCATCCGCTGA

>NG_054697.1 Escherichia coli ST101 mcr-1 gene for phosphoethanolamine--lipid A transferase MCR-1.8, complete CDS

ATGATGCGGCATACTTCTGTGTGGTACCGACGCTCGGTCAGTCCGTTTGTTCTTGTGGCGAGTGTTGCCGTTTTCTTGAC

CGCGACCGCCAATCTTACCTTTTTTGATAAAATCAGCCAAACCTATCCCATCGCGGACAATCTCGGCTTTGTGCTGACGA

TCGCTGTCGTGCTCTTTGGCGCGATGCTACTGATCACCACGCTGTTATCATCGTATCGCTATGTGCTAAAGCCTGTGTTG

ATTTTGCTATTAATCATGGGCGCGGTGACCAGTTATTTTACTGACACTTATGGCACGGTCTATGATACGACCATGCTCCA

AAATGCCCTACAGACCGACCAAGCCGAGACCAAGGATCTATTAAACGCAGCGTTTATCATGCGTATCATTGGTTTGGGTG

TGCTACCAAGTTTGCTTGTGGCTTTTGTTAAGGTGGATTATCCGACTTGGGGCAAGGGTTTGATGCGCCGATTGGGCTTG

ATCGTGGCAAGTCTTGCGCTGATTTTACTGCCTGTGGTGGCGTTCAGCAGTCATTATGCCAGTTTCTTTCGCGTGCATAA

GCCGCTGCGTAGCTATGTCAATCCGATCATGCCAATCTACTCGGTGGGTAAGCTTGCCAGTATTGAGTATAAAAAAGCCA

GTGCGCCAAAAGATACCATTTATCACGCCAAAGACGCGGTACAAGCAACCAAGCCTGATATGCGTAAGCCACGCCTAGTG

GTGTTCGTCGTCGGTGAGACGGCACGCGCCGATCATGTCAGCTTCAATGGCTATGAGCGCGATACTTTCCCACAGCTTGC

CAAGATCGATGGCGTGACCAATTTTAGCAATGTCACATCGTGCGGCACATCGACGGCGTATTCTGTGCCGTGTATGTTCA

GCTATCTGGGCGCGGATGAGTATGATGTCGATACCGCCAAATACCAAGAAAATGTGCTGGATACGCTGGATCGCTTGGGC

GTAAGTATCTTGTGGCGTGATAATAATTCGGACTCAAAAGGCGTGATGGATAAGCTGCCAAAAGCGCAATTTGCCGATTA

TAAATCCGCGACCAACAACGCCATCTGCAACACCAATCCTTATAACGAATGCCGCGATGTCGGTATGCTCGTTGGCTTAG

ATGACTTTGTCGCTGCCAATAACGGCAAAGATATGCTGATCATGCTGCACCAAATGGGCAATCACGGGCCTGCGTATTTT

AAGCGATATGATGAAAAGTTTGCCAAATTCACGCCAGTGTGTGAAGGTAATGAGCTTGCCAAGTGCGAACATCAGTCCTT

GATCAATGCTTATGACAATGCCTTGCTTGCCACCGATGATTTCATCGCTCAAAGTATCCAGTGGCTGCAGACGCACAGCA

ATGCCTATGATGTCTCAATGCTGTATGTCAGCGATCATGGCGAAAGTCTGGGTGAGAACGGTGTCTATCTACATGGTATG

CCAAATGCCTTTGCACCAAAAGAACAGCGCAGTGTGCCTGCATTTTTCTGGACGGATAAGCAAACTGGCATCACGCCAAT

GGCAACCGATACCGTCCTGACCCATGACGCGATCACGCCGACATTATTAAAGCTGTTTGATGTCACCGCGGACAAAGTCA

AAGACCGCACCGCATTCATCCGCTGA

>NG_054678.1 Escherichia coli WCHEC1604 mcr-1 gene for phosphoethanolamine--lipid A transferase MCR-1.7, complete CDS

ATGATGCAGCATACTTCTGTGTGGTACCGACGCTCGGTCAGTCCGTTTGTTCTTGTGGCGAGTGTTGCCGTTTTCTTGAC

CGCGACCGCCAATCTTACCTTTTTTGATAAAATCAGCCAAACCTATCCCATCGCGGACAATCTCGGCTTTGTGCTGACGA

TCGCTGTCGTGCTCTTTGGCGCGATGCTACTGATCACCACGCTGTTATCATCGTATCGCTATGTGCTAAAGCCTGTGTTG

ATTTTGCTATTAATCATGGGCGCGGTGACCAGTTATTTTACTGACACTTATGGCACGGTCTATGATACGACCATGCTCCA

AAATGCCCTACAGACCGACCAAGCCGAGACCAAGGATCTATTAAACGCAGCGTTTATCATGCGTATCATTGGTTTGGGTG

TGCTACCAAGTTTGCTTGTGGCTTTTGTTAAGGTGGATTATCCGACTTGGGGCAAGGGTTTGATGCGCCGATTGGGCTTG

ATCGTGGCAAGTCTTGCGCTGATTTTACTGCCTGTGGTGGCGTTCAGCAGTCATTATGCCAGTTTCTTTCGCGTGCATAA

GCCGCTGCGTAGCTATGTCAATCCGATCATGCCAATCTACTCGGTGGGTAAGCTTGCCAGTATTGAGTATAAAAAAGCCA

GTACGCCAAAAGATACCATTTATCACGCCAAAGACGCGGTACAAGCAACCAAGCCTGATATGCGTAAGCCACGCCTAGTG

GTGTTCGTCGTCGGTGAGACGGCACGCGCCGATCATGTCAGCTTCAATGGCTATGAGCGCGATACTTTCCCACAGCTTGC

CAAGATCGATGGCGTGACCAATTTTAGCAATGTCACATCGTGCGGCACATCGACGGCGTATTCTGTGCCGTGTATGTTCA

GCTATCTGGGCGCGGATGAGTATGATGTCGATACCGCCAAATACCAAGAAAATGTGCTGGATACGCTGGATCGCTTGGGC

GTAAGTATCTTGTGGCGTGATAATAATTCGGACTCAAAAGGCGTGATGGATAAGCTGCCAAAAGCGCAATTTGCCGATTA

TAAATCCGCGACCAACAACGCCATCTGCAACACCAATCCTTATAACGAATGCCGCGATGTCGGTATGCTCGTTGGCTTAG

ATGACTTTGTCGCTGCCAATAACGGCAAAGATATGCTGATCATGCTGCACCAAATGGGCAATCACGGGCCTGCGTATTTT

AAGCGATATGATGAAAAGTTTGCCAAATTCACGCCAGTGTGTGAAGGTAATGAGCTTGCCAAGTGCGAACATCAGTCCTT

GATCAATGCTTATGACAATGCCTTGCTTGCCACCGATGATTTCATCGCTCAAAGTATCCAGTGGCTGCAGACGCACAGCA

ATGCCTATGATGTCTCAATGCTGTATGTCAGCGATCATGGCGAAAGTCTGGGTGAGAACGGTGTCTATCTACATGGTATG

CCAAATGCCTTTGCACCAAAAGAACAGCGCAGTGTGCCTGCATTTTTCTGGACGGATAAGCAAACTGGCATCACGCCAAT

GGCAACCGATACCGTCCTGACCCATGACGCGATCACGCCGACATTATTAAAGCTGTTTGATGTCACCGCGGACAAAGTCA

AAGACCGCACCGCATTCATCCGCTGA

>KY685071.1 Escherichia coli strain HKSH_MCR_161114268_EC phosphoethanolamine lipid A transferase (mcr1) gene, mcr1.9 allele, complete cds

ATGATGCAGCATACTTCTGTGTGGTATCGACGCTCGGTCAGTCCGTTTGTTCTTGTGGCGAGTGTTGCCGTTTTCTTGAC

CGCGACCGCCAATCTTACCTTTTTTGATAAAATCAGCCAAACCTATCCCATCGCGGACAATCTCGGCTTTGTGCTGACGA

TCGCTGTCGTGCTCTTTGGCGCGATGCTACTGATCACCACGCTGTTATCATCGTATCGCTATGTGCTAAAGCCTGTGTTG

ATTTTGCTATTAATCATGGGCGCGGTGACCAGTTATTTTACTGACACTTATGGCACGGTCTATGATACGACCATGCTCCA

AAATGCCCTACAGACCGACCAAGCCGAGACCAAGGATCTATTAAACGCAGCGTTTATCATGCGTATCATTGGTTTGGGTG

TGCTACCAAGTTTGCTTGTGGCTTTTGTTAAGGTGGATTATCCGACTTGGGGCAAGGGTTTGATGCGCCGATTGGGCTTG

ATCGTGGCAAGTCTTGCGCTGATTTTACTGCCTGTGGTGGCGTTCAGCAGTCATTATGCCAGTTTCTTTCGCGTGCATAA

GCCGCTGCGTAGCTATGTCAATCCGATCATGCCAATCTACTCGGTGGGTAAGCTTGCCAGTATTGAGTATAAAAAAGCCA

GTGCGCCAAAAGATACCATTTATCACGCCAAAGACGCGGTACAAGCAACCAAGCCTGATATGCGTAAGCCACGCCTAGTG

GTGTTCGTCGTCGGTGAGACGGCACGCGCCGATCATGTCAGCTTCAATGGCTATGAGCGCGATACTTTCCCACAGCTTGC

CAAGATCGATGGCGTGACCAATTTTAGCAATGTCACATCGTGCGGCACATCGACGGCGTATTCTGTGCCGTGTATGTTCA

GCTATCTGGGCGCGGATGAGTATGATGTCGATACCGCCAAATACCAAGAAAATGTGCTGGATACGCTGGATCGCTTGGGC

GTAAGTATCTTGTGGCGTGATAATAATTCGGACTCAAAAGGCGTGATGGATAAGCTGCCAAAAGCGCAATTTGCCGATTA

TAAATCCGCGACCAACAACGCCATCTGCAACACCAATCCTTATAACGAATGCCGCGATGTCGGTATGCTCGTTGGCTTAG

ATGACTTTGTCGCTGCCAATAACGGCAAAGATATGCTGATCATGCTGCACCAAATGGGCAATCACGGGCCTGCGTATTTT

AAGCGATATGATGAAAAGTTTGCCAAATTCACGCCAGTGTGTGAAGGTAATGAGCTTGCCAAGTGCGAACATCAGTCCTT

GATCAATGCTTATGACAATGCCTTGCTTGCCACCGATGATTTCATCGCTCAAAGTATCCAGTGGCTGCAGACGCACAGCA

ATGCCTATGATGTCTCAATGCTGTATGTCAGCGATCATGGCGAAAGTCTGGGTGAGAACGGTGTCTATCTACATGGTATG

CCAAATGCCTTTGCACCAAAAGAACAGCGCAGTGTGCCTGCATTTTTCTGGACGGATAAGCAAACTGGCATCACGCCAAT

GGCAACCGATACCGTCCTGACCCATGACGCGATCACGCCGACATTATTAAAGCTGTTTGATGTCACCGCGGACAAAGTCA

AAGACCGCACCGCATTCATCCGCTGA

>KY683842.1 Escherichia coli strain ST101 phosphoethanolamine transferase (mcr-1.8) gene, complete cds

ATGATGCGGCATACTTCTGTGTGGTACCGACGCTCGGTCAGTCCGTTTGTTCTTGTGGCGAGTGTTGCCGTTTTCTTGAC

CGCGACCGCCAATCTTACCTTTTTTGATAAAATCAGCCAAACCTATCCCATCGCGGACAATCTCGGCTTTGTGCTGACGA

TCGCTGTCGTGCTCTTTGGCGCGATGCTACTGATCACCACGCTGTTATCATCGTATCGCTATGTGCTAAAGCCTGTGTTG

ATTTTGCTATTAATCATGGGCGCGGTGACCAGTTATTTTACTGACACTTATGGCACGGTCTATGATACGACCATGCTCCA

AAATGCCCTACAGACCGACCAAGCCGAGACCAAGGATCTATTAAACGCAGCGTTTATCATGCGTATCATTGGTTTGGGTG

TGCTACCAAGTTTGCTTGTGGCTTTTGTTAAGGTGGATTATCCGACTTGGGGCAAGGGTTTGATGCGCCGATTGGGCTTG

ATCGTGGCAAGTCTTGCGCTGATTTTACTGCCTGTGGTGGCGTTCAGCAGTCATTATGCCAGTTTCTTTCGCGTGCATAA

GCCGCTGCGTAGCTATGTCAATCCGATCATGCCAATCTACTCGGTGGGTAAGCTTGCCAGTATTGAGTATAAAAAAGCCA

GTGCGCCAAAAGATACCATTTATCACGCCAAAGACGCGGTACAAGCAACCAAGCCTGATATGCGTAAGCCACGCCTAGTG

GTGTTCGTCGTCGGTGAGACGGCACGCGCCGATCATGTCAGCTTCAATGGCTATGAGCGCGATACTTTCCCACAGCTTGC

CAAGATCGATGGCGTGACCAATTTTAGCAATGTCACATCGTGCGGCACATCGACGGCGTATTCTGTGCCGTGTATGTTCA

GCTATCTGGGCGCGGATGAGTATGATGTCGATACCGCCAAATACCAAGAAAATGTGCTGGATACGCTGGATCGCTTGGGC

GTAAGTATCTTGTGGCGTGATAATAATTCGGACTCAAAAGGCGTGATGGATAAGCTGCCAAAAGCGCAATTTGCCGATTA

TAAATCCGCGACCAACAACGCCATCTGCAACACCAATCCTTATAACGAATGCCGCGATGTCGGTATGCTCGTTGGCTTAG

ATGACTTTGTCGCTGCCAATAACGGCAAAGATATGCTGATCATGCTGCACCAAATGGGCAATCACGGGCCTGCGTATTTT

AAGCGATATGATGAAAAGTTTGCCAAATTCACGCCAGTGTGTGAAGGTAATGAGCTTGCCAAGTGCGAACATCAGTCCTT

GATCAATGCTTATGACAATGCCTTGCTTGCCACCGATGATTTCATCGCTCAAAGTATCCAGTGGCTGCAGACGCACAGCA

ATGCCTATGATGTCTCAATGCTGTATGTCAGCGATCATGGCGAAAGTCTGGGTGAGAACGGTGTCTATCTACATGGTATG

CCAAATGCCTTTGCACCAAAAGAACAGCGCAGTGTGCCTGCATTTTTCTGGACGGATAAGCAAACTGGCATCACGCCAAT

GGCAACCGATACCGTCCTGACCCATGACGCGATCACGCCGACATTATTAAAGCTGTTTGATGTCACCGCGGACAAAGTCA

AAGACCGCACCGCATTCATCCGCTGA

>NG_052664.1 Escherichia coli WCHEC1606 mcr-1 gene for phosphoethanolamine--lipid A transferase MCR-1.4, complete CDS

ATGATGCAGCATACTTCTGTGTGGTACCGACGCTCGGTCAGTCCGTTTGTTCTTGTGGCGAGTGTTGCCGTTTTCTTGAC

CGCGACCGCCAATCTTACCTTTTTTGATAAAATCAGCCAAACCTATCCCATCGCGGACAATCTCGGCTTTGTGCTGACGA

TCGCTGTCGTGCTCTTTGGCGCGATGCTACTGATCACCACGCTGTTATCATCGTATCGCTATGTGCTAAAGCCTGTGTTG

ATTTTGCTATTAATCATGGGCGCGGTGACCAGTTATTTTACTGACACTTATGGCACGGTCTATGATACGACCATGCTCCA

AAATGCCCTACAGACCGACCAAGCCGAGACCAAGGATCTATTAAACGCAGCGTTTATCATGCGTATCATTGGTTTGGGTG

TGCTACCAAGTTTGCTTGTGGCTTTTGTTAAGGTGGATTATCCGACTTGGGGCAAGGGTTTGATGCGCCGATTGGGCTTG

ATCGTGGCAAGTCTTGCGCTGATTTTACTGCCTGTGGTGGCGTTCAGCAGTCATTATGCCAGTTTCTTTCGCGTGCATAA

GCCGCTGCGTAGCTATGTCAATCCGATCATGCCAATCTACTCGGTGGGTAAGCTTGCCAGTATTGAGTATAAAAAAGCCA

GTGCGCCAAAAGATACCATTTATCACGCCAAAGACGCGGTACAAGCAACCAAGCCTGATATGCGTAAGCCACGCCTAGTG

GTGTTCGTCGTCGGTGAGACGGCACGCGCCGATCATGTCAGCTTCAATGGCTATGAGCGCGATACTTTCCCACAGCTTGC

CAAGATCGATGGCGTGACCAATTTTAGCAATGTCACATCGTGCGGCACATCGACGGCGTATTCTGTGCCGTGTATGTTCA

GCTATCTGGGCGCGGATGAGTATGATGTCGATACCGCCAAATACCAAGAAAATGTGCTGGATACGCTGGATCGCTTGGGC

GTAAGTATCTTGTGGCGTGATAATAATTCGGACTCAAAAGGCGTGATGGATAAGCTGCCAAAAGCGCAATTTGCCGATTA

TAAATCCGCGACCAACAACGCCATCTGCAACACCAATCCTTATAACGAATGCCGCGATGTCGGTATGCTCGTTGGCTTAG

ATGACTTTGTCGCTGCCAATAACGGCAAAGATATGCTGATCATGCTGCACCAAATGGGCAATCACGGGCCTGCGTATTTT
AAGCGATATGATGAAAAGTTTGCCAAATTCACGCCAGTGTGTGAAGGTAATGAGCTTGCCAAGTGCGAACATCAGTCCTT
GATCAATGCTTATGACAATGCCTTGCTTGCCACCGATAATTTCATCGCTCAAAGTATCCAGTGGCTGCAGACGCACAGCA
ATGCCTATGATGTCTCAATGCTGTATGTCAGCGATCATGGCGAAAGTCTGGGTGAGAACGGTGTCTATCTACATGGTATG
CCAAATGCCTTTGCACCAAAAGAACAGCGCAGTGTGCCTGCATTTTTCTGGACGGATAAGCAAACTGGCATCACGCCAAT
GGCAACCGATACCGTCCTGACCCATGACGCGATCACGCCGACATTATTAAAGCTGTTTGATGTCACCGCGGACAAAGTCA
AAGACCGCACCGCATTCATCCGCTGA

>NG_052663.1 Escherichia coli 1256822 mcr-1 gene for phosphoethanolamine--lipid A transferase MCR-1.5, complete CDS

ATGATGCAGCATACTTCTGTGTGGTACCGACGCTCGGTCAGTCCGTTTGTTCTTGTGGCGAGTGTTGCCGTTTTCTTGAC
CGCGACCGCCAATCTTACCTTTTTTGATAAAATCAGCCAAACCTATCCCATCGCGGACAATCTCGGCTTTGTGCTGACGA
TCGCTGTCGTGCTCTTTGGCGCGATGCTACTGATCACCACGCTGTTATCATCGTATCGCTATGTGCTAAAGCCTGTGTTG
ATTTTGCTATTAATCATGGGCGCGGTGACCAGTTATTTTACTGACACTTATGGCACGGTCTATGATACGACCATGCTCCA
AAATGCCCTACAGACCGACCAAGCCGAGACCAAGGATCTATTAAACGCAGCGTTTATCATGCGTATCATTGGTTTGGGTG
TGCTACCAAGTTTGCTTGTGGCTTTTGTTAAGGTGGATTATCCGACTTGGGGCAAGGGTTTGATGCGCCGATTGGGCTTG
ATCGTGGCAAGTCTTGCGCTGATTTTACTGCCTGTGGTGGCGTTCAGCAGTCATTATGCCAGTTTCTTTCGCGTGCATAA
GCCGCTGCGTAGCTATGTCAATCCGATCATGCCAATCTACTCGGTGGGTAAGCTTGCCAGTATTGAGTATAAAAAAGCCA
GTGCGCCAAAAGATACCATTTATCACGCCAAAGACGCGGTACAAGCAACCAAGCCTGATATGCGTAAGCCACGCCTAGTG
GTGTTCGTCGTCGGTGAGACGGCACGCGCCGATCATGTCAGCTTCAATGGCTATGAGCGCGATACTTTCCCACAGCTTGC
CAAGATCGATGGCGTGACCAATTTTAGCAATGTCACATCGTGCGGCACATCGACGGCGTATTCTGTGCCGTGTATGTTCA

GCTATCTGGGCGCGGATGAGTATGATGTCGATACCGCCAAATACCAAGAAAATGTGCTGGATACGCTGGATCGCTTGGGC

GTAAGTATCTTGTGGCGTGATAATAATTCGGACTCAAAAGGCGTGATGGATAAGCTGCCAAAAGCGCAATTTGCCGATTA

TAAATCCGCGACCAACAACGCCATCTGCAACACCAATCCTTATAACGAATGCCGCGATGTCGGTATGCTCGTTGGCTTAG

ATGACTTTGTCGCTGCCAATAACGGCAAAGATATGCTGATCATGCTGCACCAAATGGGCAATCACGGGCCTGCGTATTTT

AAGCGATATGATGAAAAGTTTGCCAAATTCACGCCAGTGTGTGAAGGTAATGAGCTTGCCAAGTGCGAACATCAGTCCTT

GATCAATGCTTATGACAATGCCTTGCTTGCCACCGATGATTTCATCGCTCAAAGTATCCAGTGGCTGCAGACGTACAGCA

ATGCCTATGATGTCTCAATGCTGTATGTCAGCGATCATGGCGAAAGTCTGGGTGAGAACGGTGTCTATCTACATGGTATG

CCAAATGCCTTTGCACCAAAAGAACAGCGCAGTGTGCCTGCATTTTTCTGGACGGATAAGCAAACTGGCATCACGCCAAT

GGCAACCGATACCGTCCTGACCCATGACGCGATCACGCCGACATTATTAAAGCTGTTTGATGTCACCGCGGACAAAGTCA

AAGACCGCACCGCATTCATCCGCTGA

>KY488488.1 Escherichia coli strain WCHEC1604 phosphoethanolamine--lipid A transferase MCR-1.7 (mcr-1) gene, mcr-1.7 allele, complete cds

ATGATGCAGCATACTTCTGTGTGGTACCGACGCTCGGTCAGTCCGTTTGTTCTTGTGGCGAGTGTTGCCGTTTTCTTGAC

CGCGACCGCCAATCTTACCTTTTTTGATAAAATCAGCCAAACCTATCCCATCGCGGACAATCTCGGCTTTGTGCTGACGA

TCGCTGTCGTGCTCTTTGGCGCGATGCTACTGATCACCACGCTGTTATCATCGTATCGCTATGTGCTAAAGCCTGTGTTG

ATTTTGCTATTAATCATGGGCGCGGTGACCAGTTATTTTACTGACACTTATGGCACGGTCTATGATACGACCATGCTCCA

AAATGCCCTACAGACCGACCAAGCCGAGACCAAGGATCTATTAAACGCAGCGTTTATCATGCGTATCATTGGTTTGGGTG

TGCTACCAAGTTTGCTTGTGGCTTTTGTTAAGGTGGATTATCCGACTTGGGGCAAGGGTTTGATGCGCCGATTGGGCTTG

ATCGTGGCAAGTCTTGCGCTGATTTTACTGCCTGTGGTGGCGTTCAGCAGTCATTATGCCAGTTTCTTTCGCGTGCATAA

GCCGCTGCGTAGCTATGTCAATCCGATCATGCCAATCTACTCGGTGGGTAAGCTTGCCAGTATTGAGTATAAAAAAGCCA

GTACGCCAAAAGATACCATTTATCACGCCAAAGACGCGGTACAAGCAACCAAGCCTGATATGCGTAAGCCACGCCTAGTG

GTGTTCGTCGTCGGTGAGACGGCACGCGCCGATCATGTCAGCTTCAATGGCTATGAGCGCGATACTTTCCCACAGCTTGC

CAAGATCGATGGCGTGACCAATTTTAGCAATGTCACATCGTGCGGCACATCGACGGCGTATTCTGTGCCGTGTATGTTCA

GCTATCTGGGCGCGGATGAGTATGATGTCGATACCGCCAAATACCAAGAAAATGTGCTGGATACGCTGGATCGCTTGGGC

GTAAGTATCTTGTGGCGTGATAATAATTCGGACTCAAAAGGCGTGATGGATAAGCTGCCAAAAGCGCAATTTGCCGATTA

TAAATCCGCGACCAACAACGCCATCTGCAACACCAATCCTTATAACGAATGCCGCGATGTCGGTATGCTCGTTGGCTTAG

ATGACTTTGTCGCTGCCAATAACGGCAAAGATATGCTGATCATGCTGCACCAAATGGGCAATCACGGGCCTGCGTATTTT

AAGCGATATGATGAAAAGTTTGCCAAATTCACGCCAGTGTGTGAAGGTAATGAGCTTGCCAAGTGCGAACATCAGTCCTT

GATCAATGCTTATGACAATGCCTTGCTTGCCACCGATGATTTCATCGCTCAAAGTATCCAGTGGCTGCAGACGCACAGCA

ATGCCTATGATGTCTCAATGCTGTATGTCAGCGATCATGGCGAAAGTCTGGGTGAGAACGGTGTCTATCTACATGGTATG

CCAAATGCCTTTGCACCAAAAGAACAGCGCAGTGTGCCTGCATTTTTCTGGACGGATAAGCAAACTGGCATCACGCCAAT

GGCAACCGATACCGTCCTGACCCATGACGCGATCACGCCGACATTATTAAAGCTGTTTGATGTCACCGCGGACAAAGTCA

AAGACCGCACCGCATTCATCCGCTGA

>KX242348.1 Escherichia coli strain A434-59 plasmid phosphoethanolamine-lipid A transferase MCR-1 (mcr-1) gene, complete cds

TGTATGGGATTGCGCAATGATTGCCTAATAAAATTTTTGAAATATTTCTGTATCGCATAATTTTTTATATCAGATAAATT

GTACTGGATTTCTTAAAAAATTGCAGTATAATTGCCGCAATTATCCCACCGTTTATTTTTTGAGTAGTTTCTCATGATGC

AGCATACTTCTGTGTGGTATCGACGCTCGGTCAGTCCGTTTGTTCTTGTGGCGAGTGTTGCCGTTTTCTTGACCGCGACC

GCCAATCTTACCTTTTTTGATAAAATCAGCCAAACCTATCCCATCGCGGACAATCTCGGCTTTGTGCTGACGATCGCTGT

CGTGCTCTTTGGCGCGATGCTACTGATCACCACGCTGTTATCATCGTATCGCTATGTGCTAAAGCCTGTGTTGATTTTGC

TATTAATCATGGGCGCGGTGACCAGTTATTTTACTGACACTTATGGCACGGTCTATGATACGACCATGCTCCAAAATGCC

CTACAGACCGACCAAGCCGAGACCAAGGATCTATTAAACGCAGCGTTTATCATGCGTATCATTGGTTTGGGTGTGCTACC

AAGTTTGCTTGTGGCTTTTGTTAAGGTGGATTATCCGACTTGGGGCAAGGGTTTGATGCGCCGATTGGGCTTGATCGTGG

CAAGTCTTGCGCTGATTTTACTGCCTGTGGTGGCGTTCAGCAGTCATTATGCCAGTTTCTTTCGCGTGCATAAGCCGCTG

CGTAGCTATGTCAATCCGATCATGCCAATCTACTCGGTGGGTAAGCTTGCCAGTATTGAGTATAAAAAAGCCAGTGCGCC

AAAAGATACCATTTATCACGCCAAAGACGCGGTACAAGCAACCAAGCCTGATATGCGTAAGCCACGCCTAGTGGTGTTCG

TCGTCGGTGAGACGGCACGCGCCGATCATGTCAGCTTCAATGGCTATGAGCGCGATACTTTCCCACAGCTTGCCAAGATC

GATGGCGTGACCAATTTTAGCAATGTCACATCGTGCGGCACATCGACGGCGTATTCTGTGCCGTGTATGTTCAGCTATCT

GGGCGCGGATGAGTATGATGTCGATACCGCCAAATACCAAGAAAATGTGCTGGATACGCTGGATCGCTTGGGCGTAAGTA

TCTTGTGGCGTGATAATAATTCGGACTCAAAAGGCGTGATGGATAAGCTGCCAAAAGCGCAATTTGCCGATTATAAATCC

GCGACCAACAACGCCATCTGCAACACCAATCCTTATAACGAATGCCGCGATGTCGGTATGCTCGTTGGCTTAGATGACTT

TGTCGCTGCCAATAACGGCAAAGATATGCTGATCATGCTGCACCAAATGGGCAATCACGGGCCTGCGTATTTTAAGCGAT

ATGATGAAAAGTTTGCCAAATTCACGCCAGTGTGTGAAGGTAATGAGCTTGCCAAGTGCGAACATCAGTCCTTGATCAAT

GCTTATGACAATGCCTTGCTTGCCACCGATGATTTCATCGCTCAAAGTATCCAGTGGCTGCAGACGCACAGCAATGCCTA

TGATGTCTCAATGCTGTATGTCAGCGATCATGGCGAAAGTCTGGGTGAGAACGGTGTCTATCTACATGGTATGCCAAATG

CCTTTGCACCAAAAGAACAGCGCAGTGTGCCTGCATTTTTCTGGACGGATAAGCAAACTGGCATCACGCCAATGGCAACC

GATACCGTCCTGACCCATGACGCGATCACGCCGACATTATTAAAGCTGTTTGATGTCACCGCGGACAAAGTCAAAGACCG

CACCGCATTCATCCGCTGATTTCTCCCTGTATTTTTTCCAAACCCACCGCACACTCCATTCGTATTATGGGCGGTGGGGT

GGGGTTTGTTATGCCGTATTTATCAAATAAACGCCTAC

>KY041856.1 Escherichia coli strain WCHEC1606 phosphoethanolamine--lipid A transferase MCR-1.4 (mcr-1) gene, mcr-1.4 allele, complete cds

ATGATGCAGCATACTTCTGTGTGGTACCGACGCTCGGTCAGTCCGTTTGTTCTTGTGGCGAGTGTTGCCGTTTTCTTGAC

CGCGACCGCCAATCTTACCTTTTTTGATAAAATCAGCCAAACCTATCCCATCGCGGACAATCTCGGCTTTGTGCTGACGA

TCGCTGTCGTGCTCTTTGGCGCGATGCTACTGATCACCACGCTGTTATCATCGTATCGCTATGTGCTAAAGCCTGTGTTG

ATTTTGCTATTAATCATGGGCGCGGTGACCAGTTATTTTACTGACACTTATGGCACGGTCTATGATACGACCATGCTCCA

AAATGCCCTACAGACCGACCAAGCCGAGACCAAGGATCTATTAAACGCAGCGTTTATCATGCGTATCATTGGTTTGGGTG

TGCTACCAAGTTTGCTTGTGGCTTTTGTTAAGGTGGATTATCCGACTTGGGGCAAGGGTTTGATGCGCCGATTGGGCTTG

ATCGTGGCAAGTCTTGCGCTGATTTTACTGCCTGTGGTGGCGTTCAGCAGTCATTATGCCAGTTTCTTTCGCGTGCATAA

GCCGCTGCGTAGCTATGTCAATCCGATCATGCCAATCTACTCGGTGGGTAAGCTTGCCAGTATTGAGTATAAAAAAGCCA

GTGCGCCAAAAGATACCATTTATCACGCCAAAGACGCGGTACAAGCAACCAAGCCTGATATGCGTAAGCCACGCCTAGTG

GTGTTCGTCGTCGGTGAGACGGCACGCGCCGATCATGTCAGCTTCAATGGCTATGAGCGCGATACTTTCCCACAGCTTGC

CAAGATCGATGGCGTGACCAATTTTAGCAATGTCACATCGTGCGGCACATCGACGGCGTATTCTGTGCCGTGTATGTTCA

GCTATCTGGGCGCGGATGAGTATGATGTCGATACCGCCAAATACCAAGAAAATGTGCTGGATACGCTGGATCGCTTGGGC

GTAAGTATCTTGTGGCGTGATAATAATTCGGACTCAAAAGGCGTGATGGATAAGCTGCCAAAAGCGCAATTTGCCGATTA

TAAATCCGCGACCAACAACGCCATCTGCAACACCAATCCTTATAACGAATGCCGCGATGTCGGTATGCTCGTTGGCTTAG

ATGACTTTGTCGCTGCCAATAACGGCAAAGATATGCTGATCATGCTGCACCAAATGGGCAATCACGGGCCTGCGTATTTT

AAGCGATATGATGAAAAGTTTGCCAAATTCACGCCAGTGTGTGAAGGTAATGAGCTTGCCAAGTGCGAACATCAGTCCTT

GATCAATGCTTATGACAATGCCTTGCTTGCCACCGATAATTTCATCGCTCAAAGTATCCAGTGGCTGCAGACGCACAGCA

ATGCCTATGATGTCTCAATGCTGTATGTCAGCGATCATGGCGAAAGTCTGGGTGAGAACGGTGTCTATCTACATGGTATG

CCAAATGCCTTTGCACCAAAAGAACAGCGCAGTGTGCCTGCATTTTTCTGGACGGATAAGCAAACTGGCATCACGCCAAT

GGCAACCGATACCGTCCTGACCCATGACGCGATCACGCCGACATTATTAAAGCTGTTTGATGTCACCGCGGACAAAGTCA

AAGACCGCACCGCATTCATCCGCTGA

>KY283125.1 Escherichia coli strain 1256822 phosphoethanolamine--lipid A transferase MCR-1.5 (mcr-1) gene, mcr-1.5 allele, complete cds

ATGATGCAGCATACTTCTGTGTGGTACCGACGCTCGGTCAGTCCGTTTGTTCTTGTGGCGAGTGTTGCCGTTTTCTTGAC

CGCGACCGCCAATCTTACCTTTTTTGATAAAATCAGCCAAACCTATCCCATCGCGGACAATCTCGGCTTTGTGCTGACGA

TCGCTGTCGTGCTCTTTGGCGCGATGCTACTGATCACCACGCTGTTATCATCGTATCGCTATGTGCTAAAGCCTGTGTTG

ATTTTGCTATTAATCATGGGCGCGGTGACCAGTTATTTTACTGACACTTATGGCACGGTCTATGATACGACCATGCTCCA

AAATGCCCTACAGACCGACCAAGCCGAGACCAAGGATCTATTAAACGCAGCGTTTATCATGCGTATCATTGGTTTGGGTG

TGCTACCAAGTTTGCTTGTGGCTTTTGTTAAGGTGGATTATCCGACTTGGGGCAAGGGTTTGATGCGCCGATTGGGCTTG

ATCGTGGCAAGTCTTGCGCTGATTTTACTGCCTGTGGTGGCGTTCAGCAGTCATTATGCCAGTTTCTTTCGCGTGCATAA

GCCGCTGCGTAGCTATGTCAATCCGATCATGCCAATCTACTCGGTGGGTAAGCTTGCCAGTATTGAGTATAAAAAAGCCA

GTGCGCCAAAAGATACCATTTATCACGCCAAAGACGCGGTACAAGCAACCAAGCCTGATATGCGTAAGCCACGCCTAGTG

GTGTTCGTCGTCGGTGAGACGGCACGCGCCGATCATGTCAGCTTCAATGGCTATGAGCGCGATACTTTCCCACAGCTTGC

CAAGATCGATGGCGTGACCAATTTTAGCAATGTCACATCGTGCGGCACATCGACGGCGTATTCTGTGCCGTGTATGTTCA

GCTATCTGGGCGCGGATGAGTATGATGTCGATACCGCCAAATACCAAGAAAATGTGCTGGATACGCTGGATCGCTTGGGC

GTAAGTATCTTGTGGCGTGATAATAATTCGGACTCAAAAGGCGTGATGGATAAGCTGCCAAAAGCGCAATTTGCCGATTA

TAAATCCGCGACCAACAACGCCATCTGCAACACCAATCCTTATAACGAATGCCGCGATGTCGGTATGCTCGTTGGCTTAG

ATGACTTTGTCGCTGCCAATAACGGCAAAGATATGCTGATCATGCTGCACCAAATGGGCAATCACGGGCCTGCGTATTTT

AAGCGATATGATGAAAAGTTTGCCAAATTCACGCCAGTGTGTGAAGGTAATGAGCTTGCCAAGTGCGAACATCAGTCCTT

GATCAATGCTTATGACAATGCCTTGCTTGCCACCGATGATTTCATCGCTCAAAGTATCCAGTGGCTGCAGACGTACAGCA

ATGCCTATGATGTCTCAATGCTGTATGTCAGCGATCATGGCGAAAGTCTGGGTGAGAACGGTGTCTATCTACATGGTATG

CCAAATGCCTTTGCACCAAAAGAACAGCGCAGTGTGCCTGCATTTTTCTGGACGGATAAGCAAACTGGCATCACGCCAAT

GGCAACCGATACCGTCCTGACCCATGACGCGATCACGCCGACATTATTAAAGCTGTTTGATGTCACCGCGGACAAAGTCA

AAGACCGCACCGCATTCATCCGCTGA

>MT770924.1 Escherichia coli isolate 1506 phosphoethanolamine--lipid A transferase MCR-1.28 (mcr-1) gene, mcr-1.28 allele, complete cds

ATGATGCAGCATACTTCTGTGTGGTACCGACGCTCGGTCAGTCCGTTTGTTCTTGTGGCGAGTGTTGCCGTTTTCTTGAC

CGCGACCGCCAATCTTACCTTTTTTGATAAAATCAGCCAAACCTATCCCATCGCGGACAATCTCGGCTTTGTGCTGACGA

TCGCTGTCGTGCTCTTTGGCGCGATGCTACTGATCACCACGCTGTTATCATCGTATCGCTATGTGCTAAAGCCTGTGTTG

ATTTTGCTATTAATCATGGGCGCGGTGACCAGTTATTTTACTGACACTTATGGCACGGTCTATGATACGACCATGCTCCA

AAATGCCCTACAGACCGACCAAGCCGAGACCAAGGATCTATTAAACGCAGCGTTTATCATGCGTATCATTGGTTTGGGTG

TGCTACCAAGTTTGCTTGTGGCTTTTGTTAAGGTGGATTATCCGACTTGGGGCAAGGGTTTGATGCGCCGATTGGGCTTG

ATCGTGGCAAGTCTTGCGCTGATTTTACTGCCTGTGGTGGCGTTCAGCAGTCATTATGCCAGTTTCTTTCGCGTGCATAA

GCCGCTGCGTAGCTATGTCAATCCGATCATGCCAATCTACTCGGTGGGTAAGCTTGCCAGTATTGAGTATAAAAAAGCCA

ATGCGCCAAAAGATACCATTTATCACGCCAAAGACGCGGTACAAGCAACCAAGCCTGATATGCGTAAGCCACGCCTAGTG

GTGTTCGTCGTCGGTGAGACGGCACGCGCCGATCATGTCAGCTTCAATGGCTATGAGCGCGATACTTTCCCACAGCTTGC

CAAGATCGATGGCGTGACCAATTTTAGCAATGTCACATCGTGCGGCACATCGACGGCGTATTCTGTGCCGTGTATGTTCA

GCTATCTGGGCGCGGATGAGTATGATGTCGATACCGCCAAATACCAAGAAAATGTGCTGGATACGCTGGATCGCTTGGGC

GTAAGTATCTTGTGGCGTGATAATAATTCGGACTCAAAAGGCGTGATGGATAAGCTGCCAAAAGCGCAATTTGCCGATTA
TAAATCCGCGACCAACAACGCCATCTGCAACACCAATCCTTATAACGAATGCCGCGATGTCGGTATGCTCGTTGGCTTAG
ATGACTTTGTCGCTGCCAATAACGGCAAAGATATGCTGATCATGCTGCACCAAATGGGCAATCACGGGCCTGCGTATTTT
AAGCGATATGATGAAAAGTTTGCCAAATTCACGCCAGTGTGTGAAGGTAATGAGCTTGCCAAGTGCGAACATCAGTCCTT
GATCAATGCTTATGACAATGCCTTGCTTGCCACCGATGATTTCATCGCTCAAAGTATCCAGTGGCTGCAGACGCACAGCA
ATGCCTATGATGTCTCAATGCTGTATGTCAGCGATCATGGCGAAAGTCTGGGTGAGAACGGTGTCTATCTACATGGTATG
CCAAATGCCTTTGCACCAAAAGAACAGCGCAGTGTGCCTGCATTTTTCTGGACGGATAAGCAAACTGGCATCACGCCAAT
GGCAACCGATACCGTCCTGACCCATGACGCGATCACGCCGACATTATTAAAGCTGTTTGATGTCACCGCGGACAAAGTCA
AAGACCGCACCGCATTCATCCGCTGA

>NG_067237.1 Escherichia coli SAUVM_E6 mcr-1 gene for phosphoethanolamine--lipid A transferase MCR-1.25, complete CDS

ATGATGCAGCATACTTCTGTGTGGTACCGACGCTCGGTCACTCCGTTTGTTCTTGTGGCGAGTGTTGCCGTTTTCTTGAC
CGCGACCGCCAATCTTACCTTTTTTGATAAAATCAGCCAAACCTATCCCATCGCGGACAATCTCGGCTTTGTGCTGACGA
TCGCTGTCGTGCTCTTTGGCGCGATGCTACTGATCACCACGCTGTTATCATCGTATCGCTATGTGCTAAAGCCTGTGTTG
ATTTTGCTATTAATCATGGGCGCGGTGACCAGTTATTTTACTGACACTTATGGCACGGTCTATGATACGACCATGCTCCA
AAATGCCCTACAGACCGACCAAGCCGAGACCAAGGATCTATTAAACGCAGCGTTTATCATGCGTATCATTGGTTTGGGTG
TGCTACCAAGTTTGCTTGTGGCTTTTGTTAAGGTGGATTATCCGACTTGGGGCAAGGGTTTGATGCGCCGATTGGGCTTG
ATCGTGGCAAGTCTTGCGCTGATTTTACTGCCTGTGGTGGCGTTCAGCAGTCATTATGCCAGTTTCTTTCGCGTGCATAA
GCCGGTGCGTAGCTATGTCAATCCGATCATGCCAATCTACTCGGTGGGTAAGCTTGCCAGTATTGAGTATAAAAAAGCCA
GTGCGCCAAAAGATACCATTTATCACGCCAAAGACGCGGTACAAGCAACCAAGCCTGATATGCGTAAGCCACGCCTAGTG

GTGTTCGTCGTCGGTGAGACGGCACGCGCCGATCATGTCAGCTTCAATGGCTATGAGCGCGATACTTTCCCACAGCTTGC

CAAGATCGATGGCGTGACCAATTTTAGCAATGTCACATCGTGCGGCACATCGACGGCGTATTCTGTGCCGTGTATGTTCA

GCTATCTGGGCGCGGATGAGTATGATGTCGATACCGCCAAATACCAAGAAAATGTGCTGGATACGCTGGATCGCTTGGGC

GTAAGTATCTTGTGGCGTGATAATAATTCGGACTCAAAAGGCGTGATGGATAAGCTGCCAAAAGCGCAATTTGCCGATTA

TAAATCCGCGACCAACAACGCCATCTGCAACACCAATCCTTATAACGAATGCCGCGATGTCGGTATGCTCGTTGGCTTAG

ATGACTTTGTCGCTGCCAATAACGGCAAAGATATGCTGATCATGCTGCACCAAATGGGCAATCACGGGCCTGCGTATTTT

AAGCGATATGATGAAAAGTTTGCCAAATTCACGCCAGTGTGTGAAGGTAATGAGCTTGCCAAGTGCGAACATCAGTCCTT

GATCAATGCTTATGACAATGCCTTGCTTGCCACCGATGATTTCATCGCTCAAAGTATCCAGTGGCTGCAGACGCACAGCA

ATGCCTATGATGTCTCAATGCTGTATGTCAGCGATCATGGCGAAAGTCTGGGTGAGAACGGTGTCTATCTACATGGTATG

CCAAATGCCTTTGCACCAAAAGAACAGCGCAGTGTGCCTGCATTTTTCTGGACGGATAAGCAAACTGGCATCACGCCAAT

GGCAACCGATACCGTCCTGACCCATGACGCGATCACGCCGACATTATTAAAGCTGTTTGATGTCACCGCGGACAAAGTCA

AAGACCGCACCGCATTCATCCGCTGA

>MN879259.1 Escherichia coli strain SAUVM_E6 phosphoethanolamine--lipid A transferase MCR-1.25 (mcr-1) gene, mcr-1.25 allele, partial cds

ATGATGCAGCATACTTCTGTGTGGTACCGACGCTCGGTCACTCCGTTTGTTCTTGTGGCGAGTGTTGCCGTTTTCTTGAC

CGCGACCGCCAATCTTACCTTTTTTGATAAAATCAGCCAAACCTATCCCATCGCGGACAATCTCGGCTTTGTGCTGACGA

TCGCTGTCGTGCTCTTTGGCGCGATGCTACTGATCACCACGCTGTTATCATCGTATCGCTATGTGCTAAAGCCTGTGTTG

ATTTTGCTATTAATCATGGGCGCGGTGACCAGTTATTTTACTGACACTTATGGCACGGTCTATGATACGACCATGCTCCA

AAATGCCCTACAGACCGACCAAGCCGAGACCAAGGATCTATTAAACGCAGCGTTTATCATGCGTATCATTGGTTTGGGTG

TGCTACCAAGTTTGCTTGTGGCTTTTGTTAAGGTGGATTATCCGACTTGGGGCAAGGGTTTGATGCGCCGATTGGGCTTG

ATCGTGGCAAGTCTTGCGCTGATTTTACTGCCTGTGGTGGCGTTCAGCAGTCATTATGCCAGTTTCTTTCGCGTGCATAA

GCCGGTGCGTAGCTATGTCAATCCGATCATGCCAATCTACTCGGTGGGTAAGCTTGCCAGTATTGAGTATAAAAAAGCCA

GTGCGCCAAAAGATACCATTTATCACGCCAAAGACGCGGTACAAGCAACCAAGCCTGATATGCGTAAGCCACGCCTAGTG

GTGTTCGTCGTCGGTGAGACGGCACGCGCCGATCATGTCAGCTTCAATGGCTATGAGCGCGATACTTTCCCACAGCTTGC

CAAGATCGATGGCGTGACCAATTTTAGCAATGTCACATCGTGCGGCACATCGACGGCGTATTCTGTGCCGTGTATGTTCA

GCTATCTGGGCGCGGATGAGTATGATGTCGATACCGCCAAATACCAAGAAAATGTGCTGGATACGCTGGATCGCTTGGGC

GTAAGTATCTTGTGGCGTGATAATAATTCGGACTCAAAAGGCGTGATGGATAAGCTGCCAAAAGCGCAATTTGCCGATTA

TAAATCCGCGACCAACAACGCCATCTGCAACACCAATCCTTATAACGAATGCCGCGATGTCGGTATGCTCGTTGGCTTAG

ATGACTTTGTCGCTGCCAATAACGGCAAAGATATGCTGATCATGCTGCACCAAATGGGCAATCACGGGCCTGCGTATTTT

AAGCGATATGATGAAAAGTTTGCCAAATTCACGCCAGTGTGTGAAGGTAATGAGCTTGCCAAGTGCGAACATCAGTCCTT

GATCAATGCTTATGACAATGCCTTGCTTGCCACCGATGATTTCATCGCTCAAAGTATCCAGTGGCTGCAGACGCACAGCA

ATGCCTATGATGTCTCAATGCTGTATGTCAGCGATCATGGCGAAAGTCTGGGTGAGAACGGTGTCTATCTACATGGTATG

CCAAATGCCTTTGCACCAAAAGAACAGCGCAGTGTGCCTGCATTTTTCTGGACGGATAAGCAAACTGGCATCACGCCAAT

GGCAACCGATACCGTCCTGACCCATGACGCGATCACGCCGACATTATTAAAGCTGTTTGATGTCACCGCGGACAAAGTCA

AAGACCGCACCGCATTCATCCGCTGA

>MF084991.1 Escherichia coli strain N8114 Mcr-1 (mcr-1) gene, mcr-1.9 allele, complete cds

ATGGTGCAGCATACTTCTGTGTGGTACCGACGCTCGGTCGGTCCGTTTGTTCTTGTGGCGAGTGTTGCCGTTTTCTTGAC

CGCGACCGCCAATCTTACCTTTTTTGATAAAATCAGCCAAACCTATCCCATCGCGGACAATCTCGGCTTTGTGCTGACGA

TCGCTGTCGTGCTCTTTGGCGCGATGCTACTGATCACCACGCTGTTATCATCGTATCGCTATGTGCTAAAGCCTGTGTTG

ATTTTGCTATTAATCATGGGCGCGGTGACCAGTTATTTTACTGACACTTATGGCACGGTCTATGATACGACCATGCTCCA

AAATGCCCTACAGACCGACCAAGCCGAGACCAAGGATCTATTAAACGCAGCGTTTATCATGCGTATCATTGGTTTGGGTG

TGCTACCAAGTTTGCTTGTGGCTTTTGTTAAGGTGGATTATCCGACTTGGGGCAAGGGTTTGATGCGCCGATTGGGCTTG

ATCGTGGCAAGTCTTGCGCTGATTTTACTGCCTGTGGTGGCGTTCAGCAGTCATTATGCCAGTTTCTTTCGCGTGCATAA

GCCGCTGCGTAGCTATGTCAATCCGATCATGCCAATCTACTCGGTGGGTAAGCTTGCCAGTATTGAGTATAAAAAAGCCA

GTGCGCCAAAAGATACCATTTATCACGCCAAAGACGCGGTACAAGCAACCAAGCCTGATATGCGTAAGCCACGCCTAGTG

GTGTTCGTCGTCGGTGAGACGGCACGCGCCGATCATGTCAGCTTCAATGGCTATGAGCGCGATACTTTCCCACAGCTTGC

CAAGATCGATGGCGTGACCAATTTTAGCAATGTCACATCGTGCGGCACATCGACGGCGTATTCTGTGCCGTGTATGTTCA

GCTATCTGGGCGCGGATGAGTATGATGTCGATACCGCCAAATACCAAGAAAATGTGCTGGATACGCTGGATCGCTTGGGC

GTAAGTATCTTGTGGCGTGATAATAATTCGGACTCAAAAGGCGTGATGGATAAGCTGCCAAAAGCGCAATTTGCCGATTA

TAAATCCGCGACCAACAACGCCATCTGCAACACCAATCCTTATAACGAATGCCGCGATGTCGGTATGCTCGTTGGCTTAG

ATGACTTTGTCGCTGCCAATAACGGCAAAGATATGCTGATCATGCTGCACCAAATGGGCAATCACGGGCCTGCGTATTTT

AAGCGATATGATGAAAAGTTTGCCAAATTCACGCCAGTGTGTGAAGGTAATGAGCTTGCCAAGTGCGAACATCAGTCCTT

GATCAATGCTTATGACAATGCCTTGCTTGCCACCGATGATTTCATCGCTCAAAGTATCCAGTGGCTGCAGACGCACAGCA

ATGCCTATGATGTCTCAATGCTGTATGTCAGCGATCATGGCGAAAGTCTGGGTGAGAACGGTGTCTATCTACATGGTATG

CCAAATGCCTTTGCACCAAAAGAACAGCGCAGTGTGCCTGCATTTTTCTGGACGGATAAGCAAACTGGCATCACGCCAAT

GGCAACCGATACCGTCCTGACCCATGACGCGATCACGCCGACATTATTAAAGCTGTTTGATGTCACCGCGGACAAAGTCA

AAGACCGCACCGCATTCATCCGCTGA

>NG_052861.1 Escherichia coli HeN867 pHeNE867 mcr-1 gene for phosphoethanolamine--lipid A transferase MCR-1.3, complete CDS

CGCATAATTTTTTATATCAGATAAATTGTACTGGATTTCTTAAAAAATTGCAGTAAAATTGCCGCAATTATCCCACCGTT

TATTTTTTGAGTAGTTTCTCATGATGCAGCATACTTCTGTGTGGTACCGACGCTCGGTCAGTCCGTTTGTTCTTGTGGCG

AGTGTTGCCGTTTTCTTGACCGCGACCGCCAATCTTACCTTTTTTGATAAGGTCAGCCAAACCTATCCCATCGCGGACAA

TCTCGGCTTTGTGCTGACGATCGCTGTCGTGCTCTTTGGCGCGATGCTACTGATCACCACGCTGTTATCATCGTATCGCT

ATGTGCTAAAGCCTGTGTTGATTTTGCTATTAATCATGGGCGCGGTGACCAGTTATTTTACTGACACTTATGGCACGGTC

TATGATACGACCATGCTCCAAAATGCCCTACAGACCGACCAAGCCGAGACCAAGGATCTATTAAACGCAGCGTTTATCAT

GCGTATCATTGGTTTGGGTGTGCTACCAAGTTTGCTTGTGGCTTTTGTTAAGGTGGATTATCCGACTTGGGGCAAGGGTT

TGATGCGCCGATTGGGCTTGATCGTGGCAAGTCTTGCGCTGATTTTACTGCCTGTGGTGGCGTTCAGCAGTCATTATGCC

AGTTTCTTTCGCGTGCATAAGCCGCTGCGTAGCTATGTCAATCCGATCATGCCAATCTACTCGGTGGGTAAGCTTGCCAG

TATTGAGTATAAAAAAGCCAGTGCGCCAAAAGATACCATTTATCACGCCAAAGACGCGGTACAAGCAACCAAGCCTGATA

TGCGTAAGCCACGCCTAGTGGTGTTCGTCGTCGGTGAGACGGCACGCGCCGATCATGTCAGCTTCAATGGCTATGAGCGC

GATACTTTCCCACAGCTTGCCAAGATCGATGGCGTGACCAATTTTAGCAATGTCACATCGTGCGGCACATCGACGGCGTA

TTCTGTGCCGTGTATGTTCAGCTATCTGGGCGCGGATGAGTATGATGTCGATACCGCCAAATACCAAGAAAATGTGCTGG

ATACGCTGGATCGCTTGGGCGTAAGTATCTTGTGGCGTGATAATAATTCGGACTCAAAAGGCGTGATGGATAAGCTGCCA

AAAGCGCAATTTGCCGATTATAAATCCGCGACCAACAACGCCATCTGCAACACCAATCCTTATAACGAATGCCGCGATGT

CGGTATGCTCGTTGGCTTAGATGACTTTGTCGCTGCCAATAACGGCAAAGATATGCTGATCATGCTGCACCAAATGGGCA

ATCACGGGCCTGCGTATTTTAAGCGATATGATGAAAAGTTTGCCAAATTCACGCCAGTGTGTGAAGGTAATGAGCTTGCC

AAGTGCGAACATCAGTCCTTGATCAATGCTTATGACAATGCCTTGCTTGCCACCGATGATTTCATCGCTCAAAGTATCCA

GTGGCTGCAGACGCACAGCAATGCCTATGATGTCTCAATGCTGTATGTCAGCGATCATGGCGAAAGTCTGGGTGAGAACG

GTGTCTATCTACATGGTATGCCAAATGCCTTTGCACCAAAAGAACAGCGCAGTGTGCCTGCATTTTTCTGGACGGATAAG

CAAACTGGCATCACGCCAATGGCAACCGATACCGTCCTGACCCATGACGCGATCACGCCGACATTATTAAAGCTGTTTGA

TGTCACCGCGGACAAAGTCAAAGACCGCACCGCATTCATCCGCTGATTTCTCCCTGTATTTTTTCCAAACCCACCGCACA

CTCCATTCGTATTATGGGCGGTGGGGTGGGGTTTGTTATGCCGTATTTATCAAATAAACGCCTACT

>NG_055784.2 Escherichia coli 347-43491A p977565 mcr-1 gene for phosphoethanolamine--lipid A transferase MCR-1.11, complete CDS

CGCATAATTTTTTATATCAGATAAATTGTACTGGATTTCTTAAAAAATTGCAGTATAATTGCCGCAATTATCCCACCGTT

TATTTTTTGAGTAGTTTCTCATGATGCAGCATACTTCTGTGGTGTGGTACCGACGCTCGGTCAGTCCGTTTGTTCTTGTG

GCGAGTGTTGCCGTTTTCTTGACCGCGACCGCCAATCTTACCTTTTTTGATAAAATCAGCCAAACCTATCCCATCGCGGA

CAATCTCGGCTTTGTGCTGACGATCGCTGTCGTGCTCTTTGGCGCGATGCTACTGATCACCACGCTGTTATCATCGTATC

GCTATGTGCTAAAGCCTGTGTTGATTTTGCTATTAATCATGGGCGCGGTGACCAGTTATTTTACTGACACTTATGGCACG

GTCTATGATACGACCATGCTCCAAAATGCCCTACAGACCGACCAAGCCGAGACCAAGGATCTATTAAACGCAGCGTTTAT

CATGCGTATCATTGGTTTGGGTGTGCTACCAAGTTTGCTTGTGGCTTTTGTTAAGGTGGATTATCCGACTTGGGGCAAGG

GTTTGATGCGCCGATTGGGCTTGATCGTGGCAAGTCTTGCGCTGATTTTACTGCCTGTGGTGGCGTTCAGCAGTCATTAT

GCCAGTTTCTTTCGCGTGCATAAGCCGCTGCGTAGCTATGTCAATCCGATCATGCCAATCTACTCGGTGGGTAAGCTTGC

CAGTATTGAGTATAAAAAAGCCAGTGCGCCAAAAGATACCATTTATCACGCCAAAGACGCGGTACAAGCAACCAAGCCTG

ATATGCGTAAGCCACGCCTAGTGGTGTTCGTCGTCGGTGAGACGGCACGCGCCGATCATGTCAGCTTCAATGGCTATGAG

CGCGATACTTTCCCACAGCTTGCCAAGATCGATGGCGTGACCAATTTTAGCAATGTCACATCGTGCGGCACATCGACGGC

GTATTCTGTGCCGTGTATGTTCAGCTATCTGGGCGCGGATGAGTATGATGTCGATACCGCCAAATACCAAGAAAATGTGC

TGGATACGCTGGATCGCTTGGGCGTAAGTATCTTGTGGCGTGATAATAATTCGGACTCAAAAGGCGTGATGGATAAGCTG

CCAAAAGCGCAATTTGCCGATTATAAATCCGCGACCAACAACGCCATCTGCAACACCAATCCTTATAACGAATGCCGCGA

TGTCGGTATGCTCGTTGGCTTAGATGACTTTGTCGCTGCCAATAACGGCAAAGATATGCTGATCATGCTGCACCAAATGG

GCAATCACGGGCCTGCGTATTTTAAGCGATATGATGAAAAGTTTGCCAAATTCACGCCAGTGTGTGAAGGTAATGAGCTT

GCCAAGTGCGAACATCAGTCCTTGATCAATGCTTATGACAATGCCTTGCTTGCCACCGATGATTTCATCGCTCAAAGTAT

CCAGTGGCTGCAGACGCACAGCAATGCCTATGATGTCTCAATGCTGTATGTCAGCGATCATGGCGAAAGTCTGGGTGAGA

ACGGTGTCTATCTACATGGTATGCCAAATGCCTTTGCACCAAAAGAACAGCGCAGTGTGCCTGCATTTTTCTGGACGGAT

AAGCAAACTGGCATCACGCCAATGGCAACCGATACCGTCCTGACCCATGACGCGATCACGCCGACATTATTAAAGCTGTT

TGATGTCACCGCGGACAAAGTCAAAGACCGCACCGCATTCATCCGCTGATTTCTCCCTGTATTTTTCCAAACCCACCGC

ACACTCCATTCGTATTATGGGCGGTGGGGTGGGGTTTGTTATGCCGTATTTATCAAATAAACGCCTACT

>MF197562.1 Escherichia coli strain MH2-19 phosphoethanolamine transferase (mcr-1) gene, partial cds

GCAGCATACTTCTGTGTGGTACCGACGCTCGGTCAGTCCGTTTGTTCTTGTGGCGAGTGTTGCCGTTTTCTTGACCGCGA

CCGCCAATCTTACCTTTTTTGATAAAATCAGCCAAACCTATCCCATCGCGGACAATCTCGGCTTTGTGCTGACGATCGCT

GTCGTGCTCTTTGGCGCGATGCTACTGATCACCACGCTGTTATCATCGTATCGCTATGTGCTAAAGCCTGTGTTGATTTT

GCTATTAATCATGGGCGCGGTGACCAGTTATTTTACTGACACTTATGGCACGGTCTATGATACGACCATGCTCCAAAATG

CCCTACAGACCGACCAAGCCGAGACCAAGGATCTATTAAACGCAGCGTTTATCATGCGTATCATTGGTTTGGGTGTGCTA

CCAAGTTTGCTTGTGGCTTTTGTTAAGGTGGATTATCCGACTTGGGGCAAGGGTTTGATGCGCCGATTGGGCTTGATCGT

GGCAAGTCTTGCGCTGATTTTACTGCCTGTGGTGGCGTTCAGCAGTCATTATGCCAGTTTCTTTCGCGTGCATAAGCCGC

TGCGTAGCTATGTCAATCCGATCATGCCAATCTACTCGGTGGGTAAGCTTGCCAGTATTGAGTATAAAAAAGCCAGTGCG

CCAAAAGATACCATTTATCACGCCAAAGACGCGGTACAAGCAACCAAGCCTGATATGCGTAAGCCACGCCTAGTGGTGTT

CGTCGTCGGTGAGACGGCACGCGCCGATCATGTCAGCTTCAATGGCTATGAGCGCGATACTTTCCCACAGCTTGCCAAGA

TCGATGGCGTGACCAATTTTAGCAATGTCACATCGTGCGGCACATCGACGGCGTATTCTGTGCCGTGTATGTTCAGCTAT

CTGGGCGCGGATGAGTATGATGTCGATACCGCCAAATACCAAGAAAATGTGCTGGATACGCTGGATCGCTTGGGCGTAAG

TATCTTGTGGCGTGATAATAATTCGGACTCAAAAGGCGTGATGGATAAGCTGCCAAAAGCGCAATTTGCCGATTATAAAT

CCGCGACCAACAACGCCATCTGCAACACCAATCCTTATAACGAATGCCGCGATGTCGGTATGCTCGTTGGCTTAGATGAC

TTTGTCGCTGCCAATAACGGCAAAGATATGCTGATCATGCTGCACCAAATGGGCAATCACGGGCCTGCGTATTTTAAGCG

ATATGATGAAAAGTTTGCCAAATTCACGCCAGTGTGTGAAGGTAATGAGCTTGCCAAGTGCGAACATCAGTCCTTGATCA

ATGCTTATGACAATGCCTTGCTTGCCACCGATGATTTCATCGCTCAAAGTATCCAGTGGCTGCAGACGCACAGCAATGCC

TATGATGTCTCAATGCTGTATGTCAGCGATCATGGCGAAAGTCTGGGTGAGAACGGTGTCTATCTACATGGTATGCCAAA

TGCCTTTGCACCAAAAGAACAGCGCAGTGTGCCTGCATTTTTCTGGACGGATAAGCAAACTGGCATCACGCCAATGGCAA

CCGATACCGTCCTGACCCATGACGCGATCACGCCGACATTATTAAAGCTGTTTGATGTCACCGCGGACAAAGTCAAAGAC

CGCAC

>NG_067236.1 Escherichia coli SAUVM_E3 mcr-1 gene for phosphoethanolamine--lipid A transferase MCR-1.24, complete CDS

ATGATGCAGCATACTTCTGTGTGGTACCGACGCTCGGTCAGTCCGTTTGTTCTTGTGGCGAGTGTTGCCGTTTTCTTGAC

CGCGACCGCCAATCTTACCTTTTTTGATAAAATCAGCCAAACCTATCCCATCGCGGACAATCTCGGCTTTGTGCTGACGA

TCGCTGTCGTGCTCTTTGGCGCGATGCTACTGATCACCACGCTGTTATCATCGTATCGCTATGTGCTAAAGCCTGTGTTG

ATTTTGCTATTAATCATGGGCGCGGTGACCAGTTATTTTACTGACACTTATGGCACGGTCTATGATACGACCATGCTCCA

AAATGCCCTACAGACCGACCAAGCCGAGACCAAGGATCTATTAAACGCAGCGTTTATCATGCGTATCATTGGTTTGGGTG

TGCTACCAAGTTTGCTTGTGGCTTTTGTTAAGGTGGATTATCCGACTTGGGGCAAGGGTTTGATGCGCCGATTGGGCTTG

ATCGTGGCAAGTCTTGCGCCGATTTTACTGCCTGTGGTGGCGTTCAGCAGTCAGTATTCCAGTTTCTTTCGCGTGCATAA

GCCGCCATGTAGCTATGTCAATCCGATCATGCCAATCTACTCGGTGGGTAAGCTGGCCAGTACTGAGTATAAAAAAGCCA

GTGCGCCAAAAGATTCCATTTATCACGCCAAAGACGCGGTACAAGCAACCAAGCCTGATATGTGTAAGCCACGCGTAGTG

GTGTTCGTCGTCGGTGAGACGGCACGCGCCGATCACGTCAGCTTCAATGGCTATGAGCGCGATACTTTCCCACAGCTTGC

CAAGATCGATGGCGTGACCAATTTTAGCAATGTCACATCGTGCGGCACATCGACGGCGTATTTTGTGCCGTGTATGTTCA

GCTATCTGGGCGCGGATGAGTATGATGTCGATACCGCCAAATACCAAGAAAATGTGCTGGATACGCTGGATCGCTTGGGC

GTAAGTATCTTGTGGCGTGATAATAATTCGGACTCAAAAGGCGTGATGGATAAGCTGCCAAAAGCGCAATTTGCCGATTA

TAAATCCGCGACCAACAACGCCATCTGCAACACCAATCCTTATAACGAATGCCGCGATGTCGGTATGCTCGTTGGCTTAG

ATGACTTTGTCGCTGCCAATAACGGCAAAGATATGCTGATCATGCTGCACCAAATGGGCAATCACGGGCCTGCGTATTTT

AAGCGATATGATGAAAAGTTTGCCAAATTCACGCCAGTGTGTGAAGGTAATGAGCTTGCCAAGTGCGAACATCAGTCCTT

GATCAATGCTTATGACAATGCCTTGCTTGCCACCGATGATTTCATCGCTCAAAGTATCCAGTGGCTGCAGACGCACAGCA

ATGCCTATGATGTCTCAATGCTGTATGTCAGCGATCATGGCGAAAGTCTGGGTGAGAACGGTGTCTATCTACATGGTATG

CCAAATGCCTTTGCACCAAAAGAACAGCGCAGTGTGCCTGCATTTTTCTGGACGGATAAGCAAACTGGCATCACGCCAAT

GGCAACCGATACCGTCCTGACCCATGACGCGATCACGCCGACATTATTAAAGCTGTTTGATGTCACCGCGGACAAAGTCA

AAGACCGCACCGCATTCATCCGCTGA

>MN879257.1 Escherichia coli strain SAUVM_E3 phosphoethanolamine--lipid A transferase MCR-1.24 (mcr-1) gene, mcr-1.24 allele, partial cds

ATGATGCAGCATACTTCTGTGTGGTACCGACGCTCGGTCAGTCCGTTTGTTCTTGTGGCGAGTGTTGCCGTTTTCTTGAC

CGCGACCGCCAATCTTACCTTTTTTGATAAAATCAGCCAAACCTATCCCATCGCGGACAATCTCGGCTTTGTGCTGACGA

TCGCTGTCGTGCTCTTTGGCGCGATGCTACTGATCACCACGCTGTTATCATCGTATCGCTATGTGCTAAAGCCTGTGTTG

ATTTTGCTATTAATCATGGGCGCGGTGACCAGTTATTTTACTGACACTTATGGCACGGTCTATGATACGACCATGCTCCA

AAATGCCCTACAGACCGACCAAGCCGAGACCAAGGATCTATTAAACGCAGCGTTTATCATGCGTATCATTGGTTTGGGTG

TGCTACCAAGTTTGCTTGTGGCTTTTGTTAAGGTGGATTATCCGACTTGGGGCAAGGGTTTGATGCGCCGATTGGGCTTG

ATCGTGGCAAGTCTTGCGCCGATTTTACTGCCTGTGGTGGCGTTCAGCAGTCAGTATTCCAGTTTCTTTCGCGTGCATAA

GCCGCCATGTAGCTATGTCAATCCGATCATGCCAATCTACTCGGTGGGTAAGCTGGCCAGTACTGAGTATAAAAAAGCCA

GTGCGCCAAAAGATTCCATTTATCACGCCAAAGACGCGGTACAAGCAACCAAGCCTGATATGTGTAAGCCACGCGTAGTG

GTGTTCGTCGTCGGTGAGACGGCACGCGCCGATCACGTCAGCTTCAATGGCTATGAGCGCGATACTTTCCCACAGCTTGC

CAAGATCGATGGCGTGACCAATTTTAGCAATGTCACATCGTGCGGCACATCGACGGCGTATTTTGTGCCGTGTATGTTCA

GCTATCTGGGCGCGGATGAGTATGATGTCGATACCGCCAAATACCAAGAAAATGTGCTGGATACGCTGGATCGCTTGGGC

GTAAGTATCTTGTGGCGTGATAATAATTCGGACTCAAAAGGCGTGATGGATAAGCTGCCAAAAGCGCAATTTGCCGATTA

TAAATCCGCGACCAACAACGCCATCTGCAACACCAATCCTTATAACGAATGCCGCGATGTCGGTATGCTCGTTGGCTTAG

ATGACTTTGTCGCTGCCAATAACGGCAAAGATATGCTGATCATGCTGCACCAAATGGGCAATCACGGGCCTGCGTATTTT

AAGCGATATGATGAAAAGTTTGCCAAATTCACGCCAGTGTGTGAAGGTAATGAGCTTGCCAAGTGCGAACATCAGTCCTT

GATCAATGCTTATGACAATGCCTTGCTTGCCACCGATGATTTCATCGCTCAAAGTATCCAGTGGCTGCAGACGCACAGCA

ATGCCTATGATGTCTCAATGCTGTATGTCAGCGATCATGGCGAAAGTCTGGGTGAGAACGGTGTCTATCTACATGGTATG

CCAAATGCCTTTGCACCAAAAGAACAGCGCAGTGTGCCTGCATTTTTCTGGACGGATAAGCAAACTGGCATCACGCCAAT

GGCAACCGATACCGTCCTGACCCATGACGCGATCACGCCGACATTATTAAAGCTGTTTGATGTCACCGCGGACAAAGTCA

AAGACCGCACCGCATTCATCCGCTGA

>MH447366.1 Escherichia coli strain F9 phosphoethanolamine transferase MCR1 (mcr1) gene, partial cds

TCGTTTGTTCTTGTGGCGAGTGTTGCCGTTTTCTTGACCGCGACCGCCAATCTTACCTTTTTTGATAAAATCAGCCAAAC

CTATCCCATCGCGGACAATCTCGGCTTTGTGCTGACGATCGCTGTCGTGCTCTTTGGCGCGATGCTACTGATCACCACGC

TGTTATCATCGTATCGCTATGTGCTAAAGCCTGTGTTGATTTTGCTATTAATCATGGGCGCGGTGACCAGTTATTTTACT

GACACTTATGGCACGGTCTATGATACGACCATGCTCCAAAATGCCCTACAGACCGACCAAGCCGAGACCAAGGATCTATT

AAACGCAGCGTTTATCATGCGTATCATTGGTTTGGGTGTGCTACCAAGTTTGCTTGTGGCTTTTGTTAAGGTGGATTATC

CGACTTGGGGCAAGGGTTTGATGCGCCGATTGGGCTTGATCGTGGCAAGTCTTGCGCTGATTTTACTGCCTGTGGTGGCG

TTCAGCAGTCATTATGCCAGTTTCTTTCGCGTGCATAAGCCGCTGCGTAGCTATGTCAATCCGATCATGCCAATCTACTC

GGTGGGTAAGCTTGCCAGTATTGAGTATAAAAAAGCCAGTGCGCCAAAAGATACCATTTATCACGCCAAAGACGCGGTAC

AAGCAACCAAGCCTGATATGCGTAAGCCACGCCTAGTGGTGTTCGTCGTCGGTGAGACGGCACGCGCCGATCATGTCAGC

TTCAATGGCTATGAGCGCGATACTTTCCCACAGCTTGCCAAGATCGATGGCGTGACCAATTTTAGCAATGTCACATCGTG

CGGCACATCGACGGCGTATTCTGTGCCGTGTATGTTCAGCTATCTGGGCGCGGATGAGTATGATGTCGATACCGCCAAAT

ACCAAGAAAATGTGCTGGATACGCTGGATCGCTTGGGCGTAAGTATCTTGTGGCGTGATAATAATTCGGACTCAAAAGGC

GTGATGGATAAGCTGCCAAAAGCGCAATTTGCCGATTATAAATCCGCGACCAACAACGCCATCTGCAACACCAATCCTTA

TAACGAATGCCGCGATGTCGGTATGCTCGTTGGCTTAGATGACTTTGTCGCTGCCAATAACGGCAAAGATATGCTGATCA

TGCTGCACCAAATGGGCAATCACGGGCCTGCGTATTTTAAGCGATATGATGAAAAGTTTGCCAAATTCACGCCAGTGTGT

GAAGGTAATGAGCTTGCCAAGTGCGAACATCAGTCCTTGATCAATGCTTATGACAATGCCTTGCTTGCCACCGATGATTT

CATCGCTCAAAGTATCCAGTGGCTGCAGACGCACAGCAATGCCTATGATGTCTCAATGCTGTATGTCAGCGATCATGGCG

AAAGTCTGGGTGAGAACGGTGTCTATCTACATGGTATGCCAAATGCCTTTGCACCAAAAGAACAGCGCAGTGTGCCTGCA

TTTTTCTGGACGGATAAGCAAACTGGCATCACGCCAATGGCAACCGATACCGTCCTGACCCATGACGCGATCACGCCGAC

ATTATTAAAGCTGTTGA

>MH447367.1 Escherichia coli strain F33 phosphoethanolamine transferase MCR1 (mcr1) gene, partial cds

TCGTTTGTTCTTGTGGCGAGTGTTGCCGTTTTCTTGACCGCGACCGCCAATCTTACCTTTTTGATAAAATCAGCCAAAC

CTATCCCATCGCGGACAATCTCGGCTTTGTGCTGACGATCGCTGTCGTGCTCTTTGGCGCGATGCTACTGATCACCACGC

TGTTATCATCGTATCGCTATGTGCTAAAGCCTGTGTTGATTTTGCTATTAATCATGGGCGCGGTGACCAGTTATTTTACT

GACACTTATGGCACGGTCTATGATACGACCATGCTCCAAAATGCCCTACAGACCGACCAAGCCGAGACCAAGGATCTATT

AAACGCAGCGTTTATCATGCGTATCATTGGTTTGGGTGTGCTACCAAGTTTGCTTGTGGCTTTTGTTAAGGTGGATTATC

CGACTTGGGGCAAGGGTTTGATGCGCCGATTGGGCTTGATCGTGGCAAGTCTTGCGCTGATTTTACTGCCTGTGGTGGCG

TTCAGCAGTCATTATGCCAGTTTCTTTCGCGTGCATAAGCCGCTGCGTAGCTATGTCAATCCGATCATGCCAATCTACTC

GGTGGGTAAGCTTGCCAGTATTGAGTATAAAAAAGCCAGTGCGCCAAAAGATACCATTTATCACGCCAAAGACGCGGTAC

AAGCAACCAAGCCTGATATGCGTAAGCCACGCCTAGTGGTGTTCGTCGTCGGTGAGACGGCACGCGCCGATCATGTCAGC

TTCAATGGCTATGAGCGCGATACTTTCCCACAGCTTGCCAAGATCGATGGCGTGACCAATTTTAGCAATGTCACATCGTG

CGGCACATCGACGGCGTATTCTGTGCCGTGTATGTTCAGCTATCTGGGCGCGGATGAGTATGATGTCGATACCGCCAAAT

ACCAAGAAAATGTGCTGGATACGCTGGATCGCTTGGGCGTAAGTATCTTGTGGCGTGATAATAATTCGGACTCAAAAGGC

GTGATGGATAAGCTGCCAAAAGCGCAATTTGCCGATTATAAATCCGCGACCAACAACGCCATCTGCAACACCAATCCTTA

TAACGAATGCCGCGATGTCGGTATGCTCGTTGGCTTAGATGACTTTGTCGCTGCCAATAACGGCAAAGATATGCTGATCA

TGCTGCACCAAATGGGCAATCACGGGCCTGCGTATTTTAAGCGATATGATGAAAAGTTTGCCAAATTCACGCCAGTGTGT

GAAGGTAATGAGCTTGCCAAGTGCGAACATCAGTCCTTGATCAATGCTTATGACAATGCCTTGCTTGCCACCGATGATTT

CATCGCTCAAAGTATCCAGTGGCTGCAGACGCACAGCAATGCCTATGATGTCTCAATGCTGTATGTCAGCGATCATGGCG

AAAGTCTGGGTGAGAACGGTGTCTATCTACATGGTATGCCAAATGCCTTTGCACCAAAAGAACAGCGCAGTGTGCCTGCA

TTTTTCTGGACGGATAAGCAAACTGGCATCACGCCAATGGCAACCGATACCGTCCTGACCCATGACGCGATCACGCCGAC

A

>KY218739.1 Escherichia coli strain W5-6 phosphoethanolamine--lipid A transferase MCR-1 (mcr-1) gene, partial cds

TTTGTTCTTGTGGCGAGTGTTGCCGTTTTCTTGACCGCGACCGCCAATCTTACCTTTTTTGATAAAATCAGCCAAACCTA

TCCCATCGCGGACAATCTCGGCTTTGTGCTGACGATCGCTGTCGTGCTCTTTGGCGCGATGCTACTGATCACCACGCTGT

TATCATCGTATCGCTATGTGCTAAAGCCTGTGTTGATTTTGCTATTAATCATGGGCGCGGTGACCAGTTATTTTACTGAC

ACTTATGGCACGGTCTATGATACGACCATGCTCCAAAATGCCCTACAGACCGACCAAGCCGAGACCAAGGATCTATTAAA

CGCAGCGTTTATCATGCGTATCATTGGTTTGGGTGTGCTACCAAGTTTGCTTGTGGCTTTTGTTAAGGTGGATTATCCGA

CTTGGGGCAAGGGTTTGATGCGCCGATTGGGCTTGATCGTGGCAAGTCTTGCGCTGATTTTACTGCCTGTGGTGGCGTTC

AGCAGTCATTATGCCAGTTTCTTTCGCGTGCATAAGCCGCTGCGTAGCTATGTCAATCCGATCATGCCAATCTACTCGGT

GGGTAAGCTTGCCAGTATTGAGTATAAAAAAGCCAGTGCGCCAAAAGATACCATTTATCACGCCAAAGACGCGGTACAAG

CAACCAAGCCTGATATGCGTAAGCCACGCCTAGTGGTGTTCGTCGTCGGTGAGACGGCACGCGCCGATCATGTCAGCTTC

AATGGCTATGAGCGCGATACTTTCCCACAGCTTGCCAAGATCGATGGCGTGACCAATTTTAGCAATGTCACATCGTGCGG

CACATCGACGGCGTATTCTGTGCCGTGTATGTTCAGCTATCTGGGCGCGGATGAGTATGATGTCGATACCGCCAAATACC

AAGAAAATGTGCTGGATACGCTGGATCGCTTGGGCGTAAGTATCTTGTGGCGTGATAATAATTCGGACTCAAAAGGCGTG

ATGGATAAGCTGCCAAAAGCGCAATTTGCCGATTATAAATCCGCGACCAACAACGCCATCTGCAACACCAATCCTTATAA

CGAATGCCGCGATGTCGGTATGCTCGTTGGCTTAGATGACTTTGTCGCTGCCAATAACGGCAAAGATATGCTGATCATGC

TGCACCAAATGGGCAATCACGGGCCTGCGTATTTTAAGCGATATGATGAAAAGTTTGCCAAATTCACGCCAGTGTGTGAA

GGTAATGAGCTTGCCAAGTGCGAACATCAGTCCTTGATCAATGCTTATGACAATGCCTTGCTTGCCACCGATGATTTCAT
CGCTCAAAGTATCCAGTGGCTGCAGACGCACAGCAATGCCTATGATGTCTCAATGCTGTATGTCAGCGATCATGGCGAAA
GTCTGGGTGAGAACGGTGTCTATCTACATGGTATGCCAAATGCCTTTGCACCAAAAGAACAGCGCAGTGTGCCTGCATTT
TTCTGGACGGATAAGCAAACTGGCATCACGCCAATGGCAACCGATACCGTCCTGACCCATGACGCGATCACGCCGACA

>KY218738.1 Escherichia coli strain W2-5 phosphoethanolamine--lipid A transferase MCR-1 (mcr-1) gene, partial cds

TTTGTTCTTGTGGCGAGTGTTGCCGTTTTCTTGACCGCGACCGCCAATCTTACCTTTTTTGATAAAATCAGCCAAACCTA
TCCCATCGCGGACAATCTCGGCTTTGTGCTGACGATCGCTGTCGTGCTCTTTGGCGCGATGCTACTGATCACCACGCTGT
TATCATCGTATCGCTATGTGCTAAAGCCTGTGTTGATTTTGCTATTAATCATGGGCGCGGTGACCAGTTATTTTACTGAC
ACTTATGGCACGGTCTATGATACGACCATGCTCCAAAATGCCCTACAGACCGACCAAGCCGAGACCAAGGATCTATTAAA
CGCAGCGTTTATCATGCGTATCATTGGTTTGGGTGTGCTACCAAGTTTGCTTGTGGCTTTTGTTAAGGTGGATTATCCGA
CTTGGGGCAAGGGTTTGATGCGCCGATTGGGCTTGATCGTGGCAAGTCTTGCGCTGATTTTACTGCCTGTGGTGGCGTTC
AGCAGTCATTATGCCAGTTTCTTTCGCGTGCATAAGCCGCTGCGTAGCTATGTCAATCCGATCATGCCAATCTACTCGGT
GGGTAAGCTTGCCAGTATTGAGTATAAAAAAGCCAGTGCGCCAAAAGATACCATTTATCACGCCAAAGACGCGGTACAAG
CAACCAAGCCTGATATGCGTAAGCCACGCCTAGTGGTGTTCGTCGTCGGTGAGACGGCACGCGCCGATCATGTCAGCTTC
AATGGCTATGAGCGCGATACTTTCCCACAGCTTGCCAAGATCGATGGCGTGACCAATTTTAGCAATGTCACATCGTGCGG
CACATCGACGGCGTATTCTGTGCCGTGTATGTTCAGCTATCTGGGCGCGGATGAGTATGATGTCGATACCGCCAAATACC
AAGAAAATGTGCTGGATACGCTGGATCGCTTGGGCGTAAGTATCTTGTGGCGTGATAATAATTCGGACTCAAAAGGCGTG
ATGGATAAGCTGCCAAAAGCGCAATTTGCCGATTATAAATCCGCGACCAACAACGCCATCTGCAACACCAATCCTTATAA
CGAATGCCGCGATGTCGGTATGCTCGTTGGCTTAGATGACTTTGTCGCTGCCAATAACGGCAAAGATATGCTGATCATGC

TGCACCAAATGGGCAATCACGGGCCTGCGTATTTTAAGCGATATGATGAAAAGTTTGCCAAATTCACGCCAGTGTGTGAA

GGTAATGAGCTTGCCAAGTGCGAACATCAGTCCTTGATCAATGCTTATGACAATGCCTTGCTTGCCACCGATGATTTCAT

CGCTCAAAGTATCCAGTGGCTGCAGACGCACAGCAATGCCTATGATGTCTCAATGCTGTATGTCAGCGATCATGGCGAAA

GTCTGGGTGAGAACGGTGTCTATCTACATGGTATGCCAAATGCCTTTGCACCAAAAGAACAGCGCAGTGTGCCTGCATTT

TTCTGGACGGATAAGCAAACTGGCATCACGCCAATGGCAACCGATACCGTCCTGACCCATGACGCGATCACGCCGACA

>KY218737.1 Escherichia coli strain BE2-5 phosphoethanolamine--lipid A transferase MCR-1 (mcr-1) gene, partial cds

TTTGTTCTTGTGGCGAGTGTTGCCGTTTTCTTGACCGCGACCGCCAATCTTACCTTTTTTGATAAAATCAGCCAAACCTA

TCCCATCGCGGACAATCTCGGCTTTGTGCTGACGATCGCTGTCGTGCTCTTTGGCGCGATGCTACTGATCACCACGCTGT

TATCATCGTATCGCTATGTGCTAAAGCCTGTGTTGATTTTGCTATTAATCATGGGCGCGGTGACCAGTTATTTTACTGAC

ACTTATGGCACGGTCTATGATACGACCATGCTCCAAAATGCCCTACAGACCGACCAAGCCGAGACCAAGGATCTATTAAA

CGCAGCGTTTATCATGCGTATCATTGGTTTGGGTGTGCTACCAAGTTTGCTTGTGGCTTTTGTTAAGGTGGATTATCCGA

CTTGGGGCAAGGGTTTGATGCGCCGATTGGGCTTGATCGTGGCAAGTCTTGCGCTGATTTTACTGCCTGTGGTGGCGTTC

AGCAGTCATTATGCCAGTTTCTTTCGCGTGCATAAGCCGCTGCGTAGCTATGTCAATCCGATCATGCCAATCTACTCGGT

GGGTAAGCTTGCCAGTATTGAGTATAAAAAAGCCAGTGCGCCAAAAGATACCATTTATCACGCCAAAGACGCGGTACAAG

CAACCAAGCCTGATATGCGTAAGCCACGCCTAGTGGTGTTCGTCGTCGGTGAGACGGCACGCGCCGATCATGTCAGCTTC

AATGGCTATGAGCGCGATACTTTCCCACAGCTTGCCAAGATCGATGGCGTGACCAATTTTAGCAATGTCACATCGTGCGG

CACATCGACGGCGTATTCTGTGCCGTGTATGTTCAGCTATCTGGGCGCGGATGAGTATGATGTCGATACCGCCAAATACC

AAGAAAATGTGCTGGATACGCTGGATCGCTTGGGCGTAAGTATCTTGTGGCGTGATAATAATTCGGACTCAAAAGGCGTG

ATGGATAAGCTGCCAAAAGCGCAATTTGCCGATTATAAATCCGCGACCAACAACGCCATCTGCAACACCAATCCTTATAA

CGAATGCCGCGATGTCGGTATGCTCGTTGGCTTAGATGACTTTGTCGCTGCCAATAACGGCAAAGATATGCTGATCATGC
TGCACCAAATGGGCAATCACGGGCCTGCGTATTTTAAGCGATATGATGAAAAGTTTGCCAAATTCACGCCAGTGTGTGAA
GGTAATGAGCTTGCCAAGTGCGAACATCAGTCCTTGATCAATGCTTATGACAATGCCTTGCTTGCCACCGATGATTTCAT
CGCTCAAAGTATCCAGTGGCTGCAGACGCACAGCAATGCCTATGATGTCTCAATGCTGTATGTCAGCGATCATGGCGAAA
GTCTGGGTGAGAACGGTGTCTATCTACATGGTATGCCAAATGCCTTTGCACCAAAAGAACAGCGCAGTGTGCCTGCATTT
TTCTGGACGGATAAGCAAACTGGCATCACGCCAATGGCAACCGATACCGTCCTGACCCATGACGCGATCACGCCGACA
>MK030127.2 Escherichia coli strain SS30 MCR family phosphoethanolamine--lipid A transferase (mcr) gene, partial cds
TCTTGTGGCGAGTGTTGCCGTTTTCTTGACCGCGACCGCCAATCTTACCTTTTTTGATAAAATCAGCCAAACCTATCCCA
TCGCGGACAATCTCGGCTTTGTGCTGACGATCGCTGTCGTGCTCTTTGGCGCGATGCTACTGATCACCACGCTGTTATCA
TCGTATCGCTATGTGCTAAAGCCTGTGTTGATTTTGCTATTAATCATGGGCGCGGTGACCAGTTATTTTACTGACACTTA
TGGCACGGTCTATGATACGACCATGCTCCAAAATGCCCTACAGACCGACCAAGCCGAGACCAAGGATCTATTAAACGCAG
CGTTTATCATGCGTATCATTGGTTTGGGTGTGCTACCAAGTTTGCTTGTGGCTTTTGTTAAGGTGGATTATCCGACTTGG
GGCAAGGGTTTGATGCGCCGATTGGGCTTGATCGTGGCAAGTCTTGCGCTGATTTTACTGCCTGTGGTGGCGTTCAGCAG
TCATTATGCCAGTTTCTTTCGCGTGCATAAGCCGCTGCGTAGCTATGTCAATCCGATCATGCCAATCTACTCGGTGGGTA
AGCTTGCCAGTATTGAGTATAAAAAAGCCAGTGCGCCAAAAGATACCATTTATCACGCCAAAGACGCGGTACAAGCAACC
AAGCCTGATATGCGTAAGCCACGCCTAGTGGTGTTCGTCGTCGGTGAGACGGCACGCGCCGATCATGTCAGCTTCAATGG
CTATGAGCGCGATACTTTCCCACAGCTTGCCAAGATCGATGGCGTGACCAATTTTAGCAATGTCACATCGTGCGGCACAT
CGACGGCGTATTCTGTGCCGTGTATGTTCAGCTATCTGGGCGCGGATGAGTATGATGTCGATACCGCCAAATACCAAGAA
AATGTGCTGGATACGCTGGATCGCTTGGGCGTAAGTATCTTGTGGCGTGATAATAATTCGGACTCAAAAGGCGTGATGGA

TAAGCTGCCAAAAGCGCAATTTGCCGATTATAAATCCGCGACCAACAACGCCATCTGCAACACCAATCCTTATAACGAAT
GCCGCGATGTCGGTATGCTCGTTGGCTTAGATGACTTTGTCGCTGCCAATAACGGCAAAGATATGCTGATCATGCTGCAC
CAAATGGGCAATCACGGGCCTGCGTATTTTAAGCGATATGATGAAAAGTTTGCCAAATTCACGCCAGTGTGTGAAGGTAA
TGAGCTTGCCAAGTGCGAACATCAGTCCTTGATCAATGCTTATGACAATGCCTTGCTTGCCACCGATGATTTCATCGCTC
AAAGTATCCAGTGGCTGCAGACGCACAGCAATGCCTATGATGTCTCAATGCTGTATGTCAGCGATCATGGCGAAAGTCTG
GGTGAGAACGGTGTCTATCTACATGGTATGCCAAATGCCTTTGCACCAAAAGAACAGCGCAGTGTGCCTGCATTTTTCTG
GACGGATAAGCAAACTGGCATCACGCCAATGGCAACCGATACCGTCCTGACCCATGACGCGATCACGCCGACATTAT

>MK030126.2 Escherichia coli strain SS20 MCR family phosphoethanolamine--lipid A transferase (mcr) gene, partial cds

GACCGTTCCTGATAGTCGTTGTTCTTGTGGCGAGTGTTGCCGTTTTCTTGACCGCGACCGCCAATCTTACCTTTTTGAT
AAAATCAGCCAAACCTATCCCATCGCGGACAATCTCGGCTTTGTGCTGACGATCGCTGTCGTGCTCTTTGGCGCGATGCT
ACTGATCACCACGCTGTTATCATCGTATCGCTATGTGCTAAAGCCTGTGTTGATTTTGCTATTAATCATGGGCGCGGTGA
CCAGTTATTTTACTGACACTTATGGCACGGTCTATGATACGACCATGCTCCAAAATGCCCTACAGACCGACCAAGCCGAG
ACCAAGGATCTATTAAACGCAGCGTTTAACAGGCGTATCACTGGTTTGGGTGTGCTACCAAGTTTGCTTGTGGCTTTTGT
TAAGGTGGATTATCCGACTTGGGGCAAGGGTTTGATGCGCCGATTGGGCTTGATCGTGGCAAGTCTTGCGCTGATTTTAC
TGCCTGTGGTGGCGTTCAGCAGTCATTATGCCAGTTTCTTTCGCGTGCATAAGCCGCTGCGTAGCTATGTCAATCCGATC
ATGCCAATCTACTCGGTGGGTAAGCTTGCCAGTATTGAGTATAAAAAAGCCAGTGCGCCAAAAGATACCATTTATCACGC
CAAAGACGCGGTACAAGCAACCAAGCCTGATATGCGTAAGCCACGCCTAGTGGTGTTCGTCGTCGGTGAGACGGCACGCG
CCGATCATGTCAGCTTCAATGGCTATGAGCGCGATACTTTCCCACAGCTTGCCAAGATCGATGGCGTGACCAATTTTAGC
AATGTCACATCGTGCGGCACATCGACGGCGTATTCTGTGCCGTGTATGTTCAGCTATCTGGGCGCGGATGAGTATGATGT

CGATACCGCCAAATACCAAGAAAATGTGCTGGATACGCTGGATCGCTTGGGCGTAAGTATCTTGTGGCGTGATAATAATT

CGGACTCAAAAGGCGTGATGGATAAGCTGCCAAAAGCGCAATTTGCCGATTATAAATCCGCGACCAACAACGCCATCTGC

AACACCAATCCTTATAACGAATGCCGCGATGTCGGTATGCTCGTTGGCTTAGATGACTTTGTCGCTGCCAATAACGGCAA

AGATATGCTGATCATGCTGCACCAAATGGGCAATCACGGGCCTGCGTTTTTTAAGCGATATGATGAAAAGTTTGCCAAAT

TCACGCCAGTGTGTGAAGGTAATGAGCTTGCCAAGTGCGAACATCAGTCCTTGATCAATGCTTATGACAATGCCTTGCTT

GCCACCGATGATTTCATCGCTCAAAGTATCCAGTGGCTGCAGACGCACAGCAATGCCTATGATGTCTCAATGCTGTATGT

CAGCGATCATGGCGAAAGTCTGGGTGAGAACGGTGTCTATCTACATGGTATGCCAAATGCCTTTGCACCAAAAGAACAGC

GCAGTGTGCCTGCATTTTTCTGGACGGATAAGCAAACTGGCATCACGCCAATGGCAACCGATACCGTCCTGACCCATGAC

GCGATCACGCCGACATTA

>KY013597.1 Escherichia coli strain C2-007R phosphoethanolamine--lipid A transferase (mcr-1) gene, partial cds

CGGTCAGTCCGTTTGTTCTTGTGGCGAGTGTTGCCGTTTTCTTGACCGCGACCGCCAATCTTACCTTTTTTGATAAAATC

AGCCAAACCTATCCCATCGCGGACAATCTCGGCTTTGTGCTGACGATCGCTGTCGTGCTCTTTGGCGCGATGCTACTGAT

CACCACGCTGTTATCATCGTATCGCTATGTGCTAAAGCCTGTGTTGATTTTGCTATTAATCATGGGCGCGGTGACCAGTT

ATTTTACTGACACTTATGGCACGGTCTATGATACGACCATGCTCCAAAATGCCCTACAGACCGACCAAGCCGAGACCAAG

GATCTATTAAACGCAGCGTTTATCATGCGTATCATTGGTTTGGGTGTGCTACCAAGTTTGCTTGTGGCTTTTGTTAAGGT

GGATTATCCGACTTGGGGCAAGGGTTTGATGCGCCGATTGGGCTTGATCGTGGCAAGTCTTGCGCTGATTTTACTGCCTG

TGGTGGCGTTCAGCAGTCATTATGCCAGTTTCTTTCGCGTGCATAAGCCGCTGCGTAGCTATGTCAATCCGATCATGCCA

ATCTACTCGGTGGGTAAGCTTGCCAGTATTGAGTATAAAAAAGCCAGTGCGCCAAAAGATACCATTTATCACGCCAAAGA

CGCGGTACAAGCAACCAAGCCTGATATGCGTAAGCCACGCCTAGTGGTGTTCGTCGTCGGTGAGACGGCACGCGCCGATC

ATGTCAGCTTCAATGGCTATGAGCGCGATACTTTCCCACAGCTTGCCAAGATCGATGGCGTGACCAATTTTAGCAATGTC

ACATCGTGCGGCACATCGACGGCGTATTCTGTGCCGTGTATGTTCAGCTATCTGGGCGCGGATGAGTATGATGTCGATAC

CGCCAAATACCAAGAAAATGTGCTGGATACGCTGGATCGCTTGGGCGTAAGTATCTTGTGGCGTGATAATAATTCGGACT

CAAAAGGCGTGATGGATAAGCTGCCAAAAGCGCAATTTGCCGATTATAAATCCGCGACCAACAACGCCATCTGCAACACC

AATCCTTATAACGAATGCCGCGATGTCGGTATGCTCGTTGGCTTAGATGACTTTGTCGCTGCCAATAACGGCAAAGATAT

GCTGATCATGCTGCACCAAATGGGCAATCACGGGCCTGCGTATTTTAAGCGATATGATGAAAAGTTTGCCAAATTCACGC

CAGTGTGTGAAGGTAATGAGCTTGCCAAGTGCGAACATCAGTCCTTGATCAATGCTTATGACAATGCCTTGCTTGCCACC

GATGATTTCATCGCTCAAAGTATCCAGTGGCTGCAGACGCACAGCAATGCCTATGATGTCTCAATGCTGTATGTCAGCGA

TCATGGCGAAAGTCTGGGTGAGAACGGTGTCTATCTACATGGTATGCCAAATGCCTTTGCACCAAAAGAACAGCGCAGTG

TGCCTGCATTTTTCTGGACGGATAAGCAAACTGGCATCACGCCAATGGCAACCGATACCGTCCTGACC

>MN148432.1 Escherichia coli strain RShimy4 MCR-1 family phosphoethanolamine--lipid A transferase (mcr-1) gene, partial cds

ATGCTACTGATCACCACGCTGTTATCATCGTATCGCTATGTGCTAAAGCCTGTGTTGATTTTGCTATTAATCATGGGCGC

GGTGACCAGTTATTTTACTGACACTTATGGCACGGTCTATGATACGACCATGCTCCAAAATGCCCTACAGACCGACCAAG

CCGAGACCAAGGATCTATTAAACGCAGCGTTTATCATGCGTATCATTGGTTTGGGTGTGCTACCAAGTTTGCTTGTGGCT

TTTGTTAAGGTGGATTATCCGACTTGGGGCAAGGGTTTGATGCGCCGATTGGGCTTGATCGTGGCAAGTCTTGCGCTGAT

TTTACTGCCTGTGGTGGCGTTCAGCAGTCATTATGCCAGTTTCTTTCGCGTGCATAAGCCGCTGCGTAGCTATGTCAATC

CGATCATGCCAATCTACTCGGTGGGTAAGCTTGCCAGTATTGAGTATAAAAAAGCCAGTGCGCCAAAAGATACCATTTAT

CACGCCAAAGACGCGGTACAAGCAACCAAGCCTGATATGCGTAAGCCACGCCTAGTGGTGTTCGTCGTCGGTGAGACGGC

ACGCGCCGATCATGTCAGCTTCAATGGCTATGAGCGCGATACTTTCCCACAGCTTGCCAAGATCGATGGCGTGACCAATT

TTAGCAATGTCACATCGTGCGGCACATCGACGGCGTATTCTGTGCCGTGTATGTTCAGCTATCTGGGCGCGGATGAGTAT

GATGTCGATACCGCCAAATACCAAGAAAATGTGCTGGATACGCTGGATCGCTTGGGCGTAAGTATCTTGTGGCGTGATAA

TAATTCGGACTCAAAAGGCGTGATGGATAAGCTGCCAAAAGCGCAATTTGCCGATTATAAATCCGCGACCAACAACGCCA

TCTGCAACACCAATCCTTATAACGAATGCCGCGATGTCGGTATGCTCGTTGGCTTAGATGACTTTGTCGCTGCCAATAAC

GGCAAAGATATGCTGATCATGCTGCACCAAATGGGCAATCACGGGCCTGCGTATTTTAAGCGATATGATGAAAAGTTTGC

CAAATTCACGCCAGTGTGTGAAGGTAATGAGCTTGCCAAGTGCGAACATCAGTCCTTGATCAATGCTTATGACAATGCCT

TGCTTGCCACCGATGATTTCATCGCTCAAAGTATCCAGTGGCTGCAGACGCACAGCAATGCCTATGATGTCTCAATGCTG

TATGTCAGCGATCATGGCGAAAGTCTGGGTGAGAACGGTGTCTATCTACATGGTATGCCAAATGCCTTTGCACCAAAAGA

ACAGCGCAGTGTGCCTGCATTTTTCTGGACGGATAAGCAAACTGGCATCACGCCAATGGCAACCGATACCGTCCTGACCC

ATGACGCGATCACGCCGACATTAT

>MF134664.1 Escherichia coli strain 1-IM phosphoethanolamine transferase gene, partial cds

CACTTATGGCACGGTCTATGATACGACCATGCTCCAAAATGCCCTACAGACCGACCAAGCCGAGACCAAGGATCTATTAA

ACGCAGCGTTTATCATGCGTATCATTGGTTTGGGTGTGCTACCAAGTTTGCTTGTGGCTTTTGTTAAGGTGGATTATCCG

ACTTGGGGCAAGGGTTTGATGCGCCGATTGGGCTTGATCGTGGCAAGTCTTGCGCTGATTTTACTGCCTGTGGTGGCGTT

CAGCAGTCATTATGCCAGTTTCTTTCGCGTGCATAAGCCGCTGCGTAGCTATGTCAATCCGATCATGCCAATCTACTCGG

TGGGTAAGCTTGCCAGTATTGAGTATAAAAAAGCCAGTGCGCCAAAAGATACCATTTATCACGCCAAAGACGCGGTACAA

GCAACCAAGCCTGATATGCGTAAGCCACGCCTAGTGGTGTTCGTCGTCGGTGAGACGGCACGCGCCGATCATGTCAGCTT

CAATGGCTATGAGCGCGATACTTTCCCACAGCTTGCCAAGATCGATGGCGTGACCAATTTTAGCAATGTCACATCGTGCG

GCACATCGACGGCGTATTCTGTGCCGTGTATGTTCAGCTATCTGGGCGCGGATGAGTATGATGTCGATACCGCCAAATAC

CAAGAAAATGTGCTGGATACGCTGGATCGCTTGGGCGTAAGTATCTTGTGGCGTGATAATAATTCGGACTCAAAAGGCGT

GATGGATAAGCTGCCAAAAGCGCAATTTGCCGATTATAAATCCGCGACCAACAACGCCATCTGCAACACCAATCCTTATA

ACGAATGCCGCGATGTCGGTATGCTCGTTGGCTTAGATGACTTTGTCGCTGCCAATAACGGCAAAGATATGCTGATCATG

CTGCACCAAATGGGCAATCACGGGCCTGCGTATTTTAAGCGATATGATGAAAAGTTTGCCAAATTCACGCCAGTGTGTGA

AGGTAATGAGCTTGCCAAGTGCGAACATCAGTCCTTGATCAATGCTTATGACAATGCCTTGCTTGCCACCGATGATTTCA

TCGCTCAAAGTATCCAGTGGCTGCAGACGCACAGCAATGCCTATGATGTCTCAATGCTGTATGTCAGCGATCATGGCGAA

AGTCTGGGTGAGAACGGTGTCTATC

>MG763104.1 Escherichia coli plasmid Mcr-1 (mcr-1) gene, partial cds

CCGTTTGTTCTTGTGGCGAGTGTTGCCGTTTTCTTGACCGCGACCGCCAATCTTACCTTTTTTGATAAAATCAGCCAAAC

CTATCCCATCGCGGACAATCTCGGCTTTGTGCTGACGATCGCTGTCGTGCTCTTTGGCGCGATGCTACTGATCACCACGC

TGTTATCATCGTATCGCTATGTGCTAAAGCCTGTGTTGATTTTGCTATTAATCATGGGCGCGGTGACCAGTTATTTTACT

GACACTTATGGCACGGTCTATGATACGACCATGCTCCAAAATGCCCTACAGACCGACCAAGCCGAGACCAAGGATCTATT

AAACGCAGCGTTTATCATGCGTATCATTGGTTTGGGTGTGCTACCAAGTTTGCTTGTGGCTTTTGTTAAGGTGGATTATC

CGACTTGGGGCAAGGGTTTGATGCGCCGATTGGGCTTGATCGTGGCAAGTCTTGCGCTGATTTTACTGCCTGTGGTGGCG

TTCAGCAGTCATTATGCCAGTTTCTTTCGCGTGCATAAGCCGCTGCGTAGCTATGTCAATCCGATCATGCCAATCTACTC

GGTGGGTAAGCTTGCCAGTATTGAGTATAAAAAAGCCAGTGCGCCAAAAGATACCATTTATCACGCCAAAGACGCGGTAC

AAGCAACCAAGCCTGATATGCGTAAGCCACGCCTAGTGGTGTTCGTCGTCGGTGAGACGGCACGCGCCGATCATGTCAGC

TTCAATGGCTATGAGCGCGATACTTTCCCACAGCTTGCCAAGATCGATGGCGTGACCAATTTTAGCAATGTCACATCGTG

CGGCACATCGACGGCGTATTCTGTGCCGTGTATGTTCAGCTATCTGGGCGCGGATGAGTATGATGTCGATACCGCCAAAT

ACCAAGAAAATGTGCTGGATACGCTGGATCGCTTGGGCGTAAGTATCTTGTGGCGTGATAATAATTCGGACTCAAAAGGC
GTGATGGATAA

>MH425550.1 Escherichia coli strain 298 clone MCR1-3-CLR5-1-F_H03 polymyxin resistance protein MCR-1 (mcr-1) gene, partial cds

GTTCTTGTGGCGAGTGTTGCCGTTTTCTTGACCGCGACCGCCAATCTTACCTTTTTGATAAAATCAGCCAAACCTATCC
CATCGCGGACAATCTCGGCTTTGTGCTGACGATCGCTGTCGTGCTCTTTGGCGCGATGCTACTGATCACCACGCTGTTAT
CATCGTATCGCTATGTGCTAAAGCCTGTGTTGATTTTGCTATTAATCATGGGCGCGGTGACCAGTTATTTTACTGACACT
TATGGCACGGTCTATGATACGACCATGCTCCAAAATGCCCTACAGACCGACCAAGCCGAGACCAAGGATCTATTAAACGC
AGCGTTTATCATGCGTATCATTGGTTTGGGTGTGCTACCAAGTTTGCTTGTGGCTTTTGTTAAGGTGGATTATCCGACTT
GGGGCAAGGGTTTGATGCGCCGATTGGGCTTGATCGTGGCAAGTCTTGCGCTGATTTTACTGCCTGTGGTGGCGTTCAGC
AGTCATTATGCCAGTTTCTTTCGCGTGCATAAGCCGCTGCGTAGCTATGTCAATCCGATCATGCCAATCTACTCGGTGGG
TAAGCTTGCCAGTATTGAGTATAAAAAAGCCAGTGCGCCAAAAGATACCATTTATCACGCCAAAGACGCGGTACAAGCAA
CCAAGCCTGATATGCGTAAGCCACGCCTAGTGGTGTTCGTCGTCGGTGAGACGGCACGCGCCGATCATGTCAGCTTCAAT
GGCTATGAGCGCGATACTTTCCCACAGCTTGCCAAGATCGATGGCGTGACCAATTTTAGCAATGTCACATCGTG

>MN148431.1 Escherichia coli strain RShimy3 MCR family phosphoethanolamine--lipid A transferase (mcr) gene, partial cds

CAGCTATCTGGGCGCGGATGAGTATGATGTCGATACCGACAAATACCAAGAAAATGTGCTGGATACGCTGGATCGCTTGG
GCGTAAGTATCTTGTGGCGTGATAATAATTCGGACTCAAAAGGCGTGATGGATAAGCTGCCAAAAGCGCAATTTGCCGAT
TATAAATCCGCGACCAACAACGCCATCTGCAACACCAATCCTTATAACGAATGCCGCGATGTCGGTATGCTCGTTGGCTT
AGATGACTTTGTCGCTGCCAATAACGGCAAAGATATGCTGATCATGCTGCACCAAATGGGCAATCACGGGCCTGCGTATT
TTAAGCGATATGATGAAAAGTTTGCCAAATTCACGCCAGTGTGTGAAGGTAATGAGCTTGCCAAGTGCGAACATCAGTCC

TTGATCAATGCTTATGACAATGCCTTGCTTGCCACCGATGATTTCATCGCTCAAAGTATCCAGTGGCTGCAGACGCACAG

CAATGCCTATGATGTCTCAATGCTGTATGTCAGCGATCATGGCGAAAGTCTGGGTGAGAACGGTGTCTATCTACATGGTA

TGCCAAATGCCTTTGCACCAAAAGAACAGCGCAGTGTGCCTGCATTTTTCTGGACGGATAAGCAAACTGGCATCACGCCA

ATGGCAACCGATACCGTCCTGACCCATGACGCGATCACGCCGACATTA

>NG_051171.1 Escherichia coli KP37 pKP37-BE mcr-2 gene for phosphoethanolamine--lipid A transferase MCR-2.1, complete CDS

ACAGCCCCTTTATTTATCTGCACAATATATCTAGATTCTACAACGACATTGAAGTATAATCGCCAACTTGTATCGTATAT

GGCATTTGTGGGTAATTTCTATGACATCACATCACTCTTGGTATCGCTATTCTATCAATCCTTTTGTGCTGATGGGTTTG

GTGGCGTTATTTTTGGCAGCGACAGCGAACCTGACATTTTTTGAAAAAGCGATGGCGGTCTATCCTGTATCGGATAACTT

AGGCTTTATCATCTCAATGGCGGTGGCGGTGATGGGTGCTATGCTACTGATTGTCGTGCTGTTATCCTATCGCTATGTGC

TAAAGCCTGTCCTGATTTTGCTACTGATTATGGGTGCGGTGACGAGCTATTTTACCGATACTTATGGCACGGTCTATGAC

ACCACCATGCTCCAAAATGCCATGCAAACCGACCAAGCCGAGTCTAAGGACTTGATGAATTTGGCGTTTTTTGTGCGAAT

TATCGGGCTTGGCGTGTTGCCAAGTGTGTTGGTCGCAGTTGCCAAAGTCAATTATCCAACATGGGGCAAAGGTCTGATTC

AGCGTGCGATGACATGGGGTGTCAGCCTTGTGCTGTTGCTTGTGCCGATTGGACTATTTAGCAGTCAGTATGCGAGTTTC

TTTCGGGTGCATAAGCCAGTGCGTTTTTATATCAACCCGATTACGCCGATTTATTCGGTGGGTAAGCTTGCCAGTATCGA

GTACAAAAAAGCCACTGCGCCAACAGACACCATCTATCATGCCAAAGACGCCGTGCAGACCACCAAGCCGAGCGAGCGTA

AGCCACGCCTAGTGGTGTTCGTCGTCGGTGAGACGGCGCGTGCTGACCATGTGCAGTTCAATGGCTATGGCCGTGAGACT

TTCCCGCAGCTTGCCAAAGTTGATGGCTTGGCGAATTTTAGCCAAGTGACATCGTGTGGCACATCGACGGCGTATTCTGT

GCCGTGTATGTTCAGCTATTTGGGTCAAGATGACTATGATGTCGATACCGCCAAATACCAAGAAAATGTGCTAGATACGC

TTGACCGCTTGGGTGTGGGTATCTTGTGGCGTGATAATAATTCAGACTCAAAAGGCGTGATGGATAAGCTACCTGCCACG

CAGTATTTTGATTATAAATCAGCAACCAACAATACCATCTGTAACACCAATCCCTATAACGAATGCCGTGATGTCGGTAT

GCTTGTCGGGCTAGATGACTATGTCAGCGCCAATAATGGCAAAGATATGCTCATCATGCTACACCAAATGGGCAATCATG

GGCCGGCGTACTTTAAGCGTTATGATGAGCAATTTGCCAAATTCACCCCCGTGTGCGAAGGCAACGAGCTTGCCAAATGC

GAACACCAATCACTCATCAATGCCTATGACAATGCGCTACTTGCGACTGATGATTTTATCGCCAAAAGCATCGATTGGCT

AAAAACGCATGAAGCGAACTACGATGTCGCCATGCTCTATGTCAGTGACCACGGCGAGAGCTTGGGCGAAAATGGTGTCT

ATCTGCATGGTATGCCAAATGCCTTTGCACCAAAAGAACAGCGAGCTGTGCCTGCGTTTTTTTGGTCAAATAATACGACA

TTCAAGCCAACTGCCAGCGATACTGTGCTGACGCATGATGCGATTACGCCAACACTGCTTAAGCTGTTTGATGTCACAGC

GGGCAAGGTCAAAGACCGCGCGGCATTTATCCAGTAAGTTATCATCATAACCCAATCTCAAGCCATCACACTACGGTATC

GTGTGATGGCGCAGGGCGGATTTTGCAATCTATTTTGCAATGTTTTTTTAGGAATGT

>NG_065452.1 Escherichia coli ECCTRSRTH05 mcr-2 gene for phosphoethanolamine--lipid A transferase MCR-2.3, complete CDS

TCAAAGTGATCTACTATTCCGAACAGTTATTTAGATTCTACAACGACATTGAAGTATAATCGCCAACTTGTATCGTCTAT

GGCAATTGCGGGTAACATCTATGACATCACATCACTCTTGGTATCGCTATTCTATCAATCCCTTTGTGCTGATGGGTTTG

GTGGCGTTATTTTTGGCAGCGACAGCGAACCTGACATTTTTTGAAAAAGCGATGGCGGTCTATCCTGTATCGGATAACTT

AGGCTTTATCATCTCAATGGCGGTGGCGGTGATGGGTGCTATGCTATTGATTGTTGTGCTATTATCCTATCGCTATGTGC

TAAAGCCTGTGCTGATTTTGCTGCTTATCATGGGTGCGGTGACGAGCTATTTTACCGATACTTATGGCACGGTCTATGAC

ACCACCATGCTCCAAAATGCCATGCAAACCGACCAAGCCGAGTCTAAAGACTTGATGAATTTGGCGTTTTTTGTGCGGAT

TATCGGGCTTGGCGTGTTGCCAAGTCTATTGGTCGCAGTTGCCAAAGTGGGTTATCCAACATGGGGCAAAAGCCTGATTC

AGCGTGCGATGACGTGGGGTGTCAGCCTTGTGTTGTTGCTTGTGCCGATTGGGTTATTTAGCAGTCAGTATGCGAGTTTC

TTTCGGGTGCATAAGCCAGTGCGTTTTTATATCAACCCGATTACGCCGATTTATTCGGTGGGTAAGCTTGCCAGTATCGA

GTACAAAAAAGCCACTGCACCAACAGACACCATCTATCATGCCAAAGATGCCGTGCAGACCACCAAGCCTAGCGAGCGTA

AGCCCCGTCTAGTGGTGTTCGTCGTCGGTGAGACGGCGCGTGCTGACCATGTGCAGTTCAATGGCTATAGCCGTGAGACT

TTCCCGCAGCTTGCCAAAGTTGATGGCTTGGCGAACTTTAGCCAAGTGACATCGTGTGGCACATCGACGGCGTATTCTGT

GCCGTGTATGTTCAGCTATTTGGGTCAAGATGACTATGATGTCGATACCGCCAAATACCAAGAAAATGTGCTAGATACGC

TTGACCGCTTGGGCGTGGGTATCTTGTGGCGTGATAATAATTCAGACTCAAAAGGCGTGATGGATAAGCTACCTGCTACG

CAGTATTTTGATTATAAATCAGCGACCAACAACACCATCTGTAACACCAACCCTTATAATGAATGTCGTGATGTCGGTAT

GCTTGTTGGGCTTGATGACTATGTCAGCGCCAATAATGGCAAAGATATGCTCATCATGCTACACCAAATGGGCAATCATG

GGCCGGCGTACTTTAAGCGTTATGATGAGCAATTTACCAAATTCACCCCTGTGTGCGAAGGCAACGAGCTTGCCAAATGC

GAACATCAATCGCTCATCAATGCCTATGATAATGCACTGCTTGCCACCGATGATTTTATCGCCAAAAGTATCGATTGGCT

AAAAACACATGAAGCAAACTACGATGTCGCCATGCTCTATGTCAGCGACCACGGCGAGAGCTTGGGCGAAAATGGTGTCT

ATCTGCATGGTATGCCAAATGCCTTTGCACCAAAAGAACAACGAGCCGTACCTGTGTTTTTTTGGTCAAATAATACGACA

TTCAAGCCAACTGCCAGCGATACTGCATTGACCCATGATGCGATTACGCCGACATTGCTTAAGCTGTTTGATGTCACAGC

TGATAAGGTCAAAGACCGCACGGCATTTATCCAGTAAGTTATCAGCATAACCCAATACCGAGCCATCACACTGCATTATC

GTGTGATGGCAAAGGGTAGATTTGCAACATTTTTTTTGGGCGTAATAGACATTTTTC

>KU323969.1 Escherichia coli strain 235 phosphoethanolamine transferase (mcr-1) gene, partial cds

TGCAGCATACTTCTGTGTGGTACCGACGCTCGGTCAGTCCGTTTGTTCTTGTGGCGAGTGTTGCCGTTTTCTTGACCGCG

ACCGCCAATCTTACCTTTTTTGATAAAATCAGCCAAACCTATCCCATCGCGGACAATCTCGGCTTTGTGCTGACGATCGC

TGTCGTGCTCTTTGGCGCGATGCTACTGATCACCACGCTGTTATCATCGTATCGCTATGTGCTAAAGCCTGTGTTGATTT

TGCTATTAATCATGGGCGCGGTGACCAGTTATTTTACTGACACTTATGGCACGGTCTATGATACGACCATGCTCCAAAAT

GCCCTACAGACCGACCAAGCCGAGACCAAGGATCTATTAAACGCAGCGTTTATCATGCGTATCATTGGTTTGGGTGTGCT

ACCAAGTTTGCTTGTGGCTTTTGTTAAGGTGGATTATCCGACTTGGGGCAAGGGTTTGATGCGCCGATTGGGCTTGATCG

TGGCAAGTCTTGCGCTGATTTTACTGCCTGTGGTGGCGTTCAGCAGTCATTATGCCAGTTTCTTTCGCGTGCATA
