# Supplementary material for: Molecular Detection of Colistin Resistance mcr-1 Gene in Multidrug-Resistant Escherichia coli Isolated from Chicken
Source: Antibiotics (Basel). 2022 Jan 13;11(1):97. doi: 10.3390/antibiotics11010097 (PMC8772701; doi:10.3390/antibiotics11010097)
Supplement: Supplementary file 1 [file antibiotics-11-00097-s001.zip › Supplementary File S3.pdf]

Supplementary File S3: A total of 52 MCR-1 proteins retrieved from NCBI database except *E. coli*.

>Aeromonas_dhakensis_MCR-1.1_1

ATGATGCAGCATACTTCTGTGTGGTACCGACGCTCGGTCAGTCCGTTTGTTCTTGTGGCGAGTGTTGCCGTTTTCTTGAC

CGCGACCGCCAATCTTACCTTTTTTGATAAAATCAGCCAAACCTATCCCATCGCGGACAATCTCGGCTTTGTGCTGACGA

TCGCTGTCGTGCTCTTTGGCGCGATGCTACTGATCACCACGCTGTTATCATCGTATCGCTATGTGCTAAAGCCTGTGTTG

ATTTTGCTATTAATCATGGGCGCGGTGACCAGTTATTTTACTGACACTTATGGCACGGTCTATGATACGACCATGCTCCA

AAATGCCCTACAGACCGACCAAGCCGAGACCAAGGATCTATTAAACGCAGCGTTTATCATGCGTATCATTGGTTTGGGTG

TGCTACCAAGTTTGCTTGTGGCTTTTGTTAAGGTGGATTATCCGACTTGGGGCAAGGGTTTGATGCGCCGATTGGGCTTG

ATCGTGGCAAGTCTTGCGCTGATTTTACTGCCTGTGGTGGCGTTCAGCAGTCATTATGCCAGTTTCTTTCGCGTGCATAA

GCCGCTGCGTAGCTATGTCAATCCGATCATGCCAATCTACTCGGTGGGTAAGCTTGCCAGTATTGAGTATAAAAAAGCCA

GTGCGCCAAAAGATACCATTTATCACGCCAAAGACGCGGTACAAGCAACCAAGCCTGATATGCGTAAGCCACGCCTAGTG

GTGTTCGTCGTCGGTGAGACGGCACGCGCCGATCATGTCAGCTTCAATGGCTATGAGCGCGATACTTTCCCACAGCTTGC

CAAGATCGATGGCGTGACCAATTTTAGCAATGTCACATCGTGCGGCACATCGACGGCGTATTCTGTGCCGTGTATGTTCA

GCTATCTGGGCGCGGATGAGTATGATGTCGATACCGCCAAATACCAAGAAAATGTGCTGGATACGCTGGATCGCTTGGGC

GTAAGTATCTTGTGGCGTGATAATAATTCGGACTCAAAAGGCGTGATGGATAAGCTGCCAAAAGCGCAATTTGCCGATTA

TAAATCCGCGACCAACAACGCCATCTGCAACACCAATCCTTATAACGAATGCCGCGATGTCGGTATGCTCGTTGGCTTAG

ATGACTTTGTCGCTGCCAATAACGGCAAAGATATGCTGATCATGCTGCACCAAATGGGCAATCACGGGCCTGCGTATTTT

AAGCGATATGATGAAAAGTTTGCCAAATTCACGCCAGTGTGTGAAGGTAATGAGCTTGCCAAGTGCGAACATCAGTCCTT

GATCAATGCTTATGACAATGCCTTGCTTGCCACCGATGATTTCATCGCTCAAAGTATCCAGTGGCTGCAGACGCACAGCA

ATGCCTATGATGTCTCAATGCTGTATGTCAGCGATCATGGCGAAAGTCTGGGTGAGAACGGTGTCTATCTACATGGTATG

CCAAATGCCTTTGCACCAAAAGAACAGCGCAGTGTGCCTGCATTTTTCTGGACGGATAAGCAAACTGGCATCACGCCAAT

GGCAACCGATACCGTCCTGACCCATGACGCGATCACGCCGACATTATTAAAGCTGTTTGATGTCACCGCGGACAAAGTCA

AAGACCGCACCGCATTCATCCGCTGA

>Aeromonas_veronii_MCR-1.1_2

ATGATGCAGCATACTTCTGTGTGGTACCGACGCTCGGTCAGTCCGTTTGTTCTTGTGGCGAGTGTTGCCGTTTTCTTGAC

CGCGACCGCCAATCTTACCTTTTTTGATAAAATCAGCCAAACCTATCCCATCGCGGACAATCTCGGCTTTGTGCTGACGA

TCGCTGTCGTGCTCTTTGGCGCGATGCTACTGATCACCACGCTGTTATCATCGTATCGCTATGTGCTAAAGCCTGTGTTG

ATTTTGCTATTAATCATGGGCGCGGTGACCAGTTATTTTACTGACACTTATGGCACGGTCTATGATACGACCATGCTCCA

AAATGCCCTACAGACCGACCAAGCCGAGACCAAGGATCTATTAAACGCAGCGTTTATCATGCGTATCATTGGTTTGGGTG

TGCTACCAAGTTTGCTTGTGGCTTTTGTTAAGGTGGATTATCCGACTTGGGGCAAGGGTTTGATGCGCCGATTGGGCTTG

ATCGTGGCAAGTCTTGCGCTGATTTTACTGCCTGTGGTGGCGTTCAGCAGTCATTATGCCAGTTTCTTTCGCGTGCATAA

GCCGCTGCGTAGCTATGTCAATCCGATCATGCCAATCTACTCGGTGGGTAAGCTTGCCAGTATTGAGTATAAAAAAGCCA

GTGCGCCAAAAGATACCATTTATCACGCCAAAGACGCGGTACAAGCAACCAAGCCTGATATGCGTAAGCCACGCCTAGTG

GTGTTCGTCGTCGGTGAGACGGCACGCGCCGATCATGTCAGCTTCAATGGCTATGAGCGCGATACTTTCCCACAGCTTGC

CAAGATCGATGGCGTGACCAATTTTAGCAATGTCACATCGTGCGGCACATCGACGGCGTATTCTGTGCCGTGTATGTTCA

GCTATCTGGGCGCGGATGAGTATGATGTCGATACCGCCAAATACCAAGAAAATGTGCTGGATACGCTGGATCGCTTGGGC

GTAAGTATCTTGTGGCGTGATAATAATTCGGACTCAAAAGGCGTGATGGATAAGCTGCCAAAAGCGCAATTTGCCGATTA

TAAATCCGCGACCAACAACGCCATCTGCAACACCAATCCTTATAACGAATGCCGCGATGTCGGTATGCTCGTTGGCTTAG

ATGACTTTGTCGCTGCCAATAACGGCAAAGATATGCTGATCATGCTGCACCAAATGGGCAATCACGGGCCTGCGTATTTT

AAGCGATATGATGAAAAGTTTGCCAAATTCACGCCAGTGTGTGAAGGTAATGAGCTTGCCAAGTGCGAACATCAGTCCTT

GATCAATGCTTATGACAATGCCTTGCTTGCCACCGATGATTTCATCGCTCAAAGTATCCAGTGGCTGCAGACGCACAGCA

ATGCCTATGATGTCTCAATGCTGTATGTCAGCGATCATGGCGAAAGTCTGGGTGAGAACGGTGTCTATCTACATGGTATG

CCAAATGCCTTTGCACCAAAAGAACAGCGCAGTGTGCCTGCATTTTTCTGGACGGATAAGCAAACTGGCATCACGCCAAT

GGCAACCGATACCGTCCTGACCCATGACGCGATCACGCCGACATTATTAAAGCTGTTTGATGTCACCGCGGACAAAGTCA

AAGACCGCACCGCATTCATCCGCTGA

>Salmonella_enterica_MCR-1_3

CAAAATTGCAGTATGATTGCCGCAATTATCCCACCGTTTATTTTTTGAGTAGTTTCTCATGATGCAGCATACTTCTGTGT

GGTACCGACGCTCGGTCAGTCCGTTTGTTCTTGTGGCGAGTGTTGCCGTTTTCTTGACCGCGACCGCCAATCTTACCTTT

TTTGATAAAATCAGCCAAACCTATCCCATCGCGGACAATCTCGGCTTTGTGCTGACGATCGCTGTCGTGCTCTTTGGCGC

GATGCTACTGATCACCACGCTGTTATCATCGTATCGCTATGTGCTAAAGCCTGTGTTGATTTTGCTATTAATCATGGGCG

CGGTGACCAGTTATTTTACTGACACTTATGGCACGGTCTATGATACGACCATGCTCCAAAATGCCCTACAGACCGACCAA

GCCGAGACCAAGGATCTATTAAACGCAGCGTTTATCATGCGTATCATTGGTTTGGGTGTGCTACCAAGTTTGCTTGTGGC

TTTTGTTAAGGTGGATTATCCGACTTGGGGCAAGGGTTTGATGCGCCGATTGGGCTTGATCGTGGCAAGTCTTGCGCTGA

TTTTACTGCCTGTGGTGGCGTTCAGCAGTCATTATGCCAGTTTCTTTCGCGTGCATAAGCCGCTGCGTAGCTATGTCAAT

CCGATCATGCCAATCTACTCGGTGGGTAAGCTTGCCAGTATTGAGTATAAAAAAGCCAGTGCGCCAAAAGATACCATTTA

TCACGCCAAAGACGCGGTACAAGCAACCAAGCCTGATATGCGTAAGCCACGCCTAGTGGTGTTCGTCGTCGGTGAGACGG

CACGCGCCGATCATGTCAGCTTCAATGGCTATGAGCGCGATACTTTCCCACAGCTTGCCAAGATCGATGGCGTGACCAAT

TTTAGCAATGTCACATCGTGCGGCACATCGACGGCGTATTCTGTGCCGTGTATGTTCAGCTATCTGGGCGCGGATGAGTA

TGATGTCGATACCGCCAAATACCAAGAAAATGTGCTGGATACGCTGGATCGCTTGGGCGTAAGTATCTTGTGGCGTGATA

ATAATTCGGACTCAAAAGGCGTGATGGATAAGCTGCCAAAAGCGCAATTTGCCGATTATAAATCCGCGACCAACAACGCC

ATCTGCAACACCAATCCTTATAACGAATGCCGCGATGTCGGTATGCTCGTTGGCTTAGATGACTTTGTCGCTGCCAATAA

CGGCAAAGATATGCTGATCATGCTGCACCAAATGGGCAATCACGGGCCTGCGTATTTTAAGCGATATGATGAAAAGTTTG

CCAAATTCACGCCAGTGTGTGAAGGTAATGAGCTTGCCAAGTGCGAACATCAGTCCTTGATCAATGCTTATGACAATGCC

TTGCTTGCCACCGATGATTTCATCGCTCAAAGTATCCAGTGGCTGCAGACGCACAGCAATGCCTATGATGTCTCAATGCT

GTATGTCAGCGATCATGGCGAAAGTCTGGGTGAGAACGGTGTCTATCTACATGGTATGCCAAATGCCTTTGCACCAAAAG

AACAGCGCAGTGTGCCTGCATTTTTCTGGACGGATAAGCAAACTGGCATCACGCCAATGGCAACCGATACCGTCCTGACC

CATGACGCGATCACGCCGACATTATTAAAGCTGTTTGATGTCACCGCGGACAAAGTCAAAGACCGCACCGCATTCATCCG

CTGATTTCTCCCTGTATTTTTTCCAAACCCACCGCACACTCCATTCGTATTATGGGCCGGTGGGGTGGAG

>Salmonella_enterica_MCR-1_4

AAAATTGCAGTATGATTGCCGCAATTATCCCACCGTTTATTTTTTGAGTAGTTTCTCATGATGCAGCATACTTCTGTGTG

GTACCGACGCTCGGTCAGTCCGTTTGTTCTTGTGGCGAGTGTTGCCGTTTTCTTGACCGCGACCGCCAATCTTACCTTTT

TTGATAAAATCAGCCAAACCTATCCCATCGCGGACAATCTCGGCTTTGTGCTGACGATCGCTGTCGTGCTCTTTGGCGCG

ATGCTACTGATCACCACGCTGTTATCATCGTATCGCTATGTGCTAAAGCCTGTGTTGATTTTGCTATTAATCATGGGCGC

GGTGACCAGTTATTTTACTGACACTTATGGCACGGTCTATGATACGACCATGCTCCAAAATGCCCTACAGACCGACCAAG

CCGAGACCAAGGATCTATTAAACGCAGCGTTTATCATGCGTATCATTGGTTTGGGTGTGCTACCAAGTTTGCTTGTGGCT

TTTGTTAAGGTGGATTATCCGACTTGGGGCAAGGGTTTGATGCGCCGATTGGGCTTGATCGTGGCAAGTCTTGCGCTGAT

TTTACTGCCTGTGGTGGCGTTCAGCAGTCATTATGCCAGTTTCTTTCGCGTGCATAAGCCGCTGCGTAGCTATGTCAATC

CGATCATGCCAATCTACTCGGTGGGTAAGCTTGCCAGTATTGAGTATAAAAAAGCCAGTGCGCCAAAAGATACCATTTAT

CACGCCAAAGACGCGGTACAAGCAACCAAGCCTGATATGCGTAAGCCACGCCTAGTGGTGTTCGTCGTCGGTGAGACGGC

ACGCGCCGATCATGTCAGCTTCAATGGCTATGAGCGCGATACTTTCCCACAGCTTGCCAAGATCGATGGCGTGACCAATT

TTAGCAATGTCACATCGTGCGGCACATCGACGGCGTATTCTGTGCCGTGTATGTTCAGCTATCTGGGCGCGGATGAGTAT

GATGTCGATACCGCCAAATACCAAGAAAATGTGCTGGATACGCTGGATCGCTTGGGCGTAAGTATCTTGTGGCGTGATAA

TAATTCGGACTCAAAAGGCGTGATGGATAAGCTGCCAAAAGCGCAATTTGCCGATTATAAATCCGCGACCAACAACGCCA

TCTGCAACACCAATCCTTATAACGAATGCCGCGATGTCGGTATGCTCGTTGGCTTAGATGACTTTGTCGCTGCCAATAAC

GGCAAAGATATGCTGATCATGCTGCACCAAATGGGCAATCACGGGCCTGCGTATTTTAAGCGATATGATGAAAAGTTTGC

CAAATTCACGCCAGTGTGTGAAGGTAATGAGCTTGCCAAGTGCGAACATCAGTCCTTGATCAATGCTTATGACAATGCCT

TGCTTGCCACCGATGATTTCATCGCTCAAAGTATCCAGTGGCTGCAGACGCACAGCAATGCCTATGATGTCTCAATGCTG

TATGTCAGCGATCATGGCGAAAGTCTGGGTGAGAACGGTGTCTATCTACATGGTATGCCAAATGCCTTTGCACCAAAAGA

ACAGCGCAGTGTGCCTGCATTTTTCTGGACGGATAAGCAAACTGGCATCACGCCAATGGCAACCGATACCGTCCTGACCC

ATGACGCGATCACGCCGACATTATTAAAGCTGTTTGATGTCACCGCGGACAAAGTCAAAGACCGCACCGCATTCATCCGC

TGATTTCTCCCTGTATTTTTTCCAAACCCACCGCACACTCCATTCGTATTATGGGCGGTGGGCTGGGC

>Klebsiella_pneumoniae_mcr1_5

TTTCTGTATCGCATAATTTTTTATATCAGATAAATTGTACTGGATTTCTTAAAAAATTGCAGTATAATTGCCGCAATTAT

CCCACCGTTTATTTTTTGAGTAGTTTCTCATGATGCAGCATACTTCTGTGTGGTATCGACGCTCGGTCAGTCCGTTTGTT

CTTGTGGCGAGTGTTGCCGTTTTCTTGACCGCGACCGCCAATCTTACCTTTTTTGATAAAATCAGCCAAACCTATCCCAT

CGCGGACAATCTCGGCTTTGTGCTGACGATCGCTGTCGTGCTCTTTGGCGCGATGCTACTGATCACCACGCTGTTATCAT

CGTATCGCTATGTGCTAAAGCCTGTGTTGATTTTGCTATTAATCATGGGCGCGGTGACCAGTTATTTTACTGACACTTAT

GGCACGGTCTATGATACGACCATGCTCCAAAATGCCCTACAGACCGACCAAGCCGAGACCAAGGATCTATTAAACGCAGC

GTTTATCATGCGTATCATTGGTTTGGGTGTGCTACCAAGTTTGCTTGTGGCTTTTGTTAAGGTGGATTATCCGACTTGGG

GCAAGGGTTTGATGCGCCGATTGGGCTTGATCGTGGCAAGTCTTGCGCTGATTTTACTGCCTGTGGTGGCGTTCAGCAGT

CATTATGCCAGTTTCTTTCGCGTGCATAAGCCGCTGCGTAGCTATGTCAATCCGATCATGCCAATCTACTCGGTGGGTAA

GCTTGCCAGTATTGAGTATAAAAAAGCCAGTGCGCCAAAAGATACCATTTATCACGCCAAAGACGCGGTACAAGCAACCA

AGCCTGATATGCGTAAGCCACGCCTAGTGGTGTTCGTCGTCGGTGAGACGGCACGCGCCGATCATGTCAGCTTCAATGGC

TATGAGCGCGATACTTTCCCACAGCTTGCCAAGATCGATGGCGTGACCAATTTTAGCAATGTCACATCGTGCGGCACATC

GACGGCGTATTCTGTGCCGTGTATGTTCAGCTATCTGGGCGCGGATGAGTATGATGTCGATACCGCCAAATACCAAGAAA

ATGTGCTGGATACGCTGGATCGCTTGGGCGTAAGTATCTTGTGGCGTGATAATAATTCGGACTCAAAAGGCGTGATGGAT

AAGCTGCCAAAAGCGCAATTTGCCGATTATAAATCCGCGACCAACAACGCCATCTGCAACACCAATCCTTATAACGAATG

CCGCGATGTCGGTATGCTCGTTGGCTTAGATGACTTTGTCGCTGCCAATAACGGCAAAGATATGCTGATCATGCTGCACC

AAATGGGCAATCACGGGCCTGCGTATTTTAAGCGATATGATGAAAAGTTTGCCAAATTCACGCCAGTGTGTGAAGGTAAT

GAGCTTGCCAAGTGCGAACATCAGTCCTTGATCAATGCTTATGACAATGCCTTGCTTGCCACCGATGATTTCATCGCTCA

AAGTATCCAGTGGCTGCAGACGCACAGCAATGCCTATGATGTCTCAATGCTGTATGTCAGCGATCATGGCGAAAGTCTGG

GTGAGAACGGTGTCTATCTACATGGTATGCCAAATGCCTTTGCACCAAAAGAACAGCGCAGTGTGCCTGCATTTTTCTGG

ACGGATAAGCAAACTGGCATCACGCCAATGGCAACCGATACCGTCCTGACCCATGACGCGATCACGCCGACATTATTAAA

GCTGTTTGATGTCACCGCGGACAAAGTCAAAGACCGCACCGCATTCATCCGCTGATTTCTCCCTGTATTTTTCCAAACC

CACCGCACACTCCATTCGTATTATGGGCGGTGGGGTGGGGTTTGTTATGCCGTATTTATCAAATAAACGCCTAC

>Klebsiella_pneumoniae_MCR-1.2_6

CGCATAATTTTTTATATCAGATAAATTGTACTGGATTTCTTAAAAAATTGCAGTATAATTGCCGCAATTATCCCACCGTT

TATTTTTTGAGTAGTTTCTCATGATGCTGCATACTTCTGTGTGGTACCGACGCTCGGTCAGTCCGTTTGTTCTTGTGGCG

AGTGTTGCCGTTTTCTTGACCGCGACCGCCAATCTTACCTTTTTTGATAAAATCAGCCAAACCTATCCCATCGCGGACAA

TCTCGGCTTTGTGCTGACGATCGCTGTCGTGCTCTTTGGCGCGATGCTACTGATCACCACGCTGTTATCATCGTATCGCT

ATGTGCTAAAGCCTGTGTTGATTTTGCTATTAATCATGGGCGCGGTGACCAGTTATTTTACTGACACTTATGGCACGGTC

TATGATACGACCATGCTCCAAAATGCCCTACAGACCGACCAAGCCGAGACCAAGGATCTATTAAACGCAGCGTTTATCAT

GCGTATCATTGGTTTGGGTGTGCTACCAAGTTTGCTTGTGGCTTTTGTTAAGGTGGATTATCCGACTTGGGGCAAGGGTT

TGATGCGCCGATTGGGCTTGATCGTGGCAAGTCTTGCGCTGATTTTACTGCCTGTGGTGGCGTTCAGCAGTCATTATGCC

AGTTTCTTTCGCGTGCATAAGCCGCTGCGTAGCTATGTCAATCCGATCATGCCAATCTACTCGGTGGGTAAGCTTGCCAG

TATTGAGTATAAAAAAGCCAGTGCGCCAAAAGATACCATTTATCACGCCAAAGACGCGGTACAAGCAACCAAGCCTGATA

TGCGTAAGCCACGCCTAGTGGTGTTCGTCGTCGGTGAGACGGCACGCGCCGATCATGTCAGCTTCAATGGCTATGAGCGC

GATACTTTCCCACAGCTTGCCAAGATCGATGGCGTGACCAATTTTAGCAATGTCACATCGTGCGGCACATCGACGGCGTA
TTCTGTGCCGTGTATGTTCAGCTATCTGGGCGCGGATGAGTATGATGTCGATACCGCCAAATACCAAGAAAATGTGCTGG
ATACGCTGGATCGCTTGGGCGTAAGTATCTTGTGGCGTGATAATAATTCGGACTCAAAAGGCGTGATGGATAAGCTGCCA
AAAGCGCAATTTGCCGATTATAAATCCGCGACCAACAACGCCATCTGCAACACCAATCCTTATAACGAATGCCGCGATGT
CGGTATGCTCGTTGGCTTAGATGACTTTGTCGCTGCCAATAACGGCAAAGATATGCTGATCATGCTGCACCAAATGGGCA
ATCACGGGCCTGCGTATTTTAAGCGATATGATGAAAAGTTTGCCAAATTCACGCCAGTGTGTGAAGGTAATGAGCTTGCC
AAGTGCGAACATCAGTCCTTGATCAATGCTTATGACAATGCCTTGCTTGCCACCGATGATTTCATCGCTCAAAGTATCCA
GTGGCTGCAGACGCACAGCAATGCCTATGATGTCTCAATGCTGTATGTCAGCGATCATGGCGAAAGTCTGGGTGAGAACG
GTGTCTATCTACATGGTATGCCAAATGCCTTTGCACCAAAAGAACAGCGCAGTGTGCCTGCATTTTTCTGGACGGATAAG
CAAACTGGCATCACGCCAATGGCAACCGATACCGTCCTGACCCATGACGCGATCACGCCGACATTATTAAAGCTGTTTGA
TGTCACCGCGGACAAAGTCAAAGACCGCACCGCATTCATCCGCTGATTTCTCCCTGTATTTTTCCAAACCCACCGCACA
CTCCATTCGTATTATGGGCGGTGGGGTGGGGTTTGTTATGCCGTATTTATCAAATAAACGCCTACT

>Uncultured_bacterium_MCR-1.1_7

ATGATGCAGCATACTTCTGTGTGGTACCGACGCTCGGTCAGTCCGTTTGTTCTTGTGGCGAGTGTTGCCGTTTTCTTGAC
CGCGACCGCCAATCTTACCTTTTTTGATAAAATCAGCCAAACCTATCCCATCGCGGACAATCTCGGCTTTGTGCTGACGA
TCGCTGTCGTGCTCTTTGGCGCGATGCTACTGATCACCACGCTGTTATCATCGTATCGCTATGTGCTAAAGCCTGTGTTG
ATTTTGCTATTAATCATGGGCGCGGTGACCAGTTATTTTACTGACACTTATGGCACGGTCTATGATACGACCATGCTCCA
AAATGCCCTACAGACCGACCAAGCCGAGACCAAGGATCTATTAAACGCAGCGTTTATCATGCGTATCATTGGTTTGGGTG

TGCTACCAAGTTTGCTTGTGGCTTTTGTTAAGGTGGATTATCCGACTTGGGGCAAGGGTTTGATGCGCCGATTGGGCTTG

ATCGTGGCAAGTCTTGCGCTGATTTTACTGCCTGTGGTGGCGTTCAGCAGTCATTATGCCAGTTTCTTTCGCGTGCATAA

GCCGCTGCGTAGCTATGTCAATCCGATCATGCCAATCTACTCGGTGGGTAAGCTTGCCAGTATTGAGTATAAAAAAGCCA

GTGCGCCAAAAGATACCATTTATCACGCCAAAGACGCGGTACAAGCAACCAAGCCTGATATGCGTAAGCCACGCCTAGTG

GTGTTCGTCGTCGGTGAGACGGCACGCGCCGATCATGTCAGCTTCAATGGCTATGAGCGCGATACTTTCCCACAGCTTGC

CAAGATCGATGGCGTAACCAATTTTAGCAATGTCACATCGTGCGGCACATCGACGGCGTATTCTGTGCCGTGTATGTTCA

GCTATCTGGGCGCGGATGAGTATGATGTCGATACCGCCAAATACCAAGAAAATGTGCTGGATACGCTGGATCGCTTGGGC

GTAAGTATCTTGTGGCGTGATAATAATTCGGACTCAAAAGGCGTGATGGATAAGCTGCCAAAAGCGCAATTTGCCGATTA

TAAATCCGCGACCAACAACGCCATCTGCAACACCAATCCTTATAACGAATGCCGCGATGTCGGTATGCTCGTTGGCTTAG

ATGACTTTGTCGCTGCCAATAACGGCAAAGATATGCTGATCATGCTGCACCAAATGGGCAATCACGGGCCTGCGTATTTT

AAGCGATATGATGAAAAGTTTGCCAAATTCACGCCAGTGTGTGAAGGTAATGAGCTTGCCAAGTGCGAACATCAGTCCTT

GATCAATGCTTATGACAATGCCTTGCTTGCCACCGATGATTTCATCGCTCAAAGTATCCAGTGGCTGCAGACGCACAGCA

ATGCCTATGATGTCTCAATGCTGTATGTCAGCGATCATGGCGAAAGTCTGGGTGAGAACGGTGTCTATCTACATGGTATG

CCAAATGCCTTTGCACCAAAAGAACAGCGCAGTGTGCCTGCATTTTTCTGGACGGATAAGCAAACTGGCATCACGCCAAT

GGCAACCGATACCGTCCTGACCCATGACGCGATCACGCCGACATTATTAAAGCTGTTTGATGTCACCGCGGACAAAGTCA

AAGACCGCACCGCATTCATCCGCTGA

>Uncultured_bacterium_MCR-1.30_8

ATGATGCAGCATACTTCTGTGTGGTACCGACGCTCGGTCAGTCCGTTTGTTCTTGTGGCGAGTGTTGCCGTTTTCTTGAC

CGCGACCGCCAATCTTACCTTTTTTGATAAAATCAGCCAAACCTATCCCATCGCGGACAATCTCGGCTTTGTGCTGACGA

TCGCTGTCGTGCTCTTTGGCGCGATGCTACTGATCACCACGCTGTTATCATCGTATCGCTATGTGCTAAAGCCTGTGTTG

ATTTTGCTATTAATCATGGGCGCGGTGACCAGTTATTTTACTGACACTTATGGCACGGTCTATGATACGACCATGCTCCA

AAATGCCCTACAGACCGACCAAGCCGAGACCAAGGATCTATTAAACGCAGCGTTTATCATGCGTATCATTGGTTTGGGTG

TGCTACCAAGTTTGCTTGTGGCTTTTGTTAAGGTGGATTATCCGACTTGGGGCAAGGGTTTGATGCGCCGATTGGGCTTG

ATCGTGGCAAGTCTTGCGCTGATTTTACTGCCTGTGGTGGCGTTCAGCAGTCATTATGCCAGTTTCTTTCGCGTGCATAA

GCCGCTGCGTAGCTATGTCAATCCGATCATGCCAATCTACTCGGTGGGTAAGCTTGCCAGTATTGAGTATAAAAAAGCCA

GTGCGCCAAAAGATACCATTTATCACGCCAAAGACGCGGTACAAGCAACCAAGCCTGATATGCGTAAGCCACGCCTAGTG

GTGTTCGTCGTCGGTGAGACGGCACGCGCCGATCATGTCAGCTTCAATGGCTATGAGCGCGATACTTTCCCACAGCTTGC

CAAGATCGATGGCGTGACCAATTTTAGCAATGTCACATCGTGCGGCACATCGACGGCGTATTCTGTGCCGTGTATGTTCA

GCTATCTGGGCGCGGATGAGTATGATGTCGATACCGCCAAATACCAAGAAAATGTGCTGGATACGCTGGATCGCTTGGGC

GTAAGTATCTTGTGGCGTGATAATAATTCGGACTCAAAAGGCGTGATGGATAAGCTGCCAAAAGCGCAATTTGCCGATTA

TAAATCCGCGACCAACAACGCCATCTGCAACACCAATCCTTATAACGAATGCCGCGATGTCGGTATGCTCGTTGGCTTAG

ATGACTTTGTCGCTGCCAATAACGGCAAAGATATGCTGATCATGCTGCACCAAATGGGCAATCACGGGCCTGCGTATTTT

AAGCGATATGATGAAAAGTTTGCCAAATTCACGCCAGTGTGTGAAGGTAATGAGCTTGCCAAGTGCGAACATCAGTCCTT

GATCAATGCTTATGACAATGCCTTGCTTGCCACCGATGATTTCATCGCTCAAAGTATCCAGTGGCTGCAGACGCACAGCA

ATGCCTATGATGTCTCAATGCTGTATGTCAGCGATCATGGCGAAAGTCTGGGTGAGAACGATGTCTATCTACATGGTATG

CCAAATGCCTTTGCACCAAAAGAACAGCGCAGTGTGCCTGCATTTTTCTGGACGGATAAGCAAACTGGCATCACGCCAAT

GGCAACCGATACCGTCCTGACCCATGACGCGATCACGCCGACATTATTAAAGCTGTTTGATGTCACCGCGGACAAAGTCA

AAGACCGCACCGCATTCATCCGCTGA

>Uncultured_bacterium_MCR-1.29_9

ATGATGCAGCATACTTCTGTGTGGTACCGACGCTCGGTCAGTCCGTTTGTTCTTGTGGCGAGTGTTGCCGTTTTCTTGAC
CGCGACCGCCAATCTTACCTTTTTTGATAAAATCAGCCAAACCTATCCCATCGCGGACAATCTCGGCTTTGTGCTGACGA
TCGCTGTCGTGCTCTTTGGCGCGATGCTACTGATCACCACGCTGTTATCATCGTATCGCTATGTGCTAAAGCCTGTGTTG
ATTTTGCTATTAATCATGGGCGCGGTGACCAGTTATTTTACTGACACTTATGGCACGGTCTATGATACGACCATGCTCCA
AAATGCCCTACAGACCGACCAAGCCGAGACCAAGGATCTATTAAACGCAGCGTTTATCATGCGTATCATTGGTTTGGGTG
TGCTACCAAGTTTGCTTGTGGCTTTTGTTAAGGTGGATTATCCGACTTGGGGCAAGGGTTTGATGCGCCGATTGGGCTTG
ATCGTGGCAAGTCTTGCGCTGATTTTACTGCCTGTGGTGGCGTTCAGCAGTCATTATGCCAGTTTCTTTCGCGTGCATAA
GCCGCTGCGTAGCTATGTCAATCCGATCATGCCAATCTACTCGGTGGGTAAGCTTGCCAGTATTGAGTATAAAAAAGCCA
GTGCGCCAAAAGATACCATTTATCACGCCAAAGACGCGGTACAAGCAACCAAGCCTGATATGCGTAAGCCACGCCTAGTG
GTGTTCGTCGTCGGTGAGACGGCACGCGCCGATCATGTCAGCTTCAATGGCTATGAGCGCGATACTTTCCCACAGCTTGC
CAAGATCGATGGCGTGACCAATTTTAGCAATGTCACATCGTGCGGCACATCGACGGCGTATTCTGTGCCGTGTATGTTCA
GCTATCTGGGCGCGGATGAGTATGATGTCGATACCGCCAAATACCAAGAAAATGTGCTGGATACGCTGGATCGCTTGGGC
GTAAGTATCTTGTGGCGTGATAATAATTCGGACTCAAAAGGCGTGATGGATAAGCTGCCAAAAGCGCAATTTGCCGATTA
TAAATCCGCGACCAACAACGCCATCTGCAACACCAATCCTTATAACGAATGCCGCGATGTCGGTATGCTCGTTGGCTTAG
ATGACTTTGTCGCTGCCAATAACGGCAAAGATATGCTGATCATGCTGCACCAAATGGGCAATCACGGGTCTGCGTATTTT
AAGCGATATGATGAAAAGTTTGCCAAATTCACGCCAGTGTGTGAAGGTAATGAGCTTGCCAAGTGCGAACATCAGTCCTT

GATCAATGCTTATGACAATGCCTTGCTTGCCACCGATGATTTCATCGCTCAAAGTATCCAGTGGCTGCAGACGCACAGCA

ATGCCTATGATGTCTCAATGCTGTATGTCAGCGATCATGGCGAAAGTCTGGGTGAGAACGGTGTCTATCTACATGGTATG

CCAAATGCCTTTGCACCAAAAGAACAGCGCAGTGTGCCTGCATTTTTCTGGACGGATAAGCAAACTGGCATCACGCCAAT

GGCAACCGATACCGTCCTGACCCATGACGCGATCACGCCGACATTATTAAAGCTGTTTGATGTCACCGCGGACAAAGTCA

AAGACCGCACCGCATTCATCCGCTGA

>Klebsiella_pneumoniae_MCR-1.15_10

ATAATTTTTTATATCAGATAAATTGTACTGGATTTCTTAAAAAATTGCAGTATAATTGCCGCAATTATCCCACCGTTTAT

TTTTTGAGTAGTTTCTCTAGATGCAGCATACTTCTGTGTGGTACCGACGCTCGGTCAGTCCGTTTGTTCTTGTGGCGAGT

GTTGCCGTTTTCTTGACCGCGACCGCCAATCTTACCTTTTTTGATAAAATCAGCCAAACCTATCCCATCGCGGACAATCT

CGGCTTTGTGCTGACGATCGCTGTCGTGCTCTTTGGCGCGATGCTACTGATCACCACGCTGTTATCATCGTATCGCTATG

TGCTAAAGCCTGTGTTGATTTTGCTATTAATCATGGGCGCGGTGACCAGTTATTTTACTGACACTTATGGCACGGTCTAT

GATACGACCATGCTCCAAAATGCCCTACAGACCGACCAAGCCGAGACCAAGGATCTATTAAACGCAGCGTTTATCATGCG

TATCATTGGTTTGGGTGTGCTACCAAGTTTGCTTGTGGCTTTTGTTAAGGTGGATTATCCGACTTGGGGCAAGGGTTTGA

TGCGCCGATTGGGCTTGATCGTGGCAAGTCTTGCGCTGATTTTACTGCCTGTGGTGGCGTTCAGCAGTCATTATGCCAGT

TTCTTTCGCGTGCATAAGCCGCTGCGTAGCTATGTCAATCCGATCATGCCAATCTACTCGGTGGGTAAGCTTGCCAGTAT

TGAGTATAAAAAAGCCAGTGCGCCAAAAGATACCATTTATCACGCCAAAGACGCGGTACAAGCAACCAAGCCTGATATGC

GTAAGCCACGCCTAGTGGTGTTCGTCGTCGGTGAGACGGCACGCGCCGATCATGTCAGCTTCAATGGCTATGAGCGCGAT

ACTTTCCCACAGCTTGCCAAGATCGATGGCGTGACCAATTTTAGCAATGTCAAATCGTGCGGCACATCGACGGCGTATTC

TGTGCCGTGTATGTTCAGCTATCTGGGCGCGGATGAGTATGATGTCGATACCGCCAAATACCAAGAAAATGTGCTGGATA
CGCTGGATCGCTTGGGCGTAAGTATCTTGTGGCGTGATAATAATTCGGACTCAAAAGGCGTGATGGATAAGCTGCCAAAA
GCGCAATTTGCCGATTATAAATCCGCGACCAACAACGCCATCTGCAACACCAATCCTTATAACGAATGCCGCGATGTCGG
TATGCTCGTTGGCTTAGATGACTTTGTCGCTGCCAATAACGGCAAAGATATGCTGATCATGCTGCACCAAATGGGCAATC
ACGGGCCTGCGTATTTTAAGCGATATGATGAAAAGTTTGCCAAATTCACGCCAGTGTGTGAAGGTAATGAGCTTGCCAAG
TGCGAACATCAGTCCTTGATCAATGCTTATGACAATGCCTTGCTTGCCACCGATGATTTCATCGCTCAAAGTATCCAGTG
GCTGCAGACGCACAGCAATGCCTATGATGTCTCAATGCTGTATGTCAGCGATCATGGCGAAAGTCTGGGTGAGAACGGTG
TCTATCTACATGGTATGCCAAATGCCTTTGCACCAAAAGAACAGCGCAGTGTGCCTGCATTTTTCTGGACGGATAAGCAA
ACTGGCATCACGCCAATGGCAACCGATACCGTCCTGACCCATGACGCGATCACGCCGACATTATTAAAGCTGTTTGATGT
CACCGCGGACAAAGTCAAAGACCGCACCGCATTCATCCGCTGATTTCTCCCTGTATTTTTCCAAACCCACCGCACACTC
CATTCGTATTATGGGCGGTGGGGTGGGGTTTGTTATGCCGTATTTATCAAATAAACGCCTACT

>Klebsiella_pneumoniae_mcr-1.15_11

CAAAAAGCCATGATTGAATTGCTGGATTCACATATATTGCGGTCAATTACGCCAGACCGTGGTAAAGAATTTGCCCAACA
TCGTTTGGTAACAGAAGCACTGGGTGTAGAATTTTACTTCCCCGAGCCGCATCAACCGTGGACACGGGGAACGAATGAAA
ATACAAATGGGTTACTTCGTGAATACTTTCCGAAGCACCAAGACATCAATCAGTGGAGCGAAGTTGATATTCAACAGGTG
ATCAATAAACTGAATTTACGACCACGTAAATGTTTAGGTTGGAAAACACCTTATGAAGTTTACTTCAAAAAATCGTTGCA
CTTGGTTTGACAATTCAAGATACAAATTATAAATACTCTCAAGTGTATATTCAGTATGGGATTGCGCAATGATTGCCTAA
TAAAATTTCTGAAATATTTCTGTATCGCATAATTTTTTATATCAGATAAATTGTACTGGATTTCTTAAAAAATTGCAGTA

TAATTGCCGCAATTATCCCACCGTTTATTTTTTGAGTAGTTTCTCTAGATGCAGCATACTTCTGTGTGGTACCGACGCTC
GGTCAGTCCGTTTGTTCTTGTGGCGAGTGTTGCCGTTTTCTTGACCGCGACCGCCAATCTTACCTTTTTTGATAAAATCA
GCCAAACCTATCCCATCGCGGACAATCTCGGCTTTGTGCTGACGATCGCTGTCGTGCTCTTTGGCGCGATGCTACTGATC
ACCACGCTGTTATCATCGTATCGCTATGTGCTAAAGCCTGTGTTGATTTTGCTATTAATCATGGGCGCGGTGACCAGTTA
TTTTACTGACACTTATGGCACGGTCTATGATACGACCATGCTCCAAAATGCCCTACAGACCGACCAAGCCGAGACCAAGG
ATCTATTAAACGCAGCGTTTATCATGCGTATCATTGGTTTGGGTGTGCTACCAAGTTTGCTTGTGGCTTTTGTTAAGGTG
GATTATCCGACTTGGGGCAAGGGTTTGATGCGCCGATTGGGCTTGATCGTGGCAAGTCTTGCGCTGATTTTACTGCCTGT
GGTGGCGTTCAGCAGTCATTATGCCAGTTTCTTTCGCGTGCATAAGCCGCTGCGTAGCTATGTCAATCCGATCATGCCAA
TCTACTCGGTGGGTAAGCTTGCCAGTATTGAGTATAAAAAAGCCAGTGCGCCAAAAGATACCATTTATCACGCCAAAGAC
GCGGTACAAGCAACCAAGCCTGATATGCGTAAGCCACGCCTAGTGGTGTTCGTCGTCGGTGAGACGGCACGCGCCGATCA
TGTCAGCTTCAATGGCTATGAGCGCGATACTTTCCCACAGCTTGCCAAGATCGATGGCGTGACCAATTTTAGCAATGTCA
AATCGTGCGGCACATCGACGGCGTATTCTGTGCCGTGTATGTTCAGCTATCTGGGCGCGGATGAGTATGATGTCGATACC
GCCAAATACCAAGAAAATGTGCTGGATACGCTGGATCGCTTGGGCGTAAGTATCTTGTGGCGTGATAATAATTCGGACTC
AAAAGGCGTGATGGATAAGCTGCCAAAAGCGCAATTTGCCGATTATAAATCCGCGACCAACAACGCCATCTGCAACACCA
ATCCTTATAACGAATGCCGCGATGTCGGTATGCTCGTTGGCTTAGATGACTTTGTCGCTGCCAATAACGGCAAAGATATG
CTGATCATGCTGCACCAAATGGGCAATCACGGGCCTGCGTATTTTAAGCGATATGATGAAAAGTTTGCCAAATTCACGCC
AGTGTGTGAAGGTAATGAGCTTGCCAAGTGCGAACATCAGTCCTTGATCAATGCTTATGACAATGCCTTGCTTGCCACCG
ATGATTTCATCGCTCAAAGTATCCAGTGGCTGCAGACGCACAGCAATGCCTATGATGTCTCAATGCTGTATGTCAGCGAT
CATGGCGAAAGTCTGGGTGAGAACGGTGTCTATCTACATGGTATGCCAAATGCCTTTGCACCAAAAGAACAGCGCAGTGT

GCCTGCATTTTTCTGGACGGATAAGCAAACTGGCATCACGCCAATGGCAACCGATACCGTCCTGACCCATGACGCGATCA
CGCCGACATTATTAAAGCTGTTTGATGTCACCGCGGACAAAGTCAAAGACCGCACCGCATTCATCCGCTGATTTCTCCCT
GTATTTTTTCCAAACCCACCGCACACTCCATTCGTATTATGGGCGGTGGGGTGGGGTTTGTTATGCCGTATTTATCAAAT
AAACGCCTACTTGCTGAGATGAGTATCGCTCTTGTCATGGCGATCGTTGCCACGCTGACCCTTGAGCACAGTCAGATTGA
TCTGATGGTCGCTGATTGGTTTTATCTGGGTATGGGGCATTGGATGGTTGCCAAGCAAGCTTTTTTGCCAGATTTGCTAC
TGTATTCTGGACTAAAAAAGCTGCTGATGGCGATGCTGATCTACTTGCTGGTTGCGACCATTTGCCGTGCTTATCATGAG
AAAAAGGGCAATGCTATCACTGCCAAGTGGCTTGTCCCAGTGACAAAATTTCGCGTGCGTGAGCTTGCGTATCTGGTGCT
GACTTTGATCCTAGTGCCGACAGTTGTCGCGTCATTGAAGGCATATACTCATGTGGTCTGCCCTGTGCATTTGACGATTT
TTGATGGTACGCTGCCGTATTTGCCGATGCTTGATAGTATGCGTAACACCATTCCTGATAAGTGCTTTCCTGCGGCGCAT
GCCAGTAGCGGATTTGCGCTGTTTGCCTTTGCGTTTGCGCCAAGTTTGCGCCGCCGTCGTGGTGCGATCATCATCGTGGT
GATGGCATTGGGCTGGGCGATGGGCTGCTATAAGATGATTATTGGCGATCATTTTTTGAGCCATACGGTGGTGTCGATGA
TGCTTGCGTGGGCGATGTCGGCAGGGCTTGCGTGGGTGTTTTTTAAGAAGGGTGAACAAGTTTAATCGGCTGAATTTACA
ATCCAAGTGCAACAAAAAAAGAAGTACTCATCAACTAGAATATAATTTTGTTTCCACACAAAAACCATTTCCAAATGATG
AGTACTTCCTACCGACATCTTACAATAAACGAGCGAGAAAAGATAATGATTTTACTCGCACAGGGCAAAAAACAAGCAGA
AATTGCCAAAGCACTGGGACGTAGCTCCAGCACCATTTCTCGCGAGCTGAAACGACACGCTCTAGAAAGCTACAGTGCAA
CGAACGCACAAAACAGCTATTTGAAGCATCGTCAAAATAGCAAAGCACAGCGCAAATTAGAGCAGCCTGAATATTTCAAT
TTGGTGCAAGAAAAGTTTCTGACAGAAAACTGGTCGCCCGAACAAATCAGCGCACGATTAAAATTGGAAAAATCTGAATT
ATCCATTAGTTATTCAACCATTTATCGTGGTATTTATTTAGGGTTGTTTGATATAGGCGAACGCAAAGCCAGTCGCAAAC
TGCGCCACAAAGGCAAAACACGGCATACAAAAAATCATCATGAAAAACGTGGCAAAATTCAGATATCCAACCATTTGAAC

GACCGTCCCATTTCGGCGCAAAATCGCAGTCGCTTTGGACATTGGGAAGCCGATACCGTACTGGGTAAAGCGGGTGGAGC
TTGTTTGCTGACGCTGACGGAACGCAAAAGTCGTTTTGAGTTGGTGAAGAAAATTCCTGCCAAAAAAGCCGAAGCAGTCC
AAAAAGCCATGATTGAATTGCTGGATTCACATATATTGCGGTCAATTACGCCAGACCGTGGTAAAGAATTTGCCCAACAT
CGTTTGGTAACAGAAGCACTGGGTGTAGAATTTTACTTCCCCGAG

>Klebsiella_pneumoniae_MCR-1.14_12
CGCATAATTTTTTATATCAGATAAATTGTACTGGATTTCTTAAAAAATTGCAGTATAATTGCCGTAATTATCCCACCGTT
TATTTTTTGAGTAGTTTCTCATGATGCAGCATACTTCTGTGTGGTACCGACGCTCGGTCAGTCCGTTTGTTCTTGTGGCG
AGTGTTGCCGTTTTCTTGACCGCGACCGCCAATCTTACCTTTTTTGATAAGGTCAGCCAAACCTATCCCATCGCGGACAA
TCTCGGCTTTGTGCTGACGATCGCTGTCGTGCTCTTTGGCGCGATGCTACTGATCACCACGCTGTTATCATCGTATCGCT
ATGTGCTAAAGCCTGTGTTGATTTTGCTATTAATCATGGGCGCGGTGACCAGTTATTTTACTGACACTTATGGCACGGTC
TATGATACGACCATGCTCCAAAATGCCCTACAGACCGACCAAGCCGAGACCAAGGATCTATTAAACGCAGCGTTTATCAT
GCGTATCATTGGTTTGGGTGTGCTACCAAGTTTGCTTGTGGCTTTTGTTAAGGTGGATTATCCGACTTGGGGCAAGGGTT
TGATGCGCCGATTGGGCTTGATCGTGGCAAGTCTTGCGCTGATTTTACTGCCTGTGGTGGCGTTCAGCAGTCATTATGCC
AGTTTCTTTCGCGTGCATAAGCCGCTGCGTAGCTATGTCAATCCGATCATACCAATCTACTCGGTGGGTAAGCTTGCCAG
TATTGAGTATAAAAAAGCCAGTGCGCCAAAAGATACCATTTATCACGCCAAAGACGCGGTACAAGCAACCAAGCCTGATA
TGCGTAAGCCACGCCTAGTGGTGTTCGTCGTCGGTGAGACGGCACGCGCCGATCATGTCAGCTTCAATGGCTATGAGCGC
GATACTTTCCCACAGCTTGCCAAGATCGATGGCGTGACCAATTTTAGCAATGTCACATCGTGCGGCACATCGACGGCGTA
TTCTGTGCCGTGTATGTTCAGCTATCTGGGCGCGGATGAGTATGATGTCGATACCGCCAAATACCAAGAAAATGTGCTGG

ATACGCTGGATCGCTTGGGCGTAAGTATCTTGTGGCGTGATAATAATTCGGACTCAAAAGGCGTGATGGATAAGCTGCCA

AAAGCGCAATTTGCCGATTATAAATCCGCGACCAACAACGCCATCTGCAACACCAATCCTTATAACGAATGCCGCGATGT

CGGTATGCTCGTTGGCTTAGATGACTTTGTCGCTGCCAATAACGGCAAAGATATGCTGATCATGCTGCACCAAATGGGCA

ATCACGGGCCTGCGTATTTTAAGCGATATGATGAAAAGTTTGCCAAATTCACGCCAGTGTGTGAAGGTAATGAGCTTGCC

AAGTGCGAACATCAGTCCTTGATCAATGCTTATGACAATGCCTTGCTTGCCACCGATGATTTCATCGCTCAAAGTATCCA

GTGGCTGCAGACGCACAGCAATGCCTATGATGTCTCAATGCTGTATGTCAGCGATCATGGCGAAAGTCTGGGTGAGAACG

GTGTCTATCTACATGGTATGCCAAATGCCTTTGCACCAAAAGAACAGCGCAGTGTGCCTGCATTTTTCTGGACGGATAAG

CAAACTGGCATCACGCCAATGGCAACCGATACCGTCCTGACCCATGACGCGATCACGCCGACATTATTAAAGCTGTTTGA

TGTCACCGCGGACAAAGTCAAAGACCGCACCGCATTCATCCGCTGATTTCTCCCTGTATTTTTCCAAACCCACCGCACA

CTCCATTCGTATTATGGGCGGTGGGGTGGGGTTTGTTATGCCGTATTTATCAAATAAACGCCTACT

>Enterobacter_cloacae_MCR-1_13

TGATGCAGCATACTTCTGTGAGGTACCGACGCTCGGTCAGTCCGTTTGTTCTTGTGGCGAGTGTTGCCGTTTTCTTGACC

GCGACCGCCAATCTTACCTTTTTTGATAAAATCAGCCAAACCTATCCCATCGCGGACAATCTCGGCTTTGTGCTGACGAT

CGCTGTCGTGCTCTTTGGCGCGATGCTACTGATCACCACGCTGTTATCATCGTATCGCTATGTGCTAAAGCCTGTGTTGA

TTTTGCTATTAATCATGGGCGCGGTGACCAGTTATTTTACTGACACTTATGGCACGGTCTATGATACGACCATGCTCCAA

AATGCCCTACAGACCGACCAAGCCGAGACCAAGGATCTATTAAACGCAGCGTTTATCATGCGTATCATTGGTTTGGGTGT

GCTACCAAGTTTGCTTGTGGCTTTTGTTAAGGTGGATTATCCGACTTGGGGCAAGGGTTTGATGCGCCGATTGGGCTTGA

TCGTGGCAAGTCTTGCGCTGATTTTACTGCCTGTGGTGGCGTTCAGCAGTCATTATGCCAGTTTCTTTCGCGTGCATAAG

CCGCTGCGTAGCTATGTCAATCCGATCATGCCAATCTACTCGGTGGGTAAGCTTGCCAGTATTGAGTATAAAAAAGCCAG

TGCGCCAAAAGATACCATTTATCACGCCAAAAGACGCGGTACAGCAACCAAGCCTGATATGCGTAAGCCACGCCTAGTGG

TGTTCGTCGTCGGTGAGACGGCACGCGCCGATCATGTCAGCTTCAATGGCTATGAGCGCGATACTTTCCCACAGCTTGCC

AAGATCGATGGCGTGACCAATTTTAGCAATGTCACATCGTGCGGCACATCGACGGCGTATTCTGTGCCGTGTATGTTCAG

CTATCTGGGCGCGGATGAGTATGATGTCGATACCGCCAAATACCAAGAAAATGTGCTGGATACGCTGGATCGCTTGGGCG

TAAGTATCTTGTGGCGTGATAATAATTCGGACTCAAAAGGCGTGATGGATAAGCTGCCAAAAGCGCAATTTGCCGATTAT

AAATCCGCGACCAACAACGCCATCTGCAACACCAATCCTTATAACGAATGCCGCGATGTCGGTATGCTCGTTGGCTTAGA

TGACTTTGTCGCTGCCAATAACGGCAAAGATATGCTGATCATGCTGCACCAAATGGGCAATCACGGGCCTGCGTATTTTA

AGCGATATGATGAAAAGTTTGCCAAATTCACGCCAGTGTGTGAAGGTAATGAGCTTGCCAAGTGCGAACATCAGTCCTTG

ATCAATGCTTATGACAATGCCTTGCTTGCCACCGATGATTTCATCGCTCAAAGTATCCAGTGGCTGCAGACGCACAGCAA

TGCCTATGATGTCTCAATGCTGTATGTCAGCGATCATGGCGAAAGTCTGGGTGAGAACGGTGTCTATCTACATGGTATGC

CAAATGCCTTTGCACCAAAAGAACAGCGCAGTGTGCCTGCATTTTTCTGGACGGATAAGCAAACTGGCATCACGCCAATG

GCAACCGATACCGTCCTGACCCATGACGCGATCACGCCGACATTATTAAAGCTGTTTGATG

>Klebsiella_pneumoniae_MCR-1_14

TGATGCAGCATACTTCTGTGAGGTACCGACGCTCGGTCAGTCCGTTTGTTCTTGTGGCGAGTGTTGCCGTTTTCTTGACC

GCGACCGCCAATCTTACCTTTTTTGATAAAATCAGCCAAACCTATCCCATCGCGGACAATCTCGGCTTTGTGCTGACGAT

CGCTGTCGTGCTCTTTGGCGCGATGCTACTGATCACCACGCTGTTATCATCGTATCGCTATGTGCTAAAGCCTGTGTTGA

TTTTGCTATTAATCATGGGCGCGGTGACCAGTTATTTTACTGACACTTATGGCACGGTCTATGATACGACCATGCTCCAA

AATGCCCTACAGACCGACCAAGCCGAGACCAAGGATCTATTAAACGCAGCGTTTATCATGCGTATCATTGGTTTGGGTGT

GCTACCAAGTTTGCTTGTGGCTTTTGTTAAGGTGGATTATCCGACTTGGGGCAAGGGTTTGATGCGCCGATTGGGCTTGA

TCGTGGCAAGTCTTGCGCTGATTTTACTGCCTGTGGTGGCGTTCAGCAGTCATTATGCCAGTTTCTTTCGCGTGCATAAG

CCGCTGCGTAGCTATGTCAATCCGATCATGCCAATCTACTCGGTGGGTAAGCTTGCCAGTATTGAGTATAAAAAAGCCAG

TGCGCCAAAAGATACCATTTATCACGCCAAAAGACGCGGTACAGCAACCAAGCCTGATATGCGTAAGCCACGCCTAGTGG

TGTTCGTCGTCGGTGAGACGGCACGCGCCGATCATGTCAGCTTCAATGGCTATGAGCGCGATACTTTCCCACAGCTTGCC

AAGATCGATGGCGTGACCAATTTTAGCAATGTCACATCGTGCGGCACATCGACGGCGTATTCTGTGCCGTGTATGTTCAG

CTATCTGGGCGCGGATGAGTATGATGTCGATACCGCCAAATACCAAGAAAATGTGCTGGATACGCTGGATCGCTTGGGCG

TAAGTATCTTGTGGCGTGATAATAATTCGGACTCAAAAGGCGTGATGGATAAGCTGCCAAAAGCGCAATTTGCCGATTAT

AAATCCGCGACCAACAACGCCATCTGCAACACCAATCCTTATAACGAATGCCGCGATGTCGGTATGCTCGTTGGCTTAGA

TGACTTTGTCGCTGCCAATAACGGCAAAGATATGCTGATCATGCTGCACCAAATGGGCAATCACGGGCCTGCGTATTTTA

AGCGATATGATGAAAAGTTTGCCAAATTCACGCCAGTGTGTGAAGGTAATGAGCTTGCCAAGTGCGAACATCAGTCCTTG

ATCAATGCTTATGACAATGCCTTGCTTGCCACCGATGATTTCATCGCTCAAAGTATCCAGTGGCTGCAGACGCACAGCAA

TGCCTATGATGTCTCAATGCTGTATGTCAGCGATCATGGCGAAAGTCTGGGTGAGAACGGTGTCTATCTACATGGTATGC

CAAATGCCTTTGCACCAAAAGAACAGCGCAGTGTGCCTGCATTTTTCTGGACGGATAAGCAAACTGGCATCACGCCAATG

GCAACCGATACCGTCCTGACCCATGACGCGATCACGCCGACATTATTAAAGCTGTTTGATG

>Escherichia_albertii_mcr-1_15

CGGTCAGTCCGTTTGTTCTTGTGGCGAGTGTTGCCGTTTTCTTGACCGCGACCGCCAATCTTACCTTTTTTGATAAAATC

AGCCAAACCTATCCCATCGCGGACAATCTCGGCTTTGTGCTGACGATCGCTGTCGTGCTCTTTGGCGCGATGCTACTGAT

CACCACGCTGTTATCATCGTATCGCTATGTGCTAAAGCCTGTGTTGATTTTGCTATTAATCATGGGCGCGGTGACCAGTT

ATTTTACTGACACTTATGGCACGGTCTATGATACGACCATGCTCCAAAATGCCCTACAGACCGACCAAGCCGAGACCAAG

GATCTATTAAACGCAGCGTTTATCATGCGTATCATTGGTTTGGGTGTGCTACCAAGTTTGCTTGTGGCTTTTGTTAAGGT

GGATTATCCGACTTGGGGCAAGGGTTTGATGCGCCGATTGGGCTTGATCGTGGCAAGTCTTGCGCTGATTTTACTGCCTG

TGGTGGCGTTCAGCAGTCATTATGCCAGTTTCTTTCGCGTGCATAAGCCGCTGCGTAGCTATGTCAATCCGATCATGCCA

ATCTACTCGGTGGGTAAGCTTGCCAGTATTGAGTATAAAAAAGCCAGTGCGCCAAAAGATACCATTTATCACGCCAAAGA

CGCGGTACAAGCAACCAAGCCTGATATGCGTAAGCCACGCCTAGTGGTGTTCGTCGTCGGTGAGACGGCACGCGCCGATC

ATGTCAGCTTCAATGGCTATGAGCGCGATACTTTCCCACAGCTTGCCAAGATCGATGGCGTGACCAATTTTAGCAATGTC

ACATCGTGCGGCACATCGACGGCGTATTCTGTGCCGTGTATGTTCAGCTATCTGGGCGCGGATGAGTATGATGTCGATAC

CGCCAAATACCAAGAAAATGTGCTGGATACGCTGGATCGCTTGGGCGTAAGTATCTTGTGGCGTGATAATAATTCGGACT

CAAAAGGCGTGATGGATAAGCTGCCAAAAGCGCAATTTGCCGATTATAAATCCGCGACCAACAACGCCATCTGCAACACC

AATCCTTATAACGAATGCCGCGATGTCGGTATGCTCGTTGGCTTAGATGACTTTGTCGCTGCCAATAACGGCAAAGATAT

GCTGATCATGCTGCACCAAATGGGCAATCACGGGCCTGCGTATTTTAAGCGATATGATGAAAAGTTTGCCAAATTCACGC

CAGTGTGTGAAGGTAATGAGCTTGCCAAGTGCGAACATCAGTCCTTGATCAATGCTTATGACAATGCCTTGCTTGCCACC

GATGATTTCATCGCTCAAAGTATCCAGTGGCTGCAGACGCACAGCAATGCCTATGATGTCTCAATGCTGTATGTCAGCGA

TCATGGCGAAAGTCTGGGTGAGAACGGTGTCTATCTACATGGTATGCCAAATGCCTTTGCACCAAAAGAACAGCGCAGTG

TGCCTGCATTTTTCTGGACGGATAAGCAAACTGGCATCACGCCAATGGCAACCGATACCGTCCTGACCCATGACGCGATC

ACGCCGACATTATTAAAGCTGTT

>Escherichia_albertii_mcr-1_16

CGGTCAGTCCGTTTGTTCTTGTGGCGAGTGTTGCCGTTTTCTTGACCGCGACCGCCAATCTTACCTTTTTTGATAAAATC

AGCCAAACCTATCCCATCGCGGACAATCTCGGCTTTGTGCTGACGATCGCTGTCGTGCTCTTTGGCGCGATGCTACTGAT

CACCACGCTGTTATCATCGTATCGCTATGTGCTAAAGCCTGTGTTGATTTTGCTATTAATCATGGGCGCGGTGACCAGTT
ATTTTACTGACACTTATGGCACGGTCTATGATACGACCATGCTCCAAAATGCCCTACAGACCGACCAAGCCGAGACCAAG
GATCTATTAAACGCAGCGTTTATCATGCGTATCATTGGTTTGGGTGTGCTACCAAGTTTGCTTGTGGCTTTTGTTAAGGT
GGATTATCCGACTTGGGGCAAGGGTTTGATGCGCCGATTGGGCTTGATCGTGGCAAGTCTTGCGCTGATTTTACTGCCTG
TGGTGGCGTTCAGCAGTCATTATGCCAGTTTCTTTCGCGTGCATAAGCCGCTGCGTAGCTATGTCAATCCGATCATGCCA
ATCTACTCGGTGGGTAAGCTTGCCAGTATTGAGTATAAAAAAGCCAGTGCGCCAAAAGATACCATTTATCACGCCAAAGA
CGCGGTACAAGCAACCAAGCCTGATATGCGTAAGCCACGCCTAGTGGTGTTCGTCGTCGGTGAGACGGCACGCGCCGATC
ATGTCAGCTTCAATGGCTATGAGCGCGATACTTTCCCACAGCTTGCCAAGATCGATGGCGTGACCAATTTTAGCAATGTC
ACATCGTGCGGCACATCGACGGCGTATTCTGTGCCGTGTATGTTCAGCTATCTGGGCGCGGATGAGTATGATGTCGATAC
CGCCAAATACCAAGAAAATGTGCTGGATACGCTGGATCGCTTGGGCGTAAGTATCTTGTGGCGTGATAATAATTCGGACT
CAAAAGGCGTGATGGATAAGCTGCCAAAAGCGCAATTTGCCGATTATAAATCCGCGACCAACAACGCCATCTGCAACACC
AATCCTTATAACGAATGCCGCGATGTCGGTATGCTCGTTGGCTTAGATGACTTTGTCGCTGCCAATAACGGCAAAGATAT
GCTGATCATGCTGCACCAAATGGGCAATCACGGGCCTGCGTATTTTAAGCGATATGATGAAAAGTTTGCCAAATTCACGC
CAGTGTGTGAAGGTAATGAGCTTGCCAAGTGCGAACATCAGTCCTTGATCAATGCTTATGACAATGCCTTGCTTGCCACC
GATGATTTCATCGCTCAAAGTATCCAGTGGCTGCAGACGCACAGCAATGCCTATGATGTCTCAATGCTGTATGTCAGCGA
TCATGGCGAAAGTCTGGGTGAGAACGGTGTCTATCTACATGGTATGCCAAATGCCTTTGCACCAAAAGAACAGCGCAGTG
TGCCTGCATTTTTCTGGACGGATAAGCAAACTGGCATCACGCCAATGGCAACCGATACCGTCCTGACCCATGACGCGATC
ACGCCGACATTATTAAAGCTGTT

>Escherichia_albertii_mcr-1_17

CGGTCAGTCCGTTTGTTCTTGTGGCGAGTGTTGCCGTTTTCTTGACCGCGACCGCCAATCTTACCTTTTTTGATAAAATC

AGCCAAACCTATCCCATCGCGGACAATCTCGGCTTTGTGCTGACGATCGCTGTCGTGCTCTTTGGCGCGATGCTACTGAT

CACCACGCTGTTATCATCGTATCGCTATGTGCTAAAGCCTGTGTTGATTTTGCTATTAATCATGGGCGCGGTGACCAGTT

ATTTTACTGACACTTATGGCACGGTCTATGATACGACCATGCTCCAAAATGCCCTACAGACCGACCAAGCCGAGACCAAG

GATCTATTAAACGCAGCGTTTATCATGCGTATCATTGGTTTGGGTGTGCTACCAAGTTTGCTTGTGGCTTTTGTTAAGGT

GGATTATCCGACTTGGGGCAAGGGTTTGATGCGCCGATTGGGCTTGATCGTGGCAAGTCTTGCGCTGATTTTACTGCCTG

TGGTGGCGTTCAGCAGTCATTATGCCAGTTTCTTTCGCGTGCATAAGCCGCTGCGTAGCTATGTCAATCCGATCATGCCA

ATCTACTCGGTGGGTAAGCTTGCCAGTATTGAGTATAAAAAAGCCAGTGCGCCAAAAGATACCATTTATCACGCCAAAGA

CGCGGTACAAGCAACCAAGCCTGATATGCGTAAGCCACGCCTAGTGGTGTTCGTCGTCGGTGAGACGGCACGCGCCGATC

ATGTCAGCTTCAATGGCTATGAGCGCGATACTTTCCCACAGCTTGCCAAGATCGATGGCGTGACCAATTTTAGCAATGTC

ACATCGTGCGGCACATCGACGGCGTATTCTGTGCCGTGTATGTTCAGCTATCTGGGCGCGGATGAGTATGATGTCGATAC

CGCCAAATACCAAGAAAATGTGCTGGATACGCTGGATCGCTTGGGCGTAAGTATCTTGTGGCGTGATAATAATTCGGACT

CAAAAGGCGTGATGGATAAGCTGCCAAAAGCGCAATTTGCCGATTATAAATCCGCGACCAACAACGCCATCTGCAACACC

AATCCTTATAACGAATGCCGCGATGTCGGTATGCTCGTTGGCTTAGATGACTTTGTCGCTGCCAATAACGGCAAAGATAT

GCTGATCATGCTGCACCAAATGGGCAATCACGGGCCTGCGTATTTTAAGCGATATGATGAAAAGTTTGCCAAATTCACGC

CAGTGTGTGAAGGTAATGAGCTTGCCAAGTGCGAACATCAGTCCTTGATCAATGCTTATGACAATGCCTTGCTTGCCACC

GATGATTTCATCGCTCAAAGTATCCAGTGGCTGCAGACGCACAGCAATGCCTATGATGTCTCAATGCTGTATGTCAGCGA

TCATGGCGAAAGTCTGGGTGAGAACGGTGTCTATCTACATGGTATGCCAAATGCCTTTGCACCAAAAGAACAGCGCAGTG

TGCCTGCATTTTTCTGGACGGATAAGCAAACTGGCATCACGCCAATGGCAACCGATACCGTCCTGACCCATGACGCGATC

ACGCCGACATTATTAAAGCTGTTGA

>Escherichia_albertii_mcr-1_18

CGGTCAGTCCGTTTGTTCTTGTGGCGAGTGTTGCCGTTTTCTTGACCGCGACCGCCAATCTTACCTTTTTTGATAAAATC

AGCCAAACCTATCCCATCGCGGACAATCTCGGCTTTGTGCTGACGATCGCTGTCGTGCTCTTTGGCGCGATGCTACTGAT

CACCACGCTGTTATCATCGTATCGCTATGTGCTAAAGCCTGTGTTGATTTTGCTATTAATCATGGGCGCGGTGACCAGTT

ATTTTACTGACACTTATGGCACGGTCTATGATACGACCATGCTCCAAAATGCCCTACAGACCGACCAAGCCGAGACCAAG

GATCTATTAAACGCAGCGTTTATCATGCGTATCATTGGTTTGGGTGTGCTACCAAGTTTGCTTGTGGCTTTTGTTAAGGT

GGATTATCCGACTTGGGGCAAGGGTTTGATGCGCCGATTGGGCTTGATCGTGGCAAGTCTTGCGCTGATTTTACTGCCTG

TGGTGGCGTTCAGCAGTCATTATGCCAGTTTCTTTCGCGTGCATAAGCCGCTGCGTAGCTATGTCAATCCGATCATGCCA

ATCTACTCGGTGGGTAAGCTTGCCAGTATTGAGTATAAAAAAGCCAGTGCGCCAAAAGATACCATTTATCACGCCAAAGA

CGCGGTACAAGCAACCAAGCCTGATATGCGTAAGCCACGCCTAGTGGTGTTCGTCGTCGGTGAGACGGCACGCGCCGATC

ATGTCAGCTTCAATGGCTATGAGCGCGATACTTTCCCACAGCTTGCCAAGATCGATGGCGTGACCAATTTTAGCAATGTC

ACATCGTGCGGCACATCGACGGCGTATTCTGTGCCGTGTATGTTCAGCTATCTGGGCGCGGATGAGTATGATGTCGATAC

CGCCAAATACCAAGAAAATGTGCTGGATACGCTGGATCGCTTGGGCGTAAGTATCTTGTGGCGTGATAATAATTCGGACT

CAAAAGGCGTGATGGATAAGCTGCCAAAAGCGCAATTTGCCGATTATAAATCCGCGACCAACAACGCCATCTGCAACACC

AATCCTTATAACGAATGCCGCGATGTCGGTATGCTCGTTGGCTTAGATGACTTTGTCGCTGCCAATAACGGCAAAGATAT

GCTGATCATGCTGCACCAAATGGGCAATCACGGGCCTGCGTATTTTAAGCGATATGATGAAAAGTTTGCCAAATTCACGC

CAGTGTGTGAAGGTAATGAGCTTGCCAAGTGCGAACATCAGTCCTTGATCAATGCTTATGACAATGCCTTGCTTGCCACC

GATGATTTCATCGCTCAAAGTATCCAGTGGCTGCAGACGCACAGCAATGCCTATGATGTCTCAATGCTGTATGTCAGCGA

TCATGGCGAAAGTCTGGGTGAGAACGGTGTCTATCTACATGGTATGCCAAATGCCTTTGCACCAAAAGAACAGCGCAGTG

TGCCTGCATTTTTCTGGACGGATAAGCAAACTGGCATCACGCCAATGGCAACCGATACCGTCCTGACCCATGACGCGATC

ACGCCGACATTATTAAAGCTGT

>Moraxella_sp.MCR-1.10_19

ATGGTGCAGCATACTTCTGTGTGGTACCGATGCTCGGTCAGTCCGTTTGTTCTTGTGGCAAGTGTTTCCGTTTTCTTGAC

CGCGACCGCCAATCTTACCTTTTTTGATAAAATCAGCCAAACCTATCCCATCGCGGACAATCTCGGCTTTGTGCTGACGA

TCGCTGTCGTGCTCTTTGGCGCGATGCTACTGATCACCACACTGTTATCATCGTATCGCTATGTGCTAAAGCCTGTGTTG

ATTTTGCTATTAATCATGGGCGCGGTGACTAGTTATTTTACTGACACTTATGGCACGGTCTATGACACGACCATGCTCCA

AAATGCCCTACAGACCGACCAAGCCGAGACCAAGGATCTATTAAACGCAGCGTTTATCATGCGTATCATTGGTTTGGGTG

TACTACCAAGTTTGCTTGTGGCTTTTGTCAAGGTGGATTATCCGACTTGGGGCAAGGGTTTGGTGCGCCGATTGGGCTTG

ATCGTGGCAAGTCTTGCGCTGATTTTACTGCCTGTGGTGGCGTTCAGCAGTCATTATGCCAGTTTTTTTCGCGTGCATAA

GCCGCTGCGTTCGTATGTCAATCCGATCATGCCAATCTACTCGGTGGGTAAGCTTGCCAGTATTGAGTATAAAAAAGCCA

GTGCGCCAAAAGATACCATTTATCACGCCAAAGACGCGGTACAAGCAACCAAGCCTGATACGCGTAAGCCACGCCTAGTG

GTGTTCGTCGTCGGTGAGACGGCACGCGCCGATCATGTCAGCTTCAATGGCTATGAGCGCGATACTTTCCCACAGCTTGC

CAAGATCGATGGCGTGACCAATTTTAGCAATGTCACATCGTGCGGCACATCGACGGCGTATTCTGTGCCGTGTATGTTCA

GCTATCTGGGCGCGGATGAGTATGATGTCGATACCGCCAAATACCAAGAAAATGTGCTTGATACGCTGGATCGTTTGGGC

GTGAGCATCTTGTGGCGTGATAATAATTCGGACTCAAAAGGCGTGATGGATAAGCTGCCAAAAGCGCAATTTGCCGATTA

TAAATCCGCAACCAACAACACCATCTGCAACACCAATCCTTATAACGAATGCCGCGATGTCGGTATGCTCGTTGGCTTAG

ATGACTTTGTCGCTGCCAATAACGGCAAAGATATGCTGATCATGCTGCACCAAATGGGCAATCACGGGCCTGCGTATTTT

AAGCGATATGATGAAAAGTTTGCCAAATTCACGCCAGTGTGTGAAGGTAATGAGCTTGCCAAGTGCGAACATCAGTCTTT
GATCAATGCTTATGACAATGCCTTGCTTGCCACCGATGATTTCATCACTCAAAGTATCCAGTGGCTGCAGACGCACAGCA
ATGCCTATGATGTCTCGATGCTGTATGTCAGCGATCATGGCGAGAGTCTAGGTGAGAATGGTGTCTATCTACATGGTATG
CCTAATGCCTTTGCACCAAAAGAACAGCGCAGTGTGCCTGCATTTTTCTGGACAGATAAGCAAACTGGCATCACGCCGAT
GGCGACCGATACTGTCCTGACCCATGATGCGATCACACCAACATTATTAAAGCTGTTCGATGTTACCGCAGATAAAGTCA
AAGACCGCACCGCATTCATCCGCTGA

>Moraxella_sp.mcr-1_20
ATGGTGCAGCATACTTCTGTGTGGTACCGATGCTCGGTCAGTCCGTTTGTTCTTGTGGCAAGTGTTTCCGTTTTCTTGAC
CGCGACCGCCAATCTTACCTTTTTTGATAAAATCAGCCAAACCTATCCCATCGCGGACAATCTCGGCTTTGTGCTGACGA
TCGCTGTCGTGCTCTTTGGCGCGATGCTACTGATCACCACACTGTTATCATCGTATCGCTATGTGCTAAAGCCTGTGTTG
ATTTTGCTATTAATCATGGGCGCGGTGACTAGTTATTTTACTGACACTTATGGCACGGTCTATGACACGACCATGCTCCA
AAATGCCCTACAGACCGACCAAGCCGAGACCAAGGATCTATTAAACGCAGCGTTTATCATGCGTATCATTGGTTTGGGTG
TACTACCAAGTTTGCTTGTGGCTTTTGTCAAGGTGGATTATCCGACTTGGGGCAAGGGTTTGGTGCGCCGATTGGGCTTG
ATCGTGGCAAGTCTTGCGCTGATTTTACTGCCTGTGGTGGCGTTCAGCAGTCATTATGCCAGTTTTTTTCGCGTGCATAA
GCCGCTGCGTTCGTATGTCAATCCGATCATGCCAATCTACTCGGTGGGTAAGCTTGCCAGTATTGAGTATAAAAAAGCCA
GTGCGCCAAAAGATACCATTTATCACGCCAAAGACGCGGTACAAGCAACCAAGCCTGATACGCGTAAGCCACGCCTAGTG
GTGTTCGTCGTCGGTGAGACGGCACGCGCCGATCATGTCAGCTTCAATGGCTATGAGCGCGATACTTTCCCACAGCTTGC
CAAGATCGATGGCGTGACCAATTTTAGCAATGTCACATCGTGCGGCACATCGACGGCGTATTCTGTGCCGTGTATGTTCA

GCTATCTGGGCGCGGATGAGTATGATGTCGATACCGCCAAATACCAAGAAAATGTGCTTGATACGCTGGATCGTTTGGGC

GTGAGCATCTTGTGGCGTGATAATAATTCGGACTCAAAAGGCGTGATGGATAAGCTGCCAAAAGCGCAATTTGCCGATTA

TAAATCCGCAACCAACAACACCATCTGCAACACCAATCCTTATAACGAATGCCGCGATGTCGGTATGCTCGTTGGCTTAG

ATGACTTTGTCGCTGCCAATAACGGCAAAGATATGCTGATCATGCTGCACCAAATGGGCAATCACGGGCCTGCGTATTTT

AAGCGATATGATGAAAAGTTTGCCAAATTCACGCCAGTGTGTGAAGGTAATGAGCTTGCCAAGTGCGAACATCAGTCTTT

GATCAATGCTTATGACAATGCCTTGCTTGCCACCGATGATTTCATCACTCAAAGTATCCAGTGGCTGCAGACGCACAGCA

ATGCCTATGATGTCTCGATGCTGTATGTCAGCGATCATGGCGAGAGTCTAGGTGAGAATGGTGTCTATCTACATGGTATG

CCTAATGCCTTTGCACCAAAAGAACAGCGCAGTGTGCCTGCATTTTTCTGGACAGATAAGCAAACTGGCATCACGCCGAT

GGCGACCGATACTGTCCTGACCCATGATGCGATCACACCAACATTATTAAAGCTGTTCGATGTTACCGCAGATAAAGTCA

AAGACCGCACCGCATTCATCCGCTGA

>Providencia_alcalifaciens_mcr-1_20

CGCGACCGCCAATCTTACCTTTTTTGATAAAATCAGCCAAACCTATCCCATCGCGGACAATCTCGGCTTTGTGCTGACGA

TCGCTGTCGTGCTCTTTGGCGCGATGCTACTGATCACCACGCTGTTATCATCGTATCGCTATGTGCTAAAGCCTGTGTTG

ATTTTGCTATTAATCATGGGCGCGGTGACCAGTTATTTTACTGACACTTATGGCACGGTCTATGATACGACCATGCTCCA

AAATGCCCTACAGACCGACCAAGCCGAGACCAAGGATCTATTAAACGCAGCGTTTATCATGCGTATCATTGGTTTGGGTG

TGCTACCAAGTTTGCTTGTGGCTTTTGTTAAGGTGGATTATCCGACTTGGGGCAAGGGTTTGATGCGCCGATTGGGCTTG

ATCGTGGCAAGTCTTGCGCTGATTTTACTGCCTGTGGTGGCGTTCAGCAGTCATTATGCCAGTTTCTTTCGCGTGCATAA

GCCGCTGCGTAGCTATGTCAATCCGATCATGCCAATCTACTCGGTGGGTAAGCTTGCCAGTATTGAGTATAAAAAAGCCA
GTGCGCCAAAAGATACCATTTATCACGCCAAAGACGCGGTACAAGCAACCAAGCCTGATATGCGTAAGCCACGCCTAGTG
GTGTTCGTCGTCGGTGAGACGGCACGCGCCGATCATGTCAGCTTCAATGGCTATGAGCGCGATACTTTCCCACAGCTTGC
CAAGATCGATGGCGTGACCAATTTTAGCAATGTCACATCGTGCGGCACATCGACGGCGTATTCTGTGCCGTGTATGTTCA
GCTATCTGGGCGCGGATGAGTATGATGTCGATACCGCCAAATACCAAGAAAATGTGCTGGATACGCTGGATCGCTTGGGC
GTAAGTATCTTGTGGCGTGATAATAATTCGGACTCAAAAGGCGTGATGGATAAGCTGCCAAAAGCGCAATTTGCCGATTA
TAAATCCGCGACCAACAACGCCATCTGCAACACCAATCCTTATAACGAATGCCGCGATGTCGGTATGCTCGTTGGCTTAG
ATGACTTTGTCGCTGCCAATAACGGCAAAGATATGCTGATCATGCTGCACCAAATGGGCAATCACGGGCCTGCGTATTTT
AAGCGATATGATGAAAAGTTTGCCAAATTCACGCCAGTGTGTGAAGGTAATGAGCTTGCCAAGTGCGAACATCAGTCCTT
GATCAATGCTTATGACAATGCCTTGCTTGCCACCGATGATTTCATCGCTCAAAGTATCCAGTGGCTGCAGACGCACAGCA
ATGCCTATGATGTCTCAATGCTGTATGTCAGCGATCATGGCGAAAGTCTGGGTGAGAACGGTGTCTATCTACATGGTATG
CCAAATGCCTTTGCACCAAAAGAACAGCGCAGTGTGCCTGCATTTTTCTGGACGGATAAGCAAACTGGCATCACGCCAAT
GGCAACCGATACCGTCCTGACCCATGACGCGATCACGCCGACATTATTAAAGCTGTT

>Enterobacter_cloacae_mcr-1_21

CGCGACCGCCAATCTTACCTTTTTTGATAAAATCAGCCAAACCTATCCCATCGCGGACAATCTCGGCTTTGTGCTGACGA
TCGCTGTCGTGCTCTTTGGCGCGATGCTACTGATCACCACGCTGTTATCATCGTATCGCTATGTGCTAAAGCCTGTGTTG
ATTTTGCTATTAATCATGGGCGCGGTGACCAGTTATTTTACTGACACTTATGGCACGGTCTATGATACGACCATGCTCCA
AAATGCCCTACAGACCGACCAAGCCGAGACCAAGGATCTATTAAACGCAGCGTTTATCATGCGTATCATTGGTTTGGGTG

TGCTACCAAGTTTGCTTGTGGCTTTTGTTAAGGTGGATTATCCGACTTGGGGCAAGGGTTTGATGCGCCGATTGGGCTTG

ATCGTGGCAAGTCTTGCGCTGATTTTACTGCCTGTGGTGGCGTTCAGCAGTCATTATGCCAGTTTCTTTCGCGTGCATAA

GCCGCTGCGTAGCTATGTCAATCCGATCATGCCAATCTACTCGGTGGGTAAGCTTGCCAGTATTGAGTATAAAAAAGCCA

GTGCGCCAAAAGATACCATTTATCACGCCAAAGACGCGGTACAAGCAACCAAGCCTGATATGCGTAAGCCACGCCTAGTG

GTGTTCGTCGTCGGTGAGACGGCACGCGCCGATCATGTCAGCTTCAATGGCTATGAGCGCGATACTTTCCCACAGCTTGC

CAAGATCGATGGCGTGACCAATTTTAGCAATGTCACATCGTGCGGCACATCGACGGCGTATTCTGTGCCGTGTATGTTCA

GCTATCTGGGCGCGGATGAGTATGATGTCGATACCGCCAAATACCAAGAAAATGTGCTGGATACGCTGGATCGCTTGGGC

GTAAGTATCTTGTGGCGTGATAATAATTCGGACTCAAAAGGCGTGATGGATAAGCTGCCAAAAGCGCAATTTGCCGATTA

TAAATCCGCGACCAACAACGCCATCTGCAACACCAATCCTTATAACGAATGCCGCGATGTCGGTATGCTCGTTGGCTTAG

ATGACTTTGTCGCTGCCAATAACGGCAAAGATATGCTGATCATGCTGCACCAAATGGGCAATCACGGGCCTGCGTATTTT

AAGCGATATGATGAAAAGTTTGCCAAATTCACGCCAGTGTGTGAAGGTAATGAGCTTGCCAAGTGCGAACATCAGTCCTT

GATCAATGCTTATGACAATGCCTTGCTTGCCACCGATGATTTCATCGCTCAAAGTATCCAGTGGCTGCAGACGCACAGCA

ATGCCTATGATGTCTCAATGCTGTATGTCAGCGATCATGGCGAAAGTCTGGGTGAGAACGGTGTCTATCTACATGGTATG

CCAAATGCCTTTGCACCAAAAGAACAGCGCAGTGTGCCTGCATTTTTCTGGACGGATAAGCAAACTGGCATCACGCCAAT

GGCAACCGATACCGTCCTGACCCATGACGCGATCACGCCGACATTATTAAAGCTGTT

>Providencia_stuartii_mcr-1_22

CGCGACCGCCAATCTTACCTTTTTTGATAAAATCAGCCAAACCTATCCCATCGCGGACAATCTCGGCTTTGTGCTGACGA

TCGCTGTCGTGCTCTTTGGCGCGATGCTACTGATCACCACGCTGTTATCATCGTATCGCTATGTGCTAAAGCCTGTGTTG

ATTTTGCTATTAATCATGGGCGCGGTGACCAGTTATTTTACTGACACTTATGGCACGGTCTATGATACGACCATGCTCCA

AAATGCCCTACAGACCGACCAAGCCGAGACCAAGGATCTATTAAACGCAGCGTTTATCATGCGTATCATTGGTTTGGGTG

TGCTACCAAGTTTGCTTGTGGCTTTTGTTAAGGTGGATTATCCGACTTGGGGCAAGGGTTTGATGCGCCGATTGGGCTTG

ATCGTGGCAAGTCTTGCGCTGATTTTACTGCCTGTGGTGGCGTTCAGCAGTCATTATGCCAGTTTCTTTCGCGTGCATAA

GCCGCTGCGTAGCTATGTCAATCCGATCATGCCAATCTACTCGGTGGGTAAGCTTGCCAGTATTGAGTATAAAAAAGCCA

GTGCGCCAAAAGATACCATTTATCACGCCAAAGACGCGGTACAAGCAACCAAGCCTGATATGCGTAAGCCACGCCTAGTG

GTGTTCGTCGTCGGTGAGACGGCACGCGCCGATCATGTCAGCTTCAATGGCTATGAGCGCGATACTTTCCCACAGCTTGC

CAAGATCGATGGCGTGACCAATTTTAGCAATGTCACATCGTGCGGCACATCGACGGCGTATTCTGTGCCGTGTATGTTCA

GCTATCTGGGCGCGGATGAGTATGATGTCGATACCGCCAAATACCAAGAAAATGTGCTGGATACGCTGGATCGCTTGGGC

GTAAGTATCTTGTGGCGTGATAATAATTCGGACTCAAAAGGCGTGATGGATAAGCTGCCAAAAGCGCAATTTGCCGATTA

TAAATCCGCGACCAACAACGCCATCTGCAACACCAATCCTTATAACGAATGCCGCGATGTCGGTATGCTCGTTGGCTTAG

ATGACTTTGTCGCTGCCAATAACGGCAAAGATATGCTGATCATGCTGCACCAAATGGGCAATCACGGGCCTGCGTATTTT

AAGCGATATGATGAAAAGTTTGCCAAATTCACGCCAGTGTGTGAAGGTAATGAGCTTGCCAAGTGCGAACATCAGTCCTT

GATCAATGCTTATGACAATGCCTTGCTTGCCACCGATGATTTCATCGCTCAAAGTATCCAGTGGCTGCAGACGCACAGCA

ATGCCTATGATGTCTCAATGCTGTATGTCAGCGATCATGGCGAAAGTCTGGGTGAGAACGGTGTCTATCTACATGGTATG

CCAAATGCCTTTGCACCAAAAGAACAGCGCAGTGTGCCTGCATTTTTCTGGACGGATAAGCAAACTGGCATCACGCCAAT

GGCAACCGATACCGTCCTGACCCATGACGCGATCACGCCGACATTATTAAAGCTGTT

>Salmonella_enterica_MCR-1_23

TAATACCCCACCGTTTATTTTTTGAGTAGTTTCTCATGATGCAGCATACTTCTGTGTGGTACCGACGCTCGGTCAGTCCG

TTTGTTCTTGTGGCGAGTGTTGCCGTTTTCTTGACCGCGACCGCCAATCTTACCTTTTTTGATAAAATCAGCCAAACCTA

TCCCATCGCGGACAATCTCGGCTTTGTGCTGACGATCGCTGTCGTGCTCTTTGGCGCGATGCTACTGATCACCACGCTGT

TATCATCGTATCGCTATGTGCTAAAGCCTGTGTTGATTTTGCTATTAATCATGGGCGCGGTGACCAGTTATTTTACTGAC

ACTTATGGCACGGTCTATGATACGACCATGCTCCAAAATGCCCTACAGACCGACCAAGCCGAGACCAAGGATCTATTAAA

CGCAGCGTTTATCATGCGTATCATTGGTTTGGGTGTGCTACCAAGTTTGCTTGTGGCTTTTGTTAAGGTGGATTATCCGA

CTTGGGGCAAGGGTTTGATGCGCCGATTGGGCTTGATCGTGGCAAGTCTTGCGCTGATTTTACTGCCTGTGGTGGCGTTC

AGCAGTCATTATGCCAGTTTCTTTCGCGTGCATAAGCCGCTGCGTAGCTATGTCAATCCGATCATGCCAATCTACTCGGT

GGGTAAGCTTGCCAGTATTGAGTATAAAAAAGCCAGTGCGCCAAAAGATACCA

>Uncultured_bacterium_MCR-2.4_24

ATGACATCACATCACTCTTGGTATCGCTACTCCATCAATCCTTTTGTACTGATGGGTTTGGTGGCGTTATTTTTGGCGGC

AACAGCGAACCTGACATTTTTTGAAAAAGCGATGGCGGTCTATCCTGTATCGGATAACTTAGGCTTTATCATCTCAATGG

CGGTTGCACTGATGGGTGCTATGCTATTGATTGTCGTTCTGTTTTCCTATCGTTATGTGCTCAAGCCTGTGCTGATTTTG

CTTTTGATGATGGGTGCGGTAACCAGTTATTTTACCGATACTTATGGCACAGTCTATGATACGACCATGCTACAAAATGC

CCTGCAAACCGACCAAGCTGAGTCCAAGGACTTGATGAATTTGGCGTTTTTTGTGCGGATTATTGGGCTTGGTGTTTTGC

CGAGCATAGTGGTGGCGTGGGTCAAGGTGGATTATCCAACATGGGGCAAAGGCCTGATTCAGCGTGCGATGACGTGGGGT

GTCAGCCTTGTGCTGTTGCTTGTGCCGATTGGGTTATTTAGCAGTCAGTATGCGAGTTTCTTTCGGGTGCATAAGCCAGT

GCGTTTTTATATCAACCCGATTACGCCGATTTATTCGGTGGGTAAGCTTGCCAGTATCGAGTACAAAAAAGCCACTGCGC

CAAAAGACACCATCTATCATGCCAAAGATGCCGTGCAGACCACCAAGCCGAGCGAGCGTAAGCCACGCCTAGTGGTGTTC

GTCGTCGGTGAGACGGTGCGCGCTGACCATGTGCAGTTCAATGGCTATAGCCGTGAGACTTTCCCGCAGCTTGCCAAAGT

TGATGGCTTGGCGAACTTTAGCCAAGTGACATCGTGTGGCACATCGACGGCGTATTCTGTGCCGTGTATGTTCAGCTATC

TGGGTCAAGATGACTATGATGTCGATACCGCCAAATACCAAGAAAATGTCCTAGATACGCTTGACCGCTTGGGCGTGGAT

ATCTTGTGGCGTGATAATAATTCAGACTCAAAAGGCGTGATGGATAAGCTGCCTGCCACGCAGTATTTTGATTATAAATC

AGCGACCAACAACACCATCTGCAACACCAATCCCTATAACGAATGTCGTGATGTCGGTATGCTTGTTGGGCTAGACGACT

ATGTCAGCGCCAATAATGGCAAAGATATGCTTATCATGCTACACCAAATGGGCAATCATGGGCCGGCGTACTTTAAGCGT

TATGATGAGCAATTTGCCAAATTCACCCCTGTGTGCGAAGGCAATGAGCTTGCCAAATGCGAACATCAATCGCTCATCAA

TGCCTATGATAATGCACTACTTGCCACCGATGATTTTATCGCCAAAAGTATCGATTGGCTAAAAACACATGAAGCAAACT

ACGATGTCGCCATGCTCTATGTCAGCGACCACGGCGAGAGCTTGGGCGAAAATGGTGTCTATTTGCATGGTATGCCAAAT

GCCTTTGCACCAAAAGAACAGCGTGCCGTACCTGCGTTTTTTTGGTCAAATAATACATCGTTCAAGCCAACTGCCAGCGA

TACTGCATTGACCCATGATGCAATTACGCCGACACTGCTTAAGCTGTTTGATGTCACAGCGGGCAAGGTCAAAGACCGCA

CGGCATTTATCCAGTAA

>Moraxella_pluranimalium_mcr-2.2_25

ATGACATCACAGCACTCTTGGTATCGCTACTCCATCAATCCTTTTGTACTGATGGGTTTGGTGGCGTTATTTTTGGCGGC

AACAGCGAACCTGACATTTTTTGAAAAAGCGATGGCGGTCTATCCTGTATCGGATAACTTAGGCTTTATCATCTCAATGG

CGGTTGCACTGATGGGTGCTATGCTATTGATTGTCGTGCTATTATCCTATCGCTATGTGCTAAAGCCTGTGCTGATTTTA

TTACTTATCATGGGTGCGGTGACGAGCTATTTTACCGATACTTATGGCACGGTCTATGATACCACCATGCTCCAAAATGC

CATGCAAACCGACCAAGCTGAATCTAAAGACTTGATGAATTTGGCGTTTTTTGTGCGGATTATCGGGCTTGGCGTGTTGC

CAAGTGTGTTGGTCGCATTTGCCAAAGTCAATTATCCAACATGGGGCAAAGGCCTGATTCAGCGTGCGATGACGTGGGGT

GTCAGCCTTGTGCTGTTGCTTGTGCCGATTGGGCTATTTAGCAGTCAGTATGCGAGTTTCTTTCGGGTGCATAAGCCAGT

GCGTTTTTATATCAATCCGATTACGCCGATTTATTCGGTGGGCAAGCTTGCCAGTATCGAGTACAAAAAAGCCACTGCAC

CAACAGACACCATCTATCATGCCAAAGATGCCGTGCAGACCACCAAGCCTAGCGAGCGTAAGCCACGCCTAGTAGTGTTC

GTCGTCGGTGAGACGGCGCGTGCTGACCATGTGCAGTTCAATGGCTATGGCCGTGAGACTTTCCCACAGCTTGCCAAAGT

TGATGGCTTGGCGAATTTTAGCCAAGTGACATCGTGTGGCACATCGACAGCGTATTCTGTGCCGTGTATGTTTAGCTATT

TGGGTCAAGATGACTATGATGTCGATACCGCCAAATACCAAGAAAATGTGCTAGATACGCTTGACCGCTTGGGCGTGGAT

ATCTTGTGGCGTGATAATAATTCAGACTCAAAAGGCGTGATGGATAAGCTACCTACCACGCAGTATTTTGATTATAAATC

AGCGACCAACAACACCATCTGTAACACCAATCCCTTTAATGAATGCCGTGATGTCGGTATGCTTGTTGGGCTAGATGACT

ATGTCAGTGCCAATAATGGCAAAGATATGCTCATCATGCTACACCAAATGGGCAATCATGGGCCGGCGTACTTTAAGCGT

TATGATGAGCAATTTGCCAAATTCACCCCTGTGTGCGAAGGCAATGAGCTTGCCAAATGCGAACACCAATCACTCATCAA

TGCCTATGATAATGCACTACTTGCCACCGATGATTTTATCGCCAAAAGTATCGATTGGCTAAAAACACATGAAGCAAACT

ACGATGTCGCTATGCTCTATGTCAGCGACCACGGCGAGAGCTTGGGCGAGAATGGTGTCTATCTGCATGGTATGCCAAAT

GCCTTTGCACCAAAAGAACAGCGAGCCGTGCCTGCGTTTTTTTGGTCAAATAATACGACATTCAAGCCAACTGCCAGCGA

CACTGTGCTGACGCATGATGCGATTACCCCGACATTGCTTAAGCTGTTTGATGTCACAGCCGACAAGGTCAAAGACCGCA

CGGCATTTATCCAGTAA

>Moraxella_pluranimalium_MCR-2.2_26

TGCACTATTTTATTTATCTGCACAATATATCTAGATTCTCCAACGACATTGAAGTATAATCGCCAACTTGTATTGTATAT

GGCATTTGTGGGTAATCTTTATGACATCACAGCACTCTTGGTATCGCTACTCCATCAATCCTTTTGTACTGATGGGTTTG

GTGGCGTTATTTTTGGCGGCAACAGCGAACCTGACATTTTTTGAAAAAGCGATGGCGGTCTATCCTGTATCGGATAACTT

AGGCTTTATCATCTCAATGGCGGTTGCACTGATGGGTGCTATGCTATTGATTGTCGTGCTATTATCCTATCGCTATGTGC

TAAAGCCTGTGCTGATTTTATTACTTATCATGGGTGCGGTGACGAGCTATTTTACCGATACTTATGGCACGGTCTATGAT

ACCACCATGCTCCAAAATGCCATGCAAACCGACCAAGCTGAATCTAAAGACTTGATGAATTTGGCGTTTTTTGTGCGGAT

TATCGGGCTTGGCGTGTTGCCAAGTGTGTTGGTCGCATTTGCCAAAGTCAATTATCCAACATGGGGCAAAGGCCTGATTC

AGCGTGCGATGACGTGGGGTGTCAGCCTTGTGCTGTTGCTTGTGCCGATTGGGCTATTTAGCAGTCAGTATGCGAGTTTC

TTTCGGGTGCATAAGCCAGTGCGTTTTTATATCAATCCGATTACGCCGATTTATTCGGTGGGCAAGCTTGCCAGTATCGA

GTACAAAAAAGCCACTGCACCAACAGACACCATCTATCATGCCAAAGATGCCGTGCAGACCACCAAGCCTAGCGAGCGTA

AGCCACGCCTAGTAGTGTTCGTCGTCGGTGAGACGGCGCGTGCTGACCATGTGCAGTTCAATGGCTATGGCCGTGAGACT

TTCCCACAGCTTGCCAAAGTTGATGGCTTGGCGAATTTTAGCCAAGTGACATCGTGTGGCACATCGACAGCGTATTCTGT

GCCGTGTATGTTTAGCTATTTGGGTCAAGATGACTATGATGTCGATACCGCCAAATACCAAGAAAATGTGCTAGATACGC

TTGACCGCTTGGGCGTGGATATCTTGTGGCGTGATAATAATTCAGACTCAAAAGGCGTGATGGATAAGCTACCTACCACG

CAGTATTTTGATTATAAATCAGCGACCAACAACACCATCTGTAACACCAATCCCTTTAATGAATGCCGTGATGTCGGTAT

GCTTGTTGGGCTAGATGACTATGTCAGTGCCAATAATGGCAAAGATATGCTCATCATGCTACACCAAATGGGCAATCATG

GGCCGGCGTACTTTAAGCGTTATGATGAGCAATTTGCCAAATTCACCCCTGTGTGCGAAGGCAATGAGCTTGCCAAATGC

GAACACCAATCACTCATCAATGCCTATGATAATGCACTACTTGCCACCGATGATTTTATCGCCAAAAGTATCGATTGGCT

AAAAACACATGAAGCAAACTACGATGTCGCTATGCTCTATGTCAGCGACCACGGCGAGAGCTTGGGCGAGAATGGTGTCT

ATCTGCATGGTATGCCAAATGCCTTTGCACCAAAAGAACAGCGAGCCGTGCCTGCGTTTTTTTGGTCAAATAATACGACA

TTCAAGCCAACTGCCAGCGACACTGTGCTGACGCATGATGCGATTACCCCGACATTGCTTAAGCTGTTTGATGTCACAGC

CGACAAGGTCAAAGACCGCACGGCATTTATCCAGTAAGTTATCAGCATCACCCAATCCCAAGCCATCATACTACATCATC

GTGTGATGGCAAAGGGCGGTTTTGCAATGTGTTTTGCAATGTTTTTTTAGGAATGTA

>Moraxella_pluranimalium_mcr-2_27

ATGACATCACAGCACTCTTGGTATCGCTACTCCATCAATCCTTTTGTACTGATGGGTTTGGTGGCGTTATTTTTGGCGGC

AACAGCGAACCTGACATTTTTTGAAAAAGCGATGGCGGTCTATCCTGTATCGGATAACTTAGGCTTTATCATCTCAATGG

CGGTTGCACTGATGGGTGCTATGCTATTGATTGTCGTGCTATTATCCTATCGCTATGTGCTAAAGCCTGTGCTGATTTTA

TTACTTATCATGGGTGCGGTGACGAGCTATTTTACCGATACTTATGGCACGGTCTATGATACCACCATGCTCCAAAATGC

CATGCAAACCGACCAAGCTGAATCTAAAGACTTGATGAATTTGGCGTTTTTTGTGCGGATTATCGGGCTTGGCGTGTTGC

CAAGTGTGTTGGTCGCATTTGCCAAAGTCAATTATCCAACATGGGGCAAAGGCCTGATTCAGCGTGCGATGACGTGGGGT

GTCAGCCTTGTGCTGTTGCTTGTGCCGATTGGGCTATTTAGCAGTCAGTATGCGAGTTTCTTTCGGGTGCATAAGCCAGT

GCGTTTTTATATCAATCCGATTACGCCGATTTATTCGGTGGGCAAGCTTGCCAGTATCGAGTACAAAAAAGCCACTGCAC

CAACAGACACCATCTATCATGCCAAAGATGCCGTGCAGACCACCAAGCCTAGCGAGCGTAAGCCACGCCTAGTAGTGTTC

GTCGTCGGTGAGACGGCGCGTGCTGACCATGTGCAGTTCAATGGCTATGGCCGTGAGACTTTCCCACAGCTTGCCAAAGT

TGATGGCTTGGCGAATTTTAGCCAAGTGACATCGTGTGGCACATCGACAGCGTATTCTGTGCCGTGTATGTTTAGCTATT

TGGGTCAAGATGACTATGATGTCGATACCGCCAAATACCAAGAAAATGTGCTAGATACGCTTGACCGCTTGGGCGTGGAT

ATCTTGTGGCGTGATAATAATTCAGACTCAAAAGGCGTGATGGATAAGCTACCTACCACGCAGTATTTTGATTATAAATC

AGCGACCAACAACACCATCTGTAACACCAATCCCTTTAATGAATGCCGTGATGTCGGTATGCTTGTTGGGCTAGATGACT

ATGTCAGTGCCAATAATGGCAAAGATATGCTCATCATGCTACACCAAATGGGCAATCATGGGCCGGCGTACTTTAAGCGT

TATGATGAGCAATTTGCCAAATTCACCCCTGTGTGCGAAGGCAATGAGCTTGCCAAATGCGAACACCAATCACTCATCAA

TGCCTATGATAATGCACTACTTGCCACCGATGATTTTATCGCCAAAAGTATCGATTGGCTAAAAACACATGAAGCAAACT

ACGATGTCGCTATGCTCTATGTCAGCGACCACGGCGAGAGCTTGGGCGAGAATGGTGTCTATCTGCATGGTATGCCAAAT

GCCTTTGCACCAAAAGAACAGCGAGCCGTGCCTGCGTTTTTTTGGTCAAATAATACGACATTCAAGCCAACTGCCAGCGA

CACTGTGCTGACGCATGATGCGATTACCCCGACATTGCTTAAGCTGTTTGATGTCACAGCCGACAAGGTCAAAGACCGCA

CGGCATTTATCCAGTAA

>Uncultured_bacterium_MCR-2.5_28

ATGACATCACAGCACTCTTGGTATCGCTACTCCATCAATCCTTTTGTACTGATGGGTTTGGTGGCGTTTTTTTTGGCAGC

GACAGCGAACCTGACATTTTTTGAAAAAGCGATGGCGGTCTATCCTGTATCGGATAACTTAGGCTTTATCGTCTCAATGG

CGGTTGCGCTGATGGGTGCTATGCTATTGATTGTCGTGCTATTATCCTATCGCTATGTGCTAAAGCCTGTCCTGATTTTG

CTACTGATTATGGGTGCGGTGACGAGCTATTTTACCGATACTTATGGCACGGTCTATGATACCACCATGCTCCAAAATGC

CATGCAAACCGACCAAGCCGAGTCTAAAGACTTGATGAATTTGGCGTTTTTTGTGCGGATTATCGGGCTTGGTGTGTTGC

CAAGTCTATTGGTCGCAGTTGCCAAAGTTAATTATCCAACATGGGGCAAAAGCCTGATTCAGCGTGCGATGACGTGGGGT

GTCAGCCTTGTGCTGTTGCTTGTGCCGATTGGACTATTTAGCAGTCAGTATGCGAGTTTCTTTCGGGTGCATAAGCCAGT

GCGTTTTTATATCAATCCGATTACGCCGATTTATTCGGTGGGTAAGCTTGCCAGTATCGAGTACAAAAAAGCCACTGCGC

CGACAGACACCATCTATCATGCCAAAGATGCCGTGCAGACCACCAAGCCTAGCGAGCGTAAGCCACGCCTAGTGGTGTTC

GTCGTCGGTGAGACGGCTCGTGCTGACCATGTGCAGTTCAATGGCTATGGCCGTGAGACTTTCCCACAGCTTGCTAAGGT

CGATGGCTTGGCGAATTTTAGCCAAGTGACATCGTGTGGCACATCGACGGCGTATTCTGTGCCGTGTATGTTCAGCTATC

TGGGTCAAGATGACTATGATGTCGATACCGCCAAATACCAAGAAAATGTGCTAGATACGCTTGACCGCTTGGGCGTGGGT

ATCTTGTGGCGTGATAATAATTCAGACTCAAAAGGCGTGATGGATAAGCTACCTGCCACGCAGTATTTTGATTATAAATC

AGCAACCAACAACACCATCTGTAACACCAACCCTTATAATGAATGTCGTGATGTCGGTATGCTTGTTGGGCTAGATGACT

ATGTCAGCACCAATAATGGTAAAGATATGCTCATCATGCTACACCAAATGGGCAATCATGGGCCGGCGTACTTTAAGCGT

TATGATGAGCAATTTGCCAAATTCACCCCCGTGTGCGAAGGCAACGAGCTTGCCAAATGCGAACATCAATCGCTCATCAA

TGCCTATGATAATGCGCTACTTGCCACCGATGATTTTATCGCCAAAAGTATCGATTGGCTAAAAACACATGAAGCAAACT

ACGATGTCGCCATGCTCTATGTCAGCGACCACGGCGAGAGTTTGGGCGAGAATGGCGTCTATTTGCATGGTATGCCAAAT

GCCTTTGCACCAAAAGAACAGCGAGCCGTGCCTGCGTTTTTTTGGTCAAATAATACGACATTCAAGCCAACTGCCAGCGA

CACTGTGCTGACGCATGATGCGATTACGCCAACACTGCTTAAGCTGTTTGATGTCACAGCGGGCAAGGTCAAAGACCGCA

CGGCATTTATCCAGTAA

>Uncultured_bacterium_MCR-2.7_29

ATGACATCACAGCACTCTTGGTATCGCTACTCCATCAATCCTTTTGTACTGATGGGTTTGGTGGCGTTTTTTTGGCAGC

GACAGCGAACCTGACATTTTTTGAAAAAGCGATGGCGGTCTATCCTGTATCGGATAACTTAGGCTTTATCGTCTCAATGG

CGGTTGCGCTGATGGGTGCTATGCTATTGATTGTCGTGCTATTATCCTATCGCTATGTGCTAAAGCCTGTCCTGATTTTG

CTACTGATTATGGGTGCGGTGACGAGCTATTTTACCGATACTTATGGCACGGTCTATGATACCACCATGCTCCAAAATGC

CATGCAAACCGACCAAGCCGAGTCTAAAGACTTGATGAATTTGGCGTTTTTTGTGCGGATTATCGGGCTTGGTGTGTTGC

CAAGTGTGTTGGTCGCAGTTGCCAAAGTCAATTATCCAACATGGGGCAAAGGCCTGATTCAGCGTGCGATGACGTGGGGT

GTCAGCCTTGTGCTGTTGCTTGTGCCGATTGGACTATTTAGCAGTCAGTATGCGAGTTTCTTTCGGGTGCATAAGCCAGT

GCGTTTTTATATTAACCCGATTACGCCGATTTATTCGGTGGGTAAGCTTGCCAGTATCGAGTACAAAAAAGCCACTGCGC

CAACAGACACTATCTATCATGCCAAAGATGCCGTGCAGACCACCAAGCCGAGCGAGCGTAAGCCACGCCTAGTGGTGTTC

GTCGTCGGTGAGACGGCTCGTGCTGACCATGTGCAGTTCAATGGCTATGGCCGCGAGACTTTCCCACAGCTTGCCAAAGT

TGACGGCTTGGCGAACTTTAGCCAAGTGACATCGTGTGGCACATCGACGGCGTATTCTGTGCCGTGTATGTTTAGCTATC

TGGGTCAAGATGACTATGATGTCGATACCGCCAAATACCAAGAAAATGTGCTGGATACGCTTGACCGCTTGGGCGTGGGC

ATCTTGTGGCGTGATAATAATTCAGACTCAAAAGGTGTGATGGATAAGCTACCTGCCACGCAGTATTTTGATTATAAATC

AGCGACCAACAACACCATCTGTAGCACCAACCCCTATAATGAATGTCGTGATGTCGGTATGCTTGTCGGGCTAGATGACT

ATGTCAGCGCCAATAATGGCAAAGATATGCTCATCATGCTACACCAAATGGGCAATCATGGGCCGGCGTACTTTAAGCGT

TATGATGAGCAATTTGCCAAATTCACCCCTGTGTGCGAAGGCAACGAGCTTGCCAAATGCGAACATCAATCGCTCATCAA

TGCCTATGATAATGCGCTACTTGCCACCGATGATTTTATCGCCAAAAGTATCGATTGGCTAAAAACACATGAAGCGAACT

ACGATGTCGCCATGCTGTATGTCAGCGACCACGGCGAGAGCTTGGGTGAGAATGGCGTCTATCTGCATGGTATGCCAAAT

GCCTTTGCACCAAAAGAACAGCGAGCCGTGCCTGCGTTTTTTTGGTCAAATAATACATCGTTCAAGCCAACTGCCAGCGA

TACTGTGCTGACTCATGATGCGATTACGCCGACACTGCTTAAGCTGTTTGATGTCACAGCCGACAAGGTCAAAGACCGCA

CGGCATTTATCCAGTAA

>Moraxella_sp._MCR-6.1_30

ATGACACAGCATAGTCCTTGGTACCGCCGTCCGGTCAATCCCTATCTGTTGATGAGCGTGGTCGCTTTATTTTTGTCAGC

GACAGCAAACCTAACTTTCTTTGATAAAATCACCAATACTTATCCGATGGCACAAAACGCAGGCTTTGTGATCTCAACGG

CGCTTGTGCTATTTGGGGCGATGCTATTGATTACTGTGCTGTTATCGTATCGCTATGTGCTTAAGCCTGTGTTGATTTTG

CTGCTTATCATGGGTGCGGTGACGAGCTATTTTACCGATACTTATGGCACCGTTTATGACACCACCATGCTCCAAAATGC

CTTGCAAACTGACCAAGCCGAGTCTAAGGACTTGATGAATATGGCGTTTTTTGTGCGGATTATCGGGCTTGGCGTGTTGC

CAAGTATCTTGGTGGCGTGGGTCAAGGTGGATTATCCGACATTGGGTAAGAGTCTGATTCAGCGTGCGATGACTTGGGGT

GTGGCAGTGGTGATGGCACTTGTGCCGATTTTGGCATTTAGTAGTCACTACGCCAGTTTCTTTCGTGAACATAAGCCACT

GCGTAGCTATGTCAATCCCGTGATGCCGATTTATTCAGTAGGTAAGCTTGCCAGTATTGAGTACAAAAAAGCCACCGCGC

CAAAAGACACCATCTATCATGCCAAAGATGCTGTACAGACGACGACGCCTGCCGAGCGTAAGCCACGACTCGTGGTGTTC

GTCGTCGGTGAGACGGCTCGAGCTGACCATGTGCAGTTTAATGGCTATAGTCGTGAGACTTTTCCGCAGCTTGCCAAGAT

TGACAACCTAGCCAATTTTAGCCAAGTGACATCGTGTGGCACATCGACGGCGTACTCTGTGCCGTGTATGTTCAGTTATC

TGGGTCAAGATGACTATGATGTCGATACCGCCAAATACCAAGAAAACGTGCTGGATACGCTTGACCGACTGGGTGTGGGT

ATCCTGTGGCGGGATAATAATTCAGACTCAAAAGGCGTGATGGATAAACTGCCTGCTTCGCAGTATTTTGATTATAAATC

AGCGACCAACAACACCATCTGTAACACCAATCCTTACAACGAATGTCGTGATGTCGGTATGTTGGTGGGGCTAGATGATT

ATGTGAGTACCAATCAAGGCAAAGATATGCTCATCATGCTACACCAAATGGGTAATCATGGGCCGGCGTACTTCAAGCGT

TATGACGAGCAATTTGCCAAATACACCCCTGTGTGCGAAGGTAATGAACTTGCCAAGTGTGAACACCAATCGCTCATCAA

CGCCTATGATAATGCACTGCTTGCGACCGATGATTTTATCGCCAAAAGTATCGATTGGCTAAAAACGCATCAGGCCAACT

ATGATGTTGCCATGCTCTATGTCAGCGACCACGGCGAGAGTCTGGGTGAAAATGGCGTCTATCTGCATGGTATGCCAAAT

GCCTTTGCACCAAAAGAACAGCGAGCGGTACCGGCATTCTTTTGGTCAAATAATCCATCGTTCACGCCAACTGCCAGCGA

CACTGTGCTGACACATGATGCGATTACGCCGACTCTACTGAAGCTGTTTGATGTCACAGCGGATAAGGTCAAAGACCGCA

CCGCATTCATCCGCTGA

>Moraxella_sp._mcr-6_31

ATGACACAGCATAGTCCTTGGTACCGCCGTCCGGTCAATCCCTATCTGTTGATGAGCGTGGTCGCTTTATTTTTGTCAGC

GACAGCAAACCTAACTTTCTTTGATAAAATCACCAATACTTATCCGATGGCACAAAACGCAGGCTTTGTGATCTCAACGG

CGCTTGTGCTATTTGGGGCGATGCTATTGATTACTGTGCTGTTATCGTATCGCTATGTGCTTAAGCCTGTGTTGATTTTG

CTGCTTATCATGGGTGCGGTGACGAGCTATTTTACCGATACTTATGGCACCGTTTATGACACCACCATGCTCCAAAATGC

CTTGCAAACTGACCAAGCCGAGTCTAAGGACTTGATGAATATGGCGTTTTTTGTGCGGATTATCGGGCTTGGCGTGTTGC

CAAGTATCTTGGTGGCGTGGGTCAAGGTGGATTATCCGACATTGGGTAAGAGTCTGATTCAGCGTGCGATGACTTGGGGT

GTGGCAGTGGTGATGGCACTTGTGCCGATTTTGGCATTTAGTAGTCACTACGCCAGTTTCTTTCGTGAACATAAGCCACT

GCGTAGCTATGTCAATCCCGTGATGCCGATTTATTCAGTAGGTAAGCTTGCCAGTATTGAGTACAAAAAAGCCACCGCGC

CAAAAGACACCATCTATCATGCCAAAGATGCTGTACAGACGACGACGCCTGCCGAGCGTAAGCCACGACTCGTGGTGTTC

GTCGTCGGTGAGACGGCTCGAGCTGACCATGTGCAGTTTAATGGCTATAGTCGTGAGACTTTTCCGCAGCTTGCCAAGAT

TGACAACCTAGCCAATTTTAGCCAAGTGACATCGTGTGGCACATCGACGGCGTACTCTGTGCCGTGTATGTTCAGTTATC

TGGGTCAAGATGACTATGATGTCGATACCGCCAAATACCAAGAAAACGTGCTGGATACGCTTGACCGACTGGGTGTGGGT

ATCCTGTGGCGGGATAATAATTCAGACTCAAAAGGCGTGATGGATAAACTGCCTGCTTCGCAGTATTTTGATTATAAATC

AGCGACCAACAACACCATCTGTAACACCAATCCTTACAACGAATGTCGTGATGTCGGTATGTTGGTGGGGCTAGATGATT

ATGTGAGTACCAATCAAGGCAAAGATATGCTCATCATGCTACACCAAATGGGTAATCATGGGCCGGCGTACTTCAAGCGT

TATGACGAGCAATTTGCCAAATACACCCCTGTGTGCGAAGGTAATGAACTTGCCAAGTGTGAACACCAATCGCTCATCAA

CGCCTATGATAATGCACTGCTTGCGACCGATGATTTTATCGCCAAAAGTATCGATTGGCTAAAAACGCATCAGGCCAACT

ATGATGTTGCCATGCTCTATGTCAGCGACCACGGCGAGAGTCTGGGTGAAAATGGCGTCTATCTGCATGGTATGCCAAAT

GCCTTTGCACCAAAAGAACAGCGAGCGGTACCGGCATTCTTTTGGTCAAATAATCCATCGTTCACGCCAACTGCCAGCGA

CACTGTGCTGACACATGATGCGATTACGCCGACTCTACTGAAGCTGTTTGATGTCACAGCGGATAAGGTCAAAGACCGCA

CCGCATTCATCCGCTGA

>Uncultured_bacterium_MCR-2.6_32

ATGACATCACAGCACTCTTGGTATCGCTACTCCATCAATCCCTTTGTACTGATGGGTTTGGTGGCGTTATTTTTGGCAGC

GACAGCGAACCTGGCATTTTTTGAAAAAGCGATGGCGGTCTATCCTGTATCGGATAACTTAGGCTTTATCATCTCAATGG

CGGTTGCACTGATGGGTGCTATGCTATTGATTGTCGTGCTGTTATCCTATCGCTATGTGCTAAAGCCTGTGCTGATTTTG

CTACTGATTATGGGTGCAGTGACGAGCTATTTTACCGATACTTATGGCACGGTCTATGACACCACCATGCTCCAAAATGC

CATGCAAACCGACCAAGCCGAGTCTAAGGACTTGATGAATTTGGCGTTTTTTGTGCGGATTATCGGGCTTGGTGTGTTGC

CAAGTGTGTTGGTCGCAGTTGCCAAAGTCAATTATCCAACATGGGGCAAAGGCCTGATTCAGCGTGCGATGACGTGGGGT

GTCAGCCTTGCGCTGTTGCTTGTGCCGATTGGGCTATTTAGCAGCCAGTATGCGAGTTTCTTTCGTGTGCATAAGCCAGT

GCGTTTTTATATCAACCCGATTACGCCGATTTATTCGGTGGGCAAGCTTGCCAGTATCGAGTACAAAAAAGCCACTGCAC

CAACAGACACCATCTATCATGCCAAAGATGCCGTACAGACCACCAAGCCTAGCGAGCGTAAGCCCCGTCTAGTGGTGTTC

GTCGTCGGTGAGACGGCGCGTGCTGACCATGTGCAGTTCAATGGCTATGGCCGTGAGACTTTCCCACAGCTTGCTAAAGT

TGATGGCTTGGCGAACTTTAGCCAAGTGACATCGTGTGGCACATCGACAGCGTATTCTGTGCCGTGTATGTTTAGCTATT

TGGGTCAAGATGACTATGATGTCGATACCGCCAAATACCAAGAAAATGTGCTAGATACGCTTGACCGCTTGGGCGTGGGT

ATCTTGTGGCGTGATAATAATTCAGACTCAAAAGGTGTGATGGATAAGCTACCTGCCACGCAGTATTTTGATTATAAATC

AGCGACCAACAACACCATCTGTAACACCAATCCTTATAACGAATGTCGTGATGTTGGTATGCTTGTCGGGCTAGATGACT

ATGTCAGCGCCAATAATGGCAAAGATATGCTCATCATGCTACACCAAATGGGCAATCATGGGCCGGCGTACTTTAAGCGT

TATGATGAGCAATTTGCCAAATTCACCCCTGTGTGCGAAGGCAACGAGCTTGCCAAATGCGAACATCAATCGCTCATCAA

TGCCTATGATAATGCGCTACTTGCCACCGATGATTTTATCGCCAAAAGTATCGATTGGCTAAAAACACATGAAGCGAACT

ATGATGTCGCCATGCTCTATGTCAGCGACCACGGCGAGAGCTTGGGTGAGAATGGCGTCTATTTGCACGGTATGCCAAAT

GCCTTTGCGCCAAAAGAACAGCGAGCCGTACCTGCATTTTTTTGGTCAAATAATACGACATTTAAGCCAACTGCCAGCGA

TACTGCATTGACCCATGATGCGATTACGCCGACACTGCTTAAGCTGTTTGATGTCACAGCCGACAAGGTCAAAGACCGCA

CGGCATTTATCCAGTAA

>Uncultured_bacterium_33

TTTCTCATGATGCAGCATACTTCTGTGTGGTACCGACGCTCGGTCAGTCCGTTTGTTCTTGTGGCGAGTGTTGCCGTTTT

CTTGACCGCGACCGCCAATCTTACCTTTTTTGATAAAATCAGCCAAACCTATCCCATCGCGGACAATCTCGGCTTTATGC

TGACGATCGCTGTCGTGCTCTTTGGCGCGATGCTACTGATCACCACGCTGTTATCATCGTATCGCTATGTGCTAAAGCCT

GTGTTGATTTTGCTATTAATCATGGGCGCGGTGACCAGTTATTTTACTGACACTTATGGCACGGTCTATGATACGACCAT

GCTCCAAAATGCCCTACAGACCGACCAAGCCGAGACCAAGGATCTATTAAACGCAGCGTTTATCATGCGTATCATTGGTT
TGGGTGTGCTACCAAGTTTGCTTGTGGCTTTTGTTAAGGTGGATTATCCGACTTGGGGCAAGGGTTTGATGCGCCGATTG
GGCTTGATCGTGGCAAGTCTTGCGCTGATTTTACTGCCTGTGGTGGCGTTCAGCAGTCATTATGCCAGTTTCTTTCGCGT
GCATAAGCCGCTGCGTAGCTATGTCAATCCGATCAT

>Uncultured_bacterium_34
CAGCATACTTCTGTGTGGTACCGACGCTCGGTCAGTCCGTTTGTTCTTGTGGCGAGTGTTGCCGTTTTCTTGACCGCGAC
CGCCAATCTTACCTTTTTTGATAAAATCAGCCAAACCTATCCCATCGCGGACAATCTCGGCTTTGTGCTGACGATCGCTG
TCGTGCTCTTTGGCGCGATGCTACTGATCACCACGCTGTTATCATCGTATCGCTATGTGCTAAAGCCTGTGTTGATTTTG
CTATTAATCATGGGCGCGGTGACCAGTTATTTTACTGACACTTATGGCACGGTCTATGATACGACCATGCTCCAAAATGC
CCTACAGACCGACCAAGCCGAGACCAAGGATCTATTAAACGCAGCGTTTATCATGCGTATCATTGGTTTGGGTGTGCTAC
CAAGTTTGCTTGTGGCTTTTGTTAAGGTGGATTATCCGACTTGGGGCAAGGGTTTGATGCGCCGATTGGGCTTGATCGTG
GCAAGTCTTGCGCTGATTTTACTGCCTGTGGTGGCGTTCAGCAGTCATTATGCCAGTTTCTTTCGCGTGCATAAGCCGCT
GCGTAGCTATGTCA

>Uncultured_bacterium_35
CAGCATACTTCTGTGTGGTACCGACGCTCGGTCAGTCCGTTTGTTCTTGTGGCGAGTGTTGCCGTTTTCTTGACCGCGAC
CGCCAATCTTACCTTTTTTGATAAAATCAGCCAAACCTATCCCATCGCGGACAATCTCGGCTTTGTGCTGACGATCGCTG
TCGTGCTCTTTGGCGCGATGCTACTGATCACCACGCTGTTATCATCGTATCGCTATGTGCTAAAGCCTGTGTTGATTTTG

CTATTAATCATGGGCGCGGTGACCAGTTATTTTACTGACACTTATGGCACGGTCTATGATACGACCATGCTCCAAAATGC

CCTACAGACCGACCAAGCCGAGACCAAGGATCTATTAAACGCAGCGTTTATCATGCGTATCATTGGTTTGGGTGTGCTAC

CAAGTTTGCTTGTGGCTTTTGTTAAGGTGGATTATCCGACTTGGGGCAAGGGTTTGATGCGCCGATTGGGCTTGATCGTG

GCAAGTCTTGCGCTGATTTTACTGCCTGTGGTGGCGTTCAGCAGTCATTATGCCAGTTTCTTTCGCGTGCATAA

>Uncultured_bacterium_36

CAGCATACTTCTGTGTGGTACCGACGCTCGGTCAGTCCGTTTGTTCTTGTGGCGAGTGTTGCCGTTTTCTTGACCGCGAC

CGCCAATCTTACCTTTTTTGATAAAATCAGCCAAACCTATCCCATCGCGGACAATCTCGGCTTTGTGCTGACGATCGCTG

TCGTGCTCTTTGGCGCGATGCTACTGATCACCACGCTGTTATCATCGTATCGCTATGTGCTAAAGCCTGTGTTGATTTTG

CTATTAATCATGGGCGCGGTGACCAGTTATTTTACTGACACTTATGGCACGGTCTATGATACGACCATGCTCCAAAATGC

CCTACAGACCGACCAAGCCGAGACCAAGGATCTATTAAACGCAGCGTTTATCATGCGTATCATTGGTTTGGGTGTGCTAC

CAAGTTTGCTTGTGGCTTTTGTTAAGGTGGATTATCCGACTTGGGGCAAGGGTTTGATGCGCCGATTGGGCTTGATCGTG

GCAAGTCTTGCGCTGATTTTACTGCCTGTGGTGGCGTTCAGCAGTCATTATGCCAGTTTCTTTCGCGTGCATAA

>Uncultured_bacterium_37

CAGCATACTTCTGTGTGGTACCGACGCTCGGTCAGTCCGTTTGTTCTTGTGGCGAGTGTTGCCGTTTTCTTGACCGCGAC

CGCCAATCTTACCTTTTTTGATAAAATCAGCCAAACCTATCCCATCGCGGACAATCTCGGCTTTGTGCTGACGATCGCTG

TCGTGCTCTTTGGCGCGATGCTACTGATCACCACGCTGTTATCATCGTATCGCTATGTGCTAAAGCCTGTGTTGATTTTG

CTATTAATCATGGGCGCGGTGACCAGTTATTTTACTGACACTTATGGCACGGTCTATGATACGACCATGCTCCAAAATGC

CCTACAGACCGACCAAGCCGAGACCAAGGATCTATTAAACGCAGCGTTTATCATGCGTATCATTGGTTTGGGTGTGCTAC

CAAGTTTGCTTGTGGCTTTTGTTAAGGTGGATTATCCGACTTGGGGCAAGGGTTTGATGCGCCGATTGGGCTTGATCGTG

GCAAGTCTTGCGCTGATTTTACTGCCTGTGGTGGCGTTCAGCAGTCATTATGCCAGTTTCTTTCGCGTGCATAA

>Uncultured_bacterium_38

CAGCATACTTCTGTGTGGTACCGACGCTCGGTCAGTCCGTTTGTTCTTGTGGCGAGTGTTGCCGTTTTCTTGACCGCGAC

CGCCAATCTTACCTTTTTTGATAAAATCAGCCAAACCTATCCCATCGCGGACAATCTCGGCTTTGTGCTGACGATCGCTG

TCGTGCTCTTTGGCGCGATGCTACTGATCACCACGCTGTTATCATCGTATCGCTATGTGCTAAAGCCTGTGTTGATTTTG

CTATTAATCATGGGCGCGGTGACCAGTTATTTTACTGACACTTATGGCACGGTCTATGATACGACCATGCTCCAAAATGC

CCTACAGACCGACCAAGCCGAGACCAAGGATCTATTAAACGCAGCGTTTATCATGCGTATCATTGGTTTGGGTGTGCTAC

CAAGTTTGCTTGTGGCTTTTGTTAAGGTGGATTATCCGACTTGGGGCAAGGGTTTGATGCGCCGATTGGGCTTGATCGTG

GCAAGTCTTGCGCTGATTTTACTGCCTGTGGTGGCGTTCAGCAGTCATTATGCCAGTTTCTTTCGCGTGCATA

>Uncultured_bacterium_39

CAGCATACTTCTGTGTGGTACCGACGCTCGGTCAGTCCGTTTGTTCTTGTGGCGAGTGTTGCCGTTTTCTTGACCGCGAC

CGCCAATCTTACCTTTTTTGATAAAATCAGCCAAACCTATCCCATCGCGGACAATCTCGGCTTTGTGCTGACGATCGCTG

TCGTGCTCTTTGGCGCGATGCTACTGATCACCACGCTGTTATCATCGTATCGCTATGTGCTAAAGCCTGTGTTGATTTTG
CTATTAATCATGGGCGCGGTGACCAGTTATTTTACTGACACTTATGGCACGGTCTATGATACGACCATGCTCCAAAATGC
CCTACAGACCGACCAAGCCGAGACCAAGGATCTATTAAACGCAGCGTTTATCATGCGTATCATTGGTTTGGGTGTGCTAC
CAAGTTTGCTTGTGGCTTTTGTTAAGGTGGATTATCCGACTTGGGGCAAGGGTTTGATGCGCCGATTGGGCTTGATCGTG
GCAAGTCTTGCGCTGATTTTACTGCCTGTGGTGGCGTTCAGCAGTCATTATGCCAGTTTCTTTCGCGTGCATA

>Uncultured_bacterium_40
CAGCATACTTCTGTGTGGTACCGACGCTCGGTCAGTCCGTTTGTTCTTGTGGCGAGTGTTGCCGTTTTCTTGACCGCGAC
CGCCAATCTTACCTTTTTTGATAAAATCAGCCAAACCTATCCCATCGCGGACAATCTCGGCTTTGTGCTGACGATCGCTG
TCGTGCTCTTTGGCGCGATGCTACTGATCACCACGCTGTTATCATCGTATCGCTATGTGCTAAAGCCTGTGTTGATTTTG
CTATTAATCATGGGCGCGGTGACCAGTTATTTTACTGACACTTATGGCACGGTCTATGATACGACCATGCTCCAAAATGC
CCTACAGACCGACCAAGCCGAGACCAAGGATCTATTAAACGCAGCGTTTATCATGCGTATCATTGGTTTGGGTGTGCTAC
CAAGTTTGCTTGTGGCTTTTGTTAAGGTGGATTATCCGACTTGGGGCAAGGGTTTGATGCGCCGATTGGGCTTGATCGTG
GCAAGTCTTGCGCTGATTTTACTGCCTGTGGTGGCGTTCAGCAGTCATTATGCCAGTTTCTTTCGCGTGCATA

>Uncultured_bacterium_41
CAGCATACTTCTGTGTGGTACCGACGCTCGGTCAGTCCGTTTGTTCTTGTGGCGAGTGTTGCCGTTTTCTTGACCGCGAC

CGCCAATCTTACCTTTTTTGATAAAATCAGCCAAACCTATCCCATCGCGGACAATCTCGGCTTTGTGCTGACGATCGCTG

TCGTGCTCTTTGGCGCGATGCTACTGATCACCACGCTGTTATCATCGTATCGCTATGTGCTAAAGCCTGTGTTGATTTTG

CTATTAATCATGGGCGCGGTGACCAGTTATTTTACTGACACTTATGGCACGGTCTATGATACGACCATGCTCCAAAATGC

CCTACAGACCGACCAAGCCGAGACCAAGGATCTATTAAACGCAGCGTTTATCATGCGTATCATTGGTTTGGGTGTGCTAC

CAAGTTTGCTTGTGGCTTTTGTTAAGGTGGATTATCCGACTTGGGGCAAGGGTTTGATGCGCCGATTGGGCTTGATCGTG

GCAAGTCTTGCGCTGATTTTACTGCCTGTGGTGGCGTTCAGCAGTCATTATGCCAGTTTCTTTCGCGTGCATA

>Uncultured_bacterium_42

CAGCATACTTCTGTGTGGTACCGACGCTCGGTCAGTCCGTTTGTTCTTGTGGCGAGTGTTGCCGTTTTCTTGACCGCGAC

CGCCAATCTTACCTTTTTTGATAAAATCAGCCAAACCTATCCCATCGCGGACAATCTCGGCTTTGTGCTGACGATCGCTG

TCGTGCTCTTTGGCGCGATGCTACTGATCACCACGCTGTTATCATCGTATCGCTATGTGCTAAAGCCTGTGTTGATTTTG

CTATTAATCATGGGCGCGGTGACCAGTTATTTTACTGACACTTATGGCACGGTCTATGATACGACCATGCTCCAAAATGC

CCTACAGACCGACCAAGCCGAGACCAAGGATCTATTAAACGCAGCGTTTATCATGCGTATCATTGGTTTGGGTGTGCTAC

CAAGTTTGCTTGTGGCTTTTGTTAAGGTGGATTATCCGACTTGGGGCAAGGGTTTGATGCGCCGATTGGGCTTGATCGTG

GCAAGTCTTGCGCTGATTTTACTGCCTGTGGTGGCGTTCAGCAGTCATTATGCCAGTTTCTTTCGCGTGCATA

>Uncultured_bacterium_43

CAGCATACTTCTGTGTGGTACCGACGCTCGGTCAGTCCGTTTGTTCTTGTGGCGAGTGTTGCCGTTTTCTTGACCGCGAC

CGCCAATCTTACCTTTTTTGATAAAATCAGCCAAACCTATCCCATCGCGGACAATCTCGGCTTTGTGCTGACGATCGCTG

TCGTGCTCTTTGGCGCGATGCTACTGATCACCACGCTGTTATCATCGTATCGCTATGTGCTAAAGCCTGTGTTGATTTTG

CTATTAATCATGGGCGCGGTGACCAGTTATTTTACTGACACTTATGGCACGGTCTATGATACGACCATGCTCCAAAATGC

CCTACAGACCGACCAAGCCGAGACCAAGGATCTATTAAACGCAGCGTTTATCATGCGTATCATTGGTTTGGGTGTGCTAC

CAAGTTTGCTTGTGGCTTTTGTTAAGGTGGATTATCCGACTTGGGGCAAGGGTTTGATGCGCCGATTGGGCTTGATCGTG

GCAAGTCTTGCGCTGATTTTACTGCCTGTGGTGGCGTTCAGCAGTCATTATGCCAGTTTCTTTCGCGTGCATA

>Uncultured_bacterium_44

CAGCATACTTCTGTGTGGTACCGACGCTCGGTCAGTCCGTTTGTTCTTGTGGCGAGTGTTGCCGTTTTCTTGACCGCGAC

CGCCAATCTTACCTTTTTTGATAAAATCAGCCAAACCTATCCCATCGCGGACAATCTCGGCTTTGTGCTGACGATCGCTG

TCGTGCTCTTTGGCGCGATGCTACTGATCACCACGCTGTTATCATCGTATCGCTATGTGCTAAAGCCTGTGTTGATTTTG

CTATTAATCATGGGCGCGGTGACCAGTTATTTTACTGACACTTATGGCACGGTCTATGATACGACCATGCTCCAAAATGC

CCTACAGACCGACCAAGCCGAGACCAAGGATCTATTAAACGCAGCGTTTATCATGCGTATCATTGGTTTGGGTGTGCTAC

CAAGTTTGCTTGTGGCTTTTGTTAAGGTGGATTATCCGACTTGGGGCAAGGGTTTGATGCGCCGATTGGGCTTGATCGTG

GCAAGTCTTGCGCTGATTTTACTGCCTGTGGTGGCGTTCAGCAGTCATTATGCCAGTTTCTTTCGCGTGCAT

>Uncultured_bacterium_45

CAGCATACTTCTGTGTGGTACCGACGCTCGGTCAGTCCGTTTGTTCTTGTGGCGAGTGTTGCCGTTTTCTTGACCGCGAC

CGCCAATCTTACCTTTTTTGATAAAATCAGCCAAACCTATCCCATCGCGGACAATCTCGGCTTTGTGCTGACGATCGCTG

TCGTGCTCTTTGGCGCGATGCTACTGATCACCACGCTGTTATCATCGTATCGCTATGTGCTAAAGCCTGTGTTGATTTTG

CTATTAATCATGGGCGCGGTGACCAGTTATTTTACTGACACTTATGGCACGGTCTATGATACGACCATGCTCCAAAATGC

CCTACAGACCGACCAAGCCGAGACCAAGGATCTATTAAACGCAGCGTTTATCATGCGTATCATTGGTTTGGGTGTGCTAC

CAAGTTTGCTTGTGGCTTTTGTTAAGGTGGATTATCCGACTTGGGGCAAGGGTTTGATGCGCCGATTGGGCTTGATCGTG

GCAAGTCTTGCGCTGATTTTACTGCCTGTGGTGGCGTTCAGCAGTCATTATGCCAGTTTCTTTCGCGTGCAT

>Uncultured_bacterium_46

CAGCATACTTCTGTGTGGTACCGACGCTCGGTCAGTCCGTTTGTTCTTGTGGCGAGTGTTGCCGTTTTCTTGACCGCGAC

CGCCAATCTTACCTTTTTTGATAAAATCAGCCAAACCTATCCCATCGCGGACAATCTCGGCTTTGTGCTGACGATCGCTG

TCGTGCTCTTTGGCGCGATGCTACTGATCACCACGCTGTTATCATCGTATCGCTATGTGCTAAAGCCTGTGTTGATTTTG

CTATTAATCATGGGCGCGGTGACCAGTTATTTTACTGACACTTATGGCACGGTCTATGATACGACCATGCTCCAAAATGC

CCTACAGACCGACCAAGCCGAGACCAAGGATCTATTAAACGCAGCGTTTATCATGCGTATCATTGGTTTGGGTGTGCTAC

CAAGTTTGCTTGTGGCTTTTGTTAAGGTGGATTATCCGACTTGGGGCAAGGGTTTGATGCGCCGATTGGGCTTGATCGTG

GCAAGTCTTGCGCTGATTTTACTGCCTGTGGTGGCGTTCAGCAGTCATTATGCCAGTTTCTTTCGCGTG

>Uncultured_bacterium_47

TCTGTGTGGTACCGACGCTCGGTCAGTCCGTTTGTTCTTGTGGCGAGTGTTGCCGTTTTCTTGACCGCGACCGCCAATCT

TACCTTTTTTGATAAAATCAGCCAAACCTATCCCATCGCGGACAATCTCGGCTTTGTGCTGACGATCGCTGTCGTGCTCT

TTGGCGCGATGCTACTGATCACCACGCTGTTATCATCGTATCGCTATGTGCTAAAGCCTGTGTTGATTTTGCTATTAATC

ATGGGCGCGGTGACCAGTTATTTTACTGACACTTATGGCACGGTCTATGATACGACCATGCTCCAAAATGCCCTACAGAC

CGACCAAGCCGAGACCAAGGATCTATTAAACGCAGCGTTTATCATGCGTATCATTGGTTTGGGTGTGCTACCAAGTTTGC

TTGTGGCTTTTGTTAAGGTGGATTATCCGACTTGGGGCAAGGGTTTGATGCGCCGATTGGGCTTGATCGTGGCAAGTCTT

GCGCTGATTTTACTGCCTGTGGTGGCGTTCAGCAGTCATTATGCCAGTTTCTTTCGCGTGCATA

>Uncultured_bacterium_48

CAGCATACTTCTGTGTGGTACCGACGCTCGGTCAGTCCGTTTGTTCTTGTGGCGAGTGTTGCCGTTTTCTTGACCGCGAC

CGCCAATCTTACCTTTTTTGATAAAATCAGCCAAACCTATCCCATCGCGGACAATCTCGGCTTTGTGCTGACGATCGCTG

TCGTGCTCTTTGGCGCGCTGCTACTGATCACCACGCTGTTATCATCGTATCGCTATGTGCTAAAGCCTGTGTTGATTTTG

CTATTAATCATGGGCGCGGTGACCAGTTATTTTACTGACACTTATGGCACGGTCTATGATACGACCATGCTCCAAAATGC

CCTACAGACCGACCAAGCCGAGACCAAGGATCTATTAAACGCAGCGTTTATCATGCGTATCATTGGTTTGGGTGTGCTAC

CAAGTTTGCTTGTGGCTTTTGTTAAGGTGGATTATCCGACTTGGGGCAAGGGTTTGATGCGCCGATTGGGCTTGATCGTG

GCAAGTCTTGCGCTGATTTTACTGCCTGTGGTGGCGTTCAGCAGTCATTATGCCAGTTTCTTTCGCG

>Uncultured_bacterium_49

CAGCATACTTCTGTGTGGTACCGACGCTCGGTCAGTCCGTTTGTTCTTGTGGCGAGTGTTGCCGTTTTCTTGACCGCGAC

CGCCAATCTTACCTTTTTTGATAAAATCAGCCAAACCTATCCCATCGCGGACAATCTCGGCTTTGTGCTGACGATCGCTG

TCGTGCTCTTTGGCGCGATGCTACTGATCACCACGCTGTTATCATCGTATCGCTATGTGCTAAAGCCTGTGTTGATTTTG

CTATTAATCATGGGCGCGGTGACCAGTTATTTTACTGACACTTATGGCACGGTCTATGATACGACCATGCTCCAAAATGC

CCTACAGACCGACCAAGCCGAGACCAAGGATCTATTAAACGCAGCGTTTATCATGCGTATCATTGGTTTGGGTGTGCTAC

CAAGTTTGCTTGTGGCTTTTGTTAAGGTGGATTATCCGACTTGGGGCAAGGGTTTGATGCGCCGATTGGGCTTGATCGTG

GCAAGTCTTGCGCTGATTTTACTGCCTGTGGTGGCGTTCAGCAGTCATTATGCCAGTTTCTTTC

>Uncultured_bacterium_50

CAGCATACTTCTGTGTGGTACCGACGCTCGGTCAGTCCGTTTGTTCTTGTGGCGAGTGTTGCCGTTTTCTTGACCGCGAC

CGCCAATCTTACCTTTTTTGATAAAATCAGCCAAACCTATCCCATCGCGGACAATCTCGGCTTTGTGCTGACGATCGCTG

TCGTGCTCTTTGGCGCGATGCTACTGATCACCACGCTGTTATCATCGTATCGCTATGTGCTAAAGCCTGTGTTGATTTTG

CTATTAATCATGGGCGCGGTGACCAGTTATTTTACTGACACTTATGGCACGGTCTATGATACGACCATGCTCCAAAATGC

CCTACAGACCGACCAAGCCGAGACCAAGGATCTATTAAACGCAGCGTTTATCATGCGTATCATTGGTTTGGGTGTGCTAC

CAAGTTTGCTTGTGGCTTTTGTTAAGGTGGATTATCCGACTTGGGGCAAGGGTTTGATGCGCCGATTGGGCTTGATCGTG

GCAAGTCTTGCGCTGATTTTACTGCCTGTGGTGGCGTTCAGCAGTCATTATGCCAGTTTCTTT

>Uncultured_bacterium_50

ACTTCTGTGTGGTACCGACGCTCGGTCAGTCCGTTTGTTCTTGTGGCGAGTGTTGCCGTTTTCTTGACCGCGACCGCCAA

TCTTACCTTTTTTGATAAAATCAGCCAAACCTATCCCATCGCGGACAATCTCGGCTTTGTGCTGACGATCGCTGTCGTGC

TCTTTGGCGCGATGCTACTGATCACCACGCTGTTATCATCGTATCGCTATGTGCTAAAGCCTGTGTTGATTTTGCTATTA

ATCATGGGCGCGGTGACCAGTTATTTTACTGACACTTATGGCACGGTCTATGATACGACCATGCTCCAAAATGCCCTACA

GACCGACCAAGCCGAGACCAAGGATCTATTAAACGCAGCGTTTATCATGCGTATCATTGGTTTGGGTGTGCTACCAAGTT

TGCTTGTGGCTTTTGTTAAGGTGGATTATCCGACTTGGGGCAAGGGTTTGATGCGCCGATTGGGCTTGATCGTGGCAAGT

CTTGCGCTGATTTTACTGCCTGTGGTGGCGTTCAGCAGTCATTATGCCAGTTTCTTTC
