# Supplementary figures and images for: Molecular Detection of Colistin Resistance mcr-1 Gene in Multidrug-Resistant Escherichia coli Isolated from Chicken
Source: Antibiotics (Basel). 2022 Jan 13;11(1):97. doi: 10.3390/antibiotics11010097 (PMC8772701; doi:10.3390/antibiotics11010097)

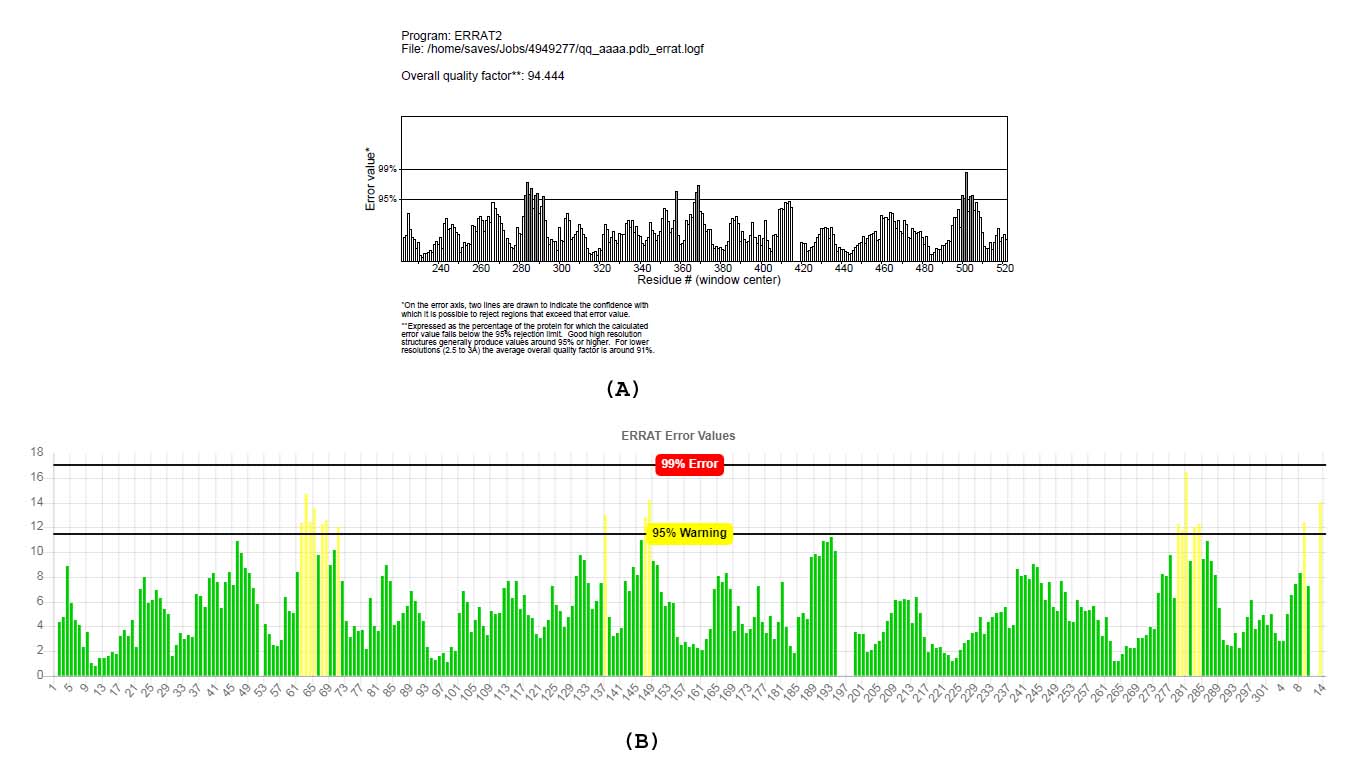

Supplement: Supplementary file 1 [file antibiotics-11-00097-s001.zip › Supplementary Figure S1.jpeg]

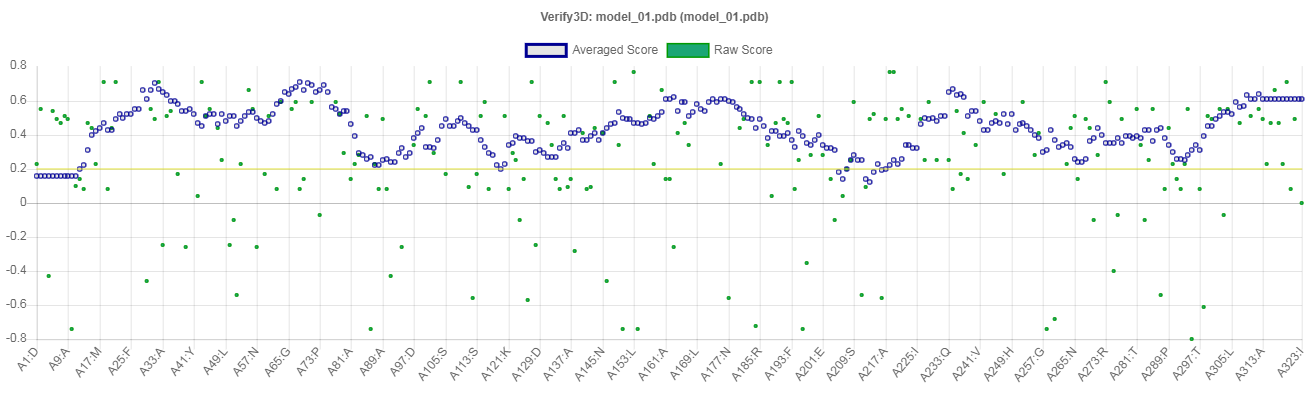

Supplement: Supplementary file 1 [file antibiotics-11-00097-s001.zip › Supplementary Figure S2.png]
